# Genome-Wide Identification and Analysis of P-Type Plasma Membrane H^+^-ATPase Sub-Gene Family in Sunflower and the Role of *HHA4* and *HHA11* in the Development of Salt Stress Resistance

**DOI:** 10.3390/genes11040361

**Published:** 2020-03-27

**Authors:** Zongchang Xu, Prince Marowa, Han Liu, Haina Du, Chengsheng Zhang, Yiqiang Li

**Affiliations:** 1 Marine Agriculture Research Center, Tobacco Research Institute of Chinese Academy of Agricultural Sciences, Qingdao 266101, China; xuzongchang@caas.cn (Z.X.); zhangchengsheng@caas.cn (C.Z.); 2 Crop Science Department, University of Zimbabwe, Harare 00263, Zimbabwe; pmarowa@agric.uz.ac.zw; 3 College of Agriculture, Qingdao Agricultural University, Qingdao 266109, China; lh17864236997@163.com (H.L.); duhainah@163.com (H.D.)

**Keywords:** P-type plasma membrane ATPase, salt stress, gene expression, bioinformatics analysis, sunflower

## Abstract

The P-type plasma membrane (PM) H^+^-ATPase plays a major role during the growth and development of a plant. It is also involved in plant resistance to a variety of biotic and abiotic factors, including salt stress. The PM H^+^-ATPase gene family has been well characterized in *Arabidopsis* and other crop plants such as rice, cucumber, and potato; however, the same cannot be said in sunflower (*Helianthus annuus*). In this study, a total of thirteen PM H^+^-ATPase genes were screened from the recently released sunflower genome database with a comprehensive genome-wide analysis. According to a systematic phylogenetic classification with a previously reported species, the sunflower PM H^+^-ATPase genes (*HHAs*) were divided into four sub-clusters (I, II, IV, and V). In addition, systematic bioinformatics analyses such as gene structure analysis, chromosome location analysis, subcellular localization predication, conserved motifs, and *Cis*-acting elements of promoter identification were also done. Semi-quantitative PCR analysis data of *HHAs* in different sunflower tissues revealed the specificity of gene spatiotemporal expression and sub-cluster grouping. Those belonging to sub-cluster I and II exhibited wide expression in almost all of the tissues studied while sub-cluster IV and V seldom showed expression. In addition, the expression of *HHA4*, *HHA11,* and *HHA13* was shown to be induced by salt stress. The transgenic plants overexpressing *HHA4* and *HHA11* showed higher salinity tolerance compared with wild-type plants. Further analysis showed that the Na^+^ content of transgenic *Arabidopsis* plants decreased under salt stress, which indicates that PM H^+^ ATPase participates in the physiological process of Na^+^ efflux, resulting in salt resistance of the plants. This study is the first to identify and analyze the sunflower PM H^+^ ATPase gene family. It does not only lay foundation for future research but also demonstrates the role played by *HHAs* in salt stress tolerance.

## 1. Introduction

P-type plasma membrane (PM) H^+^-ATPase belongs to the type IIIA sub-gene family of the P-type ATPase super-gene family [1]. P-type ATPase, which is also called E_1_-E_2_ ATPases, are widely found/present in the plasma membranes of many bacterial species, archaea, and eukaryotes [2,3]. They have the name “P-type” mainly because the protein can be phosphorylated [4].

P-type ATPases are a very large membrane protein family. The super-family can be divided into five main branches (Type I ATPase through Type V ATPase), according to the conserved sequence domain of the 159 eukarya P-type ATPases [1]. According to the different transporting substrates, these five types are subdivided into 10 different sub-families. Type I branch of P-type ATPases includes two sub-families, Type IA and Type IB. The sub-family of Type IA is a K^+^ transporter and Type IB is for heavy metal ions (Cu^2+^ and Cd^2+^). Type II ATPases branch is divided into four sub-families, exhibiting various substrates (Type IIA, endoplasmic reticulum [ER]-type Ca^2+^; Type IIB, auto inhibited Ca^2+^; Type IIC, H^+^/K^+^_,_ and Na^+^/K^+^; Type IID, Ca^2+^ or Na^+^). Type IIIA sub-family contains plasma membrane H^+^-ATPases, while the proteins belonging to type IIIB transport Mg^2+^. Type IV ATPases are essential for maintaining the homeostasis of lipid bilayers; however, the function of type V ATPases is still unknown [1,5]. Among them, Na^+^/K^+^-ATPase was the first P-type ATPase to be discovered [6]. This P-ATPase transports H^+^ to the extracellular membrane by coupling hydrolysis of ATP generating a proton motive force, which is beneficial to a large number of secondary transporters to move metabolites or ions against the concentration gradient [7,8]

P-type PM H^+^-ATPases are thought to participate in many physiological activities such as mineral nutrient transport in roots, regulation of cytoplasmic pH, metabolite translocation, cell growth, and organ movement, and to play major roles in the growth and development of plant [3,9,10,11]. It plays a similarly critical role in bacteria, archaea, fungi, and plants, but not in invertebrates and vertebrates [9]. In addition, PM H^+^-ATPase enzymes also play important roles in the development of biotic and abiotic stress tolerance/resistance in plants, particularly in the development of salt stress tolerance/resistance [10,11,12]. Although the mechanism of salt tolerance in plants is very complex, it is necessary to reduce the concentration of Na^+^ in the cells, thus reducing harm due to salt stress [13]. The transport of Na^+^ against the electrochemical gradient from the cytosol into vacuole or apoplast across the plasma membrane is facilitated by the Na^+^/H^+^ antiporter [14], with the proton concentration gradient generated by the proton pump [15]. In plants, the P-type PM H^+^-ATPase is an example of such a proton pump [15]. Several studies have indicated that the expression of the PM H^+^-ATPase genes could be induced by salt stress [16]. In this study, Binzel reported that the accumulation of PM H^+^-ATPase was increased in the roots and leaves of tomato plants after 24 h of exposure to NaCl stress [17]. NaCl could also induce the accumulation of *LHA8* transcripts [15]. In addition, the roots of transgenic tobacco plants overexpressing Δ*PMA4*, which was a dominant mutant of H^+^-ATPase *PMA4*, also grew better than those of untransformed plants under saline conditions [18]. Furthermore, the root length, germination rate, and biomass of transgenic *Arabidopsis* plants that overexpressed *PeHA1* derived from *P. euphratica* also showed greater growth habits under NaCl stress [19]. All these studies demonstrate the role played by PM H^+^-ATPase in the development of salt tolerance.

The P-type PM H^+^-ATPases have several conserved domains that could be used to screen or identify such proteins. The most conserved domain among all H^+^-ATPases is the P-domain (phosphorylation domain) [9]. The aspartate residue (D) located in the DKTGTLT conserved motif is phosphorylated by ATP, which could be used as a characteristic feature for PM H^+^-ATPase identification [8,20]. The P-domain is one of the cytoplasmic domains of PM H^+^-ATPase. The other cytoplasmic domains are A-domain (actuator domain) and N domain (nucleotide-binding domain) [2]. The typical sequence of A-domain is Thr-GlyGlu (TGE), which is located in the N-terminal cytoplasmic loop [21]. The N-domain is an insertion into the P-domain, which binds ATP and phosphorylates the P-domain. The conserved amino sequence of the N-domain is KGAP, which is located in the second and larger cytoplasmic loop [9,20]. These are the three cytoplasmic domains that show a high degree of conservation. In addition, PM H^+^-ATPases also contain two variable membrane-embedded domains—the T-domain (transport domain) and the S-domain (specific structure support domain)—which are formed with the N-terminal and C-terminal transmembrane helices, respectively [2,9]. In addition, the R-domain (regulatory domain), which is located at the C-terminal region (about 100 amino acids), was reported to be the autoinhibitory domain that is thought to be involved in the regulation of proton pumping [20]. The mechanism is the reciprocal phosphorylation of the penultimate threonine residue of the C-terminal regulatory domain to release its own inhibitory effect. Subsequently, the binding of 14-3-3 proteins results in pump activation [22]. In addition, previous studies reported that the PM H^+^-ATPase activity could also be affected by other residue phosphorylation [23], which indicates that the regulation of these proton pump activities is very complicated. For example, the PPI (proton pump interactor), which was identified in *A. thaliana* [24] and *Solanum tuberosum* [25], is a novel interaction partner of PM H^+^-ATPase. The activity of the proton pump was increased when it interacted with PPI at the C-terminus in vitro [26].

The members of P-type PM ATPase in many organisms have been identified in succession. Previous genome-wide analysis studies reported that there are 10 and 11 PM H^+^-ATPase gene families in *Oryza sativa* and *Arabidopsis thaliana*, respectively [5]. There are also 12 in *Lycopersicon esculentum* [15], 4 in *Zea mays* [27], 10 in *Cucumis sativus* [28], 7 in *Solanum tuberosum* L [26], and 9 in *Nicotiana plumbaginifolia* [16]. Phylogenetic analysis further divided the PM H^+^-ATPase into five sub-clusters according to the predicted amino acid sequences [5,16]. Sunflower is an important oil and food crop in China and many other countries. In China, its production is mainly restricted to areas in Neimenggu, Ningxia, and Gansu Provinces, where most of the land is threatened by salinity. The latest release of sunflower genomic data [29] enables researchers to identify gene families in the crop and study their biological functions. The objective of this research work is to study the PM H^+^-ATPase genes (*HHAs*) from the recently released genome database of *H. annuus* using systematic bioinformatics analysis and spatiotemporal expression patterns of *HHAs* in sunflower tissues. Furthermore, the subcellular localization and the expression profiling of *HHAs* response to saline stresses were also studied. Finally, the functions of *HHAs* in the development of salt tolerance were studied by overexpressing *HHA4* and *HHA11* in *Arabidopsis*.

## 2. Materials and Methods

### 2.1. Plant Materials and Growth Conditions

The plant materials included sunflower plants (XRQ) [29], *Arabidopsis* ((Columbia) wild-type (WT) plants and two transgenic lines (*HHA4*-OE, and *HHA11*-OE)), as well as *Nicotiana benthamiana* plants. All the plants were grown in a culture room described in our previous studies [30,31] with a relative constant temperature of 23 ± 1 °C and 16/8 h photoperiod (light/dark). The humidity was controlled at approximately 60%. The sunflower plants were used for the expression pattern analysis of PM H^+^-ATPase genes in different tissues and cloning *HHA* genes, while *Nicotiana benthamiana* plants were used for subcellular localization determination of HHA proteins. The WT and transgenic *Arabidopsis* plants were used for the salt tolerance tests.

### 2.2. Identification of P-Type PM H^+^-ATPases Sub-Gene Family Members in Sunflower

The method used to identify the P-type PM H^+^-ATPases ion pump sub-gene family members was based on a previous study [31]. The protein amino acid and nucleotide sequences of 11 published *Arabidopsis thaliana* P-type PM H^+^-ATPases [5] that were used as queries were download from the TAIR database. A genome-wide BLAST of PM H^+^-ATPases sub-gene family members was screened in the *Helianthus annuus* L. genome database [29] with the parameters id% >50% and E-value <10^−15^ [31]. Two web tools, Pfam and SMART databases, were used to identify the DKTGT[L/I/V/M][T/I] (P domain) and ProSite PS00154 conserved domains [3] of all the candidate PM H^+^-ATPase proteins after removing redundant sequence. Only genes that contained both these two conserved domains were regarded as sunflower P-type PM H^+^-ATPases genes and used to further analysis. The protein sequences, coding sequence (CDS), genomic sequences, coding sequence (CDS), and 2 kb range of promoter sequence of PM H^+^-ATPases were downloaded from the sunflower genome database [29]. Physicochemical parameters of each protein were calculated with web-tool ProtParam. The sub-cellular localizations were predicted with web-tools ProtComp 9.0 and Plant-mPLoc database [32].

### 2.3. Phylogenetic Analysis

P-type PM H^+^-ATPases protein sequence multiple alignments of *Helianthus annuus* (HHA), *A. thaliana* (AHA), *O. sativa* (OSA), *N. plumbaginifolia* (PMA), and *S. tuberosum* Phureja (PHA) [26] were aligned by ClustalW program with a gap extension penalty of 0.1. Then, a phylogenetic tree was constructed using Mega 6.0 [33]. The neighbor-joining (NJ) algorithm, bootstrap analysis with 1000 replicates, and the Poisson model were adopted.

### 2.4. Gene Structure Analysis and Conserved Motif Identification

The GSDS (Gene Structure Display Server) tool was used to display the PM H^+^-ATPases genes exon/intron structure [34]. The PM H^+^-ATPases protein conserved motif structures were identified with the MEME (multiple expectation maximization for motif elicitation) web tool [35] with the parameters 15–30 residues in motif width and a maximum of 12 motifs.

### 2.5. Cis-Acting Elements Analysis of PM H^+^-ATPase Genes Promoter Region

The 2000-bp sequences of sunflower PM H^+^-ATPase gene promoters were extracted from the *H. annuus* L. genome database [29]. The promoter *Cis*-acting elements were detected and identified using the PLACE database [36]. The distribution of salt stress-related *Cis*-acting elements were visualized by GSDS 2.0 [34].

### 2.6. Expression Pattern Analysis of PM H^+^-ATPase Genes in Different Tissues

Semi-quantitative PCR was used to determine the spatiotemporal expression patterns of PM H^+^-ATPase genes. A total of 14 different sunflower tissues, including young cotyledons, senescent cotyledons, seedling leaves, young phloem, mature leaves, senescent leaves, petals, sepals, pollens, young seeds, adult stems, piths, petioles, and young roots, were collected from 2-week-old seedlings and 8-week-old adult plants grown in the culturing room. Samples were quickly frozen in liquid nitrogen and stored at −80 °C until they were needed for RNA extraction. Three independent replicates of each tissue were performed. RNA extraction and cDNA synthesis were performed, as described in [31]. The volume of semi-quantitative PCR reaction mixture was 20 μL, including 1 μL cDNA template, 10 μL 2× Taq DNA Polymerase (P102-d1, Vazyme, China), and 0.6 μL of each of the primers (10 mM). Reaction mixtures were filled up to 20 μL with double distilled water. The semi-quantitative PCR conditions were as follows: 94 °C for 5 min, followed by 28 cycles of 94 °C for 30 s, 60 °C for 30 s, and 72 °C for 40 s, and finally, 72 °C for 10. The products of semi-quantitative PCR were detected with 2% agarose gel stained with ethidium bromide. A single product of the correct size for each gene verified the successful amplification and the specificity of the primer pairs. The sunflower *HACTIN* gene (HanXRQChr14g0446641) was used as the reference gene. The primers used in this study are shown in Supplementary File S1.

### 2.7. Expression Profiles of PM H^+^-ATPase Genes under Salt Stress

To analyze the expression profiles of H^+^-ATPase genes under salt stress, 2-week-old seedlings of sunflower plants were subjected to NaCl treatments. The seedlings were watered with 100 mL of 150 mM NaCl. Leaves were collected from the NaCl-treated seedling at 0, 0.5, 2, 4, 8, 12, 18, and 24 h after treatment initiation, and immediately froze in liquid nitrogen then stored at −80 °C for RNA extraction. The samples collected at 0 h were used as the control (CK). Each treatment contained three biological replicates. RNA extraction and cDNA synthesis methods were described above. The qRT-PCR analysis was performed with an Applied Biosystems 7500 Real-Time PCR system (Thermo Fisher Scientific) according to the manufacturer’s instructions. The 2^−∆∆Ct^ method was used to calculate the PM H^+^-ATPase gene expression levels [37], which normalized with the sunflower *HACTIN* gene described above. The volume of qRT–PCR reactions was 20 μL, including 1 μL cDNA template, 10 μL 2× SYBR green mix (Vazyme, Q321, China), 0.4 μL ROX reference dye, and 1.2 μL primer mixture (10 mM). Reaction mixtures were filled up to 20 μL with water. The qRT–PCR conditions were as follows: 50 °C for 2 min, and then 95 °C for 5 min, followed by 40 cycles of 95 °C for 10 s and 60 °C for 35 s. Each qRT–PCR was performed in triplicate. The qRT–PCR primers are listed in Supplementary File S1.

### 2.8. Subcellular Localization Determination of HHA Proteins

The full-length CDS sequences of *HHA1*, *HHA4,* and *HHA11* were amplified by gene-specific primers (Supplementary File S1) using 2× TransStart^®^ Fast Pfu PCR Super Mix (AS221-01, Transgen, China), and then cloned into the binary pCAM35tlegfps2#4 vector [30] in the space between *Kpn1* and *BamH1* to generate 35S::*HHAs*-GFP fusion proteins using a ClonExpress Ultra One Step Cloning Kit (Vazyme, C115-01, China). The positive clones were transferred into *Agrobacterium tumefaciens* strain GV3101 for transient expression in 3-week-old *Nicotiana benthamiana* plants using the infiltration method [38]. The 35S::AtCESA1-RFP fusion protein was used as a plasma membrane-anchored marker [31]. GFP and RFP signals were observed with Leica TCS SP8 (Mannheim, Germany) confocal laser scanning microscopy.

### 2.9. Plant Transformation and Salt Tolerance Test

#### 2.9.1. Vector Construction, Arabidopsis Transformation, and Positive Transgenic Plants Identification

The full-length CDS of *HHA4* and *HHA11* were amplified with gene-specific primers (Supplementary File S1) and cloned into the 35S promoter-driven vector pCam35tlegfps2#4 with *KpnI* and *XbaI* sites [30]. *Agrobacterium tumefaciens* strain GV3101 was used to transform the *Arabidopsis* wild type plants using the floral dip method [39]. The expression level of the heterologous gene in the transgenic plants screened on half-strength MS medium (1/2 MS medium, which contained 15 mg/L hygromycin) was detected by semi-quantitative PCR analysis. The *AtACTIN2* gene was used as a positive control. The wild-type and homologous T_2_ transgenic plants were used for salt tolerance experiments.

#### 2.9.2. Salt Tolerant Experiment

Seeds of WT, *HHA4,* and *HHA11* transgenic T_2_ homozygous lines were sprinkled on 1/2 MS medium after surface sterilization [30], and placed in the culture/growth room. Five days later, seedlings with basically the same root length were then transferred to 1/2 MS medium that contained different concentrations of NaCl (0, 100, and 150 mM). Root length and ion content were measured after 2 weeks.

#### 2.9.3. Root Length Measurement

After salt treatment, the main root length data was obtained from ten seedlings of each treatment. Images were captured with a Canon 5D Mark III digital camera. Image J software was used to measure the main root length [40]. The root length data were analyzed with the ANOVA method using the SPSS software and, at *p <* 0.05, differences were considered statistically significant.

#### 2.9.4. Na^+^ Content Determination

Salt treated and control seedlings were collected from the media and thoroughly flushed with distilled water to remove impurities. The samples were then heated at 120 °C for 30 min and dried at 80 °C overnight to a constant weight. Determination of Na^+^ content was done according to a previous method with minor modification [41]. In brief, the plant samples were ground into powder and passed through a 0.5 mm sieve. Each 0.25 g of plant tissues was digested in 5 mL HNO_3_ at 110 °C for 6 h (until a colorless liquid was obtained). The solution was allowed to cool down and diluted to 10 mL with deionized water. The Na^+^ content was measured with a Perkin-Elmer Model 360 atomic absorption spectrophotometer.

## 3. Results

### 3.1. Identification of P-Type PM H^+^-ATPases Sub-Gene Family Members of Sunflower

A total of 82 non-redundant *AtHA* homologous genes were detected from the sunflower genome database by the genome-wide BLAST analysis. However, after the typical conservative domain analysis (SMART and Pfam tools), only 13 candidate P-type PM H^+^-ATPase genes (*HHAs*) of sunflower were identified. All of these predicted *HHAs* showed orthologous genes of *A. thaliana*. The identity ranged from 78.80% to 92.36% (Table 1). The length of PM H^+^-ATPases proteins varied from 851 to 1050 amino acids, with an average of 952. Sub-cellular localization of all of the 13 PM H+-ATPases proteins was predicated to be plasma membrane-anchored. The other detailed parameter information of PM H^+^-ATPases proteins or genes such as accession number, chromosome and genomic location, orthologous of *Arabidopsis*, protein length, intron numbers, isoelectric point (PI), molecular weight (MW), and prediction of subcellular location are listed in Table 1. The protein sequence, genomic sequences, and CDS of PM H^+^-ATPases are shown in Supplementary Files S2–S4, respectively.

### 3.2. Phylogenetic Analysis

**Figure 1:**
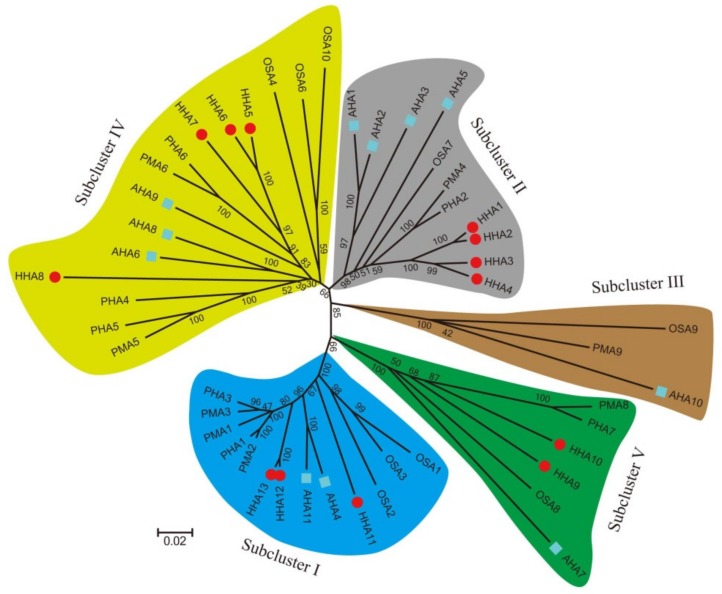
Phylogenetic tree of PM H^+^-ATPase genes in several plant species. The PM H^+^-ATPase amino acid sequences derived from *Nicotiana plumbaginifolia* (PMA1-PMA6, PMA8, and PMA9), *Oryza sativa* (OSA1-OSA10), *Solanum tuberosum* Phureja (PHA1-PHA7), *Arabidopsis thaliana* (AHA1-AHA11) were used to construct the phylogenetic tree with Mega 6.0 using the neighbor-joining method. Bootstrap analysis with 1000 replicates was used to evaluate the significance of the nodes. For the phylogenetic tree, lines with different colors indicated different sub-clusters of PM H^+^-ATPase. The blue square indicated *Arabidopsis,* and the red circle indicated sunflower.

Protein sequences of PM H^+^-ATPase derived from *A. thaliana*, *O. sativa*, *N. plumbaginifolia*, and *S. tuberosum* Phureja are listed in Supplementary File S5. According to the phylogenetic analysis results, the PM H^+^-ATPase proteins were grouped into five sub-clusters (Figure 1). However, HHA proteins were only grouped into sub-clusters I, II, IV, and V; no members of HHAs fell into sub-cluster III (Figure 1). The *HHA* genes were unevenly distributed in these four sub-clusters. The sub-clusters II and IV both contained four *HHA* genes, followed by sub-cluster I, which contained three members. Sub-cluster V had two members of the HHA gene family only. No matter how many members of *HHA* genes were grouped into the sub-cluster, the genetic relationship among members was very close except for sub-cluster IV. *HHA5*, *HHA6,* and *HHA7* showed a relatively close relationship; however, *HHA8* was far away from them (Figure 1).

### 3.3. Gene Structure Analysis, Conserved Motif Identification, and Transmembrane Analysis of Sunflower PM H^+^-ATPases

**Figure 2:**
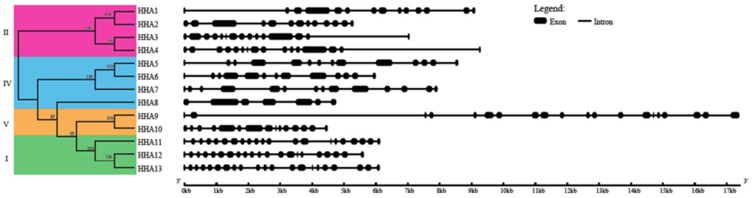
Gene structures of *HHA* genes in sunflower. The full-length CDS sequence of *HHA* genes were analyzed and displayed. The black rectangles represent exons, while black lines show introns.

The structural diversity and potential evolutionary relationship of the sunflower PM H^+^-ATPase genes were researched by studying the exon–intron structure. Gene structure analysis showed that the numbers and positions of introns in the PM H^+^-ATPase genes were diverse, and the numbers ranged from 6 (*HHA8*) to 20 (*HHA11*, *HHA12,* and *HHA13*) (Table 1, Figure 2). Among these genes, only *HHA8* contained 6 introns, and the others contained more than 12 introns. The intron numbers and exon–intron structures in each sub-cluster supported their close phylogenetic relationships and subgroup classifications. However, there were some exceptions. *HHAs* in the sub-clusters I and IV showed similar exon–intron structures (except *HHA8* in sub-clusters IV). *HHA9* and *HHA10* which fell into sub-clusters V exhibited exon–intron structure variation. In addition, the structures of exon–intron in sub-cluster II were divided into two types in which *HHA1* and *HHA2* showed similar structure, which was, however, different from that of *HHA3* and *HHA4,* which also showed a similar structure (Figure 2). According to the exon–intron distribution data, the gene structures are basically conserved in each sub-cluster, except for sub-cluster V, which showed similar evolutionary situation compared with the phylogenetic analysis. Further, conserved protein motif analysis of PM H^+^-ATPase proteins with MEME is shown in Supplementary File S6. A total of twelve conserved motifs were identified. All the conserved motifs were found to be located in the N-terminal and the middle region of the PM H^+^-ATPase proteins. All the thirteen PM H^+^-ATPase proteins contained these 12 motifs, indicating that conserved motifs analysis could not well distinguish the protein structure variation of this gene subfamily.

**Figure 3:**
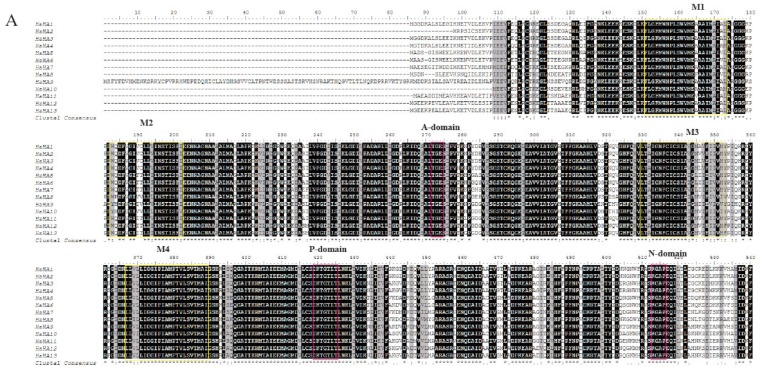

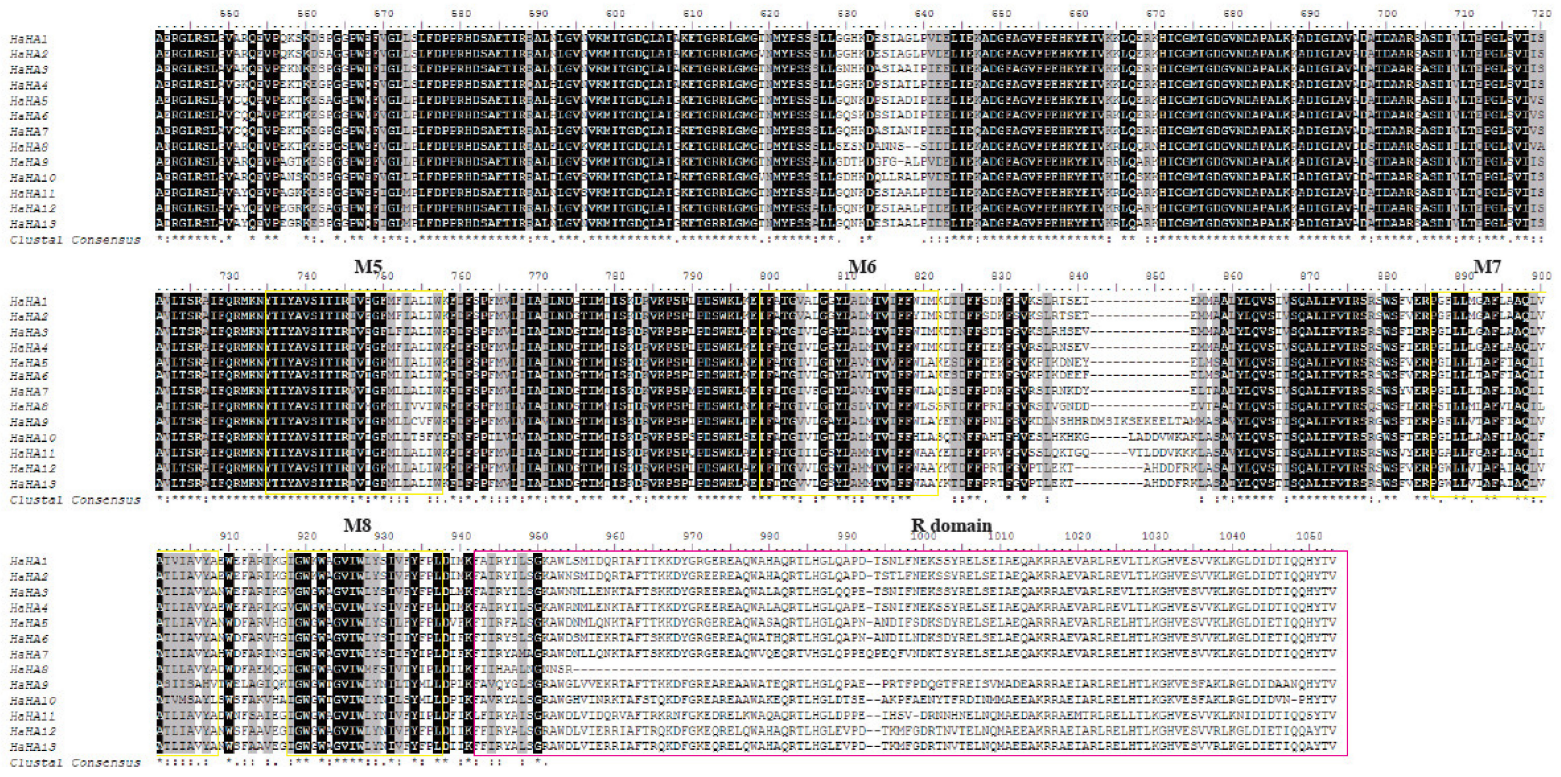

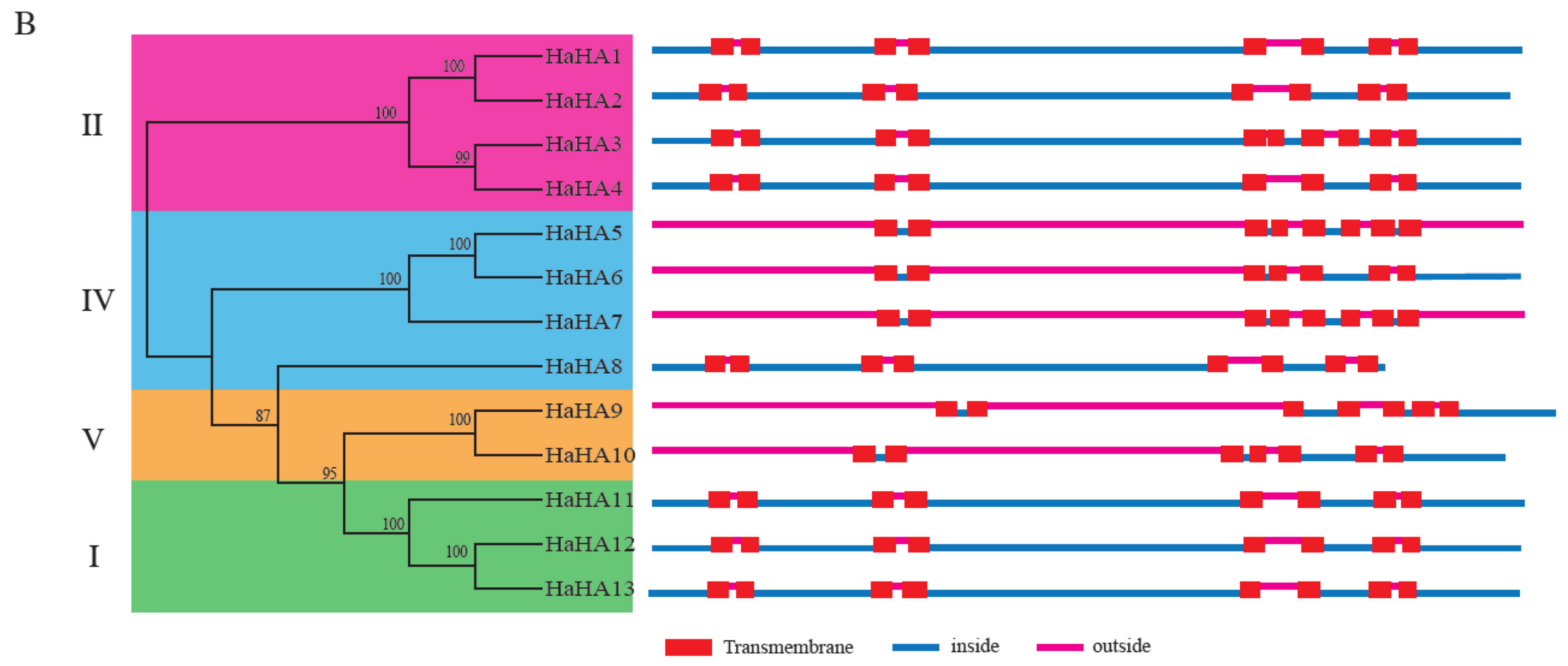
Multiple protein sequences alignment of HHA1–HHA13 (**A**) and the prediction of putative transmembrane domains (**B**). The multiple sequences alignment was analyzed with BioEdit software. High consensus amino acids are indicated with black, while low consensus residues are in gray. The pink boxes marked the typical conserved amino acid sequence of A-domain (TGE), P-domain (DKTGTLT), N-domain (KGAP), and the R-domain. The yellow boxes indicated the putative transmembrane domains (M1–M8). In panel B, the red rectangle represents the transmembrane region of the protein. The blue line represents the inner membrane area of the protein. The pink lines represent the outer membrane area of the protein.

To better understand the diversity and similarity of the PM H^+^-ATPase protein, the amino acid sequence alignment analysis was performed to analyze the transmembrane and typical domains of PM H^+^-ATPase proteins. Eight conserved domains (M1–M8) hypothesized to be essential for the transmembrane were identified in most of sunflower PM H^+^-ATPase members (Figure 3A). However, the transmembrane domain number of sunflower P-type ATPase was different among sub-clusters. Members of sub-cluster I had 8 transmembrane domains and sub-cluster V showed 7 transmembrane domains. In sub-cluster II, HHA1, HHA2, and HHA4 had 8 transmembrane domains, while HHA3 exhibited 10 transmembrane domains. In sub-cluster IV, HHA5, HHA7, and HHA8 had 8 transmembrane domains, while HHA6 exhibited 7 transmembrane domains, which is the same as sub-cluster V. Interestingly, proteins N terminal of sub-cluster IV (except for HHA8) and group V were outside the plasma membrane, and transmembrane domains were also a little far away from the N terminals. Conversely, proteins N terminal of groups/ sub-cluster I, II, and a member of sub-cluster IV (HHA8) were inside the plasma membrane with relatively close transmembrane domains (Figure 3B). Moreover, HHA8 was truncated by 99 amino acids at its C-terminus, and the 14-3-3 protein binding site was absent (Figure 3B). In addition, the typical amino acid of A-domain, N-domain, and P-domain of all the 13 HHA proteins were conserved with TGE, KGAP, and DKTGTLT, respectively (Figure 3A). However, the C-terminal regulatory domain (R-domain) showed most divergence (Figure 3A).

### 3.4. Promoter Cis-Acting Elements Analysis

**Figure 4:**
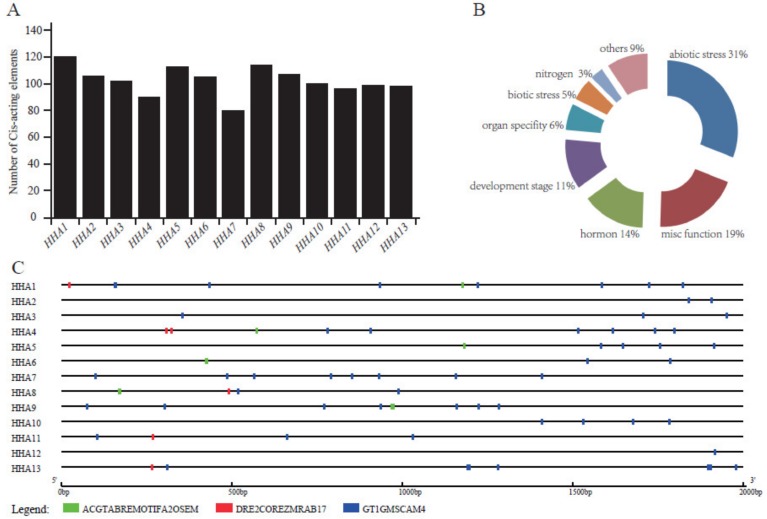
Frequency of *Cis*-acting elements in the 2 kb length promoter of *HHAs*. Statistical result of the number of non-repetitive Cis-acting elements of each *HHA* gene (A), the environmental-responsive *Cis*-acting elements (B), and the distribution of three salt stress-related *Cis*-acting elements in each of *HHA* gene promoter region (C).

The *Cis*-acting elements of gene promoters usually respond to the types of binding transcription factors, which are beneficial to the study of gene function and regulation. To further elucidate the potential regulatory mechanism of the *HHA* genes expression under environmental factors, the *Cis*-acting elements in gene promoter, which may be linked to these factors, were identified in the PLACE database. A total of 201 different non-repetitive *Cis*-acting elements were identified from thirteen PM H^+^-ATPase gene promoters (Supplementary File S7). Among the *Cis*-acting elements identified from the *HHAs* promoters, the *HHA1* promoter contained 120 *Cis*-acting elements, which is the gene with the most *Cis*-acting elements among the 13 genes. Subsequently, *HHA5* and *HHA8* both contained 113 *Cis*-acting elements. *HHA7* showed the least *Cis*-acting elements with 79 (Figure 4A). Among these various *Cis*-acting elements, the top 10 with the highest frequency in the PM H^+^-ATPase sub-gene family were CACTFTPPCA1, DOFCOREZM, CAATBOX1, ARR1AT, EBOXBNNAPA, MYCCONSENSUSAT, GT1CONSENSUS, ROOTMOTIFTAPOX1, GATABOX, and GTGANTG10. The number of the 10 *Cis*-acting elements in these genes is visualized in Supplementary File S8. Based on the statistical result, the distribution of *Cis*-acting elements in each gene was similar. Furthermore, the 201 non-repetitive *Cis*-acting elements were mainly functional, associated with abiotic stress (31%), miscellaneous function (19%), hormones (14%), development stage (11%), organ specificity (6%), and biotic stress (5%) (Figure 4B, Supplementary File S7), revealing the possible regulatory factors in the upstream of the *HHAs* promoter. Among the abiotic stress elements, light and drought response elements were dominant, while ABA and GA were the dominant response elements in the hormone elements (Supplementary File S7). In addition, three salt-stress elements GT1GMSCAM4 (all the 13 *HHAs*), DRE2COREZMRAB17 (*HHA1*, *HHA4*, *HHA8*, *HHA11*, and *HHA13*), and ACGTABREMOTIFA2OSEM (*HHA1*, *HHA4*, *HHA5*, *HHA6*, *HHA8,* and *HHA9*) were identified as unevenly distributed on the promoters of *HHAs* (Figure 4C), suggesting that *HHA*s may be regulated by salt stress.

### 3.5. Spatiotemporal Expression Patterns of HHAs in Sunflower Tissues

**Figure 5:**
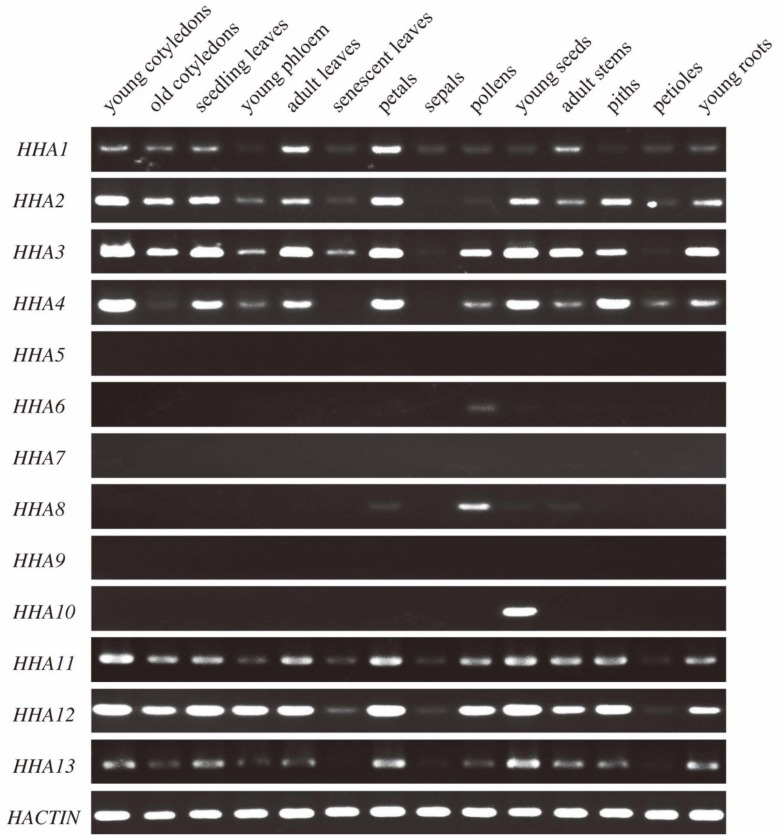
Tissue expression patterns of sunflower *HHAs* determined by semi-quantitative PCR. All the tissues were collected from the 2-week-old seedlings and 8-week-old adult plants. The *HACTIN* gene was amplified to normalize the expression level of *HHA* genes. Semi-quantitative PCR was performed at 28 cycles, and the amplified products were electrophoresed on 2% agarose gel.

The spatiotemporal expression patterns of PM H^+^-ATPase genes were detected by semi-quantitative PCR in 14 different tissues. The members of the HHA gene family belonging to sub-cluster I and II were widely expressed in almost all of the tissues studied (Figure 5). Specifically, sub-cluster I members *HHA11*, *HHA12*, and *HHA13* showed similar expression patterns in these tissues. In addition, *HHA12* exhibited the highest expression level in all but senescent leaves, sepals, and petioles. The expression patterns of members of sub-cluster II (*HHA1*, *HHA2*, *HHA3,* and *HHA4)* were also similar. The expression levels of *HHA2*, *HHA3,* and *HHA4* were higher than that of *HHA1*. However, the expression range of members in sub-cluster II was not as wide as that of sub-cluster I. Tissues such as young cotyledons, seedlings leaves, adult leaves, petals, adult stems, and young roots showed high transcripts levels of *HHA1*, *HHA2*, *HHA3,* and *HHA4*, while the expression levels of these genes were very low in other tissues (Figure 5). Surprisingly, members of sub-cluster IV and V showed almost no expression in these tissues, except a relatively high expression of *HHA8* and *HHA10* in pollens and young seeds, respectively, and a low expression of *HHA6* in pollens and *HHA8* in petals, young seeds, and adult stems (Figure 5). The absence of amplified bands of these genes was not caused by inactive primers, which produce single and strong band bands in young leave genomic DNA (Supplementary File S9). The results showed that the genes belonging to sub-clusters IV and V were probably pseudogenes and not expressed.

### 3.6. Expression Patterns of HHAs in Response to Salt Stress

**Figure 6:**
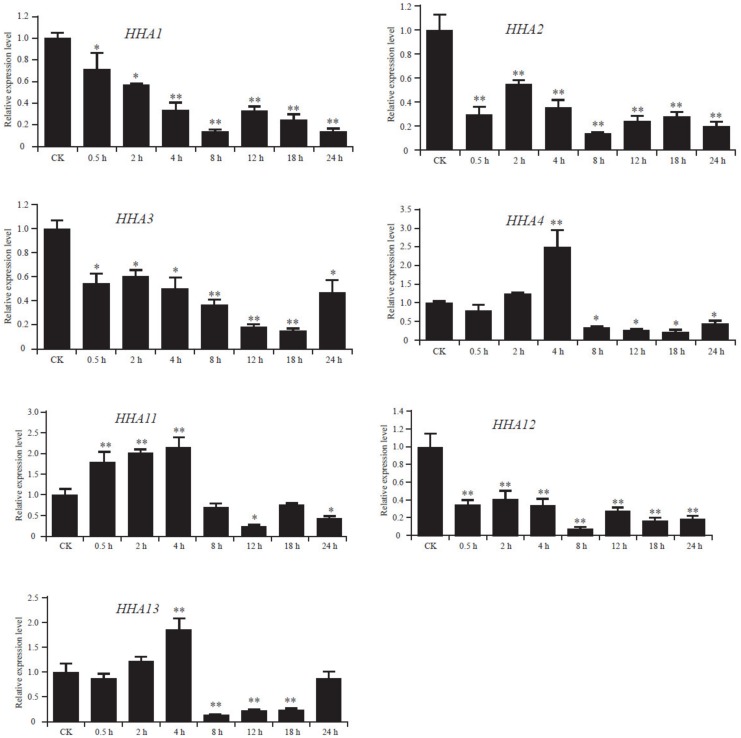
The relative expression levels of *HHA* genes under 150 mM NaCl stress. *X*-axis represents sampling time. CK is the plant that was not watered with NaCl solution. The error bar represents the standard deviation (SD) based on three biological replicates. The statistical significance was determined by the ANOVA method using SPSS software (* *p* < 0.05, ** *p* < 0.01).

To study the potential function and mechanism of *HHAs* in response to salt stress, the expression levels of the genes belonging to sub-cluster I and II were determined in leaves by qRT–PCR. According to Figure 6, the expression of *HHA1*, *HHA2*, *HHA3,* and *HHA12* was significantly reduced by salt stress. The expression levels of *HHA11* presented a trend of upregulation at first, but it was later downregulated after 4 h of exposure to 150 mM NaCl. The highest expression level of *HHA11* occurred at 4 h after exposure, and then the expression was severely inhibited by salt stress with an increase in exposure time. *HHA4* and *HHA13* showed a similar trend. At the 0.5-h time point, the expression levels of *HHA4* and *HHA13* were both downregulated in comparison with control under salt stress. At the 2-h time point, they were upregulated, and both reached a peak expression level at the 4-h time point. Subsequently, the expression of these two genes decreased dramatically and kept at a low level (Figure 6).

### 3.7. Subcellular Localization Analysis

**Figure 7:**
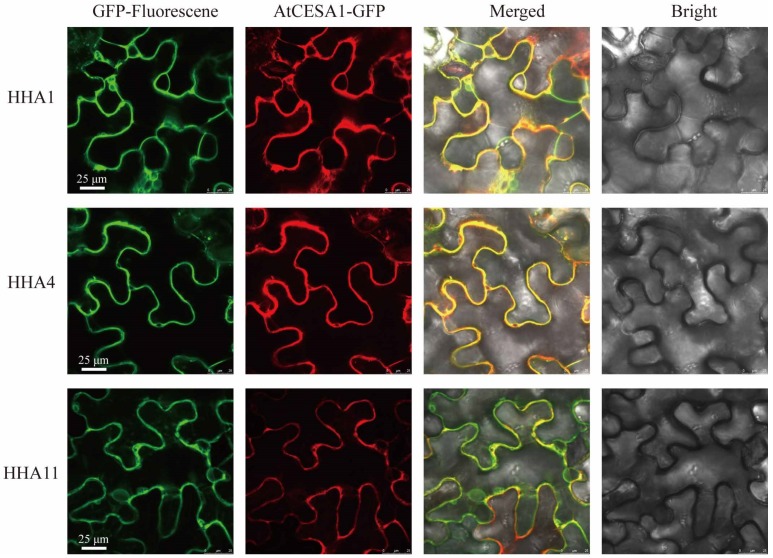
Sunflower HHA1, HHA4, and HHA11 fusion protein subcellular localization. AtCESA1-RFP is a plasma membrane-anchored marker protein.

The subcellular localizations of proteins are often closely associated with their functions. To verify the accuracy of HHA protein prediction in subcellular localization, *HHA1*, *HHA4,* and *HHA11,* which exhibited up- or downregulated expression patterns under salt stress (Figure 6) and grouped into different sub-clusters (Figure 1), were chosen for the construction of HHAs–GFP fusion proteins. As shown in Figure 7, the GFP signals of HHA1, HHA4, and HHA11 fusion proteins were all merged with the plasma membrane anchored marker protein 35S::AtCESA1-RFP after transient expression in tobacco leaves, suggesting that HHA1, HHA4, and HHA11 were indeed localized to the plasma membrane in vivo.

### 3.8. Overexpression of HHA4 and HHA11 Results in Resistance to Salinity Stress

**Figure 8:**
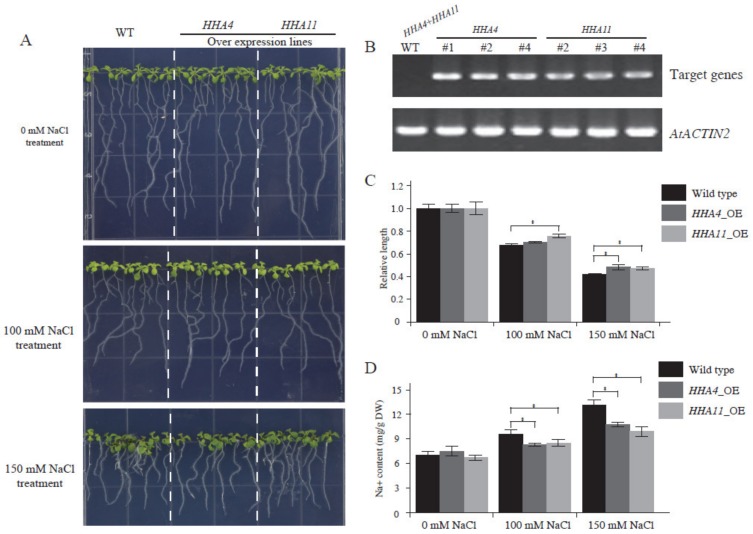
The phenotypes of transgenic *Arabidopsis* with *HHA4* and *HHA11* under NaCl stresses. The phenotypes of 2-week-old transgenic *Arabidopsis* with *HHA4* and *HHA11* treated with 100 mM and 150 mM NaCl (**A**); the expression level identification of *HHA4* and *HHA11* in *Arabidopsis* transgenic plants by semi-quantitative PCR (**B**); the main root length of transgenic plants under salt stress (**C**). Ten seedlings of transgenic lines and control plants were used to record the main root length, and the root length data were analyzed with the ANOVA method using the SPSS software (* *p* < 0.05); Na^+^ content of transgenic plants under salt stress (**D**). The statistical result was analyzed with the ANOVA method using SPSS software (* *p* < 0.05).

As shown in Figure 6, the expression of *HHA4*, *HHA11,* and *HHA13* were significantly increased during salinity stress, suggesting that these genes might play a key role during salt stress. To test whether sunflower PM H^+^-ATPase genes play an important role during salt stress, *Arabidopsis* transgenic plants over-expressing *HHA4* and *HHA11* (*HHA4*-OE, and *HHA11*-OE) were developed. The homologous T_2_ transgenic generation lines were selected for detailed characterization. Semi-quantitive PCR analysis showed that the expression level of *HHA4* and *HHA11* were both higher in transgenic lines than in wild-type plants (Figure 8B). After 10 days of culture under NaCl stress conditions, *HHA4* and *HHA11* overexpressing *Arabidopsis* lines showed better growth performance on 100 and 150 mM NaCl-containing plates when compared to wild-type seedlings plants (Figure 8A). Data from the statistical analysis show that the root length of *HHA4*-OE and *HHA11*-OE lines was significantly longer than those from control plants under 100 and 150 mM NaCl stress (Figure 8C). To further explore the function of PM H^+^-ATPase genes in salt tolerance, the Na^+^ content of transgenic lines and control samples were measured. The results showed that the *HHA4* and *HHA11* over-expression lines had less Na^+^ accumulation compared to control plants under 100 and 150 mM NaCl (Figure 8D), suggesting that PM H^+^-ATPase could participate in the efflux of Na^+^.

## 4. Discussion

The P-type PM H^+^-ATPases are actually ion pumps. They can hydrolyze adenosine triphosphate (ATP) to generate the energy required for the efflux of hydrogen ions (H^+^) from the cytoplasm at an inverse concentration to generate the H^+^ concentration gradient across the membrane [42]. Thus the H^+^ gradient provides the driving force for material transportation, including nutrient uptake, various ions transporting, and small molecule metabolites across the plasma membranes [7,8,42]. Hence, they are vital for many life activities of organisms [3,16,43]. Until now, the members of PM H^+^-ATPases were identified in many species, such as in *Arabidopsis*, *Oryza* *sativa, Lycopersicon esculentum*, *Zea mays*, *Cucumis sativus*, *Solanum tuberosum* L, and *Nicotiana plumbaginifolia* [5,15,16,26,27,28]. The recent release of a complete sunflower genome sequences and annotations provides convenience for PM H^+^-ATPase sub-gene family member identification and functional studies [29]. In this study, a total of 13 members of the sunflower PM H^+^-ATPase sub-gene family were identified, which were renamed *HHA1* through *HHA13* according to the homology of *A. thaliana*. Subcellular prediction (Table 1) and in vivo experiment (Figure 7) verified that PM H^+^-ATPases were located on the plasma membrane, which is consistent with the previous study [44].

P-type PM H^+^-ATPases are reported to participate in many physiological functions and to play key roles during plant growth and development [3,16,43]. To address the functional diversity and physiological role of sunflower PM H^+^-ATPase genes, the spatiotemporal expression patterns in different tissues were studied. The present semi-quantitative PCR results revealed that PM H^+^-ATPase genes that belonged sub-cluster I and II had a boarder expression pattern in different tissues. However, the sub-cluster IV and V members were almost not expressed (Figure 5). Interestingly, several previous studies on PM H^+^-ATPase genes have also shown similar expression patterns in tomato [45], cucumber [28], *Nicotiana plumbaginifolia* [46], Zea mays [47], and *Arabidopsis* [48]. Extensive expression patterns in many species confirmed that sub-cluster I and II genes are necessary for optimal plant growth. Detailed analysis revealed that the expression patterns of members that showed close evolutionary relationships were still different. For example, the duplicated genes of *HHA12* and *HHA13* that were grouped in sub-cluster I showed a near evolution on the phylogenetic tree (Figure 1). The gene structure and protein-conserved motifs were also similar (Figure 2 and Figure 3). The expression level of *HHA12* in different tissues was significantly higher than that of *HHA13* (Figure 5). However, *HHA13* and *HHA11* had similar expression patterns (Figure 5). For the duplication gene pairs *HHA1–HHA2* and *HHA3–HHA4*, the expression intensity and range of *HHA2* and *HHA3* were significantly higher than that of *HHA* and *HHA4* (Figure 5). These results imply that the expression of closely related genes that may be caused by duplication might be regulated in significantly different ways.

Soil salinity is a major abiotic stress in agricultural crop productivity worldwide, which has a significant negative effect on plant growth and development [49]. Osmotic stress and ion toxicity are the two main stresses resulting from the excessive uptake of less demanded elements, mainly Na^+^ under salt stress [13]. Hence, there are three different types for plant adaptations to salt stress: Na^+^ or Cl^−^ exclusion/excretion, osmotic stress tolerance, and accumulation of Na^+^ or Cl^−^ in special tissues [50]. Both salt exclusion and excretion can reduce salt accumulation in tissues. Actually, most salt-tolerant plants maintain relatively low sodium concentration in the cytoplasm [51]. However, salt exclusion and excretion are two different mechanisms that are not easy to distinguish. Generally speaking, salt exclusion is a mechanism that prevents salt from entering the cells while allowing the water to pass through, while salt excretion is a mechanism that removes sodium ions from cells and depends on the proton gradient of the electrochemical membrane catalyzed by a specific Na^+^/H^+^ antiporter. Interestingly, PM H^+^-ATPase is the only pump that generates an electrochemical proton gradient across the plasma membranes [13]. Hence, PM H^+^-ATPase is believed to play a major role in salt stress tolerance [13]. In this study, the expression level of *HHA4*, *HHA11,* and *HHA13* were increased under salt stress (Figure 6), which was consistent with many previous studies that suggested that salt stress could induce the expression of PM H^+^-ATPases in plants [11,12,26,52]. The stress-induced expression level suggests that PM H^+^-ATPases might potentially place an important role in the development of salt tolerance. Transgenic *Arabidopsis* plants overexpressing a PM H^+^-ATPase gene *PeHA1* significantly enhanced their salt tolerance capacity by maintaining the ions homeostasis and of reactive oxygen species [19]. In this study, compared to the control plants, transgenic *Arabidopsis* plants overexpressing *HHA4* and *HHA11* showed a decrease of Na^+^ content under salt stress conditions (Figure 8C), which implies the PM H^+^-ATPase participates in the physiological process of Na^+^ efflux, resulting in the resistance of these plants to salt stress.

## 5. Conclusions

This study firstly systemically analyzed the P-type PM H^+^-ATPases sub-gene family in sunflower, especially exploring the function of *HHA4* and *HHA11* in salt tolerance. A total of 13 PM H^+^-ATPase genes were identified in the sunflower genome and renamed *HHA1* to *HHA13*. *HHA* genes showed distinct spatiotemporal expression patterns in different sunflower tissues and sub-clusters. In addition, only three genes (*HHA4*, *HHA11,* and *HHA13*) were induced under salt stress. Under 100 and 150 mM NaCl treatment, the main root length of transgenic *Arabidopsis* plants overexpressing *HHA4* and *HHA11* was longer than that of wild-type plants indicating the positive function of *HHA* genes in the development of salt stress tolerance. The decrease of Na^+^ content of the transgenic plants indicated that PM H^+^-ATPase participated in the physiological process of Na^+^ efflux. The plasma membrane localization of PM H^+^-ATPase is consistent with its function. These results may provide the biological foundation for further function or salt-tolerant mechanism studies of PM H^+^-ATPase.

## Figures and Tables

**Table 1:** Characteristics of plasma membrane (PM) H^+^-ATPase sub-gene family in sunflower.

Groups	Gene Name	Accession NO.	Chr	Genomic Location	Orthologous in *Arabidopsis*	Identity with *Arabidopsis* (%)	Protein length	Molecular Weight MW (Da)	PI	Subcellular Location	Intron
II	*HHA1*	HanXRQChr12g0358351	C12	13551328 - 13560479	AT2G18960	85.84	954	104986.84	7	Plasma membrane	13
II	*HHA2*	HanXRQChr10g0308521	C10	205602015 - 205610779	AT5G57350	85.67	942	103947.71	7	Plasma membrane	12
II	*HHA3*	HanXRQChr05g0142281	C05	103008186 - 103001095	AT5G57350	85.77	954	105039.85	6	Plasma membrane	14
II	*HHA4*	HanXRQChr06g0179401	C06	55584733 - 55575412	AT5G57350	85.35	954	105292.35	7	Plasma membrane	15
IV	*HHA5*	HanXRQChr02g0034901	C02	19396893 - 19388276	AT1G80660	83.95	953	105009.63	6	Plasma membrane	13
IV	*HHA6*	HanXRQChr04g0098641	C04	17528781 - 17534812	AT1G80660	83.53	953	104886.54	6	Plasma membrane	12
IV	*HHA7*	HanXRQChr16g0500091	C16	8389529 - 8397496	AT1G80660	83.87	955	105464.42	6	Plasma membrane	13
IV	*HHA8*	HanXRQChr10g0314521	C10	228958293 - 228963082	AT3G42640	78.80	851	93419.13	5	Plasma membrane	6
V	*HHA9*	HanXRQChr13g0423171	C13	186222209 - 186239644	AT5G62670	79.48	1050	115861.86	8	Plasma membrane	19
V	*HHA10*	HanXRQChr16g0509551	C16	64253539 - 64258063	AT3G47950	79.32	937	102808.16	7	Plasma membrane	14
I	*HHA11*	HanXRQChr17g0547551	C17	51582188 - 51588359	AT3G47950	87.12	959	105493.54	6	Plasma membrane	20
I	*HHA12*	HanXRQChr05g0157971	C05	199927699 - 199933355	AT3G47950	92.36	957	105363.3	6	Plasma membrane	20
I	*HHA13*	HanXRQChr08g0235741	C08	141687703 - 141693862	AT3G47950	91.83	957	105333.27	6	Plasma membrane	20

## Supplementary Materials

The following are available online at https://www.mdpi.com/2073-4425/11/4/361/s1. Supplementary File S1: Primers used in this study. Supplementary File S2: Coding sequences of sunflower PM H^+^-ATPase genes. Supplementary File S3: Genomic sequences of sunflower PM H^+^-ATPase genes. Supplementary File S4: Protein sequences of sunflower PM H^+^-ATPase genes. Supplementary File S5: Protein sequences of PM H^+^-ATPases derived from other species. Supplementary File S6: Conserved motifs analysis in the full-length HHA proteins of sunflower with the MEME tool. Supplementary File S7: *Cis*-acting elements abundance for HHA family genes. Supplementary File S8: The distribution of the top ten most enriched *Cis*-acting elements in HHA gene family members. Supplementary File S9: Identification of the availability of *HHAs* gene-specific primers by common PCR with DNA template.
