# Supplementary material for: Genome-Wide Identification and Analysis of P-Type Plasma Membrane H+-ATPase Sub-Gene Family in Sunflower and the Role of HHA4 and HHA11 in the Development of Salt Stress Resistance
Source: Genes (Basel). 2020 Mar 27;11(4):361. doi: 10.3390/genes11040361 (PMC7231311; doi:10.3390/genes11040361)
Supplement: Supplementary file 1 [file genes-11-00361-s001.zip › Supplementary File 2.docx]

Supplementary File 2: Coding sequences of sunflower PM H^+^-ATPase genes.

# >HHA1

ATGGGCGGCGACAAGGCTCTCAGCCTCGAAGGGATTAAAAACGAGACCGTCGATCTGGAAAAAGTACCGATCGAAGAGGTGTTTGAACAGTTGAAATGTAACCGTGAAGGTCTTAGCTCCGACGAAGGAGCCCAACGTCTTGAAATTTTCGGACCCAACAAATTGGAGGAGAAAAAGGAAAGCAAAATCCTCAAGTTTCTTGGGTTTATGTGGAACCCCCTATCATGGGTCATGGAGGCTGCAGCCATCATGGCGATTGCACTCGCTAACGGCGGTGGTAAGCCGCCAGATTGGCAAGATTTCGTTGGTATCGTTTGCCTGCTTGTTATCAACTCCACCATCAGTTTCATCGAAGAAAACAACGCTGGAAACGCTGCCGCTGCACTTATGGCCGGTCTTGCACCTAAAACCAAGCTTTTGAGGGATGGCCGATGGAGCGAACAAGAAGCTGCTATACTGGTTCCCGGAGATATCATCAGTATCAAACTTGGTGATATCGTTCCTGCTGATGCGCGTCTTCTTGAAGGTGATCCCTTAAAGATCGACCAATCTGCGCTTACTGGAGAATCTCTCCCGGTCACCAAGAACCCATACGATGAAGTGTTCTCCGGTTCAACTTGCAAGCAAGGTGAACTTGAAGCCGTTGTCATCGCTACTGGCGTCCACACCTTTTTCGGAAAGGCTGCACATCTTGTGGACAGCACAAACCAAGTGGGACACTTCCAAAAGGTTCTAACCGCAATCGGAAACTTCTGTATCTGCTCCATTGCTGTCGGAATGGCTGTTGAGATTATCGTTATGTACCCGATCCAACATAGAGAGTACCGATCCGGGATTGACAATCTTCTGGTGTTGCTTATTGGTGGAATCCCGATCGCTATGCCTACTGTTCTTTCGGTCACTATGGCTATCGGCTCACACAGGCTTTCGCAGCAGGGTGCGATCACTAAACGGATGACTGCTATTGAAGAAATGGCGGGAATGGATGTTCTTTGCAGTGATAAAACCGGAACTTTGACGCTAAACAAGCTTACGGTTGATAAGAATTTGATCGAGGTGTTTGGTAAAGGCTTGGATAAGGAGCAAGTGTTGCTTTACGCTGCTCGCGCTTCTCGAATGGAAAACCAAGACGCTATCGATGCAGCCATCGTCGGAACGCTTGCTGACCCCAAAGAGGCACGAGCTGGCATTAGAGAGGTCCATTTCTTCCCGTTCAACCCTGTCGACAAGAGGACTGCGTTAACGTACATTGATAATAACGGCAACTGGTTTAGAGCTAGCAAGGGTGCGCCTGAACAGATATTGACACTTTGCGGGTGCAGGGAAGATCTCAAGAAGAAAGTTCACGCGATGATTGATAAATTCGCCGAACGTGGGTTGAGATCTTTGGGTGTTGCAAGACAGGAAGTGCCTCAAAAATCGAAAGATAGCCCGGGTGGTCCATGGGAATTCGTTGGATTGTTGTCTCTCTTTGATCCACCAAGGCATGACAGTGCCGAGACCATTAGAAGAGCTCTCAATCTCGGTGTAAACGTCAAGATGATTACTGGTGACCAACTTGCTATTGCTAAGGAAACTGGAAGACGTCTTGGTATGGGAACAAACATGTACCCTTCGTCGTCTTTACTTGGCGGACACAAGGACGAATCAATTGCTGGACTTCCAGTAGACGAGTTGATCGAGAAAGCTGATGGATTCGCCGGAGTTTTCCCTGAGCACAAGTATGAAATTGTGAAGAAACTACAAGAGAGGAAGCACATTTGTGGTATGACCGGAGATGGTGTGAACGACGCGCCTGCGTTAAAGAAGGCGGATATTGGTATTGCGGTTGCTGATGCTACCGACGCTGCAAGAAGTGCTTCCGACATTGTGCTTACCGAGCCTGGGCTCAGTGTGATCATCAGTGCTGTGCTTACCAGTAGAGCTATTTTCCAAAGAATGAAGAATTACACCATCTACGCCGTTTCAATCACCATTCGTATAGTGTTTGGATTTATGTTCATCGCGTTGATATGGAAGTTTGACTTCTCGCCTTTCATGGTTCTTATCATCGCAATCCTCAACGACGGTACTATCATGACAATCTCGAAGGATAGAGTGAAACCATCTCCGTTACCCGACAGCTGGAAGCTAAAAGAGATCTTCGCCACTGGAGTTGCTCTCGGAGGTTACTTAGCTTTAATGACAGTTATCTTCTTCTGGATCATGAAGGATACCGACTTTTTCTCAGATAAATTTGGTGTGAAATCTTTAAGAACCAGTGAAACCGAGATGATGGCCGCTTTATATCTACAAGTCAGTATCGTAAGCCAAGCTCTCATCTTCGTCACCCGTTCTCGTAGCTGGTCGTTCGTCGAACGACCTGGCTTCTTACTGATGGGCGCTTTCCTAGCAGCACAACTGGTAGCAACTGTAATTGCGGTATACGCAGAGTGGGAATTCGCGAGAATTAAAGGAATCGGATGGAAATGGGCTGGTGTTATCTGGCTTTACAGTATAGTGTTTTACTTCCCTCTTGATATTATGAAGTTTGCCATTAGATACATCCTTAGTGGCAAGGCTTGGCTCAGCATGATTGACCAAAGGACTGCTTTCACAACAAAGAAGGATTATGGTAGAGGAGAGAGAGAGGCCCAATGGGCTCATGCTCAAAGAACTTTACATGGGCTTCAAGCACCTGACACATCAAATCTCTTTAATGAGAAGAGCAGCTATAGAGAACTGTCGGAAATCGCTGAACAAGCCAAACGACGCGCTGAAGTTGCAAGGCTTCGGGAGGTGCTTACGCTCAAGGGTCACGTTGAGTCAGTGGTGAAACTGAAGGGGCTCGACATTGATACAATTCAACAGCATTATACAGTATGA

# >HHA2

ATGAGACCGTCGATCTGTTCCGAAAAAGTACCAATTGAAGAGGTGTTCGAGCAGTTGAAATGTAACCGAGAAGGTCTGAGCTCCGATGAAGGAGCCCAAAGGCTCGAAATTTTCGGACCCAACAAACTAGAAGAGAAGAAGGAAAGCAAATTCCTCAAGTTTCTCGGGTTTATGTGGAACCCTCTATCATGGGTCATGGAGGCTGCCGCCATAATGGCCATTGCACTCGCTAACGGTGGTGGTAAGCCGCCAGATTGGCAAGATTTCGTCGGTATTGTTTGCCTGCTTGTTATCAACTCCACCATCAGTTTCATCGAAGAAAACAACGCTGGAAACGCTGCTGCTGCACTTATGGCCGGTCTCGCACCTAAAACCAAGGTTCTAAGGGACGGACGCTGGAGCGAACAAGAAGCTTCTATACTGGTTCCAGGAGATATCATAAGCATCAAACTCGGTGATATTGTTCCGGCTGATGCTCGTCTACTAGAAGGCGATCCATTAAAAATCGATCAATCCGCCCTTACCGGAGAATCTCTCCCGGTAACCAAAAACCCATACGACGAAGTATTCTCCGGTTCAACTTGCAAACAAGGCGAACTGGAAGCCGTTGTGATCGCCACAGGAGTCCACACCTTTTTCGGCAAGGCTGCGCATCTAGTCGACAGCACAAACCAAGTGGGACACTTCCAAAAGGTTCTCACCGCTATCGGAAACTTCTGCATTTGCTCCATTGCTGTAGGAATGGTGGTAGAGATCATTGTCATGTACCCGATCCAACACCGAGAGTACCGAAAAGGGATTGATAATCTGTTAGTGTTGCTTATTGGTGGTATTCCGATTGCTATGCCTACTGTTCTTTCGGTTACTATGGCTATTGGATCGCATAGGCTTTCACAACAAGGTGCTATTACTAAAAGAATGACTGCTATTGAAGAAATGGCGGGAATGGATGTGCTTTGTAGTGATAAAACTGGGACTTTGACGCTTAATAAGCTTACGGTTGATAAGAATTTGATTGAGGTGTTTGGTAAAGGTTTGGATAAGGAACAGGTTTTGCTGTATGCTGCTAGAGCTTCTAGGATGGAAAACCAAGATGCTATTGATGCTGCTATTGTTGGAACCCTTGCTGATCCCAAAGAGGCACGAGCTGGCATTAGAGAGGTCCATTTCTTCCCATTCAACCCGGTGGATAAGAGGACTGCTTTGACATACATTGATAATAATGGCAACTGGTTTAGAGCTAGCAAAGGTGCACCTGAACAGATTTTGACCCTTTGCGGGTGCAAAGAAGACCTGAAGAAGAAAGTTCACGCAATGATTGATAAATTCGCTGAACGTGGGCTGCGATCTTTGGGTGTTGCGAGACAGGAAGTGCCGCAAAAATCGAAAGATAGCGCAGGCGGTCCGTGGGAGTTTGTTGGGTTGTTGTCTCTCTTTGATCCACCAAGGCATGACAGTGCCGAGACCATCCGAAGAGCTCTCAACCTTGGTGTTAATGTCAAGATGATTACTGGTGATCAACTTGCTATCGCTAAGGAAACCGGTAGAAGGCTTGGTATGGGAGTTAACATGTATCCTTCGTCGTCTTTACTTGGCGGACACAAGGACGAATCAATTGCCGGACTTCCAGTAGACGAGTTGATCGAGAAAGCTGATGGATTTGCTGGAGTTTTCCCTGAGCACAAGTATGAAATTGTGAAGAAATTACAAGAGAGGAAGCACATTTGTGGTATGACTGGAGATGGTGTGAACGACGCACCTGCTTTAAAGAAGGCTGATATTGGAATTGCGGTTGCTGATGCAACCGACGCTGCAAGAAGTGCTTCTGATATCGTGCTTACTGAGCCTGGGCTTAGTGTCATCATCAGTGCTGTGCTTACGAGTCGAGCTATTTTCCAAAGAATGAAGAATTACACCATCTATGCGGTTTCAATCACCATTCGTATCGTGTTTGGATTTATGTTTATCGCGTTGATATGGAAGTTTGACTTCTCTCCCTTTATGGTGCTTATTATTGCAATTCTTAACGACGGTACTATCATGACAATCTCCAAGGATAGGGTGAAACCATCTCCGTTACCAGACAGCTGGAAGCTAAAAGAGATCTTTGCCACCGGAGTTGCTCTTGGAGGTTACTTAGCTTTAATGACAGTTATCTTCTTCTATATCATGAGGGATACCGACTTTTTCTCGGATAAATTTAGCGTGAAATCTCTTCGGACCAGTGAAACCGAGATGATGGCTGCTTTATATTTGCAAGTCAGTATTGTGAGTCAAGCTCTCATCTTCGTCACGCGTTCACGTAGCTGGTCGTTCGTTGAACGACCCGGCTTCTTACTGATGGGCGCTTTCCTTGCAGCACAACTGGTAGCAACTTTAATCGCGGTATACGCAGAGTGGGAATTCGCAAGAATCAAAGGAATCGGATGGAAATGGGCTGGCGTTATCTGGCTTTACAGCATCGTGTTTTATTTCCCGCTTGATATCATGAAGTTTGCTATCAGATACATCCTTAGTGGCAAGGCTTGGAACAGCATGATTGACCAAAGGACTGCTTTCACAACAAAGAAGGATTATGGAAGAGAAGAAAGAGAGGCCCAATGGGCTCATGCTCAAAGGACTCTTCACGGGCTTCAAGCACCCGATACCTCAACCCTCTTCAACGAAAAGAGCAGCTACAGAGAATTGTCTGAAATCGCTGAACAGGCCAAACGACGTGCTGAAGTTGCAAGGCTTCGAGAGGTGCTTACGCTCAAGGGTCATGTTGAGTCAGTGGTGAAACTGAAGGGGCTCGACATTGATACTATTCAACAGCATTATACAGTATGA

# >HHA3

ATGGGCGGCGATAAGGCTCTCAGTCTCGAAGAGATTAAAAATGAAACTGTTGATCTGGAGAAAGTACCCATTGAAGAGGTGTTTGAACAGTTGAAATGTAACCGAGAAGGTCTAAGTTCCGATGAAGGGGCTGCAAGACTTCAAATTTTTGGTCCTAACAAATTGGAGGAGAAAAAGGAAAGCAAACTACTCAAGTTTCTTGGGTTCATGTGGAATCCTCTATCATGGGTCATGGAAGCTGCAGCCATTATGGCAATTGCACTGGCTAATGGAGGCGGGAAACCACCCGACTGGCAAGACTTTGTCGGTATTGTTTGCCTTCTTGTTATCAACTCAACCATCAGTTTTATCGAAGAAAACAATGCTGGAAATGCTGCTGCTGCACTTATGGCTGGTCTCGCCCCTAAAACCAAGGTTCTGAGGGATGGTAATTGGAGTGAACAAGAAGCTGCAATCTTGGTTCCAGGAGATATTATCAGTATCAAACTTGGTGATATCGTTCCTGCTGACGCACGTCTCCTAGAAGGTGACCCCTTAAAGATTGACCAATCTGCCCTTACTGGTGAATCGCTTCCTGTGAACAAGAACCCTTACGACGAGGTGTTTTCCGGTTCAACTTGCAAGCAAGGTGAAATAGAAGCCGTCGTTATCGCAACTGGTGTGCACACTTTCTTCGGGAAAGCCGCACACTTAGTGGATAGCACTAACCAAGTCGGTCACTTCCAACAAGTTCTAACAGCTATCGGAAACTTCTGTATCTGCTCAATCGCTGTCGGAATGTTGGTTGAGATTGTCGTCATGTATCCGATTCAGCACAGGGAGTACAGAAATGGAATCGACAATCTTCTGGTCTTGCTTATCGGTGGAATCCCAATCGCTATGCCGACGGTTCTTTCTGTCACCATGGCTATTGGATCGCATAGGCTTTCACAACAAGGAGCTATTACTAAAAGAATGACTGCCATTGAAGAAATGGCGGGAATGGACGTACTTTGCAGTGATAAAACCGGAACACTGACTCTTAACAAGCTTAGTGTTGATAAAAATTTGATTGAGGTTTTTGCTAAAGGTGTCGATAAGGATCAGGTTCTGCTTTATGCTGCTCGGGCTTCTAGGACCGAAAACCAGGACGCTATTGATGCGGCCATTGTTGGAACCCTTGCTGACCCGAAAGAGGCACGAGCTGGAATTAGGGAGGTCCATTTCTTTCCGTTTAATCCTGTTGACAAGCGGACAGCTTTGACATACATCGATGAGCGCGGTAACTGGCATAGAACAAGCAAAGGTGCTCCCGAACAGATTTTGACCCTCTGTGGATGCAAAGAAGATATGAAAAAGAAAGTTCATGCAATGATTGATAAATTTGCTGAACGTGGGTTGCGTTCTTTGGCTGTTGCGAAACAGGAAGTACCTGAAAAAAACAAAGAAAGCCCTGGTGGTCCATGGACGTTTATCGGATTGTTGTCGTTGTTTGACCCACCAAGGCATGACAGTGCTGAGACCATTCGCAGAGCTCTCAATCTCGGTGTCAATGTTAAGATGATTACTGGTGATCAACTTGCTATTGCTAAAGAGACTGGTAGACGACTCGGTATGGGAACAAACATGTACCCGTCTTCTTCTTTACTCGGTAACCACAAGGATGCATCGATAGCCGCTATCCCTATTGAAGAGTTGATCGAGAAAGCCGATGGATTTGCCGGAGTTTTCCCAGAACACAAGTATGAGATTGTGAAGAAGTTACAAGAAAGAAAGCACATTTGTGGAATGACTGGTGATGGTGTGAATGATGCGCCTGCTTTGAAGAAGGCCGATATTGGTATCGCTGTTGCCGATGCTACAGATGCCGCAAGGGGTGCTTCTGACATCGTGCTTACTGAGCCCGGGCTTAGTGTCATTATCAGTGCAGTGCTTACCAGTCGCGCTATTTTCCAAAGAATGAAGAACTACACCATCTACGCTGTTTCTATCACCATTCGTATTGTGTTTGGATTTCTGTTCATTGCTTTGATATGGAAGTTCGACTTCTCTCCCTTCATGGTTTTGATCATCGCAATCCTTAACGACGGCACAATCATGACAATCTCAAAGGATAGGGTGAAACCATCGCCATTACCCGACAGCTGGAAGCTAAAAGAGATCTTCGCCACCGGCATTGTGCTCGGAGGTTACCTTGCATTGATGACTGTCATATTCTTCTGGATCATGAAAGACACAAACTTCTTCTCGGACACGTTTGGCGTTAAATCTTTGAGACACAGTGAAGTTGAGATGATGGCTGCTTTATATCTTCAAGTCAGTATAGTCAGCCAGGCTTTGATTTTTGTGACGCGTTCTCGCAGTTGGTCGTTTATCGAACGTCCCGGTCTCTTACTTTTGGGTGCCTTCTTAGCAGCACAACTGGTGGCAACGTTAATCGCAGTGTATGCAAATTGGGAATTTGCAAGAATCAAGGGAGTCGGCTGGGGATGGGCTGGTGTCATCTGGCTCTACAGCATCGTGTTCTACTTCCCGCTCGATCTTATGAAATTCGCCATCCGATATATCCTTAGCGGGAAAGCATGGAACAACTTGCTTGAAAACAAGACTGCTTTCACAAGCAAGAAGGATTATGGAAGAGAAGAGAGAGAGGCCCAATGGGCTCTTGCTCAAAGGACATTGCATGGGCTCCAACAACCCGAAACCTCCAACATCTTTAACGAAAAGAGCAGCTACAGGGAGCTGTCTGAGATAGCGGAACAGGCTAAGAGGCGCGCTGAAGTTGCAAGGCTTCGGGAGGTGCTTACACTGAAGGGGCATGTTGAGTCGGTGGTGAAGCTGAAGGGGCTAGATATCGACACGATCCAACAGCATTACACAGTGTGA

# >HHA4

ATGGGGACCGATAAGGCTCTCAGTCTCGAAGAGATTAAAAACGAAACTATTGATCTGGAGAAAGTACCGATTGAGGAGGTGTTTCAACAGTTGAAATGTAACCGAGAAGGTCTAACCTCAGATGAAGGAGCTGCGAGACTGCAAATTTTCGGACCCAACAAATTGGAGGAGAAAAAAGAAAGCAAAATACTCAAGTTTCTCGGGTTCATGTGGAATCCTCTATCGTGGGTCATGGAAGCTGCAGCCATTATGGCAATCGCAATGGCTAATGGAGGTGGAAAGCCACCAGATTGGCAAGACTTTGTTGGTATTGTTTGCCTTCTTGTTATTAACTCAACCATCAGTTTTATTGAAGAAAACAATGCTGGAAACGCGGCTGCTGCACTTATGGCTGGTCTCGCCCCTAAAACCAAGGTTCTTCGGGATGGTCGTTGGAGTGAACAAGAAGCTTCCATCTTGGTCCCGGGAGATATTATCAGTATCAAACTTGGTGATATTGTTCCTGCTGATGCTCGTCTTCTAGAAGGTGATCCGTTAAAGATTGACCAATCTGCCCTTACTGGTGAATCACTTCCTGTGAACAAGAATCCGTATGACGAGGTGTTCTCCGGATCAACTTGCAAGCAAGGTGAAATCGAAGCGGTTGTTATCGCCACTGGTGTGCACACTTTCTTCGGAAAGGCTGCACATCTTGTGGATAGCACTAACCAAGTTGGTCATTTCCAACAAGTTCTTACGGCGATCGGAAACTTCTGTATCTGCTCCATTGCTGTTGGAATGTTGGTTGAGATAATCGTCATGTACCCGATTCAGCACAGGCAGTACAGAAGTGGGATTGACAATCTTTTGGTGTTGCTTATCGGTGGAATTCCGATTGCTATGCCTACTGTGCTTTCGGTCACCATGGCTATTGGATCGCATAGGCTTTCACAGCAGGGTGCTATTACTAAAAGAATGACTGCAATTGAAGAAATGGCTGGAATGGATGTGCTTTGCAGTGATAAGACGGGAACACTGACACTTAACAAGCTTACAGTTGATAAAAATTTAATTGAGGTGTTTGCAAAAGGTGTCGATAAGGAGCAGGTGCTGCTTTATGCTGCTAGGGCTTCTAGGACTGAGAACCAGGATGCTATTGATGCTGCCATTGTTGGAACCCTTGCTGATCCTAAAGAGGCACGAGCTGGAATTAGAGAGGTTCACTTCTTTCCATTTAACCCTGTTGACAAGAGGACAGCTTTGACGTACATCGATGAGCGTGGAAATTGGCATAGAACAAGCAAAGGTGCTCCAGAACAGATTTTGACCCTCTGTGGATGCAAAGAAGATATGAAAAAGAAAGTTCACGCAATGATTGATAAATTTGCTGAACGTGGGTTGCGTTCTTTGGCTGTTGGAAAACAGGAAGTGCCTGAAAAAACAAAAGAAGGCCCAGGTGGACCATGGCAATTTGTTGGATTGTTGTCCCTATTCGACCCACCAAGGCATGACAGTGCTGAGACCATTCGCCAAGCTCTCCATCTTGGTGTAAATGTCAAAATGATTACCGGCGACCAACTTGCCATTGCTAAGGAGACTGGTAGACGGCTCGGTATGGGAACAAACATGTACCCTTCTTCTTCTTTGCTCGGTGGCCACAAGGATCCATCCATTGCAACACTTCCTATTGAAGAGTTGATAGAGAAAGCCGATGGATTTGCAGGAGTTTTTCCAGAACACAAGTATGAGATTGTGAAGAAGTTACAAGAAAGAAAGCACATATGTGGGATGACTGGAGATGGTGTGAATGACGCCCCTGCTTTGAAGAAAGCAGACATTGGAATTGCTGTTGCTGATGCTACAGATGCTGCAAGGAGTGCTTCGGACATCGTGCTTACCGAGCCTGGGCTTAGTGTTATTATCAGTGCAGTGCTTACAAGTCGCGCTATTTTCCAAAGAATGAAGAATTATACTATCTATGCCGTCTCCATCACCATTCGTATTGTGTTTGGATTTATGTTCATCGCTTTGATATGGAAGTTTGACTTCTCTCCCTTCATGGTTTTGATTATTGCAATTCTTAACGATGGTACCATCATGACCATCTCAAAGGATAGGGTGAAACCATCTCCGTTACCTGACAGCTGGAAGCTAAAAGAGATCTTTGCCACTGGAATCGTGCTCGGAGGATACCTTGCACTGATGACCGTCATATTCTTCTGGATCATGAAAGACACCGACTTCTTCACAGAAAAATTTGGTGTTAGATCTTTGAGAAATAGTGAAGTTGAGATGATGGCGGCTTTATATCTCCAAGTCAGTATAGTCAGCCAGGCTTTGATTTTTGTAACGCGTTCTCGCAGCTGGTCGTTTATTGAACGCCCGGGTCTCTTATTATTGGGTGCTTTCCTAGCAGCACAACTGGTTGCGACTCTAATAGCAGTCTACGCAGAGTGGGAATTCGCAAGAATCAAGGGAGTCGGGTGGGGATGGGCCGGTGTCATCTGGCTCTACAGCATCGTGTTCTACTTCCCTCTCGATATTATGAAATTCGCTATTCGATACATCCTTAGCGGGAAAGCCTGGCGCAACATGCTTGAAAACAAGACGGCTTTCACGACGAAGAAGGATTATGGAAGAGAAGAGAGAGAAGCCCAATGGGCTCTTGCTCAGAGGACATTGCATGGTCTTCAAGCACCGGAAACCTCTAATATCTTTAACGAAAAGAGCAGCTACAGAGAACTGTCGGAAATAGCGGAACAGGCCAAGAGACGTGCTGAAGTTGCAAGACTTCGAGAGGTGCTTACACTGAAGGGGCATGTTGAGTCGGTGGTGAAGCTGAAGGGGCTGGATATCGACACAATCCAACAGCATTACACAGTGTGA

# >HHA5

ATGGCGGATTCTGGCATCAGTTGGGAGGAACTCAAGAAGGAGAACGTCGATCTCGAGACTGTTCCGGTTGACGAGGTATTTGAAACCTTGAAATGTACAAGACAGGGATTGACAACTGAAGAAGGGAACAGGAGGCTTAATGCATTCGGCCCGAACAAGCTGGAAGAGAAGAAGGAGAGTAAATTTCTCAAGTTCTTAGGGTTCATGTGGAATCCGCTCTCATGGGTCATGGAGGCTGCCGCAATTATGGCCATTGTCTTGGCCAATGGAGGGGGAAAGCCTCCAGATTGGCAAGACTTTGTTGGAATCACAACATTGCTGATAATCAACTCGACCATCAGTTTCATAGAGGAAAACAACGCAGGCAACGCTGCAGCCGCTTTAATGGCAGGTCTTGCACCAAAAACAAAGGTCATAAGGGACGGAAAATGGGACGAACAAGACGCAGCCATTTTAGTACCCGGAGATGTAATCAGTGTTAAATTGGGAGACATTATTCCAGCTGATGCGCGCCTTCTAGAAGGTGATCCGTTAAAGATTGACCAATCTGCATTGACTGGTGAATCATTACCAGTGACCAAGCATCCAGGTGCCAGTGTGTATTCGGGCTCGACATGTAAACAAGGTGAAATTGAAGCGGTTGTTATAGCTACTGGGGTCCACACGTTCTTCGGGAAGGCTGCTCATTTAGTCGATAGTACAAACCAAGTCGGCCATTTCCAAAAGGTGTTGACCTCCATTGGAAACTTCTGCATATGCTCCATCGCGATCGGGCTCATAATCGAAATAGTGGTGATGTACCCGATTCAGAAACGAACGTATAGAAACGGAATTGACAATTTACTGGTTCTTCTCATTGGAGGAATCCCGATTGCCATGCCAACTGTTTTGTCAGTCACCATGGCCATCGGGTCCCACAAGTTGTCGGAACAAGGTGCAATCACCAAGAGAATGACAGCCATTGAAGAAATGGCTGGGATGGATGTTTTGTGTAGTGACAAAACTGGCACTCTCACCCTCAACAAACTCACAGTTGACAAAACTCTCATTGAGGTTTTTGTAAAGGATGCTGATAAGGATCAAGTGGTTCTATTGGGAGCAAGGGCTTCAAGAGTGGAAAATCAAGATGCAATAGATGCTTGTATTGTAGGCATGCTTTCAGATCCAAAAGAGGCACGAGCAGGGATCACTGAGGTGCATTTTCTCCCCTTTAACCCAGTGGACAAAAGAACAGCTATAACATACATAGATCAAAATGGAAATTGGCATAGAGTGAGCAAAGGTGCACCCGAGCAGATTGTGGAGCTATGCGACCAAAAGGAGGAGGATAAGAAGAAAGTGTTTTCCATAATCGATAAGTTTGCCGAACGTGGTCTTCGTTCTCTAGCTGTTTGCCAACAAGCGGTACCGGAGAAAACAAAGGAGAGCGCGGGAGGCCCATGGGTGTTTGTGGGTTTACTGCCACTGTTTGACCCACCAAGGCATGACAGTGCTGAAACCATTCGACGGGCTCTACACCTTGGAGTCAATGTGAAAATGATCACGGGTGATCAGTTAGCCATTGGCAAGGAAACGGGCCGAAGGCTTGGAATGGGAACAAACATGTATCCTTCTTCTTCACTTCTGGGCCAAAATAAAGATCCATCAATTGCAGATATACCAATTGAGGAGCTTATTGAGAAAGCTGATGGTTTTGCTGGAGTCTTCCCTGAACACAAATATGAAATTGTGAAGAAACTACAAGAGAGAAAGCATATATGTGGAATGACAGGAGACGGTGTGAACGATGCACCCGCATTAAAACGGGCCGACATTGGTATCGCAGTGGCTGATGCAACGGATGCAGCCCGTGGTGCATCCGATATCGTGTTGACCGAGCCTGGACTCAGTGTAATCGTCAGTGCGGTTTTGACCAGTCGCGCCATCTTTCAAAGGATGAAAAACTACACAATTTATGCCGTCTCTATCACCATCCGTATTGTGCTCGGGTTCATGTTAATTGCACTTATATGGAAGTTTGACTTCTCGCCTTTTATGGTTTTGATTATCGCGATCCTTAATGATGGAACCATTATGACTATATCAAAGGATAAAGTCAAACCTTCACCTCTGCCGGATTCATGGAAGTTGAACGAAATCTTTGCCACTGGGATTGTTCTTGGCACTTATTTAGCTGTTATGACTGTAGTTTTCTTCTGGCTAGCAAAGGAATCTGACTTCTTTACGGAGAAATTTGGTGTGAAGCCGATTAAAGACAATGAATACGAGCTTATGTCAGCTCTTTACCTACAAGTCAGCATCATAAGTCAGGCACTCATTTTTGTTACAAGATCAAGAAGCTGGTCTTTCGTTGAACGCCCCGGTCTTTTACTGCTCACCGCCTTTTTTATAGCACAGCTGATCGCTACATTAATCGCTGTCTACGCAAACTGGGACTTCGCAAGAGTCCATGGAATCGGCTGGGGTTGGGCCGGTGTGATTTGGCTCTATAGCATACTCTTCTATTTCCCATTAGACGTCTTCAAATTCATCATCCGGTTCGCCTTGAGTGGCAAGGCATGGGACAACATGCTCCAAAATAAGACTGCTTTCACCACTAAAAAGGACTATGGACGGGGCGAGAGGGAAGCCCAATGGGCCTCGGCCCAACGCACCTTGCATGGCCTCCAAGCACCGAATGCAAACGACATTTTCAGCGATAAGAGCGACTACAGAGAGTTGTCGGAACTTGCAGAACAAGCAAGGAGGCGGGCGGAGGTTGCTAGGTTGAGGGAGCTTCATACCCTCAAAGGGCATGTCGAGTCGGTGGTGAAGCTCAAGGGTCTGGATATTGAAACCATCCAACAACACTACACGGTTTAA

# >HHA6

ATGGCTGCTTCCGGCATCAGTTGGGAGGAACTCAAGAAGGAGAACGTCGATCTCGAGACTGTTCCAGTCGATGAGGTATTCGAAACGTTGAAATGTACAAAAGCGGGATTGACGACTGAAGAAGGGAACAGGAGGCTTGCTGCTTTTGGCCCTAACAAGCTAGAAGAGAAGCATGAAAGTAAGCTTCTCAAGTTCTTAGGGTTCATGTGGAATCCATTGTCATGGGTCATGGAAGCCGCTGCAATCATGGCTATCGTCTTGGCCAATGGAGGGGGAAAGCCTCCGGATTGGCAAGATTTTGTTGGTATCACAACATTGCTGATAATCAACTCCACCATTAGTTTCATCGAGGAAAACAACGCAGGTAACGCTGCAGCGGCTTTAATGGCGGGTCTTGCTCCGAAAACTAAGGTCATAAGGGACGGGAAATGGGATGAACAAGACGCGGCTATTTTAGTACCCGGGGATGTGATTAGTGTCAAATTGGGAGATATTATTCCAGCCGATGCGCGCCTTCTTGAAGGAGATCCACTTAAGATTGATCAATCTGCGTTGACCGGTGAATCGTTGCCTGTGACCAAACATCCGGGTCAAAGTGTGTATTCCGGGTCAACATGCAAACAAGGTGAAATCGAAGCGGTTGTTATCGCTACTGGGGTCCACACTTTCTTCGGGAAGGCTGCTCACTTAGTAGATAGTACAAATCAAGTTGGCCATTTCCAAAAGGTGTTGACCTCTATTGGAAACTTCTGCATATGCTCCATTGCCATTGGTCTCATAATCGAAATAGTGGTGATGTACCCGATTCAGAAACGCTCATACAGAAACGGAATCGACAATCTATTGGTTCTACTCATCGGAGGGATCCCAATCGCCATGCCAACTGTTTTGTCAGTCACCATGGCTATCGGGTCCCACAAGTTGTCGGAACAAGGTGCAATCACCAAGAGAATGACCGCCATTGAGGAAATGGCGGGCATGGATGTTTTGTGTAGTGACAAAACTGGTACTCTTACGCTTAACAAACTCACAGTCGACAAAACGCTCATTGAGGTTTTCGCAAAGGATGTTGATAAGGATCAAGTGGTTCTATTGGGAGCCAGGGCTTCAAGAGTTGAGAATCAAGACGCGATTGATGCGTGCATTGTAGGAATGCTTTCGGATCCTAAAGAGGCACGAGCGGGGATCACTGAGGTGCATTTTCTGCCTTTCAATCCTGTAGACAAACGAACAGCAATAACGTACATAGATCAAAGCGGAAATTGGCACAGAGTGAGCAAAGGTGCACCTGAGCAGATTGTGGAGCTATGCAACCAAAAGGATGAGGATAAGAAAAAAGTGTTTTCGATCATTGATAAATTTGCCGAACGTGGTCTACGGTCTCTTGCTGTTTGCCAACAAGCGGTACCAGAGAAAACAAAGGAGAGCCCGGGAGGCCCGTGGGTGTTCGTGGGCTTACTGCCACTATTTGACCCACCAAGGCATGACAGTGCGGAAACCATTCGACGGGCCCTGCACCTTGGAGTCAATGTCAAGATGATCACTGGTGATCAGTTAGCCATTGGGAAAGAAACAGGTCGCAGGCTCGGTATGGGAACAAATATGTATCCCTCTTCTTCACTTCTTGGCCAAAGCAAAGATTCTTCTATTGCGGATATACCGATAGAGGAACTAATCGAGAAGGCTGACGGTTTTGCTGGAGTCTTCCCCGAACACAAATACGAGATTGTGAGGAAACTACAAGAGCGAAAGCATATATGCGGAATGACAGGAGATGGTGTGAACGATGCACCCGCATTGAAACGGGCCGACATTGGTATAGCGGTCGCTGATGCAACAGATGCAGCTCGTGGTGCATCCGATATAGTTTTAACTGAGCCTGGGCTCAGTGTGATTGTAAGCGCGGTTTTGACCAGCAGGGCCATCTTTCAGAGGATGAAAAATTACACCATTTATGCAGTTTCGATCACCATCCGTATCGTGATCGGGTTCATGCTGATTGCGCTTATCTGGCAATTCGATTTCTCGCCGTTTATGGTTTTGATTATTGCAATCCTCAATGATGGAACCATTATGACTATTTCTAAAGATAAAGTCAAACCTTCACCTCTGCCGGATTCATGGAAATTGAAAGAAATCTTTGCAACTGGTATTGTTCTTGGGACCTATTTGGCGGTTACGACCGTTATTTTCTTCTGGTTGGCGAAAGAATCCGACTTCTTTACTGAGAAATTTGGCGTGAAACCGATCAAAGACGAAGAATTTGAGCTTATGTCAGCTCTTTACCTTCAAGTCAGCATCATCAGTCAGGCACTCATTTTCGTTACAAGATCACGAAGCTGGTCGTTCGTTGAACGCCCCGGTCTTTTACTCCTCACCGCCTTTTTTATAGCTCAGCTGATTGCTACTTTAATTGCTGTCTACGCGAATTGGGATTTCGCAAGAGTCCATGGAATCGGCTGGGGTTGGGCCGGTGTGATTTGGCTTTATAGCATAATCATCTATTTCCCGTTAGACATTTTCAAATTCATCATCCGTTACAGTTTAAGCGGCAAGGCTTGGGACAGTATGATCGAAAAGAGGACCGCTTTCACCTCAAAGAAGGACTATGGGCGCGGAGAGAGGGAAGCCCAATGGGCCACGCACCAACGCACCTTGCATGGTCTCCAAGCACCAAATGCAAACGATATCTTGAACGACAAGAGCGACTATAGGGAGTTGTCGGAACTAGCAGAACAAGCAAAGAGGCGGGCCGAGGTTGCTAGGTTAAGGGAGCTCCACACACTTAAAGGGCATGTTGAGTCAGTGGTGAAGCTCAAGGGCCTCGATATTGAAACGATCCAACAACACTACACCGTTTAA

# >HHA7

ATGGCAGAGAGTGAGGGCATCACATGGGATGATATCATGAAGGAGACTGTAGATCTTGAGCATATGCCAATGGATGAGTTATTTGATCAATTGAAATGCACAAAAGAGGGATTGACAAGTGAAGAAGGCAAAAGAAGGCTTGGAATATTTGGTCCAAATAAATTGGAGGAGAAAAAGGAAAGCAAATTTCTCAAGTTTTTAGGTTTTATGTGGAATCCTCTTTCATGGGTTATGGAAGCTGCTGCTATCATGGCCATTGCCTTAGCAAATGGAGGGGGGAAGCCACCAGATTGGCAAGATTTTGTAGGAATAACAACATTGTTATTAATCAATTCAACCATAAGTTTCATAGAAGAAAACAATGCAGGAAATGCTGCAGCAGCATTAATGGCAGGTTTAGCTCCTAAAACAAAGCTTTTAAGGGATGGAAAATGGGCCGAAGCCGAAGCTGAGTTTCTCGTACCCGGAGATATCATTAGCATTAAGCTCGGTGATATTGTACCCGCTGATGCACGTCTCCTAGAAGGGGACCCCCTCAAGATTGACCAGGCTGCGTTGACCGGTGAGTCATTGCCGGTAACTAAGAAACCTGGGAATAGTGTTTTTTCGGGTTCGACGTGTAAACAAGGTGAGATCGAAGCTGTTGTGATCGCTACTGGGGTCCACACTTTCTTTGGTAAGGCGGCTCACTTAGTCGATAGCACAAATCAAGTCGGCCACTTCCAAAAGGTATTAACGGCTATCGGTAACTTTTGTATATGCTCGATTGCTGTCGGGCTGATAATAGAAATAGTGGTGATGTACCCGATTCAGAAACGAACCTACAGAAACGGAATCGATAACTTGCTGGTTTTACTCATAGGCGGCATCCCAATCGCAATGCCAACCGTGTTGTCGGTTACAATGGCTATCGGGTCCCACAAGTTATCACAGCAGGGAGCGATTACCAAACGGATGACAGCCATTGAAGAAATGGCTGGGATGGATGTTTTGTGCAGTGACAAGACTGGCACTCTCACACTTAACAAGCTCACAGTTGATAAAAGTCTTATTGAGGTCTTTGTAAAGGATATGGATAAGGACACAGTGATTTTAATGGGAGCAAGGGCTTCAAGAGTGGAGAATCAAGATGCAATTGATGCTTGCATTGTAGGAATGCTTGCTGATCCTAAGGAGGCAAGGGCGGGGATCAACGAGGTACATTTTCTACCATTTAACCCTGTGGATAAACGAACAGCGATAACATACACAGATCAAGATGGAAATTGGCATCGAGTGAGCAAAGGTGCACCCGAGCAGATTGTGGAGCTTTGCAACCTAAAGGGTGATACTAGCAAAAGGGTTTTCGACATTATTGACAAATTTGCTGAACGGGGTCTTCGTTCTCTTGCTGTTTGTCAACAGACGGTACCTGAAAAAACGAAAGAGGGTCCTGGCGGGCCATGGGTATTCGTTGGTCTCCTACCATTGTTTGACCCGCCAAGACATGACAGTGCTGAAACAATTAGGCGGGCCCTACACCTTGGTGTGAATGTGAAGATGATCACCGGTGATCAACTAGCCATTGGTAAAGAAACGGGCCGGAGGCTCGGAATGGGGACAAATATGTACCCCTCTTCATCCCTCTTGGGCCAACACAAAGACGCATCTATCGCTAATATTCCTATCGAGGAGCTAATCGAGCAAGCTGATGGCTTTGCTGGCGTTTTTCCTGAGCACAAGTATGAAATCGTGAAGAAGTTACAAGAGAGAAAACATATATGCGGAATGACAGGAGACGGTGTAAACGACGCACCAGCACTAAAACGGGCCGATATCGGTATCGCGGTTGCTGATGCAACCGATGCAGCCCGAGGTGCATCCGACATAGTCTTGACTGAGCCTGGGCTAAGCGTGATTGTAAGCGCGGTACTTACTAGCCGAGCCATCTTTCAGAGGATGAAAAACTACACCATATATGCTGTCTCCATCACCATTCGTGTCGTTCTTGGTTTCATGCTACTCGCACTGATCTGGAAGTTTGATTTCTCGCCTTTCATGGTTCTCATTATTGCAATTCTTAATGACGGAACCATCATGACCATATCGAAGGATAAAGTCAAGCCTTCACCGATGCCGGATTCATGGAAATTGAAGGAAATATTTCTCACTGGGATCGTTTTCGGGACTTATTTAGCGGTCATGACCGTAATTTTCTTCTGGCTAGCACAAGACTCTGACTTCTTTCCAGATAAATTCGGTGTAAGATCGATTAGAAACAAGGACTACGAGCTTACGGCAGCTTTGTACCTCCAAGTCAGCATTGTTAGTCAAGCGCTTATTTTCGTCACTAGATCAAGAAGTTGGTCGTATGTGGAACGACCCGGTCTTCTGCTTCTGACAGCCTTTTTGATCGCACAGCTGATAGCTACCCTGATCGCGGTCTATGCACACTGGGATTTTGCGAGAATCAACGGAATAGGTTGGGGATGGGCCGGTGTGATTTGGTTATATAGTATAATCTTCTACATCCCGCTAGATATTTTCAAATTCATCATACGGTATGCAATGGCTGGCAGGGCTTGGGATAATCTGCTCCAAAATAAGACCGCTTTCACTAGCAAAAAAGACTACGGGCGGGGCGAAAGGGAGGCCCAATGGGTCCAGGAACAACGGACGGTCCACGGTCTCCAGCCGCCAGAGCAGCCAGAACAGTTCGTGAACGACAAGACGAGCTACCGGGAGTTGTCGGAGCTTGCCGAACAGGCTAAGAAGCGCGCTGAAGTGGCTAGATTGAGGGAGCTTCATACAATAAAGGGTCACGTTGAATCGGTAGTGAAGCTTAAGGGTCTCGATATCGATACCATTCAACAGCACTATACGGTGTAA

# >HHA8

ATGTCTGATAATTCCTTGGAAGAAGTTAAAAGTAACCAAATTGATCTCGAAAAAATCCCTATAGAAGATGTCTTCACGATCCTAAATTGTACGCGAGATGGCTTGAACGACGAGGAAGCCACTAAGAGGCTCGACATCTTCGGTCACAATAAGCTTGAGGAGAAACAGGAAAGTAAATTGCTGAAGTTTCTAGGATTCATGTGGAATCCTCTTTCATGGGTCATGGAATTTGCAGCCATTATGGCCATTGTATTAGCCAACGGTGGCGGGAGGCCACCGGACTGGCCGGATTTCGTGGGCATAGTCGTGCTTCTTCTCATCAACTCAACCATTAGTTTCATCGAAGAAAACAATGCCGGCAATGCCGCCAGTGCCCTGATGGCAAGCCTAGCCCCGAAAGCCAAGATTTTAAGAAACGGAAAATGGAGCGAGCAAGATGCCGGCATATTGGTTCCTGGAGATGTTATTAGTGTCAAGTTAGGAGATATTATCCCAGCTGACGCTCGTCTCCTTGAAGGCGATACGTTGAAAATTGACCAGTCTGCGCTTACTGGCGAGTCGGTCCCAGTAAACAAGAACCCGGGTGAGCCGGTTTACTCTGGCTCGACGTGCAAGCAAGGCGAGATTGAAGCTGTGGTGATAGCCACGGGCGTCCGAACCTTCTTTGGGAAGGCTGCACACCTTGTGAACAGCACGGATTCAGCGGGTCATTTCCAACAGGTGTTGACATCCATTGGTAACTTTTGCATATGCTCAATTGCGATTGGAATGGTGATCGAGATTGTGGTGATATGGGTGATTCAAGGAAGAGGGTATAGAGACGGTATCGATAATTTGTTGATCCTTTTGATCGGAGGTATCCCGATCGCCATGCCTACAGTTTTGTCTGTAACGATGGCGATCGGGTCCCACCACCTTGCAAAACAAGGTGCCATCACGAAAAGGATGACCGCCATTGAAGAAATGGCGGGTATGGATATTCTTTGTAGCGATAAAACCGGTACCCTCACTCTTAACAAGCTAACGGTCGACAAATCGTTGATTGAGGTTTTCGTCAAGGATTGCGATAGGGACATGGTGGTGATGTATGGTGCTCGAGCTTCAAGGATCGAAAACCAAGACGCCATCGATGCTTGCATCGTCAACATGTTGGCTGATCCAAAAGAGGCTCGAGCCGGAATCAAAGAGGTCCACTTCCTACCGTTTAACCCTGTCGACAAACGCACAGCCATCACCTACATTGACAATAAGGGTGACTGGTATCGGTCCAGCAAGGGTGCACCAGAACAAATCATCGAACTTTGTAATCTCACCGGCGATACACTAAAGAGGGCTGAGGAAATCATTGACGGGTTCGCAAACCGAGGTCTTCGGTCTCTCGGCGTGGCTCGACAAACCGTACCCGAGAAGACAAAGGAGAGCGAGGGCTCACCTTGGGAGTTTGTAGGACTGTTGCCGCTTTTCGATCCACCACGACACGATAGTGCCGAGACTATTAGAAGGGCTTTAGAGCTTGGTGTTAAAGTAAAAATGATCACCGGAGATCAACTCGCCATTGGGAAAGAAACCGGCAGAAGACTTGGTATGGGTACCGACATGTATCCGTCATCTTCGCTTCTCTCGGAAAGCAATGATGCTAACAACTCTTCGATCGATGACCTCATTGAGAAGGCCGATGGCTTCGCCGGAGTTTTTCCCGAACACAAATACGAAATCGTCAAGAGGTTACAACAGAGGAATCACATATGCGGTATGACGGGAGATGGTGTGAACGATGCACCAGCACTCAAGCGAGCAGACATCGGTATAGCAGTGGATGACTCCACAGACGCTGCCAGGAGTGCGTCCGACATTATCTTGACCCAGCCGGGGCTTAACGTGATAGTCGCAGCTATACTAACAAGCCGAGCCATCTTCCAAAGAATGAAAAACTACACAATATACGCAGTCTCCATCACCATCCGAATTGTGATGGGGTTCATGCTCATTGTTGTCATATGGAGGTTTGACTTCTCGCCTTTCATGATTCTCGTCATCGCTATTCTTAACGATAGCACCATCATGATGATCTCCACTGACCGGGTTAAACCGTCACCACTTCCCGACTCATGGAAGCTCAATGAGATTTTCGCCACTGGTATCGTCCTCGGGACTTATCTATCACTCGTTACTGTTTTGTTCTTTTGGCTATCCTCCAGAACCGACTTCTTCCCCAGGCTTTTTGGTGTTCGATCGATTGTTGGAAACGATGATGAGGTGACCGCAGCGCTATACCTTCAAGTGAGCATCATTAGTCAAGCACTCATATTTGTGACGAGATCTCAAAGTTGGTCATTCCTCGAGCGCCCTAGCACCTTGCTCATGCTCGCATTCGTACTAGCTCAGATATTGGCTACACTTCTAGCAGTATATGCAGACTGGGATTTTGCAGAGATGCAAGGGATCGGATGGCGATGGGCCGGGGTAATCTGGATGTTCAGTATCGTTACCTACATCCCTCTAGATATACTAAAATTCATTATCCATGCGGCATTGAACGGGAACAACTCTAGATAG

# >HHA9

ATGCATTTCTATTTTGATGTGCATATGGAAAACAAGTCTAGAAAGTATTGTCCCGTGCGACGTCATTGGGAGCCTGAGGACCAGCATATCTGTCTTGCTTATGATCATGGGTCAGTGGTTTGCGCAACTCCAAACACGTGGGAATCAAGTAGTGCAAGCACTTCTAGGGTACATAGTAACCGTGCAAAGACTCATCAGGGAGTGACCCTAACTCTCAACCAGAAGGACCCTAGAAGGGTCAAAACTTACGGCCGAAAAATGGATGATAAGTCAATCGCTCTCAGTGCAGTCATCCGAGAAGCCATCGATTTGGAGAATGCACCCGTAGAAGAAGTGTTTCAACACCTGAAATGTACAAGAGAAGGGTTAAATTCGAGTGAAGTTCAAGAGCGTTTGGATTTGTTTGGCTACAATAAACTCGAAGAAAAGAAGGAAAGTAAAATCTTGAAATTTCTTGGATTCATGTGGAATCCACTCTCATGGGTGATGGAAGCAGCTGCTATCATGTCAATCGCACTTGCACGTGGAGGGGGAAAGTCTGCTGATTACCACGACTTTGTCGGGATCATCGTCTTGCTTGTTATAAACTCAACCATAAGTTTCATAGAGGAAAATAACGCGGGAAATGCAGCTGCTGCATTGATGGCCCGTTTAGCGCCAAAAGCAAAGGTTCTTCGTGATGGAAAATGGAGTGAAGAAGACGCTTCGTTATTGGTTCCAGGTGATATTATTAGTATCAAGTTAGGTGACATTGTTCCTGCTGACGCTCGTCTTTTAGAAGGCGATCCGTTAAAGATTGATCAGTCTGCACTTACTGGAGAATCACTGCCCGTGACGAAGAATCCGGGAGATGGTGTTTACTCGGGTTCCACGTGTAAGCAAGGAGAGATCGAGGCCGTTGTGATAGCGACCGGAGTTCATACGTTTTTTGGGAAAGCGGCTCATCTTGTCGAGAACACGACTCATGTTGGACATTTTCAGAGGGTTCTAACTTCAATAGGAAACTTTTGCATATGTTCAATAGCTATTGGTATGATAATTGAAGTTATTGTTGTATATGGTATCCATCAACGGGAATACCGTGTTGGAATCGATAACCTTCTAGTTCTGCTGATCGGCGGGATCCCTATCGCGATGCCAACGGTCCTTTCGGTCACCATGGCCATCGGGTCACACCGTTTGTCTCAACAGGGCGCGATAACGAAAAGAATGACCGCGATTGAAGAAATGGCCGGGATGGATGTGTTGTGTAGTGACAAGACCGGTACATTGACACTTAACAAGCTTACGGTTGACAAGAACATGATCGAGGTCTTTGCGAAAGACGTTGACAAGGATATGGTCGTGTTGATGGCTGCCCGAGCATCGCGGTTAGAGAATCAGGATGCGATCGATGGTGCTATCGTTGCTATGCTTGGAGACCCCAAGGAGGCCCGAGCTGGGATTCGAGAGGTCCATTTCCTACCGTTTAATCCAACGGACAAAAGGACCGCGCTTACGTACACAGATAAAACCGGTAAAATGCATAGAGTTAGCAAAGGCGCACCCGAGCAGATACTGAACCTTGCACATAACAAATCCGAGATCGCGAACAAGGTACATTCGATAATCGACAAGTTTGCAGAGCGTGGGCTTCGGTCGCTTGGAGTCGCTCGCCAGGAAGTGCCAGCTGGAACGAAAGAGAGCCCGGGTGGCCCATGGGAGTTCGTCGGGCTTCTTCCGCTGTTTGACCCTCCACGTCATGATAGCGCTGAAACGATCCGAAGAGCGTTAGATCTTGGCGTGAGCGTTAAAATGATAACGGGTGACCAACTGGCAATTGGCAAGGAAACAGGAAGACGGCTCGGAATGGGGACCAACATGTACCCTTCTTCAGCATTGCTCGGTGACACTAAAGACGGTTTCGGAGCTCTGCCTGTCGATGAACTCATCGAGAAAGCCGATGGTTTTGCTGGTGTCTTTCCGGAACATAAATACGAGATAGTTAGAAGATTACAAGCCCGAAAACATATATGTGGGATGACGGGTGATGGTGTAAACGATGCACCCGCTCTAAAGAAAGCAGATATCGGGATCGCAGTGGCGGATTCCACTGATGCTGCTCGTAGTGCTTCGGATATTGTTTTAACCGAACCCGGGTTAAGTGTCATTATTAGCGCGGTTTTAACTAGCCGATCCATTTTTCAAAGAATGAAGAATTATACGATATATGCGGTTTCAATCACTATTCGTATCGTGCTTGGTTTTATGTTGCTGTGTGTTTTCTGGAAGTTCGATTTCCCTCCGTTTATGGTTCTCGTTATAGCCGTCCTTAACGATGGTACGATCATGACAATATCGAAAGATAGAGTAAAGCCGTCTCCGATTCCCGACAGCTGGAAGCTCACGGAAATCTTCGCGACCGGGGTGGTTCTCGGTGCTTACCTGGCACTAATGACAGTTATTTTCTTCTGGTTGGCATATGAAACTAACTTTTTCCCGAACTTATTCAGTGTCAAGGATTTGAACTCACATCATCGCGACATGTCAATCAAATCCGAGAAAGAAGAGCTAACAGCGATGATGGCGTCAGCTGTTTATCTTCAAGTGAGCACCATCAGCCAAGCGTTGATCTTCGTGACACGTTCGCGCGGCTGGTCTTTTACTGAGAGGCCCGGGTTTTTGCTAGTAACTGCATTTTTTATTGCTCAACTGGTTGCGTCGATTATATCGGCCCATGTGACGTGGGAACTAGCCGGAATCCAAAAGATCGGATGGGGATGGACCGGGGTGATATGGTTGTATAACATCTTGACTTACATGTTACTTGATCCGCTCAAGTTTGCGGTTCAGTATGGGCTAAGTGGGAGAGCTTGGGGTCTAGTTGTGGAGAAAAGAACTGCATTTACGACGAAGAAAGACTTTGGGAGGGAAGCGCGTGAGGCAGCATGGGCAACGGAGCAACGCACATTGCATGGACTCCAGCCAGCAGAGCCAAGAACGTTTCCGGATCAAGGGACATTTAGGGAGATTAGTGTGATGGCCGATGAAGCTAGACGAAGAGCGGAGATCGCAAGGCTAAGAGAGCTTCATACACTTAAGGGCAAAGTTGAGTCTTTTGCAAAGCTAAGAGGGTTAGACATTGATGCAGCTAACCAACACTACACAGTTTAA

# >HHA10

ATGGAAGAAGTTTTCGAGAATTTAAGATGCACGAAAGAAGGACTGAACTCTGATGAGGTTGAAAAGCGTTTGAATATGTTTGGCTACAATAAGTTGGAAGAGAAGAAGGAAAGTAAAATTCTGAAGTTTTTGGGTTTTATGTGGAATCCTCTGTCTTGGGTAATGGAAGCAGCAGCAATCATGGCAATTGCTATGGCTCGAGGAGGGGGTGAACCTGCTGACTATCACGATTTTGGTGGCATAGTGGTACTTCTGCTTATCAATTCAACCATAAGTTTTGTAGAAGAGAATAACGCTGGAAACGCAGCTGCAGCCCTCATGGCTCGTTTGGCTCCAAAAGCTAAGGTTTTACGCAATGGGAAATGGAATGAAGAGGATGCATCAATACTTGTACCTGGAGACATCATAAGTATTAAGCTAGGTGACATTATTCCTGCTGATGCACGTCTGCTTGAAGGAGATCCTTTAAAGATTGATCAATCAGCTCTTACAGGTGAATCTCTCCCAGTAACGAAGAATCCAGGTGACGGGGTGTACTCAGGGTCCACGTGTAAACAAGGTGAAATTGAGGCAGTTGTTATTGCGACAGGGGTCCATACGTTCTTTGGGAAAGCAGCTCATCTTGTGGACAACACTACACACATAGGTCACTTTCAACAGGTTTTGACTGCGATTGGGAACTTCTGCATATGCTCAATTGCGATAGGCATGGTTATTGAAATAATTGTGATATATGCTCTTCAAAAGAGGCATTATCGTGAAGGAGTCGATAACCTTCTCGTGATACTCATTGGTGGGATCCCAATTGCAATGCCAACTGTTTTATCTGTTACAATGGCCATTGGCTCCCATAAACTAGCTCAACAGGGAGCTATAACAAAGAGAATGACCGCCATTGAAGAAATGGCTGGAATGGATGTATTGTGTAGTGATAAAACTGGAACTTTAACTCTCAACAAACTTACTGTTGACAAAAATTTGATTGAGGTTTTTGCAAGTGGTGTTGATAAGGACACGGTTGTGTTAATGGCTGCTAGAGCATCAAGGTTGGAAAACCAAGATGCCATTGATGCTTGTATAGTATCAATGCTGGCTGATCCTAAGGAGGCGCGATCCGGAATTACAGAAGTGCACTTCCTTCCATTTAATCCAACTGATAAGAGGACAGCTTTGACATACATTGACGGTGCTGGCAAGATGCATAGAGTTAGCAAAGGTGCACCAGAACAGATTTTGAATCTGTCAAAAAACAAATCGGAGATTGAAAAGAGGGTACACGCAATCATTGATAATTTCGCTGAACGTGGACTTCGATCTCTTGGAGTGGCTCGCCAGGAAGTACCAGCTAACAGTAAAGATAGCCCTGGTGGTCCGTGGGAATTTGTGGGGCTTCTTCCTCTATTCGATCCACCTCGCCATGACAGTGCTGAGACAATTAGAAGAGCTTTAGATCTTGGAGTTAGTGTTAAGATGATCACCGGTGACCAACTGGCCATTGCTAAGGAGACTGGGAGACGACTAGGAATGGGTGTTAACATGTATCCTTCATCATCCTTACTTGGCGATCATAAGGATCAACTACTTAGAGCTTTACCTGTTGATGAACTTATTGAAAAAGCCGATGGTTTTGCTGGTGTTTTTCCAGAGCACAAGTATGAAATTGTGAAGATTCTTCAAAGTAAAAAGCACATTTGTGGAATGACGGGTGATGGTGTAAACGATGCACCTGCATTAAAGATAGCCGACATTGGAATTGCAGTGGATGATGCCACAGATGCAGCTCGAAGCGCGTCTGACATAGTTCTAACTGAGCCGGGGCTCAGTGTCATCATCAGCGCGGTGTTAACAAGCCGAGCCATCTTCCAGAGAATGAAAAACTATACAATCTATGCCGTTTCAATCACCATACGTATTGTGTTAGGGTTCATGTTGCTGACTTCTTTTTACGAATTTAATTTCCCTCCAATTCTTGTTCTTGTCATAGCCATTCTGAATGACGGTACGATCATGACTATTTCCAAAGATAGAGTGAAACCATCTCCCAGCCCAGACAGCTGGAAGCTCAGTGAAATTTTTGCAACTGGAATAGTCATAGGCACCTACCTTGCTCTAATGACTGTCTTATTCTTTCATTTGGCTAGTCAAACCAATTTTTTTGCGCATACCTTCCATGTGGAAAGTTTACATAAGCATAAAGGCCTCGCAGATGATGTTTGGAAAGCGAAGCTAGCATCAGCTGTATACCTCCAAGTCAGCACAATTAGCCAGGCGTTAATATTTGTCACACGCTCCAGGGGTTGGTCTTTCACGGAAAGACCAGGTCTTCTGTTGCTTGCTGCATTCATTCTAGCTCAACTCTTTGCCACTGTGATGTCAGCATATTTGAGCTGGAGTTTTGCTAAGGTCCATGCGATTGGTTGGGGTTGGACTGGAGTTATATGGTTGTATAACATCTTAAGCTACATGTTGCTTGACCCTATCAAATTCGCCGTTAGATATGCGCTTAGTGGGAGAGCATGGGGCCATGTTATTAACCGTAAAACGGCATTCAGCACCCAAAAGGACTTTGGTAGGGAAGCCCGCGAGGCAGCATGGGCAAAAGAGCAAAGAACACTTCATGGTCTTGATACTTCTGAAGCAAAGCCTTTTGCTGAAAACTACACCTTCAGAGACATCAACATGATGGCGGAAGAAGCAAAACGTCGTGCAGAGATTGCAAGATTAAGGGAACTTCATACCCTGAAGGGAAAGGTAGAGTCTTTTGCGAAGCTAAGAGGATTAGACATCGATGTGAACCCACACTACACTGTGTGA

# >HHA11

ATGGCGGAGGCGGATGATATTATGGAAGCCGTGAAGAAGGAAGCGGTTGATTTGGAAACGATACCAGTCGAGGAAGTTTTCGAAAATTTGAGATGTTCCAAAGAAGGGCTAACATCAAAGGATGCTGAAAAGAGATTAGAGATATTTGGGCACAATAAGCTTGAAGAGAAGGAGGAAAGCAAAATTTTGAAGTTCTTAGGGTTTATGTGGAACCCACTATCATGGGTCATGGAAGTTGCAGCAATTATGGCCATTGCACTTGCCAATGGAGGAGGTAAACCACCTGACTGGCAGGATTTTGTTGGGATTATCACACTGCTTGTCATAAATTCAACAATAAGCTTTATAGAGGAGAACAATGCTGGTAACGCAGCAGCAGCTCTCATGGCACACCTTGCACCTAAAGCGAAGGTGCTTCGCGATGGAAAATGGAATGAGGAAGAAGCAGCAATTCTTGTTCCAGGTGATATAATCAGTATTAAACTAGGAGATATCATCCCAGCAGATGCTCGACTTCTTGAGGGTGATCCTTTAAAGATAGACCAGTCTGCTTTAACAGGTGAATCCCTTCCTGTAACAAAGCTCCCTGGGGATGGAGTTTATTCAGGCTCTACATGCAAACAGGGGGAAATTGAAGCAGTGGTTATTGCGACAGGTGTGCATACCTTCTTTGGAAAGGCTGCACATCTTGTAGATTCTACAAACCAAGTAGGCCATTTTCAGAAGGTTCTGACAGCGATTGGGAACTTCTGTATATGTTCAATTGCAATCGGAATGGTAGTAGAAATCATCGTGATGTACCCAATTCAAAACCGTAAATATCGTCCTGGAATTGACAATTTGCTAGTGCTTCTCATTGGTGGAATTCCTATTGCAATGCCAACAGTTTTATCGGTCACAATGGCAATTGGTTCTCATCGTTTATCTCAGCAGGGAGCTATCACAAAAAGAATGACAGCAATAGAAGAAATGGCAGGCATGGATGTTCTTTGCAGTGATAAAACTGGGACTTTGACCCTGAACAAGCTTTCGGTTGACAAAAATCTTATAGAGATATTTGCTAAAGGAGTGGATGCTGATATGGTGGTGTTGATGGCTGCAAGAGCTTCTAGGGTGGAAAACCAAGACGCAATAGATGCTGCCATAGTAGGGATGTTGGCTGACCCTAAGGAGGCACGGGGAGGTATTCAAGAGGTCCATTTCCTTCCATTCAACCCTACCGATAAGAGGACAGCATTAACTTACATTGATGGTGAAAACAAAATGCATCGTGTCAGTAAGGGTGCTCCTGAACAGATTTTGAATCTTGCACATAATAAATCAGAAATTGAGCGTAGAGTACATCTTGTGATAGATAAGTTTGCCGAGCGTGGTTTACGATCCCTTGCAGTAGCCTACCAGGAAGTCCCTGCGGGAAAGAAGGAAAGCCCAGGAGGTCCATGGGAGTTTATCGGTCTTATGCCTCTCTTTGACCCACCCAGACATGACAGTGCAGAGACAATTAGAAGGGCTTTGAATCTGGGAGTAAGCGTCAAAATGATCACAGGCGATCAGCTAGCAATAGGAAAGGAAACAGGACGGCGTCTAGGAATGGGGACCAACATGTACCCTTCATCTGCATTACTGGGACAGAACAAAGACGAGTCAATTGCCGCCTTACCCATTGACGAGTTGATTGAAAAAGCTGATGGATTTGCTGGTGTCTTTCCTGAGCATAAATATGAAATTGTGAAACGTTTACAAGCAAGAAAGCATATATGTGGTATGACTGGTGATGGAGTGAATGATGCACCTGCACTTAAGAAAGCGGACATTGGAATTGCGGTTGCAGATGCAACTGATGCTGCACGTAGTGCGTCTGATATAGTGCTGACTCAACCTGGTCTTAGTGTTATTATCAGTGCAGTACTAACTAGCCGAGCAATCTTTCAGAGGATGAAAAATTACACTATATACGCAGTTTCCATTACTATCCGTATAGTGCTTGGTTTCATGCTACTAGCCCTCATATGGAAGTTTGACTTTCCACCCTTCATGGTCCTGATCATTGCAATTCTTAACGACGGTACCATTATGACAATTTCCAAGGATAGAGTGAAACCGTCTCCTCAACCGGACAGCTGGAAATTGGCAGAGATTTTCGCTACAGGGATCATTCTCGGTAGCTACTTGGCTATGATGACTGTAATTTTCTTCTGGGCTGCGTATGAGACCGATTTTTTCCCGCGTGTTTTTGGAGTTTCTAGCCTTCAGAAAACTGGTCAAGTTACATTGGACGATGTTAAAAAAAAGCTAGCCTCAGCAGTATACCTACAAGTGAGCACCATCAGTCAAGCTTTAATATTTGTTACACGTTCTAGGAGCTGGTCCGTTTATGAACGTCCAGGAGCTCTACTTTTCGGTGCCTTCTTGATTGCTCAATTGATTGCAACGTTGATTGCAGTTTATGCAGACTGGAATTTTTCTGCAATCGAAGGGATCGGATGGGGTTGGGCAGGTGTAATATGGCTCTACAACATTGTATTCTACATCCCACTCGATTTCATAAAGTTGTTCATCAGATATGCTATAAGTGGTAGAGCATGGGATCTTGTCATTGACCAACGCGTTGCTTTCACCAGGAAAAGAAACTTTGGGAAGGAAGACCGTGAGCTTAAATGGGCTCAAGCACAACGGACACTACACGGGCTGGACCCGCCTGAAATACATAGCGTTGACCGCAACAACCACAATGAACTTAATCAGATGGCTGAAGATGCAAAACGCCGAGCTGAGATGACAAGGTTGCGAGAGTTGCTTACGCTGAAAGGTCACGTGGAATCAGTTGTAAAACTGAAGAACATCGACATAGATACCATCCAACAATCCTACACTGTGTGA

# >HHA12

ATGGGGGAAGAGAAGCCTGAAGTTCTTGAAGCTGTTTTGAAGGAGACTGTAGATTTGGAGAGTATACCCATTGAGGAGGTTTTTGAAAATCTGAGATGTAGCAAAGAGGGTCTCACTACTGCTGCTGCTGAAGAAAGATTAGTCATTTTTGGGCATAATAAACTTGAAGAAAAAAAGGAGAGCAAATTCTTGAAGTTTTTAGGGTTTATGTGGAACCCATTATCATGGGTTATGGAAGCTGCTGCTATTATGGCCATTGCCCTTGCAAATGGAGGAGGGAAGCCTCCTGATTGGCAAGATTTTGTGGGTATTATTACTTTGCTAATTATCAATTCCACAATTAGTTTTATTGAAGAAAACAATGCTGGTAATGCTGCAGCTGCTCTCATGGCTCGTCTTGCCCCGAAAGCAAAGATTTTACGCGATGGGAAATGGAACGAGGAAGATGCTTCTATGTTGGTTCCTGGTGATATAATTAGCATAAAGTTAGGTGATATTATTCCAGCAGATGCTCGTTTGCTCGATGGCGACCCTTTAAAGATTGACCAGTCGGCTCTAACCGGCGAGTCTCTCCCGGTCACAAAAGGTCCCGGAGATGGTGTTTATTCCGGTTCGACTTGCAAACAAGGTGAAATCGAAGCGGTTGTGATTGCCACCGGGGTCCACACGTTCTTTGGTAAAGCTGCTCATCTTGTTGATAGCACAAATCAAGTCGGTCACTTTCAAAAAGTTTTGACTGCGATCGGGAATTTCTGTATTTGTTCTATTGCTGTTGGAATGGTTATCGAAATTATCGTAATGTTCCCGATTCAAGATAGGCAATATAGGCCCGGAATCGATAATCTTCTCGTGCTTTTGATCGGAGGCATCCCGATTGCTATGCCTACCGTTCTTTCCGTAACGATGGCAATCGGGTCTCATCGATTGGCTCAACAGGGAGCGATTACGAAAAGAATGACAGCGATAGAAGAAATGGCGGGAATGGATGTGCTATGCAGTGACAAGACCGGGACGTTGACTTTGAACAAGCTTACAGTAGACAAGAATCTTATTGAAGTGTTTTCCAAAGGAGTAGATGCCGATACCGTTGTTCTGATGGCAGCGCGTGCCTCCAGAACCGAAAACCAGGATGCCATCGATGCTGCAATTGTCGGTATGCTGGCTGATCCAAAAGAGGCGCGTGCGGATGTTCAAGAGTTGCACTTTCTGCCGTTTAATCCTACCGATAAGCGTACGGCCTTAACGTATCTGGACAATCAGGGTAAAATGCATAGAGTCAGCAAAGGTGCCCCCGAGCAGATTTTGAATCTTGCGCATAACAAATCAGACATAGAACGCCGAGTTCATGCTGTCATTGATAAATTTGCAGACCGTGGGTTAAGGTCACTTGCTGTAGCATACCAGGAAGTTCCGGAGGGACGGAAAGAGAGCGCCGGAGGGCCGTGGCAGTTCATTGGGCTAATGCCGCTTTTTGATCCGCCACGTCATGATAGCGCTGAGACGATTCGAAGGGCTTTGAATCTTGGAGTAAACGTTAAAATGATTACAGGGGATCAACTGGCAATCGGGAAAGAAACCGGAAGACGTCTTGGAATGGGAACCAACATGTATCCTTCTTCGGCTTTACTCGGTCAAAACAAAGACGAGTCAATTGCCGCTTTACCCATCGACGAACTCATAGAGAAAGCCGACGGTTTTGCCGGTGTTTTTCCGGAACACAAATACGAAATAGTAAAACGTTTACAAGCTAGGAAACACATATGTGGCATGACTGGAGATGGAGTAAATGACGCTCCCGCCCTTAAAAAAGCCGATATCGGAATTGCGGTGGCTGATGCAACCGATGCAGCCCGTAGTGCTTCCGACATCGTCCTTACTGAGCCTGGGCTTAGCGTCATCATCAGTGCTGTTTTGACCAGTCGGGCCATCTTCCAAAGGATGAAAAATTACACTATTTATGCAGTTTCAATCACCATTCGTATAGTGCTCGGTTTTATGCTGCTGGCTTTGATATGGAAGTTTGACTTCCCGCCTTTCATGGTGCTTATCATCGCAATCCTTAACGACGGAACCATAATGACCATATCAAAGGATAGAGTGAAGCCATCTCCTCTTCCCGACAGCTGGAAACTGGCTGAGATCTTCACCACCGGGGTGGTTCTTGGTAGTTACCTAGCCATGATGACAGTTATATTTTTCTGGGCCGCTTATAAAACAGACTTCTTTCCGCGTACATTTGGTGTACCGACGCTTGAAAAAACAGCGCACGATGACTTCCGGAAGCTGGCGTCAGCGATATATCTCCAAGTCAGCACAATCAGCCAAGCTCTCATTTTCGTAACGAGATCTAGAAGCTGGTCATTCGTGGAACGTCCTGGTTGGCTGCTCGTTATTGCTTTCGCGATTGCCCAATTGGTTGCGACATTAATTGCGGTTTATGCCAACTGGAGCTTTGCTGCGGTAGAAGGAATCGGGTGGGGATGGGCCGGAGTCATCTGGCTCTACAATATCGTGTTCTATTTCCCGCTCGATATCATCAAGTTCTTTATCCGATACGCGCTTAGTGGGCGGGCCTGGGACCTTGTTATCGAGAGGAGGATTGCGTTCACGAGGCAAAAGGACTTTGGGAAGGAACAACGTGAGCTCCAATGGGCTCATGCGCAACGAACCCTTCACGGGCTTGAAGTTCCTGACACTAAAATGTTTGGTGACCGCACCAATGTCACGGAACTTAACCAGATGGCTGAAGAAGCCAAACGGAGGGCTGAAATTGCAAGGTTGAGAGAATTACACACGTTGAAGGGTCATGTTGAATCGGTGGTTAGACTGAAGGGTCTTGACATTGAGACGATTCAACAAGCGTACACCGTCTGA

# >HHA13

ATGGGGGAGGAGAAGCCTGAAGCTCTTGAAGCTGTTTTGAAGGAGACTGTAGATTTGGAAAGTATACCCATTGAAGAGGTTTTTGAGAATCTGAGATGTAGCAAAGATGGTCTCACCACTTCTGCTGCTGAACAAAGATTAATCATTTTTGGGCATAATAAACTTGAAGAAAAAAAGGAGAGCAAATTCCTGAAGTTTTTAGGGTTTATGTGGAATCCATTATCATGGGTTATGGAAGCTGCTGCTATCATGGCTATAGCCCTTGCTAATGGAGGAGGAAAGCCTCCTGATTGGCAAGATTTTGTGGGTATTATCACTTTGCTCATAATCAACTCCACCATTAGTTTCATTGAAGAAAACAATGCTGGTAATGCAGCAGCTGCTCTCATGGCTAGACTGGCCCCAAAAGCAAAGATCTTAAGAGATGGGAAATGGAATGAAGAAGATGCTTCCATGTTAGTCCCTGGAGATATAATTAGTATAAAGTTAGGTGATATTATTCCAGCCGATGCTCGTTTGCTCGATGGCGATCCGTTAAAGATTGACCAGTCGGCTTTAACCGGTGAGTCTCTCCCGGTCACAAAAGGTCCTGGTGACGGTGTCTATTCCGGTTCGACATGCAAACAGGGTGAAATCGAGGCGGTTGTGATAGCTACCGGGGTCCACACGTTCTTCGGGAAAGCTGCTCATCTTGTTGATAGCACAAACCAAGTTGGTCATTTTCAGAAAGTTTTGACTGCGATCGGGAATTTCTGTATTTGTTCTATTGCTGTTGGTATGGTGATTGAGATAATTGTGATGTTCCCGATTCAAGATAGGCAGTATCGCCCCGGGATCGATAATCTTCTTGTTCTTTTGATCGGAGGAATCCCGATCGCTATGCCGACCGTTCTTTCCGTAACGATGGCTATCGGGTCTCATCGATTGGCGCAACAGGGAGCGATTACGAAAAGAATGACAGCGATAGAAGAAATGGCAGGAATGGACGTTCTTTGCAGTGACAAAACCGGAACGTTGACTTTGAACAAACTTACAGTTGACAAGAATCTTATCGATGTATTTGCCAAAGGAGTAGACGCCGATACTGTGGTTCTGATGGCGGCCCGAGCCTCGAGAACCGAAAATCAAGATGCCATTGATGCTGCAATAGTCGGTATGCTGGCTGATCCAAAAGAGGCACGTGCTGATATTCAAGAGTTGCATTTTCTGCCATTTAATCCTACCGATAAGCGTACTGCATTAACGTATTTAGACAGTCAGGGTAAAATGCATAGAGTCAGCAAAGGCGCCCCCGAGCAGATCTTGAATCTTGCACACAACAAATCGGACATAGAACGCCGAGTTCATGCTGTCATCGACAAGTTTGCGGACCGTGGGTTAAGATCACTTGCTGTTGCATACCAGGAAGTTCCAGAGGGACGAAAAGAGAGTCCAGGAGGACCATGGCAGTTCATTGGGCTAATGCCGCTCTTTGATCCGCCACGTCATGATAGTGCCGAGACGATACGGAGGGCTTTGAATCTTGGAGTAAATGTTAAAATGATTACAGGGGATCAACTGGCAATCGGGAAAGAAACCGGAAGACGTCTTGGAATGGGAACCAACATGTACCCTTCTTCAGCTCTGCTCGGTCAAAATAAAGACGAGTCAATTGCTGCTTTACCCATCGACGAACTCATAGAAAAAGCCGACGGTTTCGCGGGTGTTTTCCCCGAACACAAATACGAAATTGTAAAACGTTTACAAGCTAGGAAACACATATGTGGAATGACCGGAGACGGAGTAAATGATGCTCCCGCCCTCAAGAAAGCCGATATCGGGATTGCAGTGGCTGATGCGACCGATGCAGCCCGTAGCGCTTCTGACATCGTCCTTACCGAGCCCGGGCTTAGTGTCATCATCAGTGCTGTTTTGACCAGCCGCGCCATCTTCCAAAGGATGAAAAATTACACGATTTATGCGGTTTCAATCACCATTCGTATTGTTCTCGGTTTCATGCTGCTGGCTTTGATATGGAAGTTTGACTTCCCGCCTTTCATGGTGCTTATTATCGCAATCCTTAATGACGGAACTATAATGACCATATCGAAGGATAGAGTAAAACCGTCTCCTCTTCCCGATAGCTGGAAACTGGCGGAGATTTTCACCACCGGTGTCGTTCTCGGTAGTTACTTAGCGATGATGACGGTTATATTTTTCTGGGCGGCTTATAAAACAGACTTCTTTCCACGTACATTTGGCGTTCCGACCCTTGAGAAGACGGCTCACGATGACTTCCGGAAGCTCGCTTCGGCAATATATCTCCAAGTCAGCACAATTAGTCAAGCTCTCATTTTCGTAACGAGATCTCGTAGCTGGTCGTTTGTGGAACGCCCGGGTTGGCTGCTTGTTATTGCTTTTGCTATTGCGCAATTGGTTGCTACATTGATTGCGGTTTATGCCAATTGGAGCTTTGCTGCCGTAGAAGGAATCGGGTGGGGATGGGCTGGCGTTATCTGGCTCTACAATATCGTCTTCTACTTCCCACTTGATATCATTAAGTTCTTTATCCGATATGCTCTTAGTGGGCGGGCCTGGGACCTCGTTATCGAGAGGAGGATTGCTTTCACAAGGCAAAAGGATTTTGGTAAGGAACAACGTGAGCTCCAATGGGCCCATGCGCAACGAACCCTTCACGGGCTTGAAGTTCCTGACACCAAAATGTTTGGTGATCGTACCAATGTCACTGAACTCAACCAAATGGCCGAAGAAGCCAAACGGAGAGCTGAAATCGCAAGGTTGAGAGAATTGCACACGTTGAAGGGTCATGTTGAATCGGTGGTTAGACTCAAGGGTCTTGACATAGAGACAATTCAACAAGCATACACCGTGTAA
