# Supplementary material for: Genome-Wide Identification and Analysis of P-Type Plasma Membrane H+-ATPase Sub-Gene Family in Sunflower and the Role of HHA4 and HHA11 in the Development of Salt Stress Resistance
Source: Genes (Basel). 2020 Mar 27;11(4):361. doi: 10.3390/genes11040361 (PMC7231311; doi:10.3390/genes11040361)
Supplement: Supplementary file 1 [file genes-11-00361-s001.zip › Supplementary File 3.docx]

Supplementary File 3: Genomic sequences of sunflower PM H^+^-ATPase genes.

# >HHA1

ATGGGCGGCGACAAGGCTCTCAGCCTCGAAGGGATTAAAAACGAGACCGTCGATCTGGTATAACAGACGTTTTTCTCATTTCACATTTATTTCAATATAGGTTCTAAATCTGTTATAAATTATAATTTATAAACATCATCATTATTAATTAATATTCAATAGCATATAATTTGCCCTGATGATCGGAACGAAACAATATATTAATTATTTTATTAAAAATAGCATGTTAATATTTTTTTTTTATAAATTATAAACATCATCATTATTAATATTCAATAGCATGTTGAGGAGCATTAGCATGTGAGAACTTTCCGTCACATACAACCAATTACAGATAGATTTTAAAATTTTATTTATTAATTTAATAATTTAATGGCAAAAGACAAATTAGCTGTTTCATGTGTGTGTGATTCACGGATTTGATATGACTGTCGATTTGAAAAATGAAAAAAATTATTTTGTTTCTTTATGTGTTTACTTTAGAATCATTTTAGTTCCGTACGTCTCGGTTGATGTAGATAATCTTATTAGTTAATTGATTTTGATGATGATTTGATTTTACCCCTAATCCGTTATTATCCCCCTAATTTTATGAATGATTTTGTTAAATGTTCGGTTAATGGTTATCTTTGGTCAAATGGTTGTAATTTTATAGCGGATTTGTTTGTTTTTTTTTAACATCTATAATTTTGGAATGGAATCTGATGTGAATGAACATATAAATGGAGTTTTGAAGTATGATCGTAAAGGTACATCGTTGATCGATGCAAACACATGAGATCCGTATGTATTTGTATCTTTTGCTTAAACTATGATACTTACCTATAACGGTTATTTAGCCAACGGTTATTTGACCAACGGTTATTTAAGCGCTTACCCCTTCCACTTATACCAATCTGGTACTTTGTAATGAAATTTAATGTGAATGAACATATAAATAAAGTTTTGGAAAAATGATCATAAAAGTACATGGTCGGCAAATTTAAAAACATGAGAACCGCGTGTACTTGTATCTTTTGTTTGAATTATGATAAATAAAACGCCTATTTAACCGCTTACTTCAATAATTTCTTGATTTTGAACAATTCCCGGTTATATCTAACAAATACTTTGTATAAATCTATTATGCGTTTTTTTAGACCTTAACAATAGTTTTGCTATTTATACTAATTTAATGGGTGTGGTTGTTTTATTATTTATTGTTTTTGCATTTAGCTTTTTTTAAATTACTATAAAACATCTTTAATTATATGTCTTGCTATAAGAAAATGATTTACATACCGTTAAATTTACTTTATAGAAAGATAAATATTATAATATTTAATAGCATTAAATCTGTTATTGAGGCATTATTAATTCGATTCAGAGAAAGATATTAAGAATAGTATTGATGCTTGTTCCGAACGTTGTGTACTCCGTGGTACCCGAAACTTGTCTATTTTGATGCTTTTAATTTGGTTGTTTGTTTCACTTTTGTTAGCAGCTCTGACTATTGGAATCTGACACAGACTTCTGAGTTTAAATTAATTTAATTTAATTCTATAATAAATTTTAAAACTTTACTTATATTATATTATATATTATAATAATATTAATAAAGAATGTTAACTAAATACCATAGTACCCAAAGGTGTCTCGTCTCTATGTAAGAGGTGGATTATCTAATTTTACAAGAAACGGTTAAATGAGTAATTAGGATTAGATAAGTTTATTGTTACTTTTGTAGGGAAGAAAATGAGTAAGTTCACTTAAATTAATTTCTTACAAAGAAAAAAAATAAAAATATCTTTTTAAAATAAATTGTATATTTTTATCATTATTAAATAAATAAATTTAATTATAACTAATAACTAATGCAATTATTTAGTCAAATAAATAAATAAATAAATTTAATTATAACTAATGGAATTATTTAGTCAAATAACTACTTTGTCATGGGAAATCGTTGATAGCACTTAATCTTTTTGGACTTTTTTATTTATTGGGCCAAGTGCCGTACTGGATTGGTTGATATTTTCAAAGAAACAATAAATAATTGAAAAGGATGCACCTTAATCTTGTGTGGTCCAACCCTTTTTTGTGTTAGTTAAAAAGTCAACTTGTGTTATTCATTATCATCATGTTCTATTGTTTATCCCCGTGAATTCTTTGTACCTAATCTTGACACGTATAATCTTTTATAAAAATTATAGTTAGCCATTGCCATTCCTAGTCCTTTTGTTTGTTAAAAAGTTCTTCTTCAATTAGTGTGTTGGTTAGTTATCATAATCATAATTCAATATCAAGAAAATGACTAAAGTAGCTTTTTTAGTGTGTATAGCACTATAGTGATATGAAATATGTGAATCTTCGATATGGTGTTGCGTATCACAAAGTATCATATTAGTTAAAGTCACTGCTGACGGAAAACAGAAAAAGTCTGATATCTAAGTAAAAGTAGAGATTTGACTCGTAGTTGCATATCTCATGATTAGATTAAATTAGTGATGTTTTTGAAGTTATAGAAGATCAATAAATATGATTATTTTCTTTAATTTCTCTTGCGTAATTTTAATCATCATCATCATACTCAATAAATCCCATCAATAGCAAAGCTAAGGTAGGGTATGAGGAGGGTAAGGTGTAGACAACCTTACCTCTACCCCGTAGGAATAGAGAGGCTGCTTCCAGTGAGACCCCCCGACTCGATAGTAGTTTTGCATCAAGCCTTGGACATAAGACACATAACACTCAGCAATTGGGACAAAAGCCGATTAGTGCATGTACCCCCTTGTTTTTCGGCTATCAACGCTACCACATGATGCATGATTAACCGTCCCTTGCTTTTTAACGTTATTTTCACGAAATTAGTAAAATAACGTTAAAAAACATTTTAATGATTTATTTAAAAAAACATTTCTTAAGTTGAAAAACAACCAAGTAACTCAATGGAGCAGTAACTAAAAATTTTGTTGAATAAAATTAGTTTTGTAGACATCTCACTTTTCTTTAACTGAAAAAAAAAAAAAAAGAATTAGTATCTTTTAAACAGTTATCATATTTACTTTGAAAGATATAAATTCAAGTATATGTGATCTAACATGTGTTTTTAGCTATTTCCAGTTAAAATTCATATCTTGTACATGCTTTTGTAATGTTTCTTTCACTCTGTTATTTTTCGATATGTTACTAATCCTATATTTGTTAAACACAGGAAAAAGTACCGATCGAAGAGGTGTTTGAACAGTTGAAATGTAACCGTGAAGGTCTTAGCTCCGACGAAGGAGCCCAACGTCTTGAAATTTTCGGACCCAACAAATTGGAGGAGAAAAAGGTATTACTTCATTACTTGATTATTACCATTCTATTGTCACATTTTCACTTATTTAACTTAAACTTGCGGCGTAGCTTGTTGCCCGTTTACCTGTCATTCTTATGTAATTTTTTTTAACTATACGACAAGAAACTTAAACTTTTTTTCATCAAATACAGGAAAGCAAAATCCTCAAGTTTCTTGGGTTTATGTGGAACCCCCTATCATGGGTCATGGAGGCTGCAGCCATCATGGCGATTGCACTCGCTAACGGCGGTGGTAAGCCGCCAGATTGGCAAGATTTCGTTGGTATCGTTTGCCTGCTTGTTATCAACTCCACCATCAGTTTCATCGAAGAAAACAACGCTGGAAACGCTGCCGCTGCACTTATGGCCGGTCTTGCACCTAAAACCAAGGTACATAACAATTTTCTACGCTACATCTCGCATATCAAGGACTGATATTTTGACCAGGGGACCAATAACCAATATATCGCCAATGTTAACTCATAATTTGTATGATTGTATTCAGCTTTTGAGGGATGGCCGATGGAGCGAACAAGAAGCTGCTATACTGGTTCCCGGAGATATCATCAGTATCAAACTTGGTGATATCGTTCCTGCTGATGCGCGTCTTCTTGAAGGTGATCCCTTAAAGATCGACCAATCTGCGCTTACTGGAGAATCTCTCCCGGTCACCAAGAACCCATACGATGAAGTGTTCTCCGGTTCAACTTGCAAGCAAGGTGAACTTGAAGCCGTTGTCATCGCTACTGGCGTCCACACCTTTTTCGGAAAGGCTGCACATCTTGTGGACAGCACAAACCAAGTGGGACACTTCCAAAAGGTTCTAACCGCAATCGGAAACTTCTGTATCTGCTCCATTGCTGTCGGAATGGCTGTTGAGATTATCGTTATGTACCCGATCCAACATAGAGAGTACCGATCCGGGATTGACAATCTTCTGGTGTTGCTTATTGGTGGAATCCCGATCGCTATGCCTACTGTTCTTTCGGTCACTATGGCTATCGGCTCACACAGGCTTTCGCAGCAGGGTGCGATCACTAAACGGATGACTGCTATTGAAGAAATGGCGGGAATGGATGTTCTTTGCAGTGATAAAACCGGAACTTTGACGCTAAACAAGCTTACGGTTGATAAGAATTTGATCGAGGTGTTTGGTAAAGGCTTGGATAAGGAGCAAGTGTTGCTTTACGCTGCTCGCGCTTCTCGAATGGAAAACCAAGACGCTATCGATGCAGCCATCGTCGGAACGCTTGCTGACCCCAAAGAGGTATATATGTATAAAACGATCATTACAGAACACTAATTTCGTGTTCTTGCAAAATTTCGTGTTCTGCATAGAACCTTCCCCAATATTTATATTTTTTTGGCGTTTTGTAAATAAAAATGAAAACGTCTTATTCGTTTTGCGTGTTTTCATAGGCACGAGCTGGCATTAGAGAGGTCCATTTCTTCCCGTTCAACCCTGTCGACAAGAGGACTGCGTTAACGTACATTGATAATAACGGCAACTGGTTTAGAGCTAGCAAGGGTGCGCCTGAACAGATATTGACACTTTGCGGGTGCAGGGAAGATCTCAAGAAGAAAGTTCACGCGATGATTGATAAATTCGCCGAACGTGGGTTGAGATCTTTGGGTGTTGCAAGACAGGTTTGCATCAATCTATTTTATATGCATGACAGTATGAGTTACATTTTTTTCTGTTTTTTACATGATCAATGTGTTTTTTTTTTTTTTTTTTTTTTTTTTTTTTGCAGGAAGTGCCTCAAAAATCGAAAGATAGCCCGGGTGGTCCATGGGAATTCGTTGGATTGTTGTCTCTCTTTGATCCACCAAGGCATGACAGTGCCGAGACCATTAGAAGAGCTCTCAATCTCGGTGTAAACGTCAAGATGATTACTGGTAACTATATCTCCATATAAACGACCCAGTCATGATTACGACTCGTTTAACTACTTTCTAGGGGGGGGGGGGGGGGGTTTGGAGCACGGACCTCCCGGCCGCTGGAGTGATCCCACTCCACAAGCTAGCTTAGCCCCATAGCTGCCTGGCGGTTAATACCCACCCGAGCCGAACCTTAGGGCGATTTACAGCTTGCCAAGATTCGAACCCAGGACCTCATGTTTATAAAACAGTTTTATGTTTTATGTACAAAGATGAATAAATGATAGATCCTAACATTGTAATTTTAATTATTTTAAAAATTAAAAAAGTCAAAGGGTGTATTTTTCAGTCAAACAGGTCTAATCGTGTCTTAACAGGTACCCAATTGCCAGCCCTGAGTCATCTAACTACGACATCTGTAAAAAACAAATTTTAAAAAATAGCCTAGCGTTCATTTACGCCTGGTTTTTGATAGCTTACACTGCTGTAAAAAAATTATAAAAACATATGACGTCACCCTTTTTGGCGGGTTACACTTATGCATTTTGAAAAGTTACATAATTTTAAATTTTTTGGCGAATTTTTCAAATAAAACCTTTTTTTTTTCAAAAAGTTTTTTCGGTTCTCGCAACTCGGGGGGGGGGGGGGGGGTGTTCTCTTTTAACCTCATGGTTTTAATATTTATCGTATAGGTGACCAACTTGCTATTGCTAAGGAAACTGGAAGACGTCTTGGTATGGGAACAAACATGTACCCTTCGTCGTCTTTACTTGGCGGACACAAGGACGAATCAATTGCTGGACTTCCAGTAGACGAGTTGATCGAGAAAGCTGATGGATTCGCCGGAGTTTTCCCTGGTGAGTGTTTCTTCATACAAGTTCTCGACGTTATTTAAACTGCTATACTTTGACACGTCATTATCAACAGAGCACAAGTATGAAATTGTGAAGAAACTACAAGAGAGGAAGCACATTTGTGGTATGACCGGAGATGGTGTGAACGACGCGCCTGCGTTAAAGAAGGCGGATATTGGTATTGCGGTTGCTGATGCTACCGACGCTGCAAGAAGTGCTTCCGACATTGTGCTTACCGAGCCTGGGCTCAGTGTGATCATCAGTGCTGTGCTTACCAGTAGAGCTATTTTCCAAAGAATGAAGAATTACACCGTTAGTCCCTTTCGTCGTCTTATCTAATAAACACATTTCTTTTGTTGATCATGAGAACAACGACCAGTGGCGGAACCAGAACGATTTAGTTAGGGGGCGGGGGGGCATCATAAGCTTATATTCTATACTGGTTTAAATTAGGGGGTCAATCTCTAAGTTAATTTGTTCTTCAAAAACTCAAAATTTATAAATAAAAATTCAAAAAGTTCCAGTGACCTGAGGGTCAACGGACCCTCTTGACCCCCCTCTGGTTCCGCCCCTGATAGCGACGGACACGCCAAAATGCACTAACAGACCTTTGTTATAAACACACTTTCTTGGTCAAAACTTACTTAAAACCGTTCATCTTATTCGCAGATCTACGCCGTTTCAATCACCATTCGTATAGTGTTTGGATTTATGTTCATCGCGTTGATATGGAAGTTTGACTTCTCGCCTTTCATGGTTCTTATCATCGCAATCCTCAACGACGGTAAGTTTTCTAACAACCGTAGTTCTCGTTTATGATATGTAACATTCGCTGACACCCGTTATGTTTCAAAGGTACTATCATGACAATCTCGAAGGATAGAGTGAAACCATCTCCGTTACCCGACAGCTGGAAGCTAAAAGAGATCTTCGCCACTGGAGTTGCTCTCGGAGGTTACTTAGCTTTAATGACAGTTATCTTCTTCTGGATCATGAAGGATACCGACTTTTTCTCAGTAAGATATAAATTAATAGGAGTATTAATCATGTCGGGTTGACCTGTTTGATAAAACGGGTTGAAAGTTCAATCTATTTAGTTAAACGGGTTAAACAGGTCAACCCAAACACGAGTTGTTTATTTAACGTTTTATATGACAACCCAAAACCTGGTTACTTTCATGTCGTGTCGTGTCATAACTCTCTATTTATGTTTCCTTCGTCTCCGCAGGATAAATTTGGTGTGAAATCTTTAAGAACCAGTGAAACCGAGATGATGGCCGCTTTATATCTACAAGTCAGTATCGTAAGCCAAGCTCTCATCTTCGTCACCCGTTCTCGTAGCTGGTCGTTCGTCGAACGACCTGGCTTCTTACTGATGGGCGCTTTCCTAGCAGCACAACTGGTAACCTTTTTCACAAAAAAAAAAAAAAAAAAAAAAAAACCAACTTAAATTCTCAAAACAATCACAAAACATGCGACTCGCTTGCAGGTAGCAACTGTAATTGCGGTATACGCAGAGTGGGAATTCGCGAGAATTAAAGGAATCGGATGGAAATGGGCTGGTGTTATCTGGCTTTACAGTATAGTGTTTTACTTCCCTCTTGATATTATGAAGTTTGCCATTAGATACATCCTTAGTGGCAAGGCTTGGCTCAGCATGATTGACCAAAGGGTATGTTACTATATTTCTATACTAGGTTATAACCCCGTATATTACACGGGTTGAATAAATGAATTTTATATATCAAGCAAGGGTGAGTTATTTGTAAATATAGTAGATTGAGAGAGTAACGTTAGCCATTCAAAAAAAAAAAAAAAAAACCTAATAATAAAACATTATCTTTTTAAAAGCCTCCTTTATTGCACAGGTTTAAAAAATGTAATTTTATATATTAAATAATAAAATAAGTTATATCTATAAGAACCATATGTATTGTATAGGTTGAATAAATGTAATATTATATACCAAGTAGTAAAAAAAGTTATATCTTTAAAACCATTGGATTAAACGGGTTGAATAAATATAACATTGTTTACCAAATAATAAAGAAAGTTATGTTTAAAAACCCCTGTGTATTACATGGGTTGAATAAATATGATTTTAAATACCAAATAATAAAAAAAACTAACAGTCTTTTTTATATTTAAAGTGGGATAAGATTGAATATTAATCTGAACTAACGGATTTTTTTATATTTAAAGTATGATAAGATTGAATATTAATCTTTATTTATTTAGTTAATATAATATTGAAAAATCATATGATTCAATAAGTGGGAGGTTGTATCATGATAAATCGGTTAACCGTACTGAATGATAAAGATAATAGTGATTGTTGAACAAATTAATTAATTGAATTACCAGAAACATTTGTGATGTTAGGCGGATTTTTTTAATTATTTGAAAGTTAATATCAACTTTGGAATTCGTATGAATTCTTATTAGATTTGATTAAATATTATTGATAACAAAAATTTGTATTATATTAGATTTTAGATTTGATTAAATATTATTAATAATAAGAATTTGTATTAATTTAGATTAAATATTATTATTTTTAGTATTATTAATAATTTTAATCAATTAAATGAGAGAATGACAAGTGTTCCAAAATAGGTTTCTTTTATTATATACTATAGATATCTATATTTCAGTATTTTGTAAATAACCTTTTATCATAAACTTTAGTGAATGCTAAAACTTATAAAACATTGTGTGTGTTTTTGTGTAGACTGCTTTCACAACAAAGAAGGATTATGGTAGAGGAGAGAGAGAGGCCCAATGGGCTCATGCTCAAAGAACTTTACATGGGCTTCAAGCACCTGACACATCAAATCTCTTTAATGAGAAGAGCAGCTATAGAGAACTGTCGGAAATCGCTGAACAAGCCAAACGACGCGCTGAAGTTGCAAGGTACTTTAACCGTTAACCGTTAACAGTTAACAGTTAACTAATATTTAACATTTTAACTAACACTTGGGTATGTGTGTGTATATATATAGGCTTCGGGAGGTGCTTACGCTCAAGGGTCACGTTGAGTCAGTGGTGAAACTGAAGGGGCTCGACATTGATACAATTCAACAGCATTATACAGTATGA

# >HHA2

ATGAGACCGTCGATCTGTTCCGAAAAAGTACCAATTGAAGAGGTGTTCGAGCAGTTGAAATGTAACCGAGAAGGTCTGAGCTCCGATGAAGGAGCCCAAAGGCTCGAAATTTTCGGACCCAACAAACTAGAAGAGAAGAAGGTATTCCACAATTATCACAGTACTTGTAACCTGCCACGAATAGCGTCATTTCCAAACGGTTAAAACTAACGTATATCTTTTTTTTTTCACCGAATACAGGAAAGCAAATTCCTCAAGTTTCTCGGGTTTATGTGGAACCCTCTATCATGGGTCATGGAGGCTGCCGCCATAATGGCCATTGCACTCGCTAACGGTGGTGGTAAGCCGCCAGATTGGCAAGATTTCGTCGGTATTGTTTGCCTGCTTGTTATCAACTCCACCATCAGTTTCATCGAAGAAAACAACGCTGGAAACGCTGCTGCTGCACTTATGGCCGGTCTCGCACCTAAAACCAAGGTATTTTACTATTTCCTAAACTAAAATTTTAAACGCCAACAGTGTTGTAAGAATCGCTTGGCGCCAGTCGATCGGTGGGGTACCGACTAACGATTAATTTAATTGAGAATTTATATGTAACTTTGAGTTTTATAAGTAAACACACATTTTATATGTATTTTTTAAGTAGACATGTTTAGCAGCTTCTTCGACCCATATTTTCAAGTAAACAGGATTAATTGCTGAATTTACACGTTTTAGTTAAGAATCTGGCCGGAATCGCTAGACTGCCACCGATATTCAGCCAATTAGTGTTTGATCACTTTTTGACCGCCATAAATTGACATATTAGATAGAAACTGCTCGGTGAGCCTCCAGCTAATCAGAGGCTAGTCCAGATTTTTACAACCATCGTAGTAATAAACGCTCTATAATCTTTCTTTAGGTTCTAAGGGACGGACGCTGGAGCGAACAAGAAGCTTCTATACTGGTTCCAGGAGATATCATAAGCATCAAACTCGGTGATATTGTTCCGGCTGATGCTCGTCTACTAGAAGGCGATCCATTAAAAATCGATCAATCCGCCCTTACCGGAGAATCTCTCCCGGTAACCAAAAACCCATACGACGAAGTATTCTCCGGTTCAACTTGCAAACAAGGCGAACTGGAAGCCGTTGTGATCGCCACAGGAGTCCACACCTTTTTCGGCAAGGCTGCGCATCTAGTCGACAGCACAAACCAAGTGGGACACTTCCAAAAGGTTCTCACCGCTATCGGAAACTTCTGCATTTGCTCCATTGCTGTAGGAATGGTGGTAGAGATCATTGTCATGTACCCGATCCAACACCGAGAGTACCGAAAAGGGATTGATAATCTGTTAGTGTTGCTTATTGGTGGTATTCCGATTGCTATGCCTACTGTTCTTTCGGTTACTATGGCTATTGGATCGCATAGGCTTTCACAACAAGGTGCTATTACTAAAAGAATGACTGCTATTGAAGAAATGGCGGGAATGGATGTGCTTTGTAGTGATAAAACTGGGACTTTGACGCTTAATAAGCTTACGGTTGATAAGAATTTGATTGAGGTGTTTGGTAAAGGTTTGGATAAGGAACAGGTTTTGCTGTATGCTGCTAGAGCTTCTAGGATGGAAAACCAAGATGCTATTGATGCTGCTATTGTTGGAACCCTTGCTGATCCCAAAGAGGTATTTTATATTATTTTTTAATTTCTTTGAAGTTAGAAAAACGGTTATTGTTGTTAGGGAAAATCACCAAAATAACCTTTGAGGAATTTCACTTTACGAAAATGAACATGGGTTATGTTTGGTGTAGACGTAATCTTCGATCATTGCGTCTGGCAAAGACTCGTACTCATTAGCTAATAGAAACTTGACACGTGTCATCATCTTTAGTTTTTTTTTTTAAACTTTTCCACCACGGTTTTTATACATTAATATTTTTTTGAAAAGTTTACACCAAATTAAAGAGAATTTTATTATCTTTAATTTGCTGTATTTTTTATAAAAAAATACCAGCGTATGGAAAAGTTTTTGCTCGTTTAAAAATCGTGGTCGCAATCCTCGATCTTTGCGTCTTGTATAGACTCATACTTTTCAACCAATAGAAACTTGACACGTGTCATTATCTTTAGTTTTTTTTTTTCAACTTTTTCACTATTATTTTTATACGTTAACACTTTTTGAAAAGTTTACACCACATTAAAGAGAATTTTATTATCTTTAATTTGGTGTATTTTTTATAAAAAAAATACTAACGTATGAAAAGTTTTGCTCGTTTAAAAATCGTGGCGGAAAAGAAAAAAGTTGGCGTTGAAACCCACGACAGTTTGAGTCTCAGACGCAAAAGTTGCATTTATTCCCGTGATTACGTGTGTTTATTTTCGTAAAACGGGTTGGTCAAAGGTTATGTTGGTAATTTTCTCTTATTATTATTTTGATTTGATTATTATTGACCTGTATGTTTTTTTGTAGGCACGAGCTGGCATTAGAGAGGTCCATTTCTTCCCATTCAACCCGGTGGATAAGAGGACTGCTTTGACATACATTGATAATAATGGCAACTGGTTTAGAGCTAGCAAAGGTGCACCTGAACAGGTAATGTATTCACCTACATTCTACTTGCTATAGCAAAATGAATTAAAAAAAAAAAAAAAAAAACTCAATGTTCTTAATTGTTGGATCAGATTTTGACCCTTTGCGGGTGCAAAGAAGACCTGAAGAAGAAAGTTCACGCAATGATTGATAAATTCGCTGAACGTGGGCTGCGATCTTTGGGTGTTGCGAGACAGGAAGTGCCGCAAAAATCGAAAGATAGCGCAGGCGGTCCGTGGGAGTTTGTTGGGTTGTTGTCTCTCTTTGATCCACCAAGGCATGACAGTGCCGAGACCATCCGAAGAGCTCTCAACCTTGGTGTTAATGTCAAGATGATTACTGGTAACTATATCTCCATTTAACCAAATTTTACACAAACGTAGATAAACATCTAACAAAGACATCTGTGAAAAAAAAAATAAAAAAAAATTGTCGAGCTGGCATTTATTCAACGTGTAAGTTTGTCTTACATTGACGTAAAAATGATGAATGTTACGGCTGTGTAAATTTGTTTTACATTGGTGTAAGATTTCGTATACGATATTTTTTATTGAAAAAGTAATATAATTTAAAAAATTTTTGTCAATTTTTTTTTCTTAAAAAAACCTATTTTTGAAAAAGTTCTTGTGGTTCTTGCAACTCGGGGAATTCTCATTTTAACCTTTCCCTGTATATATACATATACACCTAAATTATATAAATTAATAGTGGTTTAATATCATATAGGTGATCAACTTGCTATCGCTAAGGAAACCGGTAGAAGGCTTGGTATGGGAGTTAACATGTATCCTTCGTCGTCTTTACTTGGCGGACACAAGGACGAATCAATTGCCGGACTTCCAGTAGACGAGTTGATCGAGAAAGCTGATGGATTTGCTGGAGTTTTCCCTGGTGAATGTTTCTTCATTTAAATTCTCGACACAGTTTAAATTACTATACTTTGACACGTCATTAATTATCAACAGAGCACAAGTATGAAATTGTGAAGAAATTACAAGAGAGGAAGCACATTTGTGGTATGACTGGAGATGGTGTGAACGACGCACCTGCTTTAAAGAAGGCTGATATTGGAATTGCGGTTGCTGATGCAACCGACGCTGCAAGAAGTGCTTCTGATATCGTGCTTACTGAGCCTGGGCTTAGTGTCATCATCAGTGCTGTGCTTACGAGTCGAGCTATTTTCCAAAGAATGAAGAATTACACCGTTAGTCCCTTTCGTCATCTTATCTAATAAACACATTTATTTTATTAATATATTAAACCATTCATCTTGTTTGCAGATCTATGCGGTTTCAATCACCATTCGTATCGTGTTTGGATTTATGTTTATCGCGTTGATATGGAAGTTTGACTTCTCTCCCTTTATGGTGCTTATTATTGCAATTCTTAACGACGGTAAGTATTCTAACAACTGTAGTTCTCGTTTACATACGTAACATTTTCTAACACATACCCTTAATGTATTAAAGGTACTATCATGACAATCTCCAAGGATAGGGTGAAACCATCTCCGTTACCAGACAGCTGGAAGCTAAAAGAGATCTTTGCCACCGGAGTTGCTCTTGGAGGTTACTTAGCTTTAATGACAGTTATCTTCTTCTATATCATGAGGGATACCGACTTTTTCTCGGTAAGATATAAAATTATTAGGATCGTTAACAGTGTAGTCAAGTTGTAAACGTGTGGTAAACAGGTTGGCGTGTTGACCTGTTTATGCGACAGGTTGACAGGACAACCTGTTTTATTAAACGGGTCAGACATGACAATCCAAAACTTGATTATTTTCATGTCGTGTCATAACTCCGTATTTTTGTTTCCTTCATCTCCGCAGGATAAATTTAGCGTGAAATCTCTTCGGACCAGTGAAACCGAGATGATGGCTGCTTTATATTTGCAAGTCAGTATTGTGAGTCAAGCTCTCATCTTCGTCACGCGTTCACGTAGCTGGTCGTTCGTTGAACGACCCGGCTTCTTACTGATGGGCGCTTTCCTTGCAGCACAACTGGTAATCTTTTTTCTACAAAAAGTTTCCAAACTTTTTTAATTCTCAAAACAACCACATAACATAAAAGATTAAAACCCGTATTTGCATAAATTTCAGGTAGCAACTTTAATCGCGGTATACGCAGAGTGGGAATTCGCAAGAATCAAAGGAATCGGATGGAAATGGGCTGGCGTTATCTGGCTTTACAGCATCGTGTTTTATTTCCCGCTTGATATCATGAAGTTTGCTATCAGATACATCCTTAGTGGCAAGGCTTGGAACAGCATGATTGACCAAAGGGTATATTACTTACTATATTTAAATAATTTCATCATTCATCAATAACCTTATATCCTAAACTTTAATGATGAATACTAAAAGTTAACAAAACATTGTGTGTGTTTTTGTGTAGACTGCTTTCACAACAAAGAAGGATTATGGAAGAGAAGAAAGAGAGGCCCAATGGGCTCATGCTCAAAGGACTCTTCACGGGCTTCAAGCACCCGATACCTCAACCCTCTTCAACGAAAAGAGCAGCTACAGAGAATTGTCTGAAATCGCTGAACAGGCCAAACGACGTGCTGAAGTTGCAAGGTACTTTAACTGTTAACCGTCGACTAAAACCAACTTTTCAGATTTAAAGTCGATAAGATTTAACTAACACTTGGATATGTGTATATATATAGGCTTCGAGAGGTGCTTACGCTCAAGGGTCATGTTGAGTCAGTGGTGAAACTGAAGGGGCTCGACATTGATACTATTCAACAGCATTATACAGTATGA

# >HHA3

TCACACTGTGTAATGCTGTTGGATCGTGTCGATATCTAGCCCCTTCAGCTTCACCACCGACTCAACATGCCCCTTCAGTGTAAGCACCTCCCGAAGCCTGTATATACATCACCAAAATTTATAAAACTTTCCATTTTTAAAAAGAAAATTAAAAACTAGTTTGAAATACGGATAACAACATACCTTGCAACTTCAGCGCGCCTCTTAGCCTGTTCCGCTATCTCAGACAGCTCCCTGTAGCTGCTCTTTTCGTTAAAGATGTTGGAGGTTTCGGGTTGTTGGAGCCCATGCAATGTCCTTTGAGCAAGAGCCCATTGGGCCTCTCTCTCTTCTCTTCCATAATCCTTCTTGCTTGTGAAAGCAGTCTTGTTTTCAAGCAAGTTGTTCCATGCTTTCCCGCTAAGGATATATCGGATGGCGAATTTCATAAGATCGAGCGGGAAGTAGAACACGATGCTGTAGAGCCAGATGACACCAGCCCATCCCCAGCCGACTCCCTTGATTCTTGCAAATTCCCAATTTGCATACACTGCGATTAACGTTGCCACCTGAAAGTTTCGTATTTATAACAGCTGTTAGTTAGTTTGTTGGAAAACGATATTCGGTTAGTATAGATAAACGAAAAGAAGCAGGTTTTTTACCAGTTGTGCTGCTAAGAAGGCACCCAAAAGTAAGAGACCGGGACGTTCGATAAACGACCAACTGCGAGAACGCGTCACAAAAATCAAAGCCTGGCTGACTATACTGACTTGAAGATATAAAGCAGCCATCATCTCAACTTCACTGTGTCTCAAAGATTTAACGCCAAACGTGTCCTGTACGGTAATAAGAAAACAAGAGTTAGCTTTGTCATTTGTGTGCTGTTGTGTCGTACTGTCGTGTCTCTAATTCGTTCGTTTTTTCACGTACCGAGAAGAAGTTTGTGTCTTTCATGATCCAGAAGAATATGACAGTCATCAATGCAAGGTAACCTCCGAGCACAATGCCGGTGGCGAAGATCTCTTTTAGCTTCCAGCTGTCGGGTAATGGCGATGGTTTCACCCTATCCTTTGAGATTGTCATGATTGTGCCTTTGAAAATTTAACGAAACTTGAAATTAGTATGAAGACGGTTAAAACGTTGGTGTTTTGGAGTGTGGTAGAAGTGAAAGCTAACCGTCGTTAAGGATTGCGATGATCAAAACCATGAAGGGAGAGAAGTCGAACTTCCATATCAAAGCAATGAACAGAAATCCAAACTACAAAACGTTTCAAGCAAGTTAGCCGGTATACTCATTGTAACCACAAGTATAATGATTTTAATGTCACAAACAACTTACCACAATACGAATGGTGATAGAAACAGCGTAGATCTGGGAAAAGGAAAAAAACATGTTTAATTAAAAAACAGATTTCGTGAAAATAAATATGATAATTTGAGAAAAAGAGATAAACTAACGGTGTAGTTCTTCATTCTTTGGAAAATAGCGCGACTGGTAAGCACTGCACTGATAATGACACTAAGCCCGGGCTCAGTAAGCACGATGTCAGAAGCACCCCTTGCGGCATCTGTAGCATCGGCAACAGCGATACCAATATCGGCCTTCTTCAAAGCAGGCGCATCATTCACACCATCACCAGTCATTCCACAAATGTGCTTTCTTTCTTGTAACTTCTTCACAATCTCATACTTGTGTTCTGCATAATCAGATTCAGTGTGAGGAAACTTGAAAAGAATATTATTGTTACGATAATACCAACCACTAACCTGGGAAAACTCCGGCAAATCCATCGGCTTTCTCGATCAACTCTTCAATAGGGATAGCGGCTATCGATGCATCCTTGTGGTTACCGAGTAAAGAAGAAGACGGGTACATGTTTGTTCCCATACCGAGTCGTCTACCAGTCTCTTTAGCAATAGCAAGTTGATCACCTATATATAACGATTAAAGATATGACTTAGTTACAAAAAAAATAAAAAAATAATTGCTTTCGTAACTCAGTTATCAGTAAGTATGATTAATTACCAGTAATCATCTTAACATTGACACCGAGATTGAGAGCTCTGCGAATGGTCTCAGCACTGTCATGCCTTGGTGGGTCAAACAACGACAACAATCCGATAAACGTCCATGGACCACCAGGGCTTTCTTTGTTTTTTTCAGGTACTTCCTACAAATTCATCACGATAACAAGAGTAATAAGTAATCATGAGCAAACATAACAACATATTAGTGTGTGGATAAAAACGATAATAAAACGCAGACCTGTTTCGCAACAGCCAAAGAACGCAACCCACGTTCAGCAAATTTATCAATCATTGCATGAACTTTCTTTTTCATATCTTCTTTGCATCCACAGAGGGTCAAAATCTAATCAAAATTTCAGAATGAGAAACATGAATTTAGTAACCAAGTGATACATAAGGCACGAGAGTATACAACTATAGATAGTAAAAGTGAAACATTTACCTGTTCGGGAGCACCTTTGCTTGTTCTATGCCAGTTACCGCGCTCATCGATGTATGTCAAAGCTGTCCGCTTGTCAACAGGATTAAACGGAAAGAAATGGACCTCCCTAATTCCAGCTCGTGCCTACATAATCATTCATATATGTTTGATAAAGTTAAATTAATTTGATAACGATCTAATTTTTGTACAAAATGATATATAGATGGTAATAAAAATTACCTCTTTCGGGTCAGCAAGGGTTCCAACAATGGCCGCATCAATAGCGTCCTGGTTTTCGGTCCTAGAAGCCCGAGCAGCATAAAGCAGAACCTGATCCTTATCGACACCTTTAGCAAAAACCTCAATCAAATTTTTATCAACACTAAGCTTGTTAAGAGTCAGTGTTCCGGTTTTATCACTGCAAAGTACGTCCATTCCCGCCATTTCTTCAATGGCAGTCATTCTTTTAGTAATAGCTCCTTGTTGTGAAAGCCTATGCGATCCAATAGCCATGGTGACAGAAAGAACCGTCGGCATAGCGATTGGGATTCCACCGATAAGCAAGACCAGAAGATTGTCGATTCCATTTCTGTACTCCCTGTGCTGAATCGGATACATGACGACAATCTCAACCAACATTCCGACAGCGATTGAGCAGATACAGAAGTTTCCGATAGCTGTTAGAACTTGTTGGAAGTGACCGACTTGGTTAGTGCTATCCACTAAGTGTGCGGCTTTCCCGAAGAAAGTGTGCACACCAGTTGCGATAACGACGGCTTCTATTTCACCTTGCTTGCAAGTTGAACCGGAAAACACCTCGTCGTAAGGGTTCTTGTTCACAGGAAGCGATTCACCAGTAAGGGCAGATTGGTCAATCTTTAAGGGGTCACCTTCTAGGAGACGTGCGTCAGCAGGAACGATATCACCAAGTTTGATACTGATAATATCTCCTGGAACCAAGATTGCAGCTTCTTGTTCACTCCAATTACCATCCCTCAGAACCTGAGGTAAGAACAGATTAGTCAGTATTTAAACTCGATTATCGATTCTACATTTTTTAACTAATAAAATGAATACCTTGGTTTTAGGGGCGAGACCAGCCATAAGTGCAGCAGCAGCATTTCCAGCATTGTTTTCTTCGATAAAACTGATGGTTGAGTTGATAACAAGAAGGCAAACAATACCGACAAAGTCTTGCCAGTCGGGTGGTTTCCCGCCTCCATTAGCCAGTGCAATTGCCATAATGGCTGCAGCTTCCATGACCCATGATAGAGGATTCCACATGAACCCAAGAAACTTGAGTAGTTTGCTTTCCTATGTTCATTTAAAAGGTAATAATACATTAAGCACTTGAAAGGTGAAAAGAAACAAAATAAGTATGATCATGACTATTATTATGAATATGATTGATCATATACCTTTTTCTCCTCCAATTTGTTAGGACCAAAAATTTGAAGTCTTGCAGCCCCTTCATCGGAACTTAGACCTTCTCGGTTACATTTCAACTGTTCAAACACCTCTTCAATGGGTACTTTCTCCTGTGATTAGATATAAACTTATGAGTTAAACATCAAGGGACAGTTATTATTCGGTTAGCTACATGTGTCAGCTAACACTTAAGATGTTAGTCATTATTATACTTGTAACGCATGCACTAAACATGGTATTTACAATGGGATTGGTCATTATAGCACTAAAAGAAAAGGTTTACATAAATAACATGCCTTGGATAATTAGGTTAGATTCGACTGTAAATCTTGTATTTTAATTAAAAAGAGTAAGGAAAATAATTCAGTTATAGTAAACAATAATTGATTACAAAGTAAATAAGATATTAGTCACAATGATGCTTTAGATTATCTTAATGTTTATCTTTACTTTTTATATTCCACAATTTCATGTAAATGGTATCAGGTTCCAATGATTATGACCCAACACATAAAAATTAGGCAAGAGAATCAAAGTCCACTAAAAATGAGAACAATTCATTTTTAAATAATTAGGAATTTAATAATAAATTAAAGAGTAAATTACGATTTTGACCCCTGTGGTTCTATCACTTTTACTTTTTTAGTTCAAATTGTTTTTTAACATCTGAGCTCCCAACGTCTTTTTTTCTAACCCTTTTGGCCCCTAACACTAACCCCATCCATTAAATGTTAGGGGCCAAAAGGGTTAGAAAAAAAGACGTTAGGGGCCAAAAGGGTTAGAAAAAAAGACGTTAGGGGCCAAAAGGGTTAGAAAAAAAGACGTTGAGGGCTCAGATGTTAAAAAATTCTTTTTTGGACTAAAAGGGTAAAAGTGATATAACCACATGGGCCAAAATCGTAATTTACTCTAAATTAAATAAACGAATAAAACGCTATGAAAAACCACTCTATTTACCAAAGGCACACACATATATTATATTAAAACCACTCTATTTACCAATTCACACAACTTTAACACACACTTTAGAATGGTTTAATGGAGAGATGTTAACATATATATATATAACACAAAAAATGAGTGAAAGCTTTTAATTAAGTTAAAGTGTACTAACAATCTAAAATAGCATAAATGGTAATTTTCTTGATCAAGATGCTACCTTTTTTTATCTTGTTGGGTCAAAATGATCATTTAGTGATTTTCCCAGAAACATATTCACATGGGGGTAGATAAATTAAAAGAAAAACAACACAAACAAAGATATGGATGGTTGAGTAACGCAAGTTGACTTTCCACAAACTCAATATGGAGGGACCCAAAACTGGTGGGTGTTAGTGTGTGAAGCACCGAATACCAAAAAGTCCCTAAAAGCCAACCCAGTCACACAAAAAAGCAAAGGGTATTTCAGGAATTCCGTGCCAAAAAAAGTGGGTGGGGCAAAAGAAAAGGATCCATATTTGCATATAGAATATAAAATAACAACTAATTTGCAACTAAAATAAAAGGCAAGATAAAAAGATTCTTTATATATTCGAAACACTTGACAGCTTATAATCTATTAATATTTTGTAATATGTAAATACAAATTTTCATTAAAAAATAGAGTAAATTACTTTTGAGTCCTTAGAGCATTCACATTCTATCCACCAAATCTTGAGAGATGGGTTTTTAAATTATAAAAAGTGGTTTAAGTGGTTGTGAGTGGATGAGAGAGAAAATGTTACTGTTCATCTGTATATTTGGGGGGACACTGTTCACCTTGTATAATTTTTTAATATATTTTGAAAGTGGTTGTGAGTAGAGGAGAGAGAAAATGTAATATATATTGAAAAAGAGAGAGAAAAAGTATTTGTTTTTAGTGGAAATATATTGATATAGGAGTTGTTTTTTAGTGGAATGTATGTATATTTTTAGTGGATTGGATGTGAATGCTCTTATGTTTTAGTCGTTTTAACCATTTGAGACTAAAATCAAATAGTTAAGACCACTAAAACACAGGAACTCAAAAAGTAATTTACTCTAAAAAAATATAAAGTTTTTATACAACTAATAAATCCAAGGAGTCATGAGTCAGAGCCGTTAAACAAAAATAAATAAATTGCAAAAAAACAAGTAACATAAAATAAGACTAAAGGTACCACGGACAGGTACACACTGCCCGTTCCCCGCATTCAACAGACACAGTCTGAATAATTATTTAATTACTATTATACCCTTTCTCTCTATTTTTCTTAATTTCACATAATTAAAAACATTTGTCCAAGTCAGCATAAATCAAATAAAATATGTTTTTTACAATTTTACATCCAGATTTGAGGCCTAACAAACATCCGGATTTTTCAGTAATTTTTCAGTAATTCAAGGATTTGAAAAATCTTGAATAACAATATATAGATATAAAATCTGTGGATTTCTCCTCTTTTTCCTCGTTTATTCAAATTACCTCCCATAAATTTTGCTTAAAGATAGGGCTCCCTTTGACTAACAATTTGAATAAAGTCAAAAAAACACACATGAAACATGATCAATACGTAGGCTATTACTTTACCTCTATTTGATATTTATTCAACCTTTAAGTTTGCTATAGATGTTAAAAACTTAAAATTACCAAAAATCAATAATTAAAGAGTAGTAATTAGATAACATTTCAATCCCGCTGTTGAGATGTCGCAATTCAAGCTTCGCATTATGTTGTAGATGTTAAAAACTTATAAAATTACCAAAAATCAAGAATCTAATTCTATTATTGATTTATTTTAAGTCGGTTGTTAAAACGAGCCTGAAACCGAGCTTCGTGTATGATCACGAAGCTTAAATAGAAAATCTTAAATATTCATACACAGAAAAAAAAGAGAAAAAAAAAACAACTAATAAATTTGTCGATTAGCGTGAATACCCATCCGTACACACATTAAACCGATTATTATTTTAATCAATTTGTAACTGACGAATAATAAAAAGTAATCATCACTGTATTAAACCTTTTGTTTGTGAATCAAAGTTGAAACAAAATTACGATACGTGATGCATTAATCAACGACGATTTTGATGTTGTTCATGTGAAATTCAATAAAAAATCAAGAGAATCATCAACTAATTACTCAATTTTCAACTTTAATTAGCCAAAAAAAATGCTAAAACAAGAAACAGAGTAAGAAAACTAGGGTTTGAATTGATACCAGATCAACAGTTTCATTTTTAATCTCTTCGAGACTGAGAGCCTTATCGCCGCCCAT

# >HHA4

TCACACTGTGTAATGCTGTTGGATTGTGTCGATATCCAGCCCCTTCAGCTTCACCACCGACTCAACATGCCCCTTCAGTGTAAGCACCTCTCGAAGTCTGTACGTTGTCAAATGTCATAAACACGGTGTTAATTAAATTTAACCACAACCAACGTTTGCCATTTTAAAACTATGATCATCTTAACATACCTTGCAACTTCAGCACGTCTCTTGGCCTGTTCCGCTATTTCCGACAGTTCTCTGTAGCTGCTCTTTTCGTTAAAGATATTAGAGGTTTCCGGTGCTTGAAGACCATGCAATGTCCTCTGAGCAAGAGCCCATTGGGCTTCTCTCTCTTCTCTTCCATAATCCTTCTTCGTCGTGAAAGCCGTCTGTTTTGAAATCACACGGTTAAGCTCGTAATTCCGTCCATATGAAAACTTTATTACCATGTAACAACTAACAAGTTTTGTTAATAGTACATAGTTTTTATTGGCCCACATGTACAAAATTTATAGGTCCATGTTTGCTTTGACTTTTTTTATCAATTTAAATTCTTTATTTTGTTTCATTTGAATTATGTGCTGCTCCGATTATGTTTTATTAATATAATTTTCTTTATTTTGTTTTCATTTGGATGTATTAGCTGCTTTTATTCTTTATTAATTAGCTTTTTTATTACTTTAATGTTTATAAAACTAATTTTAGGGATATTTTATTTTAATTTTAAGTTTTTTATAAAAAAAATTGAATGTTTCATTATGACTTGCTTTTTTACATCTGATGTAAATTTCAATCGAAGCATGGGCTTACAAGTCGCCAACTCTGCTTCAAGGTTTCATTTCTTTATTAGTTTTTATTTCGTTTTCCTCTTACAACCTTTAAAATATGATATTTTATAAAATTTTGAATCTGTCGTTTGATGTGATTTTTTTGTTGAGTAAATACCAGTGTACATGTCGTCGAAACTGAGCCTTACAAATATTTAACACCCCCGCCGCAGCGCGTGGATAATATACCGCTAGTTTATTTCAAAAAGAAACATGAAGAAAAATGTACCTTGTTTTCAAGCATGTTGCGCCAGGCTTTCCCGCTAAGGATGTATCGAATAGCGAATTTCATAATATCGAGAGGGAAGTAGAACACGATGCTGTAGAGCCAGATGACACCGGCCCATCCCCACCCGACTCCCTTGATTCTTGCGAATTCCCACTCTGCGTAGACTGCTATTAGAGTCGCAACCTGAAGGTTTTCATAATAGCAACAGTTGTTAGTTTGTTAGAAAACAAAAGTCAGTTAGTATAAAAAACGTGAAAACGGGTCGTTTCTTTACCAGTTGTGCTGCTAGGAAAGCACCCAATAATAAGAGACCCGGGCGTTCAATAAACGACCAGCTGCGAGAACGCGTTACAAAAATCAAAGCCTGGCTGACTATACTGACTTGGAGATATAAAGCCGCCATCATCTCAACTTCACTATTTCTCAAAGATCTAACACCAAATTTTTCCTGCGTCACACAAACATAAAAAAGTGTTAGTTTGATGTGAGCTTGTGAAATTTCAGTTTCCGGTCAATCAATTCTGTACGTACTGTGAAGAAGTCGGTGTCTTTCATGATCCAGAAGAATATGACGGTCATCAGTGCAAGGTATCCTCCGAGCACGATTCCAGTGGCAAAGATCTCTTTTAGCTTCCAGCTGTCAGGTAACGGAGATGGTTTCACCCTATCCTTTGAGATGGTCATGATGGTACCTTTTGAGTACATACAATGGGAATTAGTGAAGTTACAGTTATGAAAATCAGACATATCAGGCGTAATGGAAGTGAAAACTCACCATCGTTAAGAATTGCAATAATCAAAACCATGAAGGGAGAGAAGTCAAACTTCCATATCAAAGCGATGAACATAAATCCAAACTACAAATGATTCAAATAATCACATGCCACATACAAAGGGATGAACATAATGTCATAATCCTAATGGGCAATGATTCAAATGTTACATATAACTTACCACAATACGAATGGTGATGGAGACGGCATAGATCTGCAAAAAGAAAAGATGTTAACAGTTAACACCTACAAACTGCTTGCATGGAATAATTATAATAAAATAAGGGAAAGAGAGGAACTAACAGTATAATTCTTCATTCTTTGGAAAATAGCGCGACTTGTAAGCACTGCACTGATAATAACACTAAGCCCAGGCTCGGTAAGCACGATGTCCGAAGCACTCCTTGCAGCATCTGTAGCATCAGCAACAGCAATTCCAATGTCTGCTTTCTTCAAAGCAGGGGCGTCATTCACACCATCTCCAGTCATCCCACATATGTGCTTTCTTTCTTGTAACTTCTTCACAATCTCATACTTGTGTTCTGTATATTTAGATCAAAAGAGTCAGTATATCAAGATATAATATAATATATTAATACCAATCAAAGGAGCACTAACCTGGAAAAACTCCTGCAAATCCATCGGCTTTCTCTATCAACTCTTCAATAGGAAGTGTTGCAATGGATGGATCCTTGTGGCCACCGAGCAAAGAAGAAGAAGGGTACATGTTTGTTCCCATACCGAGCCGTCTACCAGTCTCCTTAGCAATGGCAAGTTGGTCGCCTATAAATGATACAGTCATAATTAGATAAAAAAAGAAAGAGTAAAATGCCATTTTCCTCCTTGTGGTTTGGCCAGTTTTGCGAGTTTCGTCCAAAGGTTTGTTTTTCCGCATCTGGATCCAAAAGATTTGAAATCTTGTCATTTTCATCCGGCTCGTTAACTCCATCCATTTTTCTTTGTTAATTCAGGGGTATTTTCGTCTTTTTTGTTAACTTAAAGGGCAATTCGGTCTTTTTTTTACTTTATGTACAAGCATTTAGCATAATGTACAAGTACGCAAAGCCCTTTAAGTTATAAAAAAAAGACGGAAATACCCCTGACTTAATGGAGAAAAATGGATGGAATTAACGAGATGGATCAGAAATGGCAAGATTTCAAACCTTTTGGATCCAGATGCGGAATAACAAACCTTTTGACGAAAGTGGCAAAGCTGGCCAAACCTCAAGCATGAAAAATGGCTTAAAATTAAAAGTAGTTATTCAATATAATCAGAAGATATAGTTGATTACCGGTAATCATTTTGACATTTACACCAAGATGGAGAGCTTGGCGAATGGTCTCAGCACTGTCATGCCTTGGTGGGTCGAATAGGGACAACAATCCAACAAATTGCCATGGTCCACCTGGGCCTTCTTTTGTTTTTTCAGGCACTTCCTACAAAATCATCACAATGAAAAGAGTAAGTAATCACGATCATAAAATCAAAAATAGATGTGTGTGCATACAAATGATGTATAATCCTGACCTGTTTTCCAACAGCCAAAGAACGCAACCCACGTTCAGCAAATTTATCAATCATTGCGTGAACTTTCTTTTTCATATCTTCTTTGCATCCACAGAGGGTCAAAATCTGAAAAAAAAAGTTTGGATATATAACCAACTGATTCTGTGATTCATGAGGATCATAATAAAATAAGAAAATAAGTTAAAATTACCTGTTCTGGAGCACCTTTGCTTGTTCTATGCCAATTTCCACGCTCATCGATGTACGTCAAAGCTGTCCTCTTGTCAACAGGGTTAAATGGAAAGAAGTGAACCTCTCTAATTCCAGCTCGTGCCTACAAAGTCACGTATGTCATCGATGAGCATGAAATCAGATTGAAACGAAAGCATTTTAGTTATCAGATACCTCTTTAGGATCAGCAAGGGTTCCAACAATGGCAGCATCAATAGCATCCTGGTTCTCAGTCCTAGAAGCCCTAGCAGCATAAAGCAGCACCTGCTCCTTATCGACACCTTTTGCAAACACCTCAATTAAATTTTTATCAACTGTAAGCTTGTTAAGTGTCAGTGTTCCCGTCTTATCACTGCAAAGCACATCCATTCCAGCCATTTCTTCAATTGCAGTCATTCTTTTAGTAATAGCACCCTGCTGTGAAAGCCTATGCGATCCAATAGCCATGGTGACCGAAAGCACAGTAGGCATAGCAATCGGAATTCCACCGATAAGCAACACCAAAAGATTGTCAATCCCACTTCTGTACTGCCTGTGCTGAATCGGGTACATGACGATTATCTCAACCAACATTCCAACAGCAATGGAGCAGATACAGAAGTTTCCGATCGCCGTAAGAACTTGTTGGAAATGACCAACTTGGTTAGTGCTATCCACAAGATGTGCAGCCTTTCCGAAGAAAGTGTGCACACCAGTGGCGATAACAACCGCTTCGATTTCACCTTGCTTGCAAGTTGATCCGGAGAACACCTCGTCATACGGATTCTTGTTCACAGGAAGTGATTCACCAGTAAGGGCAGATTGGTCAATCTTTAACGGATCACCTTCTAGAAGACGAGCATCAGCAGGAACAATATCACCAAGTTTGATACTGATAATATCTCCCGGGACCAAGATGGAAGCTTCTTGTTCACTCCAACGACCATCCCGAAGAACCTGCAGTAACGACAGATTCGTCAATCTTTGAACTTATAGCGGCATTTATATATCGGTCAATTAAGTTTCGGAATACCTTGGTTTTAGGGGCGAGACCAGCCATAAGTGCAGCAGCCGCGTTTCCAGCATTGTTTTCTTCAATAAAACTGATGGTTGAGTTAATAACAAGAAGGCAAACAATACCAACAAAGTCTTGCCAATCTGGTGGCTTTCCACCTCCATTAGCCATTGCGATTGCCATAATGGCTGCAGCTTCCATGACCCACGATAGAGGATTCCACATGAACCCGAGAAACTTGAGTATTTTGCTTTCCTGTGCTCATTATAACATATAAAAATCATTAAGCATTTGGAAAGAAAACAGAATCCAACATAAGTTTGATCATGATCATGATTGAAGTAATGAAAATACTTTTTTCTCCTCCAATTTGTTGGGTCCGAAAATTTGCAGTCTCGCAGCTCCTTCATCTGAGGTTAGACCTTCTCGGTTACATTTCAACTGTTGAAACACCTCCTCAATCGGTACTTTCTCCTGTAATTATATGTCGAGAACGTTACCATTCTGTTAGATCCACAGTAATACTAGATAAACTTGTATGATAAACAACTGAATGGACAACGTTGCCATATGTCAGCTGCTTGTGTCATGATGCATGCTTGGATAACACTTAAGATTGTATTTGTTAACTTGTTTAGTTATAATGACTTGTACTCTTTGAAACAGGTATCATGCAGTGGGTTTGGTCATTGTAGTAATAAAAGAAAAGTTTCAACTTAATTTACATGCCTTTGATAGTAAGGTAAGACTCGAAAGTATCAAGTAAAGTCACATATAAGCATGCATTTATACATTAAAAATACAAATAATTCAATTAAAGACAAACAATAATTAATTACAAATTAACAAAATAAAATATTATTATTACTGCTTTAGATTATCTTCATGTTTATCTTTACTTTATTTTTATATATTTATATTCCAAAATTCCATGTGTCTGTTATAGGGTTCCAATGATTATGACCCACCACAAAAAAAATCAGGAAAGAGAATCAAGAATTAAGGTCCACTGAAAATGGGAACAATTCTTTTTTTTATTTAGAAATTAATTAAAAATAAAGTGCAGGGAAAAACCAAACTATCTACCAAAGGGTTTGTCCCTTTACCACTTTTTGCTAAGGTGGTTTGGACACACACATAGATATGGAAACTAGACAGACATGTAACGTGACTGGCCATCACTTGTTGGTGAGGTTTGGCATATATCCCAATTCCCTTATCAAATTCACAACTTTAAGATACTTTAGGCTAATTGGGTGCGGTGTGTGTGTTTGTGTGTCTCGGGGGATGTTACCAAAAAAAGTCGCATATAACTCTGTTAAGTCCCAGGTGAGAAATATAATATATGTATATACGTGTGTGCGCACACAATAGTAACACTAATGGTTATAAAAGTGATATCATAAATTCCAAACTGGTAGAGGTGTTCACGGTCCAGTTTTTTGACAAAAAAACTTTGGTCTGAACCGTTAACCATGGTTTCAATAAACAACAATTCAAACGGGCTGGGTTGGTTGGGTCGACCGGTTCAGTCAAAAAGGTTCGATTGTAGCGGTTAGAACTAGTTTTTTTTTTTTACTTTTTATATTGAATATTGTTTGAGTTAAAACAAAAAAAAAGTATTACATATATAAAATAACCATATGTTTCAGAAAAAAAATAGTACAGGTTAAAGAACTAACTTATTTACACGGCTAGTTACAATTTCCCATACTATTCAGTTTAAATGATTCAGTCATTTTGATACATGGAAGATATACTGATTTACAATTTTTAAAAGAATCATTCAAATTGAATAGTTCTTATTTTCATTCGGTGGAAATAAGATAGAGGGTGTGAGAATAGTGTAACAGCATTCACATCCTGTTTATCATGATTACACTAAAATCAACTAATTTTATCTCCCCTATTCAATTAAATAATATTTTTATAAATTTATCATTGCATTCTTTTTCCTCTATTCAAAATCACTTTCTAAATTTTTTTTTTTTTAAATTAAAGATAAACAGTGTTTTTCATATTTGCATATAAATAGTAACTAGTGGGATTACCCGCGCTTCGCAGCGGGTTTTGATCAGTATCAGTTCGGTCAGATACAGTACCAACACTCAAAATAGCAACCGAAAAAGATATGCACAAGAAGATATTGATACTTGTTTACATCGATAGAAAACCATAAAAACGAATGCCAATATCGGTATCGATGTTTTTCAGCTGGTAACAATTCGATTCATCAAGCTAAAGTTATCATACTGGCAATTGTTACCACTACGGTATAAAAATACTCTATTGCGAACACAACTCTATTTTTGACAAACTTTATACCGCCAAAGAAAACTATAAAAATAAATATGGTACCAGTACCGATGTAGTTCAGATGGTATCGGCACAATTTCTTACGATAAAGAAACAAAATCGGCTATATTTGACTCGTTCAAATATATATAAACGAAAAACAAAATATATTAAAAGTAATTATAATAATACAATATTATAATAATAATAATAATACTACAGTAATAGAAATTTTAAATTTTAAAGAAAAAAATAACAAAAATTATTATTAAAAGTAAAAGTAAAAAAAAAAACAATAACAATAAAAATTTATTGTTAATTAAGTTTGCTTGATACAAAAGTTAAATTAAATATATATTTTTAGATGTATAATTAGATGTTACTGTTTATCCTCTTTGAAAATGTAAGGATGTATAATAGTAATATATTTCTCAGCGTTAGCGTTGATAAATTTAAAAAGTTGAAATATAATATTGTTTTTCTAGGGAAGTGAGTAAAACAGAAAACTGTACACATGGGGTGGCTGATTAAAAACAACCCGAACAAAGACATGGAGGGTTGAGTAACGCAAGTTGACTTTCCATTTAACTTAAAAGGGTGGGACCTACACAACCCCCATCCACTCAAAAGACGGCACCTTGGATCAGAGCAACAAACGGGTGGGTATCACTCACGTACCGAATACCAAAAAGGCCCTAAAAGCCAACCAAACCACACGAGAAAGCAAGGGTACATCGGGTATTCCTTGCCAAAAAAAAAACTAAAAAAAATATATTCTTACCAAAAATATATGCAAAAATTAAATTAATTTAATACAAAAAATTATCTTTACTAGCTTTGACAGGAATATAAGGATGTTACGTATCTAAATTTAGATGTGAAACTCTTAAATTAAGTCGGTTTTATTATTGTATGAAAACGATGACATTTTAATAAGACATCTAGAGACTACTTTTCCGAGTTTTGCTATTTTTGTTAAATATTTGTCAAAGAAAGAACTTTTGATTTGAATGGTATAATTAGAAATATATATATATATATATATATATATATATATATATATAGTTTCTACCTATTTTATCAAAGTGACATAGCTTTGATGCATATTAACTACAATAATATGATTTCGAGTATCTTTGGGTGTATCAGTGTATGATAGCAATACAAATTAGTGATTTGGTCTTTAACTTCTCGTAACATTTTTTATAAAATGAATTAGAAACTATTATTCTTTTACCTGATAAACAAAAATCCGAGAAACAAGGTTAGAATAAATTGACCAAAGAAAAACATAAAATAAGACCAAGGTACCACGGAAATGTACCCGCATTCAACAATCAACATACACATTAAATTATTTTATTAAAATTAAGATGCTTAGGAAACTAAAACAGTTACAACATAGAGAATATTATTTCTTTTTTAAGGTTATACCGTATATAATAATATTTTCTATACACAGTAATCTTTTATTCAGATAGAAACGTATAAGTGGATGACAATTGTGATGCGTGCAAAAAATAAAGATCATTATATTGTGCAATTAATGCATACTATTTGTTTAATTATAAATCATTTACTTTTTTTCCCTTCTATTTAAAAATAAACAGAAATAAAGTACTATCACAACAGAATATATTCAGATTTAATGCCTAACAAATATCCGACTTTTCCAATGATATTCTAACAACTTAGCGTAACAATAAATCAAAATAAAAAATATGCAATAAAAATATAGTCAGATTTTTTCAATGTTTCCTCTCTAATTTATTCAAATCACTTTAAAAAAATTAAAGACTCTACCAACTGATCAAAGCTAGAGTAACTTAACTTCAAATTGAGTAAACATCTTCCGTTGCATGTAAATACAAATTTGAATAAACAAAAAATTTGACCAGTTTACCGTTAAGCTGTGATTACCGCATAATACATTTTATATACAGTAGTAGTACATAACTGATCGACTTATGTATTAAAATTAAATCATATAAATAAAATTTTAATTTAAATATTTAAATATTTAAATATAATATAAATATAAACAAAAGGAAACTAGTAATTAATTTGTCGATTAGCATGTAAACCGTACAAAAAAATAAAATCATTATTACTTTAACAATCAAAACGTAACTGATCTCAAAGGAATAACAAAAGGTAGTCAGTGAAGTAAAGTGTGAAGAGAAGAATTCGCATGCTAAAGCATCAAAAAGCAAATCACAAAACAAAATTATCATGAATATCATGACGCAATTTACTAACGATGACGTTATGTTAATCGTGTTCATCGCAATCTCAAATCGAATTCAAATCAACATCAAATTAAACCTAAATTGAAATGAAATTCAAGTTCAATGACAACAACAACGATCGATCGATCGATGAAAAATCAAAACAGTAGAGAAAACAAAAGTAAATGAAGAGTCATACCAGATCAATAGTTTCGTTTTTAATCTCTTCGAGACTGAGAGCCTTATCGGTCCCCAT

# >HHA5

ATGGCGGATTCTGGCATCAGTTGGGAGGAACTCAAGAAGGAGAACGTCGATCTCGTACGATCTCCTTCCTTTTTCCCTAACCTTTATGATTCATTTCGTAACCTGTTGTTGTTCGATACATTATTATGATTCATCGTCTGTTTGATAACGATTTGATTGATCTATCGATGCGTTAGGAGATTCGTAGATTTGTAGTTAAATTGTTGTGTTTTGCATGTAGATCTGTTGATTAGTTCAAACCGTTTGATGATTTGAAACTATTGTTATGTTAGGTTATTACTGACAGACTGTTTGATTGACTGTAAGCATTGGTATTTTGTTTTCATGATTCGTATATAACGCGCTTACGAATTTGCATGTAGGATTAATTTCGTAACCTGTTGTTGTTCGATAGATTATGAGTTGTCGTCTGTTTGATGATGAGTAGGTTGATCTATAGATGCGTTAGGAGATTCGCCGATTTGGAGTTTAAATTGTTGTGTTTTGCATGTAGATCTATTGATTAGTTTCGATAGTTTGATGATTTGAAACTTTTGTTAGAGTATTACAGATGATTATTCGATTGAGATCGAAATTTTGTGTTTATGATTTGATTGAATCGATGAGTTAGAAGAATTGTAGGTTTGTAGTTTCGGTTCGTTGTGTTTTGCATGAGGATCTATCATCTATGGACTATGGATTCAAATTGTTCGATAGTTTGAAACTATTGTTAGGTTATTACCGACTACTGTTCAGTTGACTGTAAGCATCTGAATATGTTTTTCATGATTCGACTATAACGATTATGAATGTACTATTTAGGAACATTGTAGTTTTTTTTTAACCGAACATAATCTGAAATTTTTGTTAGATTATTATAGATGACTGTTCAATTGACTGTAAGAAACGGAACTTGTTTTTCATGATTCGATTATAACGCTTACGTATTACAGACAATGTTTAATTGACTATAAGCATTGGAATTTTGTTTTCATGATTCAATTATAACACTTATGAGTTTGATTGGGGGATTTAGGAGAATTTTAGTTTTTTGAATAAACATAATCTAAAACTTTTGTTAGATTATTACAGACAACTGTTTAATTGACTATGAACGTCTGAACTTTGTTTACATGCGCTTACGAATTTGAATGAAGGTCTTAGGAGAATTGTAGTTTTTTAATCTAATATCATCTAAAACTTTTGTTATATTATTACAGTCAACTGATCAGTTGACTATGAACGTTGGAATTTTGTTTTCATGATTAGATTATAACGCTTACGAATTTGAATGGAGGATGTATGAGAATTGTAGTTTTTTACTAATTGATTTGGTGGTTTTTAACTTTGTTTTGATTGGTACGTACAGGAGACTGTTCCGGTTGACGAGGTATTTGAAACCTTGAAATGTACAAGACAGGGATTGACAACTGAAGAAGGGAACAGGAGGCTTAATGCATTCGGCCCGAACAAGCTGGAAGAGAAGAAGGTAAATCAATAACTGCAAATTGTTTTTGATTTCTAATATAATTTTGTTATACTCATGTTTATGAAACTGATGAGATCTTATTGATATACTTGAAACAGGAGAGTAAATTTCTCAAGTTCTTAGGGTTCATGTGGAATCCGCTCTCATGGGTCATGGAGGCTGCCGCAATTATGGCCATTGTCTTGGCCAATGGAGGGGTATTTTTCTTACTTGAATAAAAAAACAATTTATATACAAAATCGCGTAAACGTATCGAGTTATCAACCCCATCAAACTTGTTGAGGCTAGTCAGGAACTCAGGATACACGTTTTGAGAGGATCATTTAATCGGATAAAATAACGTGTAGATGTGATTTTGTTGCTAGTTTGGTATTTAAAGATAGTAATGGACTAATTACCAATCAGTGTTATTGTAGCTAGTATTAAATTGATTATTATTCGTTATAATTTATCATAGTCAAAAGTAGATTAACCCAAACTGGATGCTAATGATCAGATAACCACTTTACAAAAAACCATGTGACATAAAAAAACTGTAGTATTTGGTGTTATCTTGATGCTATGGTATTTGTAGAGAACAGCTATCATTCATATATATTATACATCTATAATATATACACTACATACACGTACTGATATAGATAACTTGATATCAGGGAAAGCCTCCAGATTGGCAAGACTTTGTTGGAATCACAACATTGCTGATAATCAACTCGACCATCAGTTTCATAGAGGAAAACAACGCAGGCAACGCTGCAGCCGCTTTAATGGCAGGTCTTGCACCAAAAACAAAGGTCATAAGGGACGGAAAATGGGACGAACAAGACGCAGCCATTTTAGTACCCGGAGATGTAATCAGTGTTAAATTGGGAGACATTATTCCAGCTGATGCGCGCCTTCTAGAAGGTGATCCGTTAAAGATTGACCAATCTGCATTGACTGGTGAATCATTACCAGTGACCAAGCATCCAGGTGCCAGTGTGTATTCGGGCTCGACATGTAAACAAGGTGAAATTGAAGCGGTTGTTATAGCTACTGGGGTCCACACGTTCTTCGGGAAGGCTGCTCATTTAGTCGATAGTACAAACCAAGTCGGCCATTTCCAAAAGGTACTAGTGTAGCAGAGGTGCACAAAGCGGTTAATTTCCCTAACTAGTTCGGTTAACCGGTTAAGGCGGTTATTTTCTAATTTTTGCTTAACCGATTATTGCTTTAACGGGTTATTACTTTAACTGGTTCGGTTATTCACTTTAACCGGTTATTTTCAACAAAAAAAGAAATCATTTTTTTGGTTTTTTCCCGGTTAACCAATACATCGGTTAAAAACCGGTTATTAACTAGTTGCAGTTCCTGTTAATAACCGGTTTCGGTTATTTTGCCCACCTCTATTAAGTGTAGTAATGTCACAAAGATTAGGCCCAACATATTAGTGTCACTAATATGGGCCTAACAATCCTAACCTGCCTGATTCTATTTTTATTTTAATATCTAACTAACTCCTAGCATATTTATACTTTTAAATTATAATTACACCATGGCTTAAATCAAAGGAAGGTATAATAAGACTTTTACTCTTATAGTAATTAAACTTTTGAAATTTATTTGTTTTTTTAAATTATCTCATATTAGTTATATAAGACATAAAAGTATATAAATTCTCATTTTTTGTTACATTTTAAAAGAGTTTCACTAAAAAATGATTAAATAGTATAAGAAAATCAGAAAATTACAATCACATTGTATTTTAGTTTTTACATACTGGGCTTACTGGACCGGGCTTTAGAACCCGACAACGGGCTTAACAGGTCCTAGTATTGGGCCTAGGAATGGTGCTGGTTTGTGCATTTTAAAGCATTCATTCTTAACTAATAACTACCATCATGTTAACAATATCTGATGGACTGTCCGTTAGGTGTTGACCTCCATTGGAAACTTCTGCATATGCTCCATCGCGATCGGGCTCATAATCGAAATAGTGGTGATGTACCCGATTCAGAAACGAACGTATAGAAACGGAATTGACAATTTACTGGTTCTTCTCATTGGAGGAATCCCGATTGCCATGCCAACTGTTTTGTCAGTCACCATGGCCATCGGGTCCCACAAGTTGTCGGAACAAGGTGCAATCACCAAGAGAATGACAGCCATTGAAGAAATGGCTGGGATGGATGTTTTGTGTAGTGACAAAACTGGCACTCTCACCCTCAACAAACTCACAGTTGACAAAACTCTCATTGAGGTCAGTTTTAAGTTTTAACCTCCGCGTTTGTTTAAAACGGGACGAAACAATCAGGGTTGACCCGCAAACACTTTTTCAACTTTATTTTTAAATAGTTAATGTTTCAAGTATGGTTAAAAAATAAACGGGTCGAGAATGCTACCTCTGATTGACATTGAACAACTGAAATAAACTCCTTTCTTCTTCAGGTTTTTGTAAAGGATGCTGATAAGGATCAAGTGGTTCTATTGGGAGCAAGGGCTTCAAGAGTGGAAAATCAAGATGCAATAGATGCTTGTATTGTAGGCATGCTTTCAGATCCAAAAGAGGTAATGACATAACAACAATAAAAATAATGATAAGTTAATAATAATAATAATAATAATAACAATAATAATAGTAATAATGATAATAATAATAATAATAATAATAATAATAATAATAATAATAATAATAATAATAAATATAAATATAACAAAATGCCTTGAATATTTCTTTTCTTTATGCAGGCACGAGCAGGGATCACTGAGGTGCATTTTCTCCCCTTTAACCCAGTGGACAAAAGAACAGCTATAACATACATAGATCAAAATGGAAATTGGCATAGAGTGAGCAAAGGTGCACCCGAGCAGGTACAATAAACTCCTTACCCCTCTATAGTGTAACTAAATGTGCTGGCTAACGGGTCAAAACAAATAACTATTTGGTATAGCTTTAAACGCATAAGTTGACCCGAAACAACCCCCCCTCTAAGAAAAAAAAGGAATTTCTTCTGTATTATTCTAATAATAGTCAGACTTTCGGCATACCTGACCCATTTTCCTTTTAGCTAATTTTTATTTGACCCGTTAAAGATAACACAGATCTCAAGTCAACCCATTCATAATAATCCTATTTCAGTTTATATGTACATGATATCTCAAAATATCTCAGATTGTGGAGCTATGCGACCAAAAGGAGGAGGATAAGAAGAAAGTGTTTTCCATAATCGATAAGTTTGCCGAACGTGGTCTTCGTTCTCTAGCTGTTTGCCAACAAGTGAGTTTTTAACTTTTTATATTTCAGTTTTTTAACCTGGAAACTAATTATTACTAATATTATATAGGCGGTACCGGAGAAAACAAAGGAGAGCGCGGGAGGCCCATGGGTGTTTGTGGGTTTACTGCCACTGTTTGACCCACCAAGGCATGACAGTGCTGAAACCATTCGACGGGCTCTACACCTTGGAGTCAATGTGAAAATGATCACGGGTGATCAGTTAGCCATTGGCAAGGAAACGGGCCGAAGGCTTGGAATGGGAACAAACATGTATCCTTCTTCTTCACTTCTGGGCCAAAATAAAGATCCATCAATTGCAGATATACCAATTGAGGAGCTTATTGAGAAAGCTGATGGTTTTGCTGGAGTCTTCCCTGGTAAATGATTCACACTCACTCCTATTAGATAGGGATGTTAAAAGTTAAAACAAACGGGCCCGGTAATAGGTCAAAACATGAGCGTTTTTAGTGCGAGTTGAATGTAGGGGTGCTAAATGGGTCGTGTTCGCAGGTTGACCCTACCCGAACCCAAAAATCGTGTCATACATTTGAACCCAAACACGACCCGTTCAACCCGATTTTTATTATTTTTAATTTTTTCTGCAACTTAATATATTAAAATCAAAATTTAATAAAGAAAAATACTAATGTATATAACACAAATACATTTAAGTTATGAAATCGTATGTCAATTTCTCTTTTTAAGTTATAATTATGGCATAAAATACACACCCAATGTAATTTATGATATAAAACATATTGTAAAAAAGGTATAATATAATATAAATGGGTTAAACGGTCAACCCGCCAATCCGACCGGGTTGACCTGAACACGACCCGTTTAGCTAAACAGGTTCACAACTTCAACCTGAAACTGACCCGAACTCGTTTAGACTAAACCCAAACCCGTGAATTTTGTGTTAGGTTGGTGTCGTGTTTTCAAGTCGCGTTGGAAATTCACACCCCTAGTTAAATGGGTTGGGTCGGGTTTGACATACCCACAAACAACTTTACATTCATGTGTTTTGGAATTTTTATAAATAGTTCAGGGTTAATATATTTAAAATTAGGGATAATTGTGCAAAATAGCAACATACATCAACTCTTTTACCAACAATAGTCAAGTCTTTTGAATTAATATTAAACAAGTAACATATTTTTTTATTTTGTCAATTATAGCAACCATAGTTATATAAAAAAATTGGTAGAAACATGAAAGTATGTAGCTATGATTGGTAGAAAAGGTGAAAGTATGTTTATCTTTAATAGCTTTCATCATCTACTGTTAAACCTTACTCTTTTTTTTTTTCAGAACACAAATATGAAATTGTGAAGAAACTACAAGAGAGAAAGCATATATGTGGAATGACAGGAGACGGTGTGAACGATGCACCCGCATTAAAACGGGCCGACATTGGTATCGCAGTGGCTGATGCAACGGATGCAGCCCGTGGTGCATCCGATATCGTGTTGACCGAGCCTGGACTCAGTGTAATCGTCAGTGCGGTTTTGACCAGTCGCGCCATCTTTCAAAGGATGAAAAACTACACAATTTATGCCGTCTCTATCACCATCCGTATTGTGCTCGGGTTCATGTTAATTGCACTTATATGGAAGTTTGACTTCTCGCCTTTTATGGTTTTGATTATCGCGATCCTTAATGATGGAACCATTATGACTATATCAAAGGATAAAGTCAAACCTTCACCTCTGCCGGATTCATGGAAGTTGAACGAAATCTTTGCCACTGGGATTGTTCTTGGCACTTATTTAGCTGTTATGACTGTAGTTTTCTTCTGGCTAGCAAAGGAATCTGACTTCTTTACGGTAAGATAAACCGTTGATTGTTTAGTTGGTTCACAGTTAGTGAGATGAGCGGGTTGGGTAATGGTCATATGGAGGGGGTTGAAATGGGTCAAAACGGATTCGCGTAGGCCAGTTTGACCCTTTTCAACCCATTTTGACCCATCCATAAGTAAATGGGCTGGTGGAGCGGGTTGGTAACGGGCCAAAACAAGATTTGATCAAATGGCTCAAAACGTGTTCAGTTGACATGTTTGACCCGTTTCCTTTTTAGCTGAACTTTATGATTTACTTTAGATTTTAAAAAAAAAAGTTTAACAATAATAATAAGAATAAGAAGAAGTTTAAAAAAATAAAAAAAAAATCTAGAGTAAAATGTCACTTTCATCCCTGAGCTAACTGAGTTATGAGTTTTTTTTTAACTGGGGTTAGTTTTTTCGGACTGAAATGGCATGTTTCGAGAATGTCAGGGAGGAAAATGGTACAATTTGGAAATTTGGTCTGAAATGGCAAAGTGGACAAACCACAGGGACGTAAATGGCACTTAACTCTAAAAAACGTAACCTATATCGACCCATTCATATGTCAGTGGGTTGGAATTGCAACCTCTACACAGTATCGTTCAGTTATTAACTAACGATATTCATTAATGACTGTATTCTCAGGAGAAATTTGGTGTGAAGCCGATTAAAGACAATGAATACGAGCTTATGTCAGCTCTTTACCTACAAGTCAGCATCATAAGTCAGGCACTCATTTTTGTTACAAGATCAAGAAGCTGGTCTTTCGTTGAACGCCCCGGTCTTTTACTGCTCACCGCCTTTTTTATAGCACAGCTGGTGAGTTTATTCTTATTGGGCTGACCCGACACACCTTTTTCAAAACGAACATAAAATATTTTTACATAAAGAATTCTAACAAATCTTTATTTAATGAAGATCGCTACATTAATCGCTGTCTACGCAAACTGGGACTTCGCAAGAGTCCATGGAATCGGCTGGGGTTGGGCCGGTGTGATTTGGCTCTATAGCATACTCTTCTATTTCCCATTAGACGTCTTCAAATTCATCATCCGGTTCGCCTTGAGTGGCAAGGCATGGGACAACATGCTCCAAAATAAGGTAAGCCCGTTTCGTTAATCATCGTTAATATTTTTAAACTAAATTGATCGTTATGTTAGTTATTATTTATTAACACGCAACTTTTGTGTACGTCTAGACTGCTTTCACCACTAAAAAGGACTATGGACGGGGCGAGAGGGAAGCCCAATGGGCCTCGGCCCAACGCACCTTGCATGGCCTCCAAGCACCGAATGCAAACGACATTTTCAGCGATAAGAGCGACTACAGAGAGTTGTCGGAACTTGCAGAACAAGCAAGGAGGCGGGCGGAGGTTGCTAGGTAAGTCTTGTTTCTTGATGGGTCAACTGTCTTGTTTATAAACATGGTTGTAGAAATCTCGACAAGCCTCCGATTAGCCGTTGATTAATTGCTAGTCGGAGGTTCACCGAGTAACTTTCATCTAATCAACCAATTAATTAGTAGGCGGTCAACAATGGTCAATTTCAGTCCTAATTGGCCAAAATCGGTGGCAGTCTAGCGATTCCAGCCAGATTCTTGACCAAAATCTGTAAATTCCGACAGTTAATAATATCTACTTGAAAATATGGGTCGAAGATGGTGTTAGAATATGTCTACTTAAAAAAATACATCTAAAAATGTGTATATACATATAAAATTGAAAATTACATATAAAATCCAAATCCGATTAATCTCGATTAATCGCTAGTTGGTACCCCACCAGCGGACTAGCGCCTAGCATTTCTTACAACACTGTTTATAAATACATGATTCAAAATGTTGTTGAAAAGTCAACTTAACCTACCCTAGCTCGTTTTGACTAAATGTATAGATCACATGTTTTGACCCGTTACCTAACGTGACAGACTGGCCCATACTACACTAAATTAATATCTATATTTGTTGTTTGTTATAGGTTGAGGGAGCTTCATACCCTCAAAGGGCATGTCGAGTCGGTGGTGAAGCTCAAGGGTCTGGATATTGAAACCATCCAACAACACTACACGGTTTAA

# >HHA6

ATGGCTGCTTCCGGCATCAGTTGGGAGGAACTCAAGAAGGAGAACGTCGATCTCGTATGATCCATTCCCCTCTCTCTCCCTCTCTCTCTCTCTCTCTATATGTATATTGCTTTAGTATTTTTAGGTTTTGCATGAATTTTGTGTTGATTAGTGGAGGAGACAGTTCATTTTGTTATGATTGATTGATGATTTCTCGATTCGATTAGGTTAATTATAGTTTTGTAATTCTGTTTGATATGTTTTGCATGAGGATTTGATCGGTTGATTATGTTATTTGAGTATTTTGTGTTGATTACTGAAGGAAACTGTTCGATTAGTTAACGATTTCGTTACTAGATCTAGGCGAATTGTAGTTTTTCTCGTTATATTTGATATGGCATACGATTAAATGAGGATTTCATGATTAGATCTAGGATTAACTGATTCGGTAATCTGTAACTCTTGTGTCGATTACTGAAGGAAACTGTTTACTTTATTATGATTGATTGAAGGTTTTGTGATTCGATTAGGCGAATTGTAGTTCTGTAAGTATTTGATATGTTTTGCATGAAGATCAATTGATTCATTAGTCTATAATTTTTTGTTAAGATGTGAAGATTGATTGATGACTTCATGATTTGATCTAGGCGAATTGTAGTTTTGTACTTCTATTTGATATGTTTTGGATAAGGATCAATCGATTGAATCTGTTAATTGAATTTTTCGTGTTGTTGATTACTAAAGAAGACTGATGAATTAGTTATAATTGATTGACCATTTCATGTTTCGATCTAGGCGAATTGTGGTTTTGTACTTCTATTTGATATGTTTTGCATGGAGATCATTTAATTTGGTTATCTTAAATTATGTTTTGATTGTTACAGGAGACTGTTCCAGTCGATGAGGTATTCGAAACGTTGAAATGTACAAAAGCGGGATTGACGACTGAAGAAGGGAACAGGAGGCTTGCTGCTTTTGGCCCTAACAAGCTAGAAGAGAAGCATGTAGATACAAAACTATTGATTATTATTAGTTTTATAATAATATTATTTATTTTTTTTATACTGAAGCTCAAATTCTATTGATATTGTTTAAACAGGAAAGTAAGCTTCTCAAGTTCTTAGGGTTCATGTGGAATCCATTGTCATGGGTCATGGAAGCCGCTGCAATCATGGCTATCGTCTTGGCCAATGGAGGGGTATCTCTCTTTCTCATTTCTCAAACCGATAATAATTCGAATATAAGTAATGTAACCTGATCTGGAATACCGAATTGCAGGGAAAGCCTCCGGATTGGCAAGATTTTGTTGGTATCACAACATTGCTGATAATCAACTCCACCATTAGTTTCATCGAGGAAAACAACGCAGGTAACGCTGCAGCGGCTTTAATGGCGGGTCTTGCTCCGAAAACTAAGGTCATAAGGGACGGGAAATGGGATGAACAAGACGCGGCTATTTTAGTACCCGGGGATGTGATTAGTGTCAAATTGGGAGATATTATTCCAGCCGATGCGCGCCTTCTTGAAGGAGATCCACTTAAGATTGATCAATCTGCGTTGACCGGTGAATCGTTGCCTGTGACCAAACATCCGGGTCAAAGTGTGTATTCCGGGTCAACATGCAAACAAGGTGAAATCGAAGCGGTTGTTATCGCTACTGGGGTCCACACTTTCTTCGGGAAGGCTGCTCACTTAGTAGATAGTACAAATCAAGTTGGCCATTTCCAAAAGGTATCATACTTGTTAAAGGCGTCGTTGAAACGCTCGGTTTGGGCCCAATGGGCCTTAACCATGCCCGATTCTATTTTCTCAAGAACTGGACATTTGGGCCTAAGAGGTCCTAGGACTGGGCCTAAAGTAACCAGACCTTTTCGGGCTCAGGCCCATGAATGTTTATGTATCTCTAAGTATTCTGTTAACAGTTATTGATGGATTGTATGTAGGTGTTGACCTCTATTGGAAACTTCTGCATATGCTCCATTGCCATTGGTCTCATAATCGAAATAGTGGTGATGTACCCGATTCAGAAACGCTCATACAGAAACGGAATCGACAATCTATTGGTTCTACTCATCGGAGGGATCCCAATCGCCATGCCAACTGTTTTGTCAGTCACCATGGCTATCGGGTCCCACAAGTTGTCGGAACAAGGTGCAATCACCAAGAGAATGACCGCCATTGAGGAAATGGCGGGCATGGATGTTTTGTGTAGTGACAAAACTGGTACTCTTACGCTTAACAAACTCACAGTCGACAAAACGCTCATTGAGGTTTTCGCAAAGGATGTTGATAAGGATCAAGTGGTTCTATTGGGAGCCAGGGCTTCAAGAGTTGAGAATCAAGACGCGATTGATGCGTGCATTGTAGGAATGCTTTCGGATCCTAAAGAGGTAATAACTAATAACAACTATAAATTAAATACTTAAATAATAATTATTAAACGTAATAATATTATCTTACATGAATATTTCCTTTGATCATGCAGGCACGAGCGGGGATCACTGAGGTGCATTTTCTGCCTTTCAATCCTGTAGACAAACGAACAGCAATAACGTACATAGATCAAAGCGGAAATTGGCACAGAGTGAGCAAAGGTGCACCTGAGCAGGTACCGTACAATAAAAATTCTCTCGACTACAACTACTAAACGGGTCAGGTAACGGGTCAAAAGGGACAATTTCTCGGTACCCTTAAAACAATTTCCCTCAAAAAGTTATAAATTTCTTTTATAAAGGACCGTATGTTACATACGATTACAAAAAGTTATACATAGCGTGAGTTCTGACTTCTGACCCATTTGACCCGTTTTCTTTTAGCTAGTTTTAGTTGACCCGTTAAAGATAACATAGAGCCCGAACTAATATCTCCGTATCTCTCAGATTGTGGAGCTATGCAACCAAAAGGATGAGGATAAGAAAAAAGTGTTTTCGATCATTGATAAATTTGCCGAACGTGGTCTACGGTCTCTTGCTGTTTGCCAACAAGTGAGTTTCTACATTTCTACATATCAACATTTCCAGTTATCCAAATTTTCATTTTTTTTTTGGTGTTTATTACTTTATTAATTATTATACAGGCGGTACCAGAGAAAACAAAGGAGAGCCCGGGAGGCCCGTGGGTGTTCGTGGGCTTACTGCCACTATTTGACCCACCAAGGCATGACAGTGCGGAAACCATTCGACGGGCCCTGCACCTTGGAGTCAATGTCAAGATGATCACTGGTGATCAGTTAGCCATTGGGAAAGAAACAGGTCGCAGGCTCGGTATGGGAACAAATATGTATCCCTCTTCTTCACTTCTTGGCCAAAGCAAAGATTCTTCTATTGCGGATATACCGATAGAGGAACTAATCGAGAAGGCTGACGGTTTTGCTGGAGTCTTCCCCGGTAAACAGTTTTCATTCTTGTTATCAATGACGTTTTTACTGGGGTGTACAAATGGCGGTTTGGTTTTACCTTTAACCATAACTGTAACCGCTAGCTTCGGTTAGGTTAATCGGTTACCAGTTATGGTTTGGTTTCGGTTAATTTTGGTTAATAAACCGATCTATGTTTGAACGAAAATTCAAACTAAAAAAACAATTATTGTCTCGGTTACAGTCGTTTTTATAAATATATATAAAAAAAAAATAAGTACACATGCATAAAACGTGTCAAGTTTTATACAATAATTATTTTTTAAACATTTTAAAACTAATTATATAACATAAAAAGTTTTCGTATATGTTCTTTATGTATATACTGATTCCTTGAACATGTACATTTTAAGTATAGATATATTTTAAAAGCATGTCTTAATCCATTTCGGTTCGGTTAATGTTCGGTTAATCAAATTGCATTATCCATAACCGTTGGCTAATATCGGTTATTGGTTAATTCAGTTTAGTGGTGTTATCGGTTAATTTCGGTTAACGGTTAAGGTTTGCTGACCCCTAGTTTTAGTAAGTAAACGCTGAAACTTTTGCTTGCCACTAATCGAATGCAGAACACAAATACGAGATTGTGAGGAAACTACAAGAGCGAAAGCATATATGCGGAATGACAGGAGATGGTGTGAACGATGCACCCGCATTGAAACGGGCCGACATTGGTATAGCGGTCGCTGATGCAACAGATGCAGCTCGTGGTGCATCCGATATAGTTTTAACTGAGCCTGGGCTCAGTGTGATTGTAAGCGCGGTTTTGACCAGCAGGGCCATCTTTCAGAGGATGAAAAATTACACCATTTATGCAGTTTCGATCACCATCCGTATCGTGATCGGGTTCATGCTGATTGCGCTTATCTGGCAATTCGATTTCTCGCCGTTTATGGTTTTGATTATTGCAATCCTCAATGATGGAACCATTATGACTATTTCTAAAGATAAAGTCAAACCTTCACCTCTGCCGGATTCATGGAAATTGAAAGAAATCTTTGCAACTGGTATTGTTCTTGGGACCTATTTGGCGGTTACGACCGTTATTTTCTTCTGGTTGGCGAAAGAATCCGACTTCTTTACTGTAAGTTAAACTTGTTTATCATATTTGACACATTTAAGGTAAAATATAACCCAAATCGACCCACTTGAGATGAAACACAACCCAAACTGACTCATTAATAAGTAAATGGCAGGTTGAGCGGGTTAACTAACGGGTCAAATGGGTCAAAATGGCCCAGGTTGACCTGTTTGACCCCTTTAAATAAAACATACCCCAAATCGACCCGTGCATATGTAAATGGGTCAAAATATGTGTACCGTTGAGTTGTAATTTACGATATACCATAATGTTCGTTTTTTAGGAGAAATTTGGCGTGAAACCGATCAAAGACGAAGAATTTGAGCTTATGTCAGCTCTTTACCTTCAAGTCAGCATCATCAGTCAGGCACTCATTTTCGTTACAAGATCACGAAGCTGGTCGTTCGTTGAACGCCCCGGTCTTTTACTCCTCACCGCCTTTTTTATAGCTCAGCTGGTGAGTTTATTTATATTTATATAACACCGAAAGTAAATGTGACGCGTTTAATCTTATGAAAGTTCATTTATTATGAAGATTGCTACTTTAATTGCTGTCTACGCGAATTGGGATTTCGCAAGAGTCCATGGAATCGGCTGGGGTTGGGCCGGTGTGATTTGGCTTTATAGCATAATCATCTATTTCCCGTTAGACATTTTCAAATTCATCATCCGTTACAGTTTAAGCGGCAAGGCTTGGGACAGTATGATCGAAAAGAGGGTAAGCCCGTTTGTTAAATGTTAATCATATTCATTTACACTAACCAACATTTATACATTATATTAATTAGTATTACTAACACAATTTTTTTTTAATCCAGACCGCTTTCACCTCAAAGAAGGACTATGGGCGCGGAGAGAGGGAAGCCCAATGGGCCACGCACCAACGCACCTTGCATGGTCTCCAAGCACCAAATGCAAACGATATCTTGAACGACAAGAGCGACTATAGGGAGTTGTCGGAACTAGCAGAACAAGCAAAGAGGCGGGCCGAGGTTGCTAGGTAAGTATTGTTTCTTAACACTAGACTTAACGGGCTAAATTTGTTCAAAAACGACCAAGCACACCAAACACGCTTAAACTTAACTCAAACGTCAAACAAAAGTTCTTAATCCACAGTATAGGATTATTTTATAAATCATATAACCCTTCTAAAGTTCAAAATTCATATACATTTTTAGTTATCTTTGCTTATTAAATGAATTTTACTATTATAAGTAACTAATTTCATGAATTTTTGTACAAAAAAAGGTTATTGTTAGTGTTTTAAAAGGATAAATATGAAATTAAGATACATACAAAGATATATATGAAGTTTTGAACTTATGATATGTATGAAATTATACATACTTGATACATTTATCACTAAAAATTTAAAAAGTGCTTAGACAAAAAGTGTTAGCGGGTCAATCCAACCCATTACAGAGGCGTTACCATGTCTCATATCCGCGTTAAGCTTGCAGGTTAAGGGAGCTCCACACACTTAAAGGGCATGTTGAGTCAGTGGTGAAGCTCAAGGGCCTCGATATTGAAACGATCCAACAACACTACACCGTTTAA

# >HHA7

ATGGCAGAGAGTGAGGGCATCACATGGGATGATATCATGAAGGAGACTGTAGATCTTGTATGATTTTTATATAATTTTATTATTTTTTTTTTAAAAAAAGGCATTTTTTCTTTTTTTTTTATATATATGATTTTGATTACAGGAGCATATGCCAATGGATGAGTTATTTGATCAATTGAAATGCACAAAAGAGGGATTGACAAGTGAAGAAGGCAAAAGAAGGCTTGGAATATTTGGTCCAAATAAATTGGAGGAGAAAAAGGTTATTTCATTTTTCTCAATTTAATTTATACTTTCATAAAACTATCTCCGACGTTAACAAGGTGGAAGGCGCCCGTGGCTTGTAAAAGAGAGGAGGGCAGACGGGCCGGGTTCTAATGTGGCGAGAGAGGGAGACCGCCATAAGGAGGGGTCGGTCATCAGACGTGGGACCGGGCGCCTGGGCAGCCCGCGCGATGGAGATGGTCTAATTTTAATGTTTTTTTTTTTAATTATGTCACTTTCATGCATGCAGGAAAGCAAATTTCTCAAGTTTTTAGGTTTTATGTGGAATCCTCTTTCATGGGTTATGGAAGCTGCTGCTATCATGGCCATTGCCTTAGCAAATGGAGGGGTCAATCTCTCTCTCTCTCTCTCTCTATATATATATATATAGGGAGAGAATCATGAGAAAACTACATCTAAATGAGAAAACTCGAAAACTAACTAAAAAAAGCTAAAAACCATACCATTTTTTTTACAATTTTTGTATATATAAAAAAATTAAAAAAAAAAATTTGTTGTACTGTACATGTGCATTATATGTGTACTACACATGTGCACTATATCCGTAATAGTGCACATGTGCAATACAACAAAAAAAAATTTAATTTTTTTTTTTTTTGAAATTTTTTTCCATGCATAAAATATAGCGATTTTTTTTTATAAAAAATGTAAAAAAAATGGTATTTTTTTTTGGCTTTTTTAGTTAGTTTTTTGGTTTTCTTATTTAAACTAGTTTTCTGATGATCCATCCCCTATATATATACACTTGTAATTATACAAACGCATATACCATCGTCTAAAATACATGTCTCACGTACCCGAAACTTCGTACTAAAACCACTTAATACATAAACCGGGCTCTATGTTGATTTCTAAATTTGTTTATATATTATGAATATATTGCAGGGGAAGCCACCAGATTGGCAAGATTTTGTAGGAATAACAACATTGTTATTAATCAATTCAACCATAAGTTTCATAGAAGAAAACAATGCAGGAAATGCTGCAGCAGCATTAATGGCAGGTTTAGCTCCTAAAACAAAGCTTTTAAGGGATGGAAAATGGGCCGAAGCCGAAGCTGAGTTTCTCGTACCCGGAGATATCATTAGCATTAAGCTCGGTGATATTGTACCCGCTGATGCACGTCTCCTAGAAGGGGACCCCCTCAAGATTGACCAGGCTGCGTTGACCGGTGAGTCATTGCCGGTAACTAAGAAACCTGGGAATAGTGTTTTTTCGGGTTCGACGTGTAAACAAGGTGAGATCGAAGCTGTTGTGATCGCTACTGGGGTCCACACTTTCTTTGGTAAGGCGGCTCACTTAGTCGATAGCACAAATCAAGTCGGCCACTTCCAAAAGGTACTAGTCAAAATGGATCGGGTCAGTTTGACCTAGAAATTTTATTTAGATACTTTGTACATCAAATATGATTACATAATTAAAAGTAATTAGTGCGCCAAATTTGCTTCCGAAACTAGGGGTGTTCAGAATTCGATTCGAATTCGAAAAATTCGAAATTCGACTCAATTCGATTCGATTATCAAGTATTCGATTCGATTATCAAGAACTCGATTCGATAATTTGAATTCGAGTCCAGTAAATCAAATACGAATTTATAATGATAAATTCGATTCGAAATTCAAATTATACATATTTATTTATTATATTTATATATAATTCACACAAATAATTTTAATTTGGGTTATGGTATAAAATTTTATATAAATTTGATCTACGTCCCAAAATAGTTCATTACCAAGCCCAAATAGCCCATAACGAATTTGCATATGTTAAATGAATTTGAATTAAAACGAATTTATCCGAGTTTGAATTCTTAATCGAATACGAGTTGGTGTTTCAATTCAAATACGAATTGACCACTTTCTAATCCAATTCGAATTCAAGCAAAAAAAAAATTATTAAAAAAATCCGATTCGAGTAATTCGAAAATTTGTTATTCGATTTGATAAACACCCCTATCCGAAACATATTATTTCAATAGCCATTTAACTTTTGAAAAACCGATTAAGAGATTCAAAGCATTCAAAATACACTTAGGATGACTTTCGACATGTTCAACCCATTTGCTACGTTTGATTTAAGCTATGTCAAATTTTAGAGGCTGTTTGGGAACATCTGAATGGTTAAGTACTGAACCAGTAAGAGGTCTGAACCATTAAGAGCCTGTATAATGCTTAACCGTTCAGAGGCAAATGTCTGAACCATTCAGACATCTACTCGTGAAACAAACAGTCTAAACCATTAAGTGTTGAACCAGTAAGAGGTCTGAACCATTAAGAGGCTCATTAAGAGGTAAACAAACAGCCCCTTAGTGGTTTAACCCGCTAGAGATATAAGATAACCGGAACCAGTACATTCATAAGTAAACATGTCAAAATTATGCATGTGTTGATATCTTTGGTTTTTGTTTGTTAGGTATTAACGGCTATCGGTAACTTTTGTATATGCTCGATTGCTGTCGGGCTGATAATAGAAATAGTGGTGATGTACCCGATTCAGAAACGAACCTACAGAAACGGAATCGATAACTTGCTGGTTTTACTCATAGGCGGCATCCCAATCGCAATGCCAACCGTGTTGTCGGTTACAATGGCTATCGGGTCCCACAAGTTATCACAGCAGGGAGCGATTACCAAACGGATGACAGCCATTGAAGAAATGGCTGGGATGGATGTTTTGTGCAGTGACAAGACTGGCACTCTCACACTTAACAAGCTCACAGTTGATAAAAGTCTTATTGAGGTACAGCCAAAAGTCAAAAGTCAAAAGTCAAAACTGAAGCATGAAAGATTCAAGTGTTGACTTTTTTTTTTTTTTTGGTCTGTAGGTCTTTGTAAAGGATATGGATAAGGACACAGTGATTTTAATGGGAGCAAGGGCTTCAAGAGTGGAGAATCAAGATGCAATTGATGCTTGCATTGTAGGAATGCTTGCTGATCCTAAGGAGGTAATGATACATAAAAATATAATAATATTTATATTAGTATTATTAAGTTATTAACCAAAAGCGGTTAATTGTTTTGTTAACTAGTAAATTGCCCTGATCGTTGCCGCGACGTTTGGTAAAATTTTTACGTCAAAACGTAGACCAACTGAACATCATCATCATACTCAGTAAATCCCACCAATAGCAAAGCTAAGTTAGGGTCTGAGGAGGGTAAGATGTAGACAGTCTTACCTCTACCCCGTAGGAATAGAGAGGCTGCTTCCAGTGAGACCCCCGGCTCGATAGTAGTTTTGCATCAAGCCTTGGACATAAGGCACATAACACTCAGCAATTGAGACAACTGCCGATTAGTGCATGTGCCCCATCGTCTTTCGGCTATCAACGCCACCACATAATGCATGATTAACCATCCCCCTCTTTTAACGTTATTTTCACAAAATTAGTAAAATAACGTTAAAATTAGTGCACTTTCACTTTTGCCTCCCGAGCGCCCACACATATATACATTATATGCGCATACCGCATGCTTCCAGTAGACCAACTGAAAAACGTACATAAAAATAAGCACGGAAACATATTATATTGACCAGACTCATTTTTTCAGTCGTAGTATTCATATTCATATGAAGAATAGCGATATTGGTTCTTTGCCATAACGCCCATTCTGGGACGAGTTGGAAGAGGAATTCCTAATTTTTTTCCGGAAAATTTTACGTTGATTACAAAGTCGGTAGAAAGTTAAAGGGTTGTGTAAGTGTCAATGCTGAAATTTGAGGGGTAAAAGTATTAAAAAACCAAAAAAAAGACAAAAAAATAAAAAATAAAAACACTGTTCACAAACTTTGCTAATTGTATATATATTTAGGCAAGGGCGGGGATCAACGAGGTACATTTTCTACCATTTAACCCTGTGGATAAACGAACAGCGATAACATACACAGATCAAGATGGAAATTGGCATCGAGTGAGCAAAGGTGCACCCGAGCAGGTAGATTATTATTTGATTTTTTTTTTTTTTTTTTTTTTTTTTTTGCGTTTGTTTTGTTTGGTTTAGATACTAATATAATAACTTGAAAGCTATCAGATTGTGGAGCTTTGCAACCTAAAGGGTGATACTAGCAAAAGGGTTTTCGACATTATTGACAAATTTGCTGAACGGGGTCTTCGTTCTCTTGCTGTTTGTCAACAGGCGAGTTTCAATATTTCTAAAAAACGACACATTTCAACCCGACCCATTTTGACATGTGACTGATCTTGCCACCTCTATTTCTCGTATATGGCGGTTTAGTCATTTGATCTAAGAAAGTGGTTTTTGGTGGTTATACGCAGACGGTACCTGAAAAAACGAAAGAGGGTCCTGGCGGGCCATGGGTATTCGTTGGTCTCCTACCATTGTTTGACCCGCCAAGACATGACAGTGCTGAAACAATTAGGCGGGCCCTACACCTTGGTGTGAATGTGAAGATGATCACCGGTGATCAACTAGCCATTGGTAAAGAAACGGGCCGGAGGCTCGGAATGGGGACAAATATGTACCCCTCTTCATCCCTCTTGGGCCAACACAAAGACGCATCTATCGCTAATATTCCTATCGAGGAGCTAATCGAGCAAGCTGATGGCTTTGCTGGCGTTTTTCCTGGTAAGTACACGAATTTAATTTTAGAGTAAAGTACGCGGATGGTCCCTCTGGTTTACCAAATTTTGGATTTGGTCCCCAGCTTTTCAAAAGTACACGGATGGTCCATATGGTTTGCAGTTTGTAACACATTTAGTCCCCAACCAACAAATCTAAAGGATTCAGCAGGTCCAAGTTAGGGACTAGATGCGTTACAAAGTGCAAACCACAGGGGCCATCCATGTGACTTTTGGCAAAGTTAGGGGCTAGATTCGTTACAAAGTGCAAACCACTGGGACCATCTGTGACTTATAGAAAGCTAGGGACCAAATCCAAATTTTGGTAAACCACAAGAACCATTCGTGTACTTAACTCTTAATTTATTAATATTTTAAATGATTTAGAACTAAAATTATAGAAGGGGTTGAGAAATGCAGAGCACAAGTATGAAATCGTGAAGAAGTTACAAGAGAGAAAACATATATGCGGAATGACAGGAGACGGTGTAAACGACGCACCAGCACTAAAACGGGCCGATATCGGTATCGCGGTTGCTGATGCAACCGATGCAGCCCGAGGTGCATCCGACATAGTCTTGACTGAGCCTGGGCTAAGCGTGATTGTAAGCGCGGTACTTACTAGCCGAGCCATCTTTCAGAGGATGAAAAACTACACCATATATGCTGTCTCCATCACCATTCGTGTCGTTCTTGGTTTCATGCTACTCGCACTGATCTGGAAGTTTGATTTCTCGCCTTTCATGGTTCTCATTATTGCAATTCTTAATGACGGAACCATCATGACCATATCGAAGGATAAAGTCAAGCCTTCACCGATGCCGGATTCATGGAAATTGAAGGAAATATTTCTCACTGGGATCGTTTTCGGGACTTATTTAGCGGTCATGACCGTAATTTTCTTCTGGCTAGCACAAGACTCTGACTTCTTTCCAGTAAGATAAACTGTAAAATTATATACTTTATGACATGGTTGTAAAAATCACGCCTAGCCTCCGATTAATCCCTAGTCGGAGTTTCACCGAGAAATTTTCTTCTGATCGGCCGATTAATTGCTAGGTGATCAACAGTGGTTAAATCCAGTCCTAATCTGCCAAAAATCGGTCACAGTCTAGCGATTCTGGCCAAATTCCGGCCGAAACCTGTAATTTCCGGCCAGATTGCGGCCAAAACTTGTAAATTTTGGCAATTAATCCTATGTACTTCATTAAAACATGTCTACTTAAAAAGTTACATATAGAAATATGTGTATATACATATAAAACTGAAAATTACATACATAATCTCAATCCGATTAATCCCGACTAATCGCTAGTCGGAATCCCACAGGCCGTCTAGCACCTGGCAGTTCTTACAACACCGCTTAACGATGATGAATGAATTGTTGTGATTTTTCTAAATTTGATAGTGATTGTAACACTGTATAGTGGGGTTTGGTCACAGGATAAATTCGGTGTAAGATCGATTAGAAACAAGGACTACGAGCTTACGGCAGCTTTGTACCTCCAAGTCAGCATTGTTAGTCAAGCGCTTATTTTCGTCACTAGATCAAGAAGTTGGTCGTATGTGGAACGACCCGGTCTTCTGCTTCTGACAGCCTTTTTGATCGCACAGCTGGTAAGTTTTTTGTTACACATTCTAAAGTAAACTTCCGTTTTGCTCCCTGTGGTTTGGTCACTTTAACGGTTTTGCTCCAAACCTTTAAAAATAGCCATTTCACTCCCTGATGTTTCGGTTTTTTTGCCAGTTTGCTCCCCGCCTCTAACTCCATCCAATTTGTTTGTTTTTCCATTTTGCTCCCCGCAGGGAGCAAACTGGCAACAAAACCAAAACATCAGGGAGTAAAATGATTCTTTTTAAAGGTTTGGGACAAAACCGTTAAAGTGACCAAACCACAGGGAGCAAAACGGAAGTTTACTCTTTATAAAATGTTGTTTTGTATTCTAAGTTTGTTATTCTCATGTTTAATGAAGATAGCTACCCTGATCGCGGTCTATGCACACTGGGATTTTGCGAGAATCAACGGAATAGGTTGGGGATGGGCCGGTGTGATTTGGTTATATAGTATAATCTTCTACATCCCGCTAGATATTTTCAAATTCATCATACGGTATGCAATGGCTGGCAGGGCTTGGGATAATCTGCTCCAAAATAAGGTACAATAAATCTTTTCGGTTAGGTTATAAGTAGGGGTGCAAACGAGCCGAGACTTATCGAGCCCGAGCTCTAGCCTGACGTGTGAAGGCTTGTCAGGCTCGTCGAGCCTTCGTATAATATTGTAATATTAGTTTTTATTAATATTTAATAAAATTTATCCCTTATTTGAAGTTGTCTAGTAAATGAGCCGAGCCGAGCCGAGGTTATTTAAGCTTGTTCACAAGCTGAGCCCGAACCTAAAAATAAGCGTGTTTAGTAAATGAGCCCGAGCTCGAGCTTCACTTATCGAGCTCGCAAGCCTAAACGAGTCTATTATTTATATTATTTTTATTTAATACATTAATTAATTGATAATAAACGAGCCGAGCCGAGCCGAGCCGAGCCTGAGCCGAGCTCGAGCTTGAGAAACTACCAACGAGCCGAGCTCGAGCTTTGAAAACAAAGCTCGAACTGAGCCAAGCTCGAGCTCTCATAAGTTAATCGTCGAGCTCGGGCTCGGCTCGGCTCGTTTGCACCCCTAGTTATAAGGATTGTTAACTTTCTTAGTTGTTGACTGAATGAATTTTTCTTTGTTCTGATCTTATGAGAAACTAGACCGCTTTCACTAGCAAAAAAGACTACGGGCGGGGCGAAAGGGAGGCCCAATGGGTCCAGGAACAACGGACGGTCCACGGTCTCCAGCCGCCAGAGCAGCCAGAACAGTTCGTGAACGACAAGACGAGCTACCGGGAGTTGTCGGAGCTTGCCGAACAGGCTAAGAAGCGCGCTGAAGTGGCTAGGTGGTTTCCAAAACAAATAATTCATCATTATTTGTATTTTTAATTTCTTATTATTATATCTCTTCTTACAGATTGAGGGAGCTTCATACAATAAAGGGTCACGTTGAATCGGTAGTGAAGCTTAAGGGTCTCGATATCGATACCATTCAACAGCACTATACGGTGTAA

# >HHA8

ATGTCTGATAATTCCTTGGAAGAAGTTAAAAGTAACCAAATTGATCTCGAAAAAATCCCTATAGAAGATGTCTTCACGATCCTAAATTGTACGCGAGATGGCTTGAACGACGAGGAAGCCACTAAGAGGCTCGACATCTTCGGTCACAATAAGCTTGAGGAGAAACAGGCACGTTCTTTTTTTATTTATAATCATTAAGCTTCACCCTCATCAGATTTTTTTTTTTTTTTTAAATGAGCTTTTGTTTACCATAGTCTGAACCCCTTGGTGGTATGTAACACATACCAAAAAGTTCTTATTCAATTAAAAACACACAATAAATATCATATATATTCTATTGGGGTGTAAACGAGCCAGCCAGACTGAGCCTGAGCCCGACTAGGCTCGAGCTAGGCTCGTTATGTTTTTATGAAGCTCAAGCTCGAGTTTGGCTCGTGAGTAAAACCCAAAGCTCAAGCTCGGCTCGACTTGGCTCGAGTTATTTTGAGAACAACCTCAAACGAGCTAAATGCTCGGCTCGAGCTCGCTTCAATATCGCTTAACGAACCAAAGCTCAACACGAGCTCGGCTCGCCAATATTTGTTTGGGCTCGTTTACGCTCGTGAGCCTAAACGAGCTTTGTATTGAGGCTCGAGCTCGGGCTCGATAACTAAACGATCTCTATTTCAGGCTCGAGCTCGGGCTTGATAACTAAACGAGCTCTATTTCAGGCTCGAGCTCGGGCTCGTTCGAGCTTTTGGCCGAGCTGATCACGGGTAGCTCTCGAGCCTCTGGGCTTGTTTACACCGCTGTATAATTCTAACCACCTACTATTATTAACTATGTTGAGTTATGTTTGAAACAGGAAAGTAAATTGCTGAAGTTTCTAGGATTCATGTGGAATCCTCTTTCATGGGTCATGGAATTTGCAGCCATTATGGCCATTGTATTAGCCAACGGTGGCGGGAGGCCACCGGACTGGCCGGATTTCGTGGGCATAGTCGTGCTTCTTCTCATCAACTCAACCATTAGTTTCATCGAAGAAAACAATGCCGGCAATGCCGCCAGTGCCCTGATGGCAAGCCTAGCCCCGAAAGCCAAGATTTTAAGAAACGGAAAATGGAGCGAGCAAGATGCCGGCATATTGGTTCCTGGAGATGTTATTAGTGTCAAGTTAGGAGATATTATCCCAGCTGACGCTCGTCTCCTTGAAGGCGATACGTTGAAAATTGACCAGTCTGCGCTTACTGGCGAGTCGGTCCCAGTAAACAAGAACCCGGGTGAGCCGGTTTACTCTGGCTCGACGTGCAAGCAAGGCGAGATTGAAGCTGTGGTGATAGCCACGGGCGTCCGAACCTTCTTTGGGAAGGCTGCACACCTTGTGAACAGCACGGATTCAGCGGGTCATTTCCAACAGGTGTTGACATCCATTGGTAACTTTTGCATATGCTCAATTGCGATTGGAATGGTGATCGAGATTGTGGTGATATGGGTGATTCAAGGAAGAGGGTATAGAGACGGTATCGATAATTTGTTGATCCTTTTGATCGGAGGTATCCCGATCGCCATGCCTACAGTTTTGTCTGTAACGATGGCGATCGGGTCCCACCACCTTGCAAAACAAGGTGCCATCACGAAAAGGATGACCGCCATTGAAGAAATGGCGGGTATGGATATTCTTTGTAGCGATAAAACCGGTACCCTCACTCTTAACAAGCTAACGGTCGACAAATCGTTGATTGAGGTCAGATTTCAAATTTTCAAAATTTTCCTTGTCAAAAAATGTACGGTCAACCATTTTCGGTTATCTAATCGGTCAAAATTTGTTACAGGTTTTCGTCAAGGATTGCGATAGGGACATGGTGGTGATGTATGGTGCTCGAGCTTCAAGGATCGAAAACCAAGACGCCATCGATGCTTGCATCGTCAACATGTTGGCTGATCCAAAAGAGGCTCGAGCCGGAATCAAAGAGGTCCACTTCCTACCGTTTAACCCTGTCGACAAACGCACAGCCATCACCTACATTGACAATAAGGGTGACTGGTATCGGTCCAGCAAGGGTGCACCAGAACAAGTAATGAACCACTTGTTTTCTAATGTTTTCTTTCCAATTCATAGTTAATGGCGAATCGCGACAAATAGCGATAAGGTATATGCTACATAGCAAAAACCGATAAATAGTGGGCGCTATTTTATAAATAGCGATACACTAGAAAAAATCTTGAAAAAAAAATTATATTTATATTATATCAAAATACCCTGGTATATACACTATTTTAAATGTATATTTAACAAAAATCTAAAATCCAGCTATTTTATAGCTATATTTAATTGCTAATTATATTTAAAAAAAACTGAATTCCCGCTATTTATGGCTACATACCCTCTACCGACCTTCGACCTATACGCTACGCTATTCGCTATAGCGACCGCTATTGACAACTATGTTCCAATTACAAGGTTTTATAGCCGATAAGTGATAACGATTAAATTTCTTGATAGATCATCGAACTTTGTAATCTCACCGGCGATACACTAAAGAGGGCTGAGGAAATCATTGACGGGTTCGCAAACCGAGGTCTTCGGTCTCTCGGCGTGGCTCGACAAACCGTACCCGAGAAGACAAAGGAGAGCGAGGGCTCACCTTGGGAGTTTGTAGGACTGTTGCCGCTTTTCGATCCACCACGACACGATAGTGCCGAGACTATTAGAAGGGCTTTAGAGCTTGGTGTTAAAGTAAAAATGATCACCGGAGATCAACTCGCCATTGGGAAAGAAACCGGCAGAAGACTTGGTATGGGTACCGACATGTATCCGTCATCTTCGCTTCTCTCGGAAAGCAATGATGCTAACAACTCTTCGATCGATGACCTCATTGAGAAGGCCGATGGCTTCGCCGGAGTTTTTCCCGGTAAATATTACTAGACTCAACTACCATTAGGGCTTCAAACGAACCGAACTAATTCGTTTGTTAAGAAATATATGTGTTCACGAACTGTTCATGAACACTTACGGAACGAGATTTTATGTTTGTGTTCGTTTGTTAAGGAAATGAAGTGTTCGTGTTCGTTTGTTAATTTTAGGCAACAAACGAAAACGAACAGTGAACGAACGAAAACGAACAGTGATGAACACAAACTAATGTTCATGAACAGAAATGGAAACAAACGAACATAAACAAACGTTCATGAACAGAATATATAATATACTAACGCTTATTAAATATTTTATTTGTCGGAATTTTGAAGTATTTAAATAAAATATAAAAACTAACCACACTAATGAACTATCGAACACAATCGAACATAAACGAACACGTTATCGAACCTTCACGAACATAAATGAACGAACGCGACCTGGGTTCATGTTCGTTCATTTAACTAAACAAACGAAATTCATGTTCGTTTGTTTAATAAACGAACAAACACAAACGAACTTCCCGCCGAACAGTTCACGAACTGTTCGCTGAACGTGTGGTTCGTTTGCAGTCCTAACTACCATCATAGACAACGTTTACAAACCTTAATTTTCATTCTTGTAGAACACAAATACGAAATCGTCAAGAGGTTACAACAGAGGAATCACATATGCGGTATGACGGGAGATGGTGTGAACGATGCACCAGCACTCAAGCGAGCAGACATCGGTATAGCAGTGGATGACTCCACAGACGCTGCCAGGAGTGCGTCCGACATTATCTTGACCCAGCCGGGGCTTAACGTGATAGTCGCAGCTATACTAACAAGCCGAGCCATCTTCCAAAGAATGAAAAACTACACAATATACGCAGTCTCCATCACCATCCGAATTGTGATGGGGTTCATGCTCATTGTTGTCATATGGAGGTTTGACTTCTCGCCTTTCATGATTCTCGTCATCGCTATTCTTAACGATAGCACCATCATGATGATCTCCACTGACCGGGTTAAACCGTCACCACTTCCCGACTCATGGAAGCTCAATGAGATTTTCGCCACTGGTATCGTCCTCGGGACTTATCTATCACTCGTTACTGTTTTGTTCTTTTGGCTATCCTCCAGAACCGACTTCTTCCCCGTAAGTCTACGACATTTTATCTTCTGTTCTTCATTCATTATACAAAGTCTTTTTAGAGAAATGAACATGACAAGAAATAACTATTATGTTCAGAGGCTTTTTGGTGTTCGATCGATTGTTGGAAACGATGATGAGGTGACCGCAGCGCTATACCTTCAAGTGAGCATCATTAGTCAAGCACTCATATTTGTGACGAGATCTCAAAGTTGGTCATTCCTCGAGCGCCCTAGCACCTTGCTCATGCTCGCATTCGTACTAGCTCAGATAGTAAGTACCAGCTACCATAACATCTCTTTGATATGACCAGGGGTGTTCATCAGTTCGGTTTTGGTTTATTCGGTTTTCAAAATTTCGTACCCCAAACCAAAAACCAAACCGAATTAGAAATATGCAAGCCTGACCAAATTCAGCTGAATTCAATTCGGTTTGGTTTCGGTTAAAAACCAAACCGAACACCGGCTATTCGGATCGGTTTTTTCGGTTACGGTTCGGTTTACCGTTTTGATCCGGTTTCGGTTTTTAACACCCCTAGATATGAGTTGTGTTTATAAGTTATAACTGTTTAACATTTGTGTGTTTTATACAGTTGGCTACACTTCTAGCAGTATATGCAGACTGGGATTTTGCAGAGATGCAAGGGATCGGATGGCGATGGGCCGGGGTAATCTGGATGTTCAGTATCGTTACCTACATCCCTCTAGATATACTAAAATTCATTATCCATGCGGCATTGAACGGGAACAACTCTAGATAG

# >HHA9

ATGCATTTCTATTTTGATGTGCATATGGAAAACAAGTCTAGAAAGTATTGTTGTTACTTTGATTAATTTTTTCAATTTTCTAGTAAATTCTTCATGATCTCAAATATAATTGAGCAACTATAGAATCTTTGATCCCATTTCATGTCAAAACATCTCAATCTTTACTAGCTTGAGTCAACCCTCCCCCCCCCCCCCCCCCTTGTTATCACGAAGCTGTCTTGTGAGGTCCCGTGCGACGTCATTGGGAGCCTGAGGACCAGCATATCTGTCTTGCTTATGATCATGGGTCAGTGGTTTGCGCAACTCCAAACACGTGGGAATCAAGTAGTGCAAGCACTTCTAGGGTACATAGTAACCGTGCAAAGACTCATCAGGGAGTGACCCTAACTCTCAACCAGAAGGACCCTAGAAGGGTCAAAACTTACGGGTACACTTTCATTAATCGTTGAGATACTCCCTAAAGATATACAGTAGTCAGAACCCCACATGGTTAAGTTCAAAATCAAGTAGATATAATCTTCCAACATTTAGCATGTAACATTTGCAAACTAGTCTACTAGGAGGGAGACGGTGTTCAATGTCTGGTTTCAAGCTGATTAAACCAAGAACCAAACTGAATCAAAATGATTTAAACCAAAACCAGGTATATATTTAATTTTGTATAGTTTTAAACTTAAACCAAATTAGGTAATCGATTTGCGGTTCTAGAAAACCCAATCCGGTTAACCAAGGAACATGAATTACATATGTTGGGGTATTGATTTTTCTATTTGTACATCATATGACCTATTACAAAACTGAGGAAACTGAACTCCAAACCAATAAAAACTCATCTAGTTAAAACTTTTTTTTTTAACAGGAACCAAACCAAAATAGAATTCGGTTTCAGTTCAATTTTGCCTACTTTGAATTCGTTCGGTTTGCTTCTCAATTTAGACTACAAAAATTTAAACCAAAAAAACCCAATCCGTTTAGCACCACCAAGTCTCAGCTTTAATTTTTTACTTCTAAACTAGGTTTAAAGAAAAGTTCTGTGTTGCGGCGAGTTTCTTTTGTTATTTAGTATGTGTTTACATGTGTTTTGAAATCACTACGAGCGTGTTGCGAACGGAACTGCTGACAATAATAAAAAGAAAATGTATAAAAAAAAAACACTAAAAGATAACCCAAAGAAAATCAACCAAAAAAGTTGTATGGTAACGATGTTGTTCGTATGAAACACGTGGTCAAAGATGGGCAAATTGTTTTGGGTACTGGTACCAAATTTGCCGAACCGAAAGATCTTAAGTACCAATTTGGTACCGATTTTTGGCGTTCCTGGTACCGGTTCACAACCGTTTTTTACCTTCATATACCGGTACCGTAACTGGTGTTCTGTATCGGTTGGCACCGAGCTCATCCCTACCCCTGAAACTAAAAACGAAAAAAAATAAAGTTAAAGAAAATCAACAAAAAAAGTTGTACGATACTGTTGTTCGTACGGTAACCGTGATCAGGGATGAGCAAATGGTATCGGGTAGCAACATTGAATTTCCGGAACCAAAAGATTTCCAATATGTACTGATACCGAACAGGACCGTCCGGTATTTTCAATACCAGTACTGGTTCGATACCGGTTGATACTAAGATTCTCCCAACCCCTGATATTAAATAAAGGATTAAAGTTTAAAAGTAAAGAAAATATCTATATATTATAATATTAAAATAGTAAAAATATAAAAAAAAACTATATATTATTATAATATTAAAATCACTATTCATTTCAATTCGTTTAGTAATATAATAATAATAATAATAATAATAATAATAATAATAATTTGTAAATGACTAAGAATCTTATGAATCTACTCAAATATGTTTTTACAATACACCATATTCAAAATTATTTGATTTATTCAATGACATTTTTATCGTAATATAACTTCAGATGTGACAAAATATTTAGGTATTGTCCGTTTCATGGATCGTAAGGGAATTGGAATAGGAATTCGCGCAGAAATTTAAATTTAATTTGTAATTTAGGTGTGTTTGTTTTGCAAAATGGAAAAATTTGGGACAATCCCATATTTCCTCTTAAAAAACTTCTTAAGTGCGTATTTACCCAACTTAAAATTAAAATTGCATATATCCCCTTCTTTAACTAATTAAATTGCAAATTTATCTATTTTGATTTATTCATTAAAAAAGTCATATAATGACTTTTTTACCTTTATTTTTACTGAAACTTTAAAGAACTACATATTTATTCAGTTTTACTAGTTTTTGTGGTTTTTTCACAATTCTTTTTGTTTTTAATTTTTTACGTTTTGCTAGTTAGTTTTTTTGTTGTTGTTTTTTTTTCTTTCTACTAAGTTATTTATTTTACTAAGATATATCTAGGGGTGTTTGTGATTTTCAGCTGATTTATAAATTATATACATTAAAATACGTTTAATGATTAAAAGAAAAAACACAAAACAAATAACTTAGTAAAAAACTAACTAGAATGATTTTCACCGGATTTATAAATATACTAGGTTATAGCCCGCGTGTACACGCGGTTTAACATATGAAGATACTTTGAATTTGTTCAGATTTAATGAAAACTAAATGAAAATAAATTCAATATTAATAATATAAACTTAAAATTGTATTTGTTCAATACACTTCTCAAACTTATTATATATTGAATAAATCCACAACTGAAGTGTAATCACTTGGGCCTCACCTTCATTGACAGTGCCATGATTTGTTTGTAACTTGTAATACACATTAGCAGTACCAGCAGTTCCAGGACCAAAATCATACTATCTCCATTGTATGAGCCAATCTGAAAAAAAAAAACAGATATAAAAAATCAAAAAATAAAAAAACACCAAGTCCCATTTCATATCTTAAATTATCTTGAACAGAAGTGAAGCCATCAAATAGCATCACTTGCCTTTAGTACATTCCTTCTATGATACTTGAATAGTTAAAGAATATGGTTTTAATTTCACTAAGGGAAGTATACTGGTAATGTCCAATTAAGATTCAAAATACAATCTGATTCAAACAAAGAAGCTCCAAAACCTTATACGCCCAGTTTTAGATGCACGGGTCAGAATTGCCAGGCCAAGGCAGCATTGAGTTATGGGTCGAAAGTCAAAACGGACAGTTGTTTTACGAACCAGCCCATATGGGTTTCGGATCGGGAATGCTACTAACAAATCTTAACAGCATTACAGTTCTAAAATCATGATTATGGTCATTAAATTCCACTATAATTACCTCACAATTTCCATCAAAACAACTAATCAAAATACAAATCACTCTTTCTTTAACCTAATATCACAGAAGACTTCGATTCTTAGTTGATTTGTGTACCTAGTTCCCCATTATCATTTCACTATGTACCTAGTTCCCCATTTTACTGATCAACATACTCTATTCCCCACCAACAATTTCTTTACTTCTGTTATGCTTCACCACCACTTGAAATTAAAAAAAAAAAAAAAACTGATAGAACTAACCTTGAAGAAGCCCACTGGTCCACCGTCTTCCGCTATATCATCATCTCTAACACCATCAATCGAAACAGAAATAAACAGAAAAACACTAAGAGATTACAAAGTCAAAGTCGTATGTTCTTTGTTATATAAATACTATACATCTAACAAATAATCAATTCTTAGTTCTTTGTTTACAGAAAAAACTTATGACATATCGGCTTTCCGAAAGTAACTCACTTCAATCGATTAACTTAAAAAATCAGATTAGGTCATTCAACTATTCAAATTGTAGTAAATCCTAAATTGTTTAAAAAATTAAAACTACACTGTGCATCATAACAATTACTTTGTAAACAATTAAAACACGTTGATCAAAATAATACTATCATTTGTTACAAACTACAGTCAAATAACACAAATTAACTATCAATCAATAACAGATACAAATGAACAGAACAATAAACGAATTATAAAACAACTTACAACACGATTTCTTAGCTTCGAAAGAAGCGAAACGCTCCGATAAAACACCATCGCTGATAAACAAAACAAACTGCAATGTAGAATCAACAAAACAATCGTCAAATAGGCGCAAAAACGCAGATCTGGAGACTGAAAATAATAAAATTGAAATTGATGTTGTGATGATGATTGATTGATTAGAGTTGTTGTACCTTGACTCGGAAAGGAAATGAGAGGAGAATACTGGAAAGAAGGAGAGATGGAATGGTGATGCGTTGTTGAACTTGAATATGTAAACGAATAAAAACAAATTTAGGGTTTCTTGGCTGAAAGGAAAAAGGAAATAAGGTGCAGGGAAATAATGAATCCAAAAGTTGATAGTGAGAGGACAGGGTAATGAGTTTCTTGGGATGAGTAAATGGAATTCAAATTCAAAAATTGAATCCACATAATCTGGCCGCCCAAATTTCAGCATATTTGAAAGAATTAATAATACACAAAAATTGAAAAATCTCACAAAATTACACCTAGGATAGGATGATGTGGACTGGCTAGAATTTTGACATGTGTATTGGTTGAATGTGGGGGTGCCAAGTGGCAGAACCTTGTTCTTTTATTAGGTATAGAGATACATTATTATACGTTTAAAGCAAGTTACATTCTTTTCTTTGGTTTGTAGTCCTCTAATTAAGCTCCCACTTATTTAGAAATTATTTATATTCTATTGAAAAATATGCATCTACGTACTTTAGCGGTTTATTTCAGTGTTTAGATTTTAAAAAACACAACAAACGAAAACTAACAAGCAAAAGGTAAAAAAAAATAATAAAAACAAAAATAAATTGTGAAAAAGCCACAAAAACTAGTAAAAATGAGTAAATATGTATTTTTTTTAAAGTTCCAGTAAAAATAAGGGTAAAAAGTCATTATATAACTTGTTTTTAATTAAAATAAATCAAAATGGGTAAATTTGCAATTTTATTAGTCAAGGAAAGGGGATATAATCAATTTTAATTTTAAGTTAGGTTAATATGCAATTAAGAAGTTTTTAAGGTTAAATATGCAATTGTTCCAAAAGAACTGAGATAGAATTAGAATGTAGATTCACTTCTTTATGTTGTTCAGTTTCAATTCATTTTTTTGTTGAAACCAAGAACATAAGAAGTGAAATCAAGATTTCAATTCACTCAAAATTCTATTCCATTCTTTGAAATAAACACCAACTTAGAATTCAAAGTACCTTTTTATTTTATATTTTTTCGTAAATTTCTATACAATAACTAAAATAAACGAAATCACAATATCACACCAACACCTTTAAAATACTTCATAGTTTTTAGTGACTTTAATAGAACTTTTTTATTGAGTTTAGGCCACTTTTACCACTTCAGTACAAATATAAAACCTTCACTTTTGTATCATCAGTTTATGGTTCTGAGGTTTCATTTTTGTTGCCATTTTCATCCAATGGGCAAACTCGGTTAGAATTTTCTGTTAACTTCTCCTTTTTTGTCGTTTTCCTCATCTAAATGAAGGGTATAATCGTCATTTTATGCCATAATATATTTTAATTAATTGAGGAAAACGACAAAAAAAAAAGGGGGAGAAGTTAACAGAAAATTCTAACATAGTTTGCAAATTGGATGAAAATGGCAACAAAAATGAAACCTCAGGGATCTAGATACAAAAGGTTTATTTTCAGACTAAAGTGACAAAAATGGCCTAAACTCAGGGACCATTTTGGCATTTTACTCTTATTTTTATAGATCTTTTTAGAAAACAAACCCATTTTTAATCCAAAAATAAATTATAATGAAAAGTTAAAAAAATCGAATAATTAGCATCCTCTCACGTTATATATAACCAAACATATAACGCCAGAGAAGATGTGCTAACAATATATTTGCTGAAAGTTGGAGAGGCAAATATGAAAAGTTTAAATAACTTGAGGACAAAAAGTGAAAAACCAAGTATAGTAATACCCGAAAAATATCGTTATATTCTAAAGTTGTCATATGGCGCCAAGTTAACCGGCTTAATCCGACCGGTCAGTTGCCGGTTTTGTGTTCACTTTGCCGGCTTTTAAGATTAAACAAGTACATGTACGGCCATTTGGCACACTAATACTAACAAAAGGTAAAATAAAATTAAATATATTCTATATTGTTTTTAGTTAATATACATTGTAGAAAAAAATAATAGATTAATGATTTTTAAAAAAAATGTGGGTCATGGTACCTCTTTTATTTTATTCCAGTTGTTTGACAACTTTTGAATGAGTAAGTGTTGAACAGTAAGTGGTCTAAACTATTAAGCGTTGAATCAGTAAGAGGTCTGAACCATTAGAAGCCAATTTAATACTTAATCATTTAGAGACAAATATCTAACCAATTCAGATAAGAGGTTTTAACCATTCAGACTTAGTATAATGCTTAACCATTCGGAGGCAAATGTCTGAACCATTCAGACATCTGCTCGCGAAACAGACAGTCTGAACTCTGAACCATTAAGAGCTGAATCAAACGGCCCCTAAGTACTCAAATAATTCATTTTTAAAGATTAAAAAAATTGATTAAGAATTTTTTTTAAAGTTTATTGTGAATAAATGGGATGAAAGCAAGAATGATATGTGTGTATATATAGTTGGCCTGCTTCAAAACAAACACAAACACAAAAACACATACATCTAATCATTCATTCATTCATTCATTTATTCCCTCTTGAAGTCTAGTGAACATTTTCATTAGATTTCAACCTTTCATGTTAACGAATTTGTGTTTCGTGTTCTAAATCTTCGTATCTTAAGCTTATCAGGTATCTAAATATATCTACTTCGTTGCGATTATATTAGTTGTATTGTTCTTTTATATGTTTATTTTCTGAATGACTAACAAGAATGGCAATCTTGGGCCGCAATAATATTCATTCGCTTCATGACATTGTTCTTCTCTTTGTCGTCTTGATATGATCATGTATTATAATGGGATTCAATAAATTTTAATATGTCAAGTTGCGTAAAGTTTGAATCCGAATTTGCATGTATGTTTTTATTTATAATGGCTTGATTTTTAGAAAAAAAAAAAAAAACTTGTGAGTTGGTGCTAATGACGCATCCCATGGATCGAGCTTAACGTCTAGGCCGGGCCTGGGCCTAGACAAGCTCGACCCATTTGACATGATCCAACCCACATTCAATTATTGTAGCCAAAATAGCAAGAATTCTTTTTAAAATAATAAGATTCAAGAAATTAACCGAAACATAGAGATTCAAGAATTCAGGGGCGGAACCAGAGGGGGGTCAGGAGGGTCCATTGACCCTTCGGTCACCGGAACTTTTTGAATTTTTATTTATAAATTTTGAGTTTTTGAAGAACAGACTTAGAGATGGACCCCCTAACTTGAACCAATATAGAATATAAGCTTATGATGACCCCCTAACTAAATCGTTCTGGTTCCGCCACTGCAAGAATTATTCAAGTCTTGTTCATATGGGTGCATAATAGTAGATACATTTCAAAGAATTTACAGAAAAAAAGGCAAAACTTCATTAAAAGAAATCAAAGATTCATATTGTGTTCATCTAAGCTAGTTTAAACAACAATGCAGATACTAGTGAACAATCAAAAGCCGAAAAATGGATGATAAGTCAATCGCTCTCAGTGCAGTCATCCGAGAAGCCATCGATTTGGTATCTCTGAGCTTTATCACATCGAGCAAACGTTTTTTTTCTTTTTAATAATTTTATCACGCGAAAGATGCAAACTTAATGTTTTTTTGGGTTGTAGGAGAATGCACCCGTAGAAGAAGTGTTTCAACACCTGAAATGTACAAGAGAAGGGTTAAATTCGAGTGAAGTTCAAGAGCGTTTGGATTTGTTTGGCTACAATAAACTCGAAGAAAAGAAGGTATTTATATAGAGCTCGCAAAAGTGGCGTTTTGTGCTAAAAGGGTCTTGTACAACATGACACATATAACACGACTATGAACCTGAACACACACATTTAACATGATTAAATATAAACATGCGTGAATTTCAATGATTGTCCTTTATCTTTATACCTATTTTCAGACGCTGTCCTTTATGTTTAAAATTGACGAGTTTTGTCCTTTATGTTTTCATATCATACACATTTTGTCCTTTGGGCTTAACCCAGTTAGTTTTTTTAGTTAAATTTGGTCATATGCTTTGCACATAAGGGCATTTTTGTCAATCCAAAGAAAAGTTCAACGGCAGATTTACAGCTCAAAGCTTCTGCAACCTTTGAATTGACAAAAATGCCCCATGTGCAAAGCACATGACCAAATTTAACTGAAAAAACTAATTGGGTTAGGCCTAAAGGACAAAACGTGTATAATATGAAAACATAAAGGACAAAACTTGTCAATTTTGAACATAAAGGACAGCGCCTGAAAATGGGTATAAAGATAAAGGACAATACTTGAAATTAACTCTATAAACATTTTAGTAACACACATGACACGACACTACACCTTTAGCACGATTGTTAACATGACTCACTAACACGATTAAGAAATAATACGACACAGTTAACACAATAAAATAAAAGTTACCAAAAATTATAATCCATATTTTACTATAACATATGTAATTATTTGTAAAACTACGTAGTTATGATAAACTCGTGACGTATGTGTCGTGTCAAACTATAAACTAACTTAACATAATAAGTTAAACGTGTCATAAAAGTGTTAAGCGGGTTGAACGCGTTTAGACACATTTTTAATCGTGTCATTAACGTGTCAACCCGTTGATGACCCAAACATGTTTACCCCAAACATTGAACCTGTATCGAATTATGATCATCTTAACGTCACAAATAATGTACAAATCATTGTTTTTTGAGTAAATTACAATTTTGGCCCCTGTGGTTATATCACTTTTACCCTTTTAGCCAAAAAATGAATCTTTTAACATTTAAGCCCCCAACGTCTTTTTTTCTAACCCTTTTGGCCCCTAACACTATCCCCATCCATTTACTTTAGGGGCCAAAAGGGTTAGAAAAAAAGACGTTGAAGGCTCAGATGTTAAAAAAAAAATTGGGCTAAAAGGGTAAAAGTAATATAACCACAGGGGCCAAAATCGTAATTTACTCTTGTTTTTATTGATATTTTAATAATGAATTATTGCATTTCTTTTTACAGGAAAGTAAAATCTTGAAATTTCTTGGATTCATGTGGAATCCACTCTCATGGGTGATGGAAGCAGCTGCTATCATGTCAATCGCACTTGCACGTGGAGGGGTAAATTCTCATTTTACTTTGATGTTGACTTTGACTTTGACTTTGACTAAAGTCAACTAGGGACGGAAATGGGCTGGGTTTGAATTCTCATTTTACTTTGATGTTGACTTTGACTTTGACTAAAGTCAACTAGGGACGGAAATGGGCCAGGTTTGGGACAAATACTGTACTTTATCTGGTCTATTACCTGTCGGGTATCAGGCATAACCGCACGCATTATATAATATGACTCTTGAATCTTGATTTACTTCAGGGAAAGTCTGCTGATTACCACGACTTTGTCGGGATCATCGTCTTGCTTGTTATAAACTCAACCATAAGTTTCATAGAGGAAAATAACGCGGGAAATGCAGCTGCTGCATTGATGGCCCGTTTAGCGCCAAAAGCAAAGGTTCTTCGTGATGGAAAATGGAGTGAAGAAGACGCTTCGTTATTGGTTCCAGGTGATATTATTAGTATCAAGTTAGGTGACATTGTTCCTGCTGACGCTCGTCTTTTAGAAGGCGATCCGTTAAAGATTGATCAGGTATTGTTCTGTTATACAACGGGTCAAAACGGCCTAACGGGTCAGTTTGGGTTGATAAACAAATAGTTGACTGTGTTTGTTGAATGTTTATAGTCTGCACTTACTGGAGAATCACTGCCCGTGACGAAGAATCCGGGAGATGGTGTTTACTCGGGTTCCACGTGTAAGCAAGGAGAGATCGAGGCCGTTGTGATAGCGACCGGAGTTCATACGTTTTTTGGGAAAGCGGCTCATCTTGTCGAGAACACGACTCATGTTGGACATTTTCAGAGGGTATGTGTGTCGAACATTGTTCGATTGTTAGGTTTTTGGTTACGATCAGGGGTGTTCATGGTCTGGTTTTGACGGTTTTTAAGTTAAACAGTGAGTCAAACCGTCAGTAACGGAATTTGACTTTAAGTAAATTAAAACCAAATTGTTGAAAATTTAAAAAGAAGCAAACCATTTCGGTTTGGTTTCACGGTTTGGGCTTTATTAACCTCTATAATTAAAAAAACATCTACAATACGATAAAAGGGTGGGGTTCTAGAGTAAACACTAGTGTATTTGCGAACTGAGTGAACAAATCCTGCCATTGATCTACACACGTGTATGGCCAGGATCTCATCATCAAATCGTAAAATACACTAGTGTATTTCAACATCAAATCCTGGCCATTGATCTACACGTGTGTATGGCCAGGATTAGTTCACTCAGTTCGCAAGCTACACTAGTGTTCACTTTTGAACCTAATCCAACGATAAAAATGAAAAAAATTAACGATATTGTTTCAACGGTTAAAACTTAAAAGACGAATGTCATGCATTCTATCTTTTTAGGCAAAACGCAATCATGTGTATAGTCCAACTAAAAATAATTTAAAAACTCGCGTAAAATATAAAAAGTTACATAGCCCAAAAAGCTCGGTTTTTCCGATCCATTTTGAGTATAAAGCTTAAAAAAATTTGTTCTTCGCGGTTTGAAAAAAATTCTAATCCGACTGACAAAAGGTTTAAAAACCGACCACGAAAACAAACCAACAAACCAATAACCGGTTTGGATGGTTTTACGTCTTACAGGGGCGCAGCTTCCTTGGGGCGGGACCCCCCCCCCTAACTTTTCGACGCGTAGTGTTATGAACAGTACCTTCGTATAAAATTTTTTAGGCATAAACAACTAGTAACAACATAGGACATCTTATGATTGTTTTTTTTTATGTATTGTAACACAGGTTCTAACTTCAATAGGAAACTTTTGCATATGTTCAATAGCTATTGGTATGATAATTGAAGTTATTGTTGTATATGGTATCCATCAACGGGAATACCGTGTTGGAATCGATAACCTTCTAGTTCTGCTGATCGGCGGGATCCCTATCGCGATGCCAACGGTCCTTTCGGTCACCATGGCCATCGGGTCACACCGTTTGTCTCAACAGGTAAACCGAAATGCACCAGTTTTGTCTCTAGTCGTCATTGATTAACTCTAACATGATTTGAACAGGGCGCGATAACGAAAAGAATGACCGCGATTGAAGAAATGGCCGGGATGGATGTGTTGTGTAGTGACAAGACCGGTACATTGACACTTAACAAGCTTACGGTTGACAAGAACATGATCGAGGTCTTTGCGAAAGACGTTGACAAGGATATGGTCGTGTTGATGGCTGCCCGAGCATCGCGGTTAGAGAATCAGGATGCGATCGATGGTGCTATCGTTGCTATGCTTGGAGACCCCAAGGAGGTACTATTGTTTTCGGTTTCTTTTGTAAACAAGAAAAGTAAATAATTTGGTTTGGTGGCCAGTGGTGAAGCTTGACCCGAATGACCGGGGGGGGGGGGGGGGGGGGGGGGTCGAAAACGCATATACCCAAAAATTTCTATACAAAAACTACATATATAACACTACTGAGCGAAAAGTTCGGGGGGTCGGGCGCCCCTCCCCGCCCCTTCTATACTTCGCCACTGTTGGTGGCGGAACCAGAATATTTTTTTCGTGTTTTTCTTAGAAAAACCTCGGATTTTATATATAAAAATCCAACAAATTCCAGTGAGAAGTCAACAGACCCTCTTGACCCCCTGTTTATTAGATTTTAATTCTTAATATTTTAGGCCCGAGCTGGGATTCGAGAGGTCCATTTCCTACCGTTTAATCCAACGGACAAAAGGACCGCGCTTACGTACACAGATAAAACCGGTAAAATGCATAGAGTTAGCAAAGGCGCACCCGAGCAGGTATAAACATTGATCTAAAAAGAGTTAATTACTGTTTTCGTCCCTGTGGTTTGTCAAAAATCACTATTTCAGTCCATTAGTTTAAAAATTGCGATTTCAGTCCCTGTGGTTTCACTTTCGTAACTATTTCAGTCCACCTCGTAACCATTTCAGTCCCTGTACTAACAGAATAAATGGATTGAAATGGTTACGAAAGTAAAACCACATGGACTGAAATGGTTACGAAAGTGAAACCACAGGGACTGAAATCTCAATTTTTAAACTAATGGACTGAAATAGTGATTTTTGACAAACCACGGGGACGAAAACAGTAATTAACTCAAAAAGAGTTAATTAAATTACTGTTTTCGTCCCTGTGGTTTGTCAAAAATCACTATATCAGTCCATTAGTTTAAAAATTGCGATTTCAGTCCCTGTGGTTTCACTTTTGTAACCATTTCAATCCCTATGGTTTCACTTTCGTAACCCTTTCAATCCATTTATTATGTTAGTACAGGGACTGAAATGGTTACGAGGTGGACTGAAATGGTTACGAAAGTGAAACCAAAGGGACTGAAATCTCAATTTTTAAACTAATGGACTGAAATAGTGATTTTTGACAAACCACGGGGACGAAAACAGTAATTAACTCATCTAAACATATGTCTAAAATGTAACATGTAACATTTCACATCAATAAATCATTTGATAACATTGTGCAGATACTGAACCTTGCACATAACAAATCCGAGATCGCGAACAAGGTACATTCGATAATCGACAAGTTTGCAGAGCGTGGGCTTCGGTCGCTTGGAGTCGCTCGCCAGGTGATTCAACTGTTTAACACGGGCTCGATGGTTAATATCATAACTTTGTTTATAAAAAGTTGATTTTTTTTTTGTTTCAGGAAGTGCCAGCTGGAACGAAAGAGAGCCCGGGTGGCCCATGGGAGTTCGTCGGGCTTCTTCCGCTGTTTGACCCTCCACGTCATGATAGCGCTGAAACGATCCGAAGAGCGTTAGATCTTGGCGTGAGCGTTAAAATGATAACGGGTACTTTTAAAACCTGTCCCCGTTTCAGTTTTAAGTGTATATATGAGTCTAGGGGTGCTAAACGGGTCGTGCTCGTGGGTTGGTGGGTCCAACCCGAACCCGAAAATTTTGGATGAACCCGAATACGATCCAAACCTGAAAATCATGTCATACATATGAACCCGAACACGACCCGAATATTTGCAGGTTGACCCGAACACGACCCATTCAACCCGATATTTTTCTATTTTTTTTTTATTTTTTTTCACAAATTAATATATTAGAATTAAAAGTTTACTACATATTGTAAAATAATTCTGAGAAAAATGCCCGGGTAGTCCCTGTGGTTTGCTCCGTTTCTCTTTTTAAGTTATAATTATTACATAACATACTCACCCAATTTAATTTATGACATAAAACATATTGTAAAATAATAAAATATAATATAATTGGGTTAAACGGGTCAACCTGGCATCCCGACCGGGTTGACATGAACCTGACCCGTTTAGGTAAACGGGTTTGCGGGTTCAACCTGTAACTGACTCGACCCGTTTAGACTAAACCCAAACCCATAAATTTTGTGTTAGGTTCGTGTCGTATCAGAAATTCACACCCCTGTATGAGTCATGTTTGGGTTCATTTATTAAAACTATCAACCTGACACGATTGACACCCTTATGATTGCAGGTGACCAACTGGCAATTGGCAAGGAAACAGGAAGACGGCTCGGAATGGGGACCAACATGTACCCTTCTTCAGCATTGCTCGGTGACACTAAAGACGGTTTCGGAGCTCTGCCTGTCGATGAACTCATCGAGAAAGCCGATGGTTTTGCTGGTGTCTTTCCGGGTAAGAACTACAATCTTCATAACTTACTAAAAGAGTGAAGTACACGGATAGTCCATGTGGTTTAACAAAATTTTGAATTTGGTCCCTAGCTTTCCAAAAGTACACGGATGGTCCCTGTGGTTTGCACTTTGTAATGCATTTAGTCCCCACCCAACAAATCTAAAGGTTTTTGCATGTCCAAGTTAGGGACTAAACGCGTTACATAGTGCAAACCACAGGGACCATCCATGTACTTTTGGAAAAAGTTGGGGACTAAATGTGTTACAAGGTGCAAACCACAGGGACCATTTGTGTACTTTTGGAAAGCTAGGGACCACATCCAAAATTTTGGTAAAGCACAGGGACCAGTGGTGAAGCTTGACGTTTTCGACCGGGGGGGGGGGGTCGGAAAACGTATATACCAAAAATTTCTATAGAATCGGGGGGTCGAAAACGTATATACCCAAAAATTTCTATACGAAAACTACATATATAACACTAATGAGCGAAAAGTTCGGGGGGTCTCCAGGCCCCTTCAAAGCTTCGCCAATGACAGGGACCATCCGTGTACTTTACTCTTACTAAAACATACTAGATGGTGTTCTCAGTTAACGTGTTTCTCGCGTGCAGAACATAAATACGAGATAGTTAGAAGATTACAAGCCCGAAAACATATATGTGGGATGACGGGTGATGGTGTAAACGATGCACCCGCTCTAAAGAAAGCAGATATCGGGATCGCAGTGGCGGATTCCACTGATGCTGCTCGTAGTGCTTCGGATATTGTTTTAACCGAACCCGGGTTAAGTGTCATTATTAGCGCGGTTTTAACTAGCCGATCCATTTTTCAAAGAATGAAGAATTATACGGTAACTAACTATCTACTCGCTTAAAGTCTTCTGTTTTGTAGTGCGTATCTTTTGGACTAATAACATTTTTTATTTTCTTTTTCAGATATATGCGGTTTCAATCACTATTCGTATCGTGGTAAGTGTATCCGTTTTAACGTTAAAAAGTCGTATGTTTGACCTCGAAAGTCAAGGTAACTTGTTGACCGTCTCATTGTTGACTTTTTCCGCGTGCAGCTTGGTTTTATGTTGCTGTGTGTTTTCTGGAAGTTCGATTTCCCTCCGTTTATGGTTCTCGTTATAGCCGTCCTTAACGATGGTAAATCGAAGCATTCGTTATCAGTTTACGTAGTCAAACAAAATAACAAATGCTGACTTTTTTTTATCGTTCAGGTACGATCATGACAATATCGAAAGATAGAGTAAAGCCGTCTCCGATTCCCGACAGCTGGAAGCTCACGGAAATCTTCGCGACCGGGGTGGTTCTCGGTGCTTACCTGGCACTAATGACAGTTATTTTCTTCTGGTTGGCATATGAAACTAACTTTTTCCCGGTATGCAGCCTTTTCAATCTGACTCATTGTTTTCATAACCGGTCTGACCAGTGAACCGCTAGTTTGATGACTGGTCCAGTTTTCAAAACATTGCTTTGACTCAGATTTTTAAGCTTTTTAAGACATTTTAAGCTTTTTAAGACATTTTAAGCTTTTTTTATATATTATTTTAAAAAATACATATTTTGGGGTCTGATTAAAAAAAATGATATTTTGATATATATATTTTAATTTTTATTATATATTTTTAGTTTTTTAATACATTTTAAGCTTATATATATATTGGGGATGGATCATGAGAAAACTAGTTTAAATGAGAAAACCAAAAAACTAACTAAAAAAGCCTAAAAAGGATACCAATTTTTTTTTTACATTTTTTTTATAAAAAATCGCTATATTTTATGCATGGAAAAAAATATATAAAAAAAAATAAATAATAATTTTTTTTTGTTGTATTACACATGTGCACTATTACGGATATAGTGTACATGTGTAGTACACATATAATGCACATGTGCAGTACAACAAATTTTTTTTTAAATTTTTTTATATATAAAAAATAGCGATTTTTTTTATAAAAAATTGTAAAAAATAAATTTGGTATGTTTTTTAGCTTTTTTTTAGGCTTTTTTAGTTAGTTTTCTAGTTTTCTCATTTAGATGTAGTTTTCTCATGATCCTCTCCCTATATATATTAAAGAAATATATATATATGTATATTAAGGCCCTATTTTTATAACAGGGCCCAAAGATTTTTTAGGATTCTCGGGGACGGCCCTGCTCTGACTTTACGATATTCTGACCCGTTTTCTTGTTTCTCATGCAGAACTTATTCAGTGTCAAGGATTTGAACTCACATCATCGCGACATGTCAATCAAATCCGAGAAAGAAGAGCTAACAGCGATGATGGCGTCAGCTGTTTATCTTCAAGTGAGCACCATCAGCCAAGCGTTGATCTTCGTGACACGTTCGCGCGGCTGGTCTTTTACTGAGAGGCCCGGGTTTTTGCTAGTAACTGCATTTTTTATTGCTCAACTGGTAAAACACTAAAACTTACCTTTATTATGGTAACTTTTTAGCCTAATGTTTATTTTGAATATCGTTTTGCGTTTTTAGGTTGCGTCGATTATATCGGCCCATGTGACGTGGGAACTAGCCGGAATCCAAAAGATCGGATGGGGATGGACCGGGGTGATATGGTTGTATAACATCTTGACTTACATGTTACTTGATCCGCTCAAGTTTGCGGTTCAGTATGGGCTAAGTGGGAGAGCTTGGGGTCTAGTTGTGGAGAAAAGAGTAAGTTTATAGTCTGTTTGACTTTAAAACTTAAGTTTGACCTTTAAAGTCAAAGAGTTGTAGTTTGACCTTAAAAGTCAAACTGGTATCCTCTTTGACTTTAAAAAGTCCATCTAAGTGGAAAGTCAAAGCGGAGAAAAACTGATGCGATTATAGGGGATTGAATTTGTGACATGACACGAATGACGAATTCAACGTGAAATTCATGGGTTTGGGCCGAACCCCGTGAACACGTTTATCTAAACATGTTACCTTGTTTTGGGCCGAACCCCGTGAACACGTTTATCTAAACATGTTACCTTGTTTAACCCGTTAATTTTTGTATTTTGTACATGTTACCCCCGTGAACACGTTTATCTGAACACGTTTTCAGAAAGGATTGGCGGCGCAGCTTGTTGCCCGTCTACCTTCTGTTTTGATATAACTTAACAAGTTGTTCTAAATAAATTATATTTTATCTTAAAAAAAAAAAACTGACATAAACTTTTATAATTTAGACTTCACTATTTTACATACCTTTGTATTTTATAGAATATTATTGCACAAAAAATAAAATAAAAAATTGTGTTAAATAGGTCGTGTCTTTGTCAACCTGCAAATATTAGTGCCTTGTTCAGATCCACGTGTCTAACCCACAAACACGACCTGTTTAACACCCCTGTATAATTAACCGTTGTGGGTTTTGTAGACTGCATTTACGACGAAGAAAGACTTTGGGAGGGAAGCGCGTGAGGCAGCATGGGCAACGGAGCAACGCACATTGCATGGACTCCAGCCAGCAGAGCCAAGAACGTTTCCGGATCAAGGGACATTTAGGGAGATTAGTGTGATGGCCGATGAAGCTAGACGAAGAGCGGAGATCGCAAGGCTAAGAGAGCTTCATACACTTAAGGGCAAAGTTGAGTCTTTTGCAAAGCTAAGAGGGTTAGACATTGATGCAGCTAACCAACACTACACAGTTTAA

# >HHA10

ATGGAAGAAGTTTTCGAGAATTTAAGATGCACGAAAGAAGGACTGAACTCTGATGAGGTTGAAAAGCGTTTGAATATGTTTGGCTACAATAAGTTGGAAGAGAAGAAGGTTTGGTTGTAATTTGGATTGCATATGAATAGTTTGTTTTATTTATCGTTGGCTTTTTATATTTTAAGCATATAATAATCAATCATGAAACTTGCAGGAAAGTAAAATTCTGAAGTTTTTGGGTTTTATGTGGAATCCTCTGTCTTGGGTAATGGAAGCAGCAGCAATCATGGCAATTGCTATGGCTCGAGGAGGGGTTAGTCCATTTTTGTATCTTATTTGAAATTTATATATATGTATATATAGATGTTGATTATATGATTGTATATAAAACTATATGTTATTTTCTTTTTTTTCTGATTCTTGCACTTGGAATTATTGCTTCCAGGGTGAACCTGCTGACTATCACGATTTTGGTGGCATAGTGGTACTTCTGCTTATCAATTCAACCATAAGTTTTGTAGAAGAGAATAACGCTGGAAACGCAGCTGCAGCCCTCATGGCTCGTTTGGCTCCAAAAGCTAAGGTGCACCTTAATTAATGTGAACGTAAAAATAAGAAAAACGCAGATAAACATGATTAGTTACTGTATTTTTATAATATGTGTAACGGCTCTGGTCAGGTTGGCTTGACCATTAAACACCTTTTTTGTCTCTATAAAAAGAGTCACAGTTGCTTGTGTTTTTTTGAATACGAAACACGGTCATTACAGTAATATTATAAATATCTAAGTAAAACAAGTTAGATGGTTGTGTATATGATGTATATAGATTTTAGATAATTTAAACTCATTTGAACCGTTTCTATTTTCGTTAAATAATGTCATTTTGATATAAAATAGGTTTTACGCAATGGGAAATGGAATGAAGAGGATGCATCAATACTTGTACCTGGAGACATCATAAGTATTAAGCTAGGTGACATTATTCCTGCTGATGCACGTCTGCTTGAAGGAGATCCTTTAAAGATTGATCAAGTAAGAAGTTTAAGTTAATTATTATTTATTTTTTTATTTTAAACAAGAATCATATAGTTATTGACTTTTGATACGTTTTGCAGTCAGCTCTTACAGGTGAATCTCTCCCAGTAACGAAGAATCCAGGTGACGGGGTGTACTCAGGGTCCACGTGTAAACAAGGTGAAATTGAGGCAGTTGTTATTGCGACAGGGGTCCATACGTTCTTTGGGAAAGCAGCTCATCTTGTGGACAACACTACACACATAGGTCACTTTCAACAGGTTTTGACTGCGATTGGGAACTTCTGCATATGCTCAATTGCGATAGGCATGGTTATTGAAATAATTGTGATATATGCTCTTCAAAAGAGGCATTATCGTGAAGGAGTCGATAACCTTCTCGTGATACTCATTGGTGGGATCCCAATTGCAATGCCAACTGTTTTATCTGTTACAATGGCCATTGGCTCCCATAAACTAGCTCAACAGGGAGCTATAACAAAGAGAATGACCGCCATTGAAGAAATGGCTGGAATGGATGTATTGTGTAGTGATAAAACTGGAACTTTAACTCTCAACAAACTTACTGTTGACAAAAATTTGATTGAGGTGATGTAATTTTTTTTTTCTATTGGTTTTGGAAAAAAAAAAAAGGATTATTTTTTCTTAAATTCATGAATTATGCAGGTTTTTGCAAGTGGTGTTGATAAGGACACGGTTGTGTTAATGGCTGCTAGAGCATCAAGGTTGGAAAACCAAGATGCCATTGATGCTTGTATAGTATCAATGCTGGCTGATCCTAAGGAGGTATTCATATTATCTCTTTTTTATTGGAATGATTTTTTTTTTTTTTTAAGTTTTTAACATTATAATTAATTATATATATGGAATGAATTAAGCAAAACTTAAATAATATTTATTATTGTGAATGTTGCAAGGCGCGATCCGGAATTACAGAAGTGCACTTCCTTCCATTTAATCCAACTGATAAGAGGACAGCTTTGACATACATTGACGGTGCTGGCAAGATGCATAGAGTTAGCAAAGGTGCACCAGAACAGATTTTGAATCTGTCAAAAAACAAATCGGAGATTGAAAAGAGGGTACACGCAATCATTGATAATTTCGCTGAACGTGGACTTCGATCTCTTGGAGTGGCTCGCCAGGAAGTACCAGCTAACAGTAAAGATAGCCCTGGTGGTCCGTGGGAATTTGTGGGGCTTCTTCCTCTATTCGATCCACCTCGCCATGACAGTGCTGAGACAATTAGAAGAGCTTTAGATCTTGGAGTTAGTGTTAAGATGATCACCGGTGACCAACTGGCCATTGCTAAGGAGACTGGGAGACGACTAGGAATGGGTGTTAACATGTATCCTTCATCATCCTTACTTGGCGATCATAAGGATCAACTACTTAGAGCTTTACCTGTTGATGAACTTATTGAAAAAGCCGATGGTTTTGCTGGTGTTTTTCCAGGTAACCATTCAACTCATATTGGTATTTCCACCTTTAGATGTTCATGGTAAACGCTTGAATCCTATTACATACAGAGCACAAGTATGAAATTGTGAAGATTCTTCAAAGTAAAAAGCACATTTGTGGAATGACGGGTGATGGTGTAAACGATGCACCTGCATTAAAGATAGCCGACATTGGAATTGCAGTGGATGATGCCACAGATGCAGCTCGAAGCGCGTCTGACATAGTTCTAACTGAGCCGGGGCTCAGTGTCATCATCAGCGCGGTGTTAACAAGCCGAGCCATCTTCCAGAGAATGAAAAACTATACAGTAAAATCCTCAACTATTATGCTGTTTTATTTTTTTTTTTACATGGATTCAAATTTTTAAACATTCCCTCCTTTTTCATTCTCCAGATCTATGCCGTTTCAATCACCATACGTATTGTGGTAAGTTAACTTAATCTTTACTTGCTAAATCAGCATTGTTTGTAATCTTTAAATTTTGTTCTGTTGCAGTTAGGGTTCATGTTGCTGACTTCTTTTTACGAATTTAATTTCCCTCCAATTCTTGTTCTTGTCATAGCCATTCTGAATGACGGTATGGTTGATCGAAATGATACAAATATGTATTTTCAAAAATCTGTTTTTAATCAACAATATGTATCCAGGTACGATCATGACTATTTCCAAAGATAGAGTGAAACCATCTCCCAGCCCAGACAGCTGGAAGCTCAGTGAAATTTTTGCAACTGGAATAGTCATAGGCACCTACCTTGCTCTAATGACTGTCTTATTCTTTCATTTGGCTAGTCAAACCAATTTTTTTGCGGTACAAAAAAATTATCGTTTCATGTTTAATGGATTATGTAATGTAATAAATAATAACTCTTAACCGCTGTTACATGCAGACATTTCTAACTTTCTTTCACTCTGTTATCCTTTCAGCATACCTTCCATGTGGAAAGTTTACATAAGCATAAAGGCCTCGCAGATGATGTTTGGAAAGCGAAGCTAGCATCAGCTGTATACCTCCAAGTCAGCACAATTAGCCAGGCGTTAATATTTGTCACACGCTCCAGGGGTTGGTCTTTCACGGAAAGACCAGGTCTTCTGTTGCTTGCTGCATTCATTCTAGCTCAACTCGTAAGCATCTTATTTTCTTGGTTGGTTTCCCTTTGATATAGTTGTTGTTTTATTGTAATGTGGATGTAATATCTTTGCAGTTTGCCACTGTGATGTCAGCATATTTGAGCTGGAGTTTTGCTAAGGTCCATGCGATTGGTTGGGGTTGGACTGGAGTTATATGGTTGTATAACATCTTAAGCTACATGTTGCTTGACCCTATCAAATTCGCCGTTAGATATGCGCTTAGTGGGAGAGCATGGGGCCATGTTATTAACCGTAAAGTGAGTAAAACTTTCATTTACTTTCGTGTCAAGTAAAATAAGACTCAGAAAATGAAAACCATAGATTATTGAATTTTGCAGACGGCATTCAGCACCCAAAAGGACTTTGGTAGGGAAGCCCGCGAGGCAGCATGGGCAAAAGAGCAAAGAACACTTCATGGTCTTGATACTTCTGAAGCAAAGCCTTTTGCTGAAAACTACACCTTCAGAGACATCAACATGATGGCGGAAGAAGCAAAACGTCGTGCAGAGATTGCAAGGTCTATATTTTCCTTCTTTCCTACACCGCTAACCATTTATTTAAAAGTTATTAATAAAACAAGACTACAAAAAATGATGAAACATGATTTAATAAATAAATATTTTAGTAAATAAACTCGCGAGACCCCACCACCAATGCCTTGCTCACTCAAGAGTGGAAGCAGGAAATCCAAACACTTTGGGTAGTTTTTGGACCCTTTATTTTTCAACTTATTATTTTTGTTTAGAAATAAATGTGCCCGGTTAAAAAGAAAACCCGAGTACATGATCTCTCACATCATCCGTTGTTGCCCTACAGATTAAGGGAACTTCATACCCTGAAGGGAAAGGTAGAGTCTTTTGCGAAGCTAAGAGGATTAGACATCGATGTGAACCCACACTACACTGTGTGA

# >HHA11

ATGGCGGAGGCGGATGATATTATGGAAGCCGTGAAGAAGGAAGCGGTTGATTTGGTTAGGAACTGTAACCTATATCTGTGTGTGTTTTATATTATGAACGATTTCTTAGTCGATTATGATGTTAATGCTAGCTTTTGCCAGACTACTTGATTGTTATTTAGTAAAGGATTATGAATTATTTAATACAGTTATATACTTTTATTTTTGTCTTGTATGGTTAATGTAATAAATTTGAATTATAGGAAACGATACCAGTCGAGGAAGTTTTCGAAAATTTGAGATGTTCCAAAGAAGGGCTAACATCAAAGGATGCTGAAAAGAGATTAGAGATATTTGGGCACAATAAGCTTGAAGAGAAGGAGGTGTGTGGCGTATTGTACCTACATATCTAATTATTTGTTGCTTGTTAGGTGTCAGGCTTACAATTATTGTTCTTAAACCTGATTAATTTGTAAATTTCTGTTGAGGAGATTTGAGATTCACTAAATGTTTTAATTTCAGGAAAGCAAAATTTTGAAGTTCTTAGGGTTTATGTGGAACCCACTATCATGGGTCATGGAAGTTGCAGCAATTATGGCCATTGCACTTGCCAATGGAGGAGTGAGTAATCTCAAATGTATTTAAATATTTAGCACTGTGCTGATAGCTATATAAGTTCTATATACATGTGGTGTTATGTTGACGTTTCTTTCAAATTAATTGATGGATCTTGGTTATTGTGCAGGGTAAACCACCTGACTGGCAGGATTTTGTTGGGATTATCACACTGCTTGTCATAAATTCAACAATAAGCTTTATAGAGGAGAACAATGCTGGTAACGCAGCAGCAGCTCTCATGGCACACCTTGCACCTAAAGCGAAGGTACTAACATCTCATTTGAGTTTGGCATCTCTTGTGATTGACTGTAATATGTTCTATATATTAAATTAACAATGTCAATAAATGAAATATTAGGTGCTTCGCGATGGAAAATGGAATGAGGAAGAAGCAGCAATTCTTGTTCCAGGTGATATAATCAGTATTAAACTAGGAGATATCATCCCAGCAGATGCTCGACTTCTTGAGGGTGATCCTTTAAAGATAGACCAGGTCTTTTTTTTTCTTTTATTATTATGACAGTCATATTCATCGTATACTGAATTATTTGGTTCATCCTTTTCTTATACATTTCATCTTCTAATGATTCCAATTGGTTGATTTGCAGTCTGCTTTAACAGGTGAATCCCTTCCTGTAACAAAGCTCCCTGGGGATGGAGTTTATTCAGGCTCTACATGCAAACAGGGGGAAATTGAAGCAGTGGTTATTGCGACAGGTGTGCATACCTTCTTTGGAAAGGCTGCACATCTTGTAGATTCTACAAACCAAGTAGGCCATTTTCAGAAGGTATGAAAGTACACGTGCATCTAATTATATTTGAAACAAATACCAGCTTTTGTTAAGTTTGAAAAACGATGGCTGCTCTGGTAATAAAGATATCACAATTTGCAGGTTCTGACAGCGATTGGGAACTTCTGTATATGTTCAATTGCAATCGGAATGGTAGTAGAAATCATCGTGATGTACCCAATTCAAAACCGTAAATATCGTCCTGGAATTGACAATTTGCTAGTGCTTCTCATTGGTGGAATTCCTATTGCAATGCCAACAGTTTTATCGGTCACAATGGCAATTGGTTCTCATCGTTTATCTCAGCAGGTACGTTTAAAACCCTTTCTGCTTTTGTTGTTTCATATGCTTTTCTTGTCAGAAGGACCCTAATATGTGCGCAACTTTTCTACATCACTACAGGGAGCTATCACAAAAAGAATGACAGCAATAGAAGAAATGGCAGGCATGGATGTTCTTTGCAGTGATAAAACTGGGACTTTGACCCTGAACAAGCTTTCGGTTGACAAAAATCTTATAGAGGTTGCTTGATCATTTGAAGTTTTGAACACTTGGTTTGTTTCAAAATTCTGTCTCACTTTAAATTCTATAACGCATCCTTTATCTTTGCAGATATTTGCTAAAGGAGTGGATGCTGATATGGTGGTGTTGATGGCTGCAAGAGCTTCTAGGGTGGAAAACCAAGACGCAATAGATGCTGCCATAGTAGGGATGTTGGCTGACCCTAAGGAGGTTAATATCTATCGTTAACGATAATACACAAAATTAGATCAATTGTTTTTCTGATCTGGTTTGAAATAATCTAATAATATATTAATATCATATGTTTTATCTAGGCACGGGGAGGTATTCAAGAGGTCCATTTCCTTCCATTCAACCCTACCGATAAGAGGACAGCATTAACTTACATTGATGGTGAAAACAAAATGCATCGTGTCAGTAAGGGTGCTCCTGAACAGGTAAAGCAACTGATGCACATACATTTTAACTTTTAACAATCATCAGTAACTGACCAACCGACTACATGTCTTTTTATAATTTTTCAGATTTTGAATCTTGCACATAATAAATCAGAAATTGAGCGTAGAGTACATCTTGTGATAGATAAGTTTGCCGAGCGTGGTTTACGATCCCTTGCAGTAGCCTACCAGGTTTGAAAAAAAAAATCTAAAACAAAAGACCCTGTAGTTAGTTTTTCGATATATACTAATCTTTTTAAACAGGAAGTCCCTGCGGGAAAGAAGGAAAGCCCAGGAGGTCCATGGGAGTTTATCGGTCTTATGCCTCTCTTTGACCCACCCAGACATGACAGTGCAGAGACAATTAGAAGGGCTTTGAATCTGGGAGTAAGCGTCAAAATGATCACAGGTGTGGGTTCTTCTATCATAGCAGATGTGACAAGTATCATAGAAACTTATATTTATATATATAGTAAAATGCTATTTTCGTCCCTAAGGTTTGACCAATATTACGACTTTCGTCCAAAGGTTTGTTTTTTCGCATCTGGATCCAAAAGGTTTGAAATCTTGCCATTTTCATCCTGCTCGTTAACTCCATCCATTATTCTCCGTTAAGTCAGAGGTATTTCCGTCTTTTTTGCTAACTTAAAGGGCAATTCGGTCTTTCCACTTTATGTAAAAAGACCCAATACCCTTGGAAAAGATCGAATTAAGTTAACAAAAAAAGACGGAAATACCCCTGACTTAATGGAGAAAAATGGATGGAGTATCGAGCTGGATGAAAATGGCAACATTTCAAATCTTTTGGATCCAGATGCGGAAAAACAACCCTTTGGACGAAAGTCGCAAAACTGGCCAAACCTCAAGGACGAAAATGACATTTTACTCTCATTTAGTCATTTATATTAGCAAAACGCAACTTGTGCTGGGAAGGTGGCCTTCAAGGGGGTTAAAGCCGGTGGAAGTTGGATTTAAACCTGAGACCTCTGTTAGAGGAACTCAAGGGCGCTATCCCCTTGAAGGTTAATAAAACAAGTTTCTAAGTCTGTGGTCCATATGCATGACCTTTTTGATCTCGAATAGGCGATCAGCTAGCAATAGGAAAGGAAACAGGACGGCGTCTAGGAATGGGGACCAACATGTACCCTTCATCTGCATTACTGGGACAGAACAAAGACGAGTCAATTGCCGCCTTACCCATTGACGAGTTGATTGAAAAAGCTGATGGATTTGCTGGTGTCTTTCCTGGTACGTTCATTGTATATATGCATGAGAGCTTCATCACTAACGTAATAAACAATAATCATAGGCATAAATTTGTGGCTTTGCTACAGAGCATAAATATGAAATTGTGAAACGTTTACAAGCAAGAAAGCATATATGTGGTATGACTGGTGATGGAGTGAATGATGCACCTGCACTTAAGAAAGCGGACATTGGAATTGCGGTTGCAGATGCAACTGATGCTGCACGTAGTGCGTCTGATATAGTGCTGACTCAACCTGGTCTTAGTGTTATTATCAGTGCAGTACTAACTAGCCGAGCAATCTTTCAGAGGATGAAAAATTACACTGTAAGTTGAAGCTCTGATCTTGAGAAGCGTATTCATTCATTTTGCCAATCTTTATCTCATGGACCTACCTCCATTTATTCTTATATTTTGGTAATAGACCACAAAATAACAAAGTTTGACCCATATTTGGTTGTGTTTTATTATAATGGATCAACAATATTAGCTAAATGATTTAAGGAAAAATGATACGGGTCAAATGAGTTGTATTTAAATGCATAAAACACCCAAGATGGTTTTATAAAAAAAAGTTAGTTTATTAGCTATTATTTCAGTGAATAGTTTAATGTATTCGTCACTGTTCAGTTATTTTACAAAAAAGACACTAAACTTTTTGCAAGTTTGCATTTGGGATACTTAACTTTTTTCCGATTAAAAGTATCATACTTTTTTTGTCCATTTTGGGCACTCGATTTTTACCATTTCTAGTTAAAATCATTTCTTTTTGCTCTAACCAACACTGCATATCTAACAGTTTTGACCCGTTACCCGATCCGTCCAATTTTGACCCATTTTGCCATCTATTAATATTTAATATGATAACTTAGACTGAAAGAAAATGGGCAGGACTTATAGGTTCGGGTAAAAAATAAATTAAGAAGCTAACAAAAGAGCACTCTTTGTAACAGATATTTGTTATATCATGTTAAGAAGCCCTCTAATTTTGTAATACCCAATTTCTCTTATGCAGATATACGCAGTTTCCATTACTATCCGTATAGTGGTATGCTACTATGCTGTCCTGACTTTTACTATGCACAACTCTATGTTTTCTACCTATTATTTCACTTACATTCTCAAATCTCTTTCTCAACAGCTTGGTTTCATGCTACTAGCCCTCATATGGAAGTTTGACTTTCCACCCTTCATGGTCCTGATCATTGCAATTCTTAACGACGGTTAGAATTTGTTCTTCAACACAGCCATAAGCCCATAATACCTTCTTATTAATGTATTTATCTATTTTGTTGATAGTGTCATCTCAACTAACTAGTTGCACTTTCCAGGTACCATTATGACAATTTCCAAGGATAGAGTGAAACCGTCTCCTCAACCGGACAGCTGGAAATTGGCAGAGATTTTCGCTACAGGGATCATTCTCGGTAGCTACTTGGCTATGATGACTGTAATTTTCTTCTGGGCTGCGTATGAGACCGATTTTTTCCCGGTAAGAAAGTTTCTCTTCATTGTTTAATTTTAGAAATAATCATTGCTTTTCTATATTTATAGAGCATTGTCTAGATTTTATAAGAAGTACTCCTAAGTTATTATTTTCAAAATTTCTCAATCATCCACTTTATAAGTTTAAAAATTTTTCCTGGCGTGCCATTTTCCAGCGTGTTTTTGGAGTTTCTAGCCTTCAGAAAACTGGTCAAGTTACATTGGACGATGTTAAAAAAAAGCTAGCCTCAGCAGTATACCTACAAGTGAGCACCATCAGTCAAGCTTTAATATTTGTTACACGTTCTAGGAGCTGGTCCGTTTATGAACGTCCAGGAGCTCTACTTTTCGGTGCCTTCTTGATTGCTCAATTGGTGAGCGGATTTACCAATTATGATTATTATTGATTATTACGTAGTTAGTTAATAAATCTTTTTTTTTTCAGATTGCAACGTTGATTGCAGTTTATGCAGACTGGAATTTTTCTGCAATCGAAGGGATCGGATGGGGTTGGGCAGGTGTAATATGGCTCTACAACATTGTATTCTACATCCCACTCGATTTCATAAAGTTGTTCATCAGATATGCTATAAGTGGTAGAGCATGGGATCTTGTCATTGACCAACGCGTGAGTAAAACTTTGACCAACTAATTAATGCACCCCTTATCTACAGGATACTTGAAATACATTTTTGGTGCAGGTTGCTTTCACCAGGAAAAGAAACTTTGGGAAGGAAGACCGTGAGCTTAAATGGGCTCAAGCACAACGGACACTACACGGGCTGGACCCGCCTGAAATACATAGCGTTGACCGCAACAACCACAATGAACTTAATCAGATGGCTGAAGATGCAAAACGCCGAGCTGAGATGACAAGGTACACTTCCCTTTTTAGTATTCTTACATAGCTAGAACAAACATAAAAGATAAGCTCATAAGCCACTGATATATTTATTTGAAAGATATCAGTTTATACGCGTGCATTGCATACATGCAGGTTGCGAGAGTTGCTTACGCTGAAAGGTCACGTGGAATCAGTTGTAAAACTGAAGAACATCGACATAGATACCATCCAACAATCCTACACTGTGTGA

# >HHA12

ATGGGGGAAGAGAAGCCTGAAGTTCTTGAAGCTGTTTTGAAGGAGACTGTAGATTTGGTAATGGCTCTTGGATCTGGGTTTCAAATTTTTATTTTTTTTTTGTTTTTGATTTTAATTTTTCAGTGTTTTTTTTTTTTTTTTTTTTTTTTTTTTTTTTTTTTTTTTTTTTTTTTTGCAGGAGAGTATACCCATTGAGGAGGTTTTTGAAAATCTGAGATGTAGCAAAGAGGGTCTCACTACTGCTGCTGCTGAAGAAAGATTAGTCATTTTTGGGCATAATAAACTTGAAGAAAAAAAGGTGAACTTTAATAAAAAAAATCAAGATTTTATATGGGTTTTTCTTGGAAAATCTTGTTATTATGTTATTGTTTTTGTTTTTAATAAATTTGATGAATTTATGATGTGGGTGTGTTTAGGAGAGCAAATTCTTGAAGTTTTTAGGGTTTATGTGGAACCCATTATCATGGGTTATGGAAGCTGCTGCTATTATGGCCATTGCCCTTGCAAATGGAGGAGTAAGATTTAAGAACTAAGAACCCTAATTGATTCATTATTTTCTTAAATTTACCATTTTTTTTCTAAGAAAAACCTTACCTAAATTCAATTTATTTTTTTGTAGGGGAAGCCTCCTGATTGGCAAGATTTTGTGGGTATTATTACTTTGCTAATTATCAATTCCACAATTAGTTTTATTGAAGAAAACAATGCTGGTAATGCTGCAGCTGCTCTCATGGCTCGTCTTGCCCCGAAAGCAAAGGTATCGTATCGTCTTTTTTTTTTTTTTTTTTTTTATAATTATAATTCAATAATTTGGAGATTATGGTTTTGTTGTTGATTAGTTAATTATTATTATGAATTTTATATAAGATTTTACGCGATGGGAAATGGAACGAGGAAGATGCTTCTATGTTGGTTCCTGGTGATATAATTAGCATAAAGTTAGGTGATATTATTCCAGCAGATGCTCGTTTGCTCGATGGCGACCCTTTAAAGATTGACCAGGTGAGCGGTCAACCGCCTGAAGTTTTGTTGTGTAATTGTTTCTTGATTTAATTCTGGTATTTTGTACTAAACGGACTGTCTTTTGTAGTCGGCTCTAACCGGCGAGTCTCTCCCGGTCACAAAAGGTCCCGGAGATGGTGTTTATTCCGGTTCGACTTGCAAACAAGGTGAAATCGAAGCGGTTGTGATTGCCACCGGGGTCCACACGTTCTTTGGTAAAGCTGCTCATCTTGTTGATAGCACAAATCAAGTCGGTCACTTTCAAAAAGTAAGCACAAAAAAAATCAAAACTTTACGATTACAATCCGAGTATGAGTTATTTTGTTTTTCAAGATTTTACTTATTATGATTTACTTGTTAGGTTTTGACTGCGATCGGGAATTTCTGTATTTGTTCTATTGCTGTTGGAATGGTTATCGAAATTATCGTAATGTTCCCGATTCAAGATAGGCAATATAGGCCCGGAATCGATAATCTTCTCGTGCTTTTGATCGGAGGCATCCCGATTGCTATGCCTACCGTTCTTTCCGTAACGATGGCAATCGGGTCTCATCGATTGGCTCAACAGGTTTCTTAACTTCCTACATGATCTTTCTTTTTAGATTTTATGTTTTTATTTTGATTTTTTTGTGGTTAATGTTTTATTATTATGATTATTACAGGGAGCGATTACGAAAAGAATGACAGCGATAGAAGAAATGGCGGGAATGGATGTGCTATGCAGTGACAAGACCGGGACGTTGACTTTGAACAAGCTTACAGTAGACAAGAATCTTATTGAAGTCGGTTTCCTAGATGCTTGTTTCTTTTTTTTAATAACAAATTTTGTGTTTTTTACTTCTAGTTTGACCTTTTGAGTTGACTTAATTTATTAGGTGTTTTCCAAAGGAGTAGATGCCGATACCGTTGTTCTGATGGCAGCGCGTGCCTCCAGAACCGAAAACCAGGATGCCATCGATGCTGCAATTGTCGGTATGCTGGCTGATCCAAAAGAGGTAATATTATCTAAATGAACGATCTATATATTTATATATATATACACATCGTTCTCATACAAGTTCACTAATTTTGTGTCTTCACAGGCGCGTGCGGATGTTCAAGAGTTGCACTTTCTGCCGTTTAATCCTACCGATAAGCGTACGGCCTTAACGTATCTGGACAATCAGGGTAAAATGCATAGAGTCAGCAAAGGTGCCCCCGAGCAGGTCTGTATCTATCCAAACCAATGACGAAAAGACGGTTGTGTGAAAAAAAAATATATATTTTTTTTAATTTTTGTATATATTTCGTAATTCATTAATGGTTTTGTTTTTAATTGGTGTAGATTTTGAATCTTGCGCATAACAAATCAGACATAGAACGCCGAGTTCATGCTGTCATTGATAAATTTGCAGACCGTGGGTTAAGGTCACTTGCTGTAGCATACCAGGTAACCCGTTCTGACCGTTCATGCTTTTATACACGAAAGCTTGTTTTGCTAATTTTCTTTTATTTTTTTAGGGTTTGAACATTGACTGTTTTGTTTATTTATTTATTTATTTATGTGAAAATAGGAAGTTCCGGAGGGACGGAAAGAGAGCGCCGGAGGGCCGTGGCAGTTCATTGGGCTAATGCCGCTTTTTGATCCGCCACGTCATGATAGCGCTGAGACGATTCGAAGGGCTTTGAATCTTGGAGTAAACGTTAAAATGATTACAGGTGCATATCGTCTTGAAGTTTTTGTTTTTGTTTTTGTTTTGACTCTTTGTGGTCAAACATATTTCTTTAAATAAATATGATTCCTTCGTTTTACAAGAAAAATGACAAAAATAAGTCGACAACAAACATAATTACAAATTTTACAAATTATTGTTTTTGGTTTTAAATTTTAATCTTCTGATTAAAATAGCTTCTAATCATAACACATGTATGATCACTAAAAGGGTTGACCAAAAAGCGACCAGCAATCGTTTGACCCGCTAACCTTTGAACAGGGGATCAACTGGCAATCGGGAAAGAAACCGGAAGACGTCTTGGAATGGGAACCAACATGTATCCTTCTTCGGCTTTACTCGGTCAAAACAAAGACGAGTCAATTGCCGCTTTACCCATCGACGAACTCATAGAGAAAGCCGACGGTTTTGCCGGTGTTTTTCCGGGTACGTATTCTGCATATAAATTGGCAAAAGTCGAAACAATATAACAGATATAACGTTAATATGTTATATACAATTACATATCCTTGCAATTTTTATTTCAGAACACAAATACGAAATAGTAAAACGTTTACAAGCTAGGAAACACATATGTGGCATGACTGGAGATGGAGTAAATGACGCTCCCGCCCTTAAAAAAGCCGATATCGGAATTGCGGTGGCTGATGCAACCGATGCAGCCCGTAGTGCTTCCGACATCGTCCTTACTGAGCCTGGGCTTAGCGTCATCATCAGTGCTGTTTTGACCAGTCGGGCCATCTTCCAAAGGATGAAAAATTACACTGTAATGACCCTTTAACGTTAACCTTCTCTAGCTCGTTTTAAACATTCTTTTGAGCTTAACTATATTGAATTTTCGCAGATTTATGCAGTTTCAATCACCATTCGTATAGTGGTAAGTTATGCAGTGTCATCACGTGAATGTTATGTTTATGTGTATAATATTAATATCTTTTAAACTTTTTTTTTCTTGTTTCAGCTCGGTTTTATGCTGCTGGCTTTGATATGGAAGTTTGACTTCCCGCCTTTCATGGTGCTTATCATCGCAATCCTTAACGACGGTTTGTGCTTTTTTCTTTGTTCTCTAATCCGTTTTATTAGATAAAGTTATTATTATTATTTTTAAGATAATAAATAATAGAGTTAATTGCCCGGATGGTCCCTGTGGTTTCACGTTTTTTCACGTTTAGTCCCCACCTTTTGGAAATAGCAGGTATGCTCCCTATGGTTTGTCATTTTGTTACTCGGATAGTCCCCTGACATTTACTCCGAGTAACAAAATGACAAACCATAGGGAGCATACCTGCTATTTCCAAACTATCTGACATCTACTCAGGGGACTATCCGAGTAACAAAATAACAAACCATAGGGAGCATACCTACTATTTTCAAAAGGTGGGGACTAAACGTGAAAAAACGTGAAACCACAGGGACCATCCGGGCAATTAACTCTAAATAATATTTACAATCATATTTCATTTCAGGAACCATAATGACCATATCAAAGGATAGAGTGAAGCCATCTCCTCTTCCCGACAGCTGGAAACTGGCTGAGATCTTCACCACCGGGGTGGTTCTTGGTAGTTACCTAGCCATGATGACAGTTATATTTTTCTGGGCCGCTTATAAAACAGACTTCTTTCCGGTACGTGCATTTATGCTTTAGCATCTTACCCTAAACGGTGACATGTTTTATTTATTTTTTTGTTAACGTATTTCCAGCGTACATTTGGTGTACCGACGCTTGAAAAAACAGCGCACGATGACTTCCGGAAGCTGGCGTCAGCGATATATCTCCAAGTCAGCACAATCAGCCAAGCTCTCATTTTCGTAACGAGATCTAGAAGCTGGTCATTCGTGGAACGTCCTGGTTGGCTGCTCGTTATTGCTTTCGCGATTGCCCAATTGGTAAATTTATATTCCTATTATTTATTTTATTCCGAGATAAAGGATAAATAAATTAGTAGTAATAATAATATTGTCTTCGGTATCTACCAGGTTGCGACATTAATTGCGGTTTATGCCAACTGGAGCTTTGCTGCGGTAGAAGGAATCGGGTGGGGATGGGCCGGAGTCATCTGGCTCTACAATATCGTGTTCTATTTCCCGCTCGATATCATCAAGTTCTTTATCCGATACGCGCTTAGTGGGCGGGCCTGGGACCTTGTTATCGAGAGGAGGGTATGCGCTAATGTGCTAACGTTTTGTGGTATAAAACTTGGGCGAAAATGTTTTGTTTTGTCTAATGATGTTTTCTGTTTTTGGCTTTTTTAAGATTGCGTTCACGAGGCAAAAGGACTTTGGGAAGGAACAACGTGAGCTCCAATGGGCTCATGCGCAACGAACCCTTCACGGGCTTGAAGTTCCTGACACTAAAATGTTTGGTGACCGCACCAATGTCACGGAACTTAACCAGATGGCTGAAGAAGCCAAACGGAGGGCTGAAATTGCAAGGTATGGCCAACCCGTTATCCAATATAAACATATACCGTTATCGTTGATGGTTTTCAATAAGTTTAGGGGCTGTTTGTTTACCTCTTAATGGGGCTCTTAATGGTTCAGACCTCTTACTGGTTCAGCACTTAATGGTTCAGACTGTTTGTTTAGCGAACAAATGTCTGAATGGTTCAGACATTTGCTTCTGAATGGTTAAGAATTATACGAAGTCTGAATGGTTAAGACCTTTAATCTGAATTGGTCAGACATTTGCCTCTGAACAGTTAAGCATTATACTGGCTCTTGATGGTTCAACACTTAATGATTCAGGCCTAGGCCTCTTACTGGTTCAGCACTTAACCATTCAGAAGTTGCCAAACAACCCCTTACTCTTTGTGTTTTCTAATTGGTGTTTGGGTTTTTGTTAGGTTGAGAGAATTACACACGTTGAAGGGTCATGTTGAATCGGTGGTTAGACTGAAGGGTCTTGACATTGAGACGATTCAACAAGCGTACACCGTCTGA

# >HHA13

ATGGGGGAGGAGAAGCCTGAAGCTCTTGAAGCTGTTTTGAAGGAGACTGTAGATTTGGTAACTTATTGTTATGGATCTGTAGTTCTCATTTTGGTTTTTTGTAATTTTTTTTAAAGAAAAAAATTGATTTTTTTTTTGTTTTTTTTTTTTTTTTTGCAGGAAAGTATACCCATTGAAGAGGTTTTTGAGAATCTGAGATGTAGCAAAGATGGTCTCACCACTTCTGCTGCTGAACAAAGATTAATCATTTTTGGGCATAATAAACTTGAAGAAAAAAAGGTAATTTGGTTTTCCACCCAAATCTTGTGATGTTTTTTTTTTTTTTTTTTAATAATTTATGATGTGGGTATTTTCAGGAGAGCAAATTCCTGAAGTTTTTAGGGTTTATGTGGAATCCATTATCATGGGTTATGGAAGCTGCTGCTATCATGGCTATAGCCCTTGCTAATGGAGGAGTAAGAACATCAACACCTTCAAAATTTATAAAAAAAAATTAATAAAAAAAAACAAAAACAATGATAATTCAAAATAATGATAATTTTGCAGGGAAAGCCTCCTGATTGGCAAGATTTTGTGGGTATTATCACTTTGCTCATAATCAACTCCACCATTAGTTTCATTGAAGAAAACAATGCTGGTAATGCAGCAGCTGCTCTCATGGCTAGACTGGCCCCAAAAGCAAAGGTATTATAAGTTCATATCATTTATTTATTTATTTTGTTCATTATTTATTAATTTTGAATTTAAATTATATCAAAAGATCTTAAGAGATGGGAAATGGAATGAAGAAGATGCTTCCATGTTAGTCCCTGGAGATATAATTAGTATAAAGTTAGGTGATATTATTCCAGCCGATGCTCGTTTGCTCGATGGCGATCCGTTAAAGATTGACCAGGTGAGTGGTCAGTCAAACTTGTATGATTGTTTCTTGATCATGTTTTAGTAATCATGTACTGAACCCGTGAACCGGTGAACCCAACTTTTGTAGTCGGCTTTAACCGGTGAGTCTCTCCCGGTCACAAAAGGTCCTGGTGACGGTGTCTATTCCGGTTCGACATGCAAACAGGGTGAAATCGAGGCGGTTGTGATAGCTACCGGGGTCCACACGTTCTTCGGGAAAGCTGCTCATCTTGTTGATAGCACAAACCAAGTTGGTCATTTTCAGAAAGTAAGCACGAAAAGGATTCGAATTTCACATCCGGGCATGTGTGTTTTCGAGGTTTTGAATTTGTTTGTGATCTTTTTAGGTTTTGACTGCGATCGGGAATTTCTGTATTTGTTCTATTGCTGTTGGTATGGTGATTGAGATAATTGTGATGTTCCCGATTCAAGATAGGCAGTATCGCCCCGGGATCGATAATCTTCTTGTTCTTTTGATCGGAGGAATCCCGATCGCTATGCCGACCGTTCTTTCCGTAACGATGGCTATCGGGTCTCATCGATTGGCGCAACAGGTTTCTTGACTTGCATCATCTTTCTTTTATGAATTTTATATTTTTATAACATTTTTGACAATTTATATGATTAATACAGGGAGCGATTACGAAAAGAATGACAGCGATAGAAGAAATGGCAGGAATGGACGTTCTTTGCAGTGACAAAACCGGAACGTTGACTTTGAACAAACTTACAGTTGACAAGAATCTTATCGATGTCTGTCTCTTAGATGCGTATTTTTTTATTACAAATTCAGATTTTTTTACCTGTAGTTTGACTTTTGAGTTGACTTACTTTGTTAGGTATTTGCCAAAGGAGTAGACGCCGATACTGTGGTTCTGATGGCGGCCCGAGCCTCGAGAACCGAAAATCAAGATGCCATTGATGCTGCAATAGTCGGTATGCTGGCTGATCCAAAAGAGGTAAAATATTATCTCAAAAAAAATATGTCAATGTTGCTTAGAAAGTCGAAAAATACCAAAAAAAATAAATATATGTAAAATTTAAGACAGCCAAAAAACACATTAAATTTGGAGGGTAGGGGCCTGTTGATCTCTTTATATTCTCAAATTAATTTCTAGAGGTGGGCCAGGTGGCGGTTTTGACCTATTTACTTTTTGTTGGGTCAATTTCGGTACCGGTTTATCAAAACATTTAGCTAAAATGCAAACGGGTCGAATGGGTCTCTTATGTTGTTTTATTTTGAAATTAGATTGCTATTGTAATAACTAATAACATAGTTTCTATAATCATATTTAAAGCACTTACTATATTCAATTTGTTTATCTGTTGCTGTTGTTCTTAGATAAGTTGTTCACTTATGCTTTTATCTTAATAGGCACGTGCTGATATTCAAGAGTTGCATTTTCTGCCATTTAATCCTACCGATAAGCGTACTGCATTAACGTATTTAGACAGTCAGGGTAAAATGCATAGAGTCAGCAAAGGCGCCCCCGAGCAGGTATGTGTGTGTATCTATCGAATTCCGTGAAAAAAAAAATTAATAATTAAGTTTTCGTGTACACCTTTTTATTTATTTGTGCTTTTTTTTTTTTCTTAAATTATTTCAGATCTTGAATCTTGCACACAACAAATCGGACATAGAACGCCGAGTTCATGCTGTCATCGACAAGTTTGCGGACCGTGGGTTAAGATCACTTGCTGTTGCATACCAGGTGAAACATGATGTTCATTAAGTTTTTTTTTTTTTTTAATAATTAAAATTGTTTGGGTTTTGATTGTTGGTTGTGTTTTAAAAAAAAAATAGGAAGTTCCAGAGGGACGAAAAGAGAGTCCAGGAGGACCATGGCAGTTCATTGGGCTAATGCCGCTCTTTGATCCGCCACGTCATGATAGTGCCGAGACGATACGGAGGGCTTTGAATCTTGGAGTAAATGTTAAAATGATTACAGGTGCATTTCATTTTTAACTATAGTTTTGACTTTTTGAGGTCAAACATATTACCCTTAAATACAATGCCTTCGAGTTTCACGAAAAATATGACAAGAACAAAGGTTCAAAGTTCAAACATAAAATGACAATATTCACTAATCGGAAAAGTATCGGTATTAATCTGCTAGAAAATCCGTTTCATTTTCAGTTTTGAATGGTACCCATTTCTTGTAAGAGAGCTAGCAATGTTTCTTTCGGTTGTTCCATTGATATTATTTGTTTCCATATACAATAGGTGTCATTTTGATTATTTTTTTTAGCTCAAACGGGTCAAAAGTTGCCCAAATGTACTTTTAATGTGTACAACCTTTTATTAACGATTCGGATTATTAATAAAATAATATAGTTTTGTAATCGTGGTTGATAAGAGTTGTCTATTTTCGATAAATGATTAAAAAAGTGCTTCGAGTTCAACCTGACCCGTTTCAATCTGCACTAAACAGGGGATCAACTGGCAATCGGGAAAGAAACCGGAAGACGTCTTGGAATGGGAACCAACATGTACCCTTCTTCAGCTCTGCTCGGTCAAAATAAAGACGAGTCAATTGCTGCTTTACCCATCGACGAACTCATAGAAAAAGCCGACGGTTTCGCGGGTGTTTTCCCCGGTACGTTTTTGGTTTTCCCCATTTCATTAATCAGATCTGAAACAACATAACAAAGCTAATGTTATACACATCCTTGTAATTTTGTTTCAGAACACAAATACGAAATTGTAAAACGTTTACAAGCTAGGAAACACATATGTGGAATGACCGGAGACGGAGTAAATGATGCTCCCGCCCTCAAGAAAGCCGATATCGGGATTGCAGTGGCTGATGCGACCGATGCAGCCCGTAGCGCTTCTGACATCGTCCTTACCGAGCCCGGGCTTAGTGTCATCATCAGTGCTGTTTTGACCAGCCGCGCCATCTTCCAAAGGATGAAAAATTACACGGTAATGTCCCCATTATTTTTTTCTAAAGTACACGGATGGTCCGTGTCGTTTACCAAAATTTCGGATTTGGTCCCTAGCTTTCTAAAAGTACATGGATGGTCCCAAATTTTGGTAAACCATAGGGACCGTCCGTGTACTTTACTCTTTTCTCTACTTTTTTTAATGCATTTTGTTGAGCCTAATCGTATTCGGTCTTTCGCAGATTTATGCGGTTTCAATCACCATTCGTATTGTTGTAAGTTACTTGTTACCATCGCACCATTCACACACACATATTTTATTTTTCATGTGTATCTCGGCTCGGGTCAGCTCGGTTTTTAAACTTTGTTTGTTATTTGTTTTCGGGTCAGCTCGGTTTCATGCTGCTGGCTTTGATATGGAAGTTTGACTTCCCGCCTTTCATGGTGCTTATTATCGCAATCCTTAATGACGGTTCGGGCCTTTTTCTCCCTTCTCTAATCTTCTTTATTAGAAAAAAAAGCCCGATTTATTTTTGTCATAATAAATTATTTTTTGCCATTATATTTCATTTCAGGAACTATAATGACCATATCGAAGGATAGAGTAAAACCGTCTCCTCTTCCCGATAGCTGGAAACTGGCGGAGATTTTCACCACCGGTGTCGTTCTCGGTAGTTACTTAGCGATGATGACGGTTATATTTTTCTGGGCGGCTTATAAAACAGACTTCTTTCCAGTATGTGATTTAATTAGTGGGCCGGGCCGGGCTGGTTGGGTATCGATTAAAACGGGCCAGGTTGCTTGGCTCGAAACACTTATAATTTTTTTTTTTGCAAATGAATCCGCAGCGTACATTTGGCGTTCCGACCCTTGAGAAGACGGCTCACGATGACTTCCGGAAGCTCGCTTCGGCAATATATCTCCAAGTCAGCACAATTAGTCAAGCTCTCATTTTCGTAACGAGATCTCGTAGCTGGTCGTTTGTGGAACGCCCGGGTTGGCTGCTTGTTATTGCTTTTGCTATTGCGCAATTGGTATGCTATATCTAATCTAACTTATATTTGACTTTAAAAAATTTTTAAATCCTTATCCTTCTCTCAATTTCTTTAAAATAAATATGATAACAAAGACGTTATATAATATTACAAACAATATTAGAATTTTAAATTATAATATTATAAACTACCACCTCCGCACAAATCTTGTAAACAAAAAAATGACTGAATTTATCACATGGAAAATTTAAATTATATTATAATTTATGAAACAATTGTTATACCCATTAACAATACAAGATATTCGACCGTGTGATAAGATGATAATATTATTACATTAATTAACCGTGTTACACATTTTTATAGAATTATAGTTTATATTTCCTCAACACGTGTGATAAAATTCATGATTTATGAATGTATAATATTATAATATTATTACACTTTTTAACCATTTGATAAGGATTATATAAAAGTTAATGATTTATATTTTTTTTCTACATTCAATCTGTGTCATACTATAAATTAAAATAATATTCAATCTATTTTTTTTTTAATATTTAACACGTGTAACAAAAGGGGTTGTAAGCTAGTGTAATAATTAATTATTAATAATAACAATAATAATCATAAGTTAGTAATATTTTCTTTTGGTTTATACCAGGTTGCTACATTGATTGCGGTTTATGCCAATTGGAGCTTTGCTGCCGTAGAAGGAATCGGGTGGGGATGGGCTGGCGTTATCTGGCTCTACAATATCGTCTTCTACTTCCCACTTGATATCATTAAGTTCTTTATCCGATATGCTCTTAGTGGGCGGGCCTGGGACCTCGTTATCGAGAGGAGGGTACGTGTTTAACTTTTTGTGGTTTAAACTCGGGTGATAACGTCTTTTTTTTAATGATGTATGTTTGTGGGATTATTTAGATTGCTTTCACAAGGCAAAAGGATTTTGGTAAGGAACAACGTGAGCTCCAATGGGCCCATGCGCAACGAACCCTTCACGGGCTTGAAGTTCCTGACACCAAAATGTTTGGTGATCGTACCAATGTCACTGAACTCAACCAAATGGCCGAAGAAGCCAAACGGAGAGCTGAAATCGCAAGGTATGGCCTTCTGAACTCACTGTGGGCCATTTATTTATGAATGGGCCGTGGTTCGGCCCATACCAAATTACTTGCTTTGATAAGTTACCCGATAGAACATACCGATTTTCCTCGGATTATCTCTTTTAAGTTGCATTGTGTTTTCTGAATCTTAATCTGTGTCGGGATTTTGTTAGGTTGAGAGAATTGCACACGTTGAAGGGTCATGTTGAATCGGTGGTTAGACTCAAGGGTCTTGACATAGAGACAATTCAACAAGCATACACCGTGTAA
