# Supplementary material for: Genome-Wide Identification and Analysis of P-Type Plasma Membrane H+-ATPase Sub-Gene Family in Sunflower and the Role of HHA4 and HHA11 in the Development of Salt Stress Resistance
Source: Genes (Basel). 2020 Mar 27;11(4):361. doi: 10.3390/genes11040361 (PMC7231311; doi:10.3390/genes11040361)
Supplement: Supplementary file 1 [file genes-11-00361-s001.zip › Supplementary File 4.docx]

Supplementary File 4: Protein sequences of sunflower PM H^+^-ATPase genes.

# >HHA1

MGGDKALSLEGIKNETVDLEKVPIEEVFEQLKCNREGLSSDEGAQRLEIFGPNKLEEKKESKILKFLGFMWNPLSWVMEAAAIMAIALANGGGKPPDWQDFVGIVCLLVINSTISFIEENNAGNAAAALMAGLAPKTKLLRDGRWSEQEAAILVPGDIISIKLGDIVPADARLLEGDPLKIDQSALTGESLPVTKNPYDEVFSGSTCKQGELEAVVIATGVHTFFGKAAHLVDSTNQVGHFQKVLTAIGNFCICSIAVGMAVEIIVMYPIQHREYRSGIDNLLVLLIGGIPIAMPTVLSVTMAIGSHRLSQQGAITKRMTAIEEMAGMDVLCSDKTGTLTLNKLTVDKNLIEVFGKGLDKEQVLLYAARASRMENQDAIDAAIVGTLADPKEARAGIREVHFFPFNPVDKRTALTYIDNNGNWFRASKGAPEQILTLCGCREDLKKKVHAMIDKFAERGLRSLGVARQEVPQKSKDSPGGPWEFVGLLSLFDPPRHDSAETIRRALNLGVNVKMITGDQLAIAKETGRRLGMGTNMYPSSSLLGGHKDESIAGLPVDELIEKADGFAGVFPEHKYEIVKKLQERKHICGMTGDGVNDAPALKKADIGIAVADATDAARSASDIVLTEPGLSVIISAVLTSRAIFQRMKNYTIYAVSITIRIVFGFMFIALIWKFDFSPFMVLIIAILNDGTIMTISKDRVKPSPLPDSWKLKEIFATGVALGGYLALMTVIFFWIMKDTDFFSDKFGVKSLRTSETEMMAALYLQVSIVSQALIFVTRSRSWSFVERPGFLLMGAFLAAQLVATVIAVYAEWEFARIKGIGWKWAGVIWLYSIVFYFPLDIMKFAIRYILSGKAWLSMIDQRTAFTTKKDYGRGEREAQWAHAQRTLHGLQAPDTSNLFNEKSSYRELSEIAEQAKRRAEVARLREVLTLKGHVESVVKLKGLDIDTIQQHYTV

# >HHA2

MRPSICSEKVPIEEVFEQLKCNREGLSSDEGAQRLEIFGPNKLEEKKESKFLKFLGFMWNPLSWVMEAAAIMAIALANGGGKPPDWQDFVGIVCLLVINSTISFIEENNAGNAAAALMAGLAPKTKVLRDGRWSEQEASILVPGDIISIKLGDIVPADARLLEGDPLKIDQSALTGESLPVTKNPYDEVFSGSTCKQGELEAVVIATGVHTFFGKAAHLVDSTNQVGHFQKVLTAIGNFCICSIAVGMVVEIIVMYPIQHREYRKGIDNLLVLLIGGIPIAMPTVLSVTMAIGSHRLSQQGAITKRMTAIEEMAGMDVLCSDKTGTLTLNKLTVDKNLIEVFGKGLDKEQVLLYAARASRMENQDAIDAAIVGTLADPKEARAGIREVHFFPFNPVDKRTALTYIDNNGNWFRASKGAPEQILTLCGCKEDLKKKVHAMIDKFAERGLRSLGVARQEVPQKSKDSAGGPWEFVGLLSLFDPPRHDSAETIRRALNLGVNVKMITGDQLAIAKETGRRLGMGVNMYPSSSLLGGHKDESIAGLPVDELIEKADGFAGVFPEHKYEIVKKLQERKHICGMTGDGVNDAPALKKADIGIAVADATDAARSASDIVLTEPGLSVIISAVLTSRAIFQRMKNYTIYAVSITIRIVFGFMFIALIWKFDFSPFMVLIIAILNDGTIMTISKDRVKPSPLPDSWKLKEIFATGVALGGYLALMTVIFFYIMRDTDFFSDKFSVKSLRTSETEMMAALYLQVSIVSQALIFVTRSRSWSFVERPGFLLMGAFLAAQLVATLIAVYAEWEFARIKGIGWKWAGVIWLYSIVFYFPLDIMKFAIRYILSGKAWNSMIDQRTAFTTKKDYGREEREAQWAHAQRTLHGLQAPDTSTLFNEKSSYRELSEIAEQAKRRAEVARLREVLTLKGHVESVVKLKGLDIDTIQQHYTV

# >HHA3

MGGDKALSLEEIKNETVDLEKVPIEEVFEQLKCNREGLSSDEGAARLQIFGPNKLEEKKESKLLKFLGFMWNPLSWVMEAAAIMAIALANGGGKPPDWQDFVGIVCLLVINSTISFIEENNAGNAAAALMAGLAPKTKVLRDGNWSEQEAAILVPGDIISIKLGDIVPADARLLEGDPLKIDQSALTGESLPVNKNPYDEVFSGSTCKQGEIEAVVIATGVHTFFGKAAHLVDSTNQVGHFQQVLTAIGNFCICSIAVGMLVEIVVMYPIQHREYRNGIDNLLVLLIGGIPIAMPTVLSVTMAIGSHRLSQQGAITKRMTAIEEMAGMDVLCSDKTGTLTLNKLSVDKNLIEVFAKGVDKDQVLLYAARASRTENQDAIDAAIVGTLADPKEARAGIREVHFFPFNPVDKRTALTYIDERGNWHRTSKGAPEQILTLCGCKEDMKKKVHAMIDKFAERGLRSLAVAKQEVPEKNKESPGGPWTFIGLLSLFDPPRHDSAETIRRALNLGVNVKMITGDQLAIAKETGRRLGMGTNMYPSSSLLGNHKDASIAAIPIEELIEKADGFAGVFPEHKYEIVKKLQERKHICGMTGDGVNDAPALKKADIGIAVADATDAARGASDIVLTEPGLSVIISAVLTSRAIFQRMKNYTIYAVSITIRIVFGFLFIALIWKFDFSPFMVLIIAILNDGTIMTISKDRVKPSPLPDSWKLKEIFATGIVLGGYLALMTVIFFWIMKDTNFFSDTFGVKSLRHSEVEMMAALYLQVSIVSQALIFVTRSRSWSFIERPGLLLLGAFLAAQLVATLIAVYANWEFARIKGVGWGWAGVIWLYSIVFYFPLDLMKFAIRYILSGKAWNNLLENKTAFTSKKDYGREEREAQWALAQRTLHGLQQPETSNIFNEKSSYRELSEIAEQAKRRAEVARLREVLTLKGHVESVVKLKGLDIDTIQQHYTV

# >HHA4

MGTDKALSLEEIKNETIDLEKVPIEEVFQQLKCNREGLTSDEGAARLQIFGPNKLEEKKESKILKFLGFMWNPLSWVMEAAAIMAIAMANGGGKPPDWQDFVGIVCLLVINSTISFIEENNAGNAAAALMAGLAPKTKVLRDGRWSEQEASILVPGDIISIKLGDIVPADARLLEGDPLKIDQSALTGESLPVNKNPYDEVFSGSTCKQGEIEAVVIATGVHTFFGKAAHLVDSTNQVGHFQQVLTAIGNFCICSIAVGMLVEIIVMYPIQHRQYRSGIDNLLVLLIGGIPIAMPTVLSVTMAIGSHRLSQQGAITKRMTAIEEMAGMDVLCSDKTGTLTLNKLTVDKNLIEVFAKGVDKEQVLLYAARASRTENQDAIDAAIVGTLADPKEARAGIREVHFFPFNPVDKRTALTYIDERGNWHRTSKGAPEQILTLCGCKEDMKKKVHAMIDKFAERGLRSLAVGKQEVPEKTKEGPGGPWQFVGLLSLFDPPRHDSAETIRQALHLGVNVKMITGDQLAIAKETGRRLGMGTNMYPSSSLLGGHKDPSIATLPIEELIEKADGFAGVFPEHKYEIVKKLQERKHICGMTGDGVNDAPALKKADIGIAVADATDAARSASDIVLTEPGLSVIISAVLTSRAIFQRMKNYTIYAVSITIRIVFGFMFIALIWKFDFSPFMVLIIAILNDGTIMTISKDRVKPSPLPDSWKLKEIFATGIVLGGYLALMTVIFFWIMKDTDFFTEKFGVRSLRNSEVEMMAALYLQVSIVSQALIFVTRSRSWSFIERPGLLLLGAFLAAQLVATLIAVYAEWEFARIKGVGWGWAGVIWLYSIVFYFPLDIMKFAIRYILSGKAWRNMLENKTAFTTKKDYGREEREAQWALAQRTLHGLQAPETSNIFNEKSSYRELSEIAEQAKRRAEVARLREVLTLKGHVESVVKLKGLDIDTIQQHYTV

# >HHA5

MADSGISWEELKKENVDLETVPVDEVFETLKCTRQGLTTEEGNRRLNAFGPNKLEEKKESKFLKFLGFMWNPLSWVMEAAAIMAIVLANGGGKPPDWQDFVGITTLLIINSTISFIEENNAGNAAAALMAGLAPKTKVIRDGKWDEQDAAILVPGDVISVKLGDIIPADARLLEGDPLKIDQSALTGESLPVTKHPGASVYSGSTCKQGEIEAVVIATGVHTFFGKAAHLVDSTNQVGHFQKVLTSIGNFCICSIAIGLIIEIVVMYPIQKRTYRNGIDNLLVLLIGGIPIAMPTVLSVTMAIGSHKLSEQGAITKRMTAIEEMAGMDVLCSDKTGTLTLNKLTVDKTLIEVFVKDADKDQVVLLGARASRVENQDAIDACIVGMLSDPKEARAGITEVHFLPFNPVDKRTAITYIDQNGNWHRVSKGAPEQIVELCDQKEEDKKKVFSIIDKFAERGLRSLAVCQQAVPEKTKESAGGPWVFVGLLPLFDPPRHDSAETIRRALHLGVNVKMITGDQLAIGKETGRRLGMGTNMYPSSSLLGQNKDPSIADIPIEELIEKADGFAGVFPEHKYEIVKKLQERKHICGMTGDGVNDAPALKRADIGIAVADATDAARGASDIVLTEPGLSVIVSAVLTSRAIFQRMKNYTIYAVSITIRIVLGFMLIALIWKFDFSPFMVLIIAILNDGTIMTISKDKVKPSPLPDSWKLNEIFATGIVLGTYLAVMTVVFFWLAKESDFFTEKFGVKPIKDNEYELMSALYLQVSIISQALIFVTRSRSWSFVERPGLLLLTAFFIAQLIATLIAVYANWDFARVHGIGWGWAGVIWLYSILFYFPLDVFKFIIRFALSGKAWDNMLQNKTAFTTKKDYGRGEREAQWASAQRTLHGLQAPNANDIFSDKSDYRELSELAEQARRRAEVARLRELHTLKGHVESVVKLKGLDIETIQQHYTV

# >HHA6

MAASGISWEELKKENVDLETVPVDEVFETLKCTKAGLTTEEGNRRLAAFGPNKLEEKHESKLLKFLGFMWNPLSWVMEAAAIMAIVLANGGGKPPDWQDFVGITTLLIINSTISFIEENNAGNAAAALMAGLAPKTKVIRDGKWDEQDAAILVPGDVISVKLGDIIPADARLLEGDPLKIDQSALTGESLPVTKHPGQSVYSGSTCKQGEIEAVVIATGVHTFFGKAAHLVDSTNQVGHFQKVLTSIGNFCICSIAIGLIIEIVVMYPIQKRSYRNGIDNLLVLLIGGIPIAMPTVLSVTMAIGSHKLSEQGAITKRMTAIEEMAGMDVLCSDKTGTLTLNKLTVDKTLIEVFAKDVDKDQVVLLGARASRVENQDAIDACIVGMLSDPKEARAGITEVHFLPFNPVDKRTAITYIDQSGNWHRVSKGAPEQIVELCNQKDEDKKKVFSIIDKFAERGLRSLAVCQQAVPEKTKESPGGPWVFVGLLPLFDPPRHDSAETIRRALHLGVNVKMITGDQLAIGKETGRRLGMGTNMYPSSSLLGQSKDSSIADIPIEELIEKADGFAGVFPEHKYEIVRKLQERKHICGMTGDGVNDAPALKRADIGIAVADATDAARGASDIVLTEPGLSVIVSAVLTSRAIFQRMKNYTIYAVSITIRIVIGFMLIALIWQFDFSPFMVLIIAILNDGTIMTISKDKVKPSPLPDSWKLKEIFATGIVLGTYLAVTTVIFFWLAKESDFFTEKFGVKPIKDEEFELMSALYLQVSIISQALIFVTRSRSWSFVERPGLLLLTAFFIAQLIATLIAVYANWDFARVHGIGWGWAGVIWLYSIIIYFPLDIFKFIIRYSLSGKAWDSMIEKRTAFTSKKDYGRGEREAQWATHQRTLHGLQAPNANDILNDKSDYRELSELAEQAKRRAEVARLRELHTLKGHVESVVKLKGLDIETIQQHYTV

# >HHA7

MAESEGITWDDIMKETVDLEHMPMDELFDQLKCTKEGLTSEEGKRRLGIFGPNKLEEKKESKFLKFLGFMWNPLSWVMEAAAIMAIALANGGGKPPDWQDFVGITTLLLINSTISFIEENNAGNAAAALMAGLAPKTKLLRDGKWAEAEAEFLVPGDIISIKLGDIVPADARLLEGDPLKIDQAALTGESLPVTKKPGNSVFSGSTCKQGEIEAVVIATGVHTFFGKAAHLVDSTNQVGHFQKVLTAIGNFCICSIAVGLIIEIVVMYPIQKRTYRNGIDNLLVLLIGGIPIAMPTVLSVTMAIGSHKLSQQGAITKRMTAIEEMAGMDVLCSDKTGTLTLNKLTVDKSLIEVFVKDMDKDTVILMGARASRVENQDAIDACIVGMLADPKEARAGINEVHFLPFNPVDKRTAITYTDQDGNWHRVSKGAPEQIVELCNLKGDTSKRVFDIIDKFAERGLRSLAVCQQTVPEKTKEGPGGPWVFVGLLPLFDPPRHDSAETIRRALHLGVNVKMITGDQLAIGKETGRRLGMGTNMYPSSSLLGQHKDASIANIPIEELIEQADGFAGVFPEHKYEIVKKLQERKHICGMTGDGVNDAPALKRADIGIAVADATDAARGASDIVLTEPGLSVIVSAVLTSRAIFQRMKNYTIYAVSITIRVVLGFMLLALIWKFDFSPFMVLIIAILNDGTIMTISKDKVKPSPMPDSWKLKEIFLTGIVFGTYLAVMTVIFFWLAQDSDFFPDKFGVRSIRNKDYELTAALYLQVSIVSQALIFVTRSRSWSYVERPGLLLLTAFLIAQLIATLIAVYAHWDFARINGIGWGWAGVIWLYSIIFYIPLDIFKFIIRYAMAGRAWDNLLQNKTAFTSKKDYGRGEREAQWVQEQRTVHGLQPPEQPEQFVNDKTSYRELSELAEQAKKRAEVARLRELHTIKGHVESVVKLKGLDIDTIQQHYTV

# >HHA8

MSDNSLEEVKSNQIDLEKIPIEDVFTILNCTRDGLNDEEATKRLDIFGHNKLEEKQESKLLKFLGFMWNPLSWVMEFAAIMAIVLANGGGRPPDWPDFVGIVVLLLINSTISFIEENNAGNAASALMASLAPKAKILRNGKWSEQDAGILVPGDVISVKLGDIIPADARLLEGDTLKIDQSALTGESVPVNKNPGEPVYSGSTCKQGEIEAVVIATGVRTFFGKAAHLVNSTDSAGHFQQVLTSIGNFCICSIAIGMVIEIVVIWVIQGRGYRDGIDNLLILLIGGIPIAMPTVLSVTMAIGSHHLAKQGAITKRMTAIEEMAGMDILCSDKTGTLTLNKLTVDKSLIEVFVKDCDRDMVVMYGARASRIENQDAIDACIVNMLADPKEARAGIKEVHFLPFNPVDKRTAITYIDNKGDWYRSSKGAPEQIIELCNLTGDTLKRAEEIIDGFANRGLRSLGVARQTVPEKTKESEGSPWEFVGLLPLFDPPRHDSAETIRRALELGVKVKMITGDQLAIGKETGRRLGMGTDMYPSSSLLSESNDANNSSIDDLIEKADGFAGVFPEHKYEIVKRLQQRNHICGMTGDGVNDAPALKRADIGIAVDDSTDAARSASDIILTQPGLNVIVAAILTSRAIFQRMKNYTIYAVSITIRIVMGFMLIVVIWRFDFSPFMILVIAILNDSTIMMISTDRVKPSPLPDSWKLNEIFATGIVLGTYLSLVTVLFFWLSSRTDFFPRLFGVRSIVGNDDEVTAALYLQVSIISQALIFVTRSQSWSFLERPSTLLMLAFVLAQILATLLAVYADWDFAEMQGIGWRWAGVIWMFSIVTYIPLDILKFIIHAALNGNNSR

# >HHA9

MHFYFDVHMENKSRKYCPVRRHWEPEDQHICLAYDHGSVVCATPNTWESSSASTSRVHSNRAKTHQGVTLTLNQKDPRRVKTYGRKMDDKSIALSAVIREAIDLENAPVEEVFQHLKCTREGLNSSEVQERLDLFGYNKLEEKKESKILKFLGFMWNPLSWVMEAAAIMSIALARGGGKSADYHDFVGIIVLLVINSTISFIEENNAGNAAAALMARLAPKAKVLRDGKWSEEDASLLVPGDIISIKLGDIVPADARLLEGDPLKIDQSALTGESLPVTKNPGDGVYSGSTCKQGEIEAVVIATGVHTFFGKAAHLVENTTHVGHFQRVLTSIGNFCICSIAIGMIIEVIVVYGIHQREYRVGIDNLLVLLIGGIPIAMPTVLSVTMAIGSHRLSQQGAITKRMTAIEEMAGMDVLCSDKTGTLTLNKLTVDKNMIEVFAKDVDKDMVVLMAARASRLENQDAIDGAIVAMLGDPKEARAGIREVHFLPFNPTDKRTALTYTDKTGKMHRVSKGAPEQILNLAHNKSEIANKVHSIIDKFAERGLRSLGVARQEVPAGTKESPGGPWEFVGLLPLFDPPRHDSAETIRRALDLGVSVKMITGDQLAIGKETGRRLGMGTNMYPSSALLGDTKDGFGALPVDELIEKADGFAGVFPEHKYEIVRRLQARKHICGMTGDGVNDAPALKKADIGIAVADSTDAARSASDIVLTEPGLSVIISAVLTSRSIFQRMKNYTIYAVSITIRIVLGFMLLCVFWKFDFPPFMVLVIAVLNDGTIMTISKDRVKPSPIPDSWKLTEIFATGVVLGAYLALMTVIFFWLAYETNFFPNLFSVKDLNSHHRDMSIKSEKEELTAMMASAVYLQVSTISQALIFVTRSRGWSFTERPGFLLVTAFFIAQLVASIISAHVTWELAGIQKIGWGWTGVIWLYNILTYMLLDPLKFAVQYGLSGRAWGLVVEKRTAFTTKKDFGREAREAAWATEQRTLHGLQPAEPRTFPDQGTFREISVMADEARRRAEIARLRELHTLKGKVESFAKLRGLDIDAANQHYTV

# >HHA10

MEEVFENLRCTKEGLNSDEVEKRLNMFGYNKLEEKKESKILKFLGFMWNPLSWVMEAAAIMAIAMARGGGEPADYHDFGGIVVLLLINSTISFVEENNAGNAAAALMARLAPKAKVLRNGKWNEEDASILVPGDIISIKLGDIIPADARLLEGDPLKIDQSALTGESLPVTKNPGDGVYSGSTCKQGEIEAVVIATGVHTFFGKAAHLVDNTTHIGHFQQVLTAIGNFCICSIAIGMVIEIIVIYALQKRHYREGVDNLLVILIGGIPIAMPTVLSVTMAIGSHKLAQQGAITKRMTAIEEMAGMDVLCSDKTGTLTLNKLTVDKNLIEVFASGVDKDTVVLMAARASRLENQDAIDACIVSMLADPKEARSGITEVHFLPFNPTDKRTALTYIDGAGKMHRVSKGAPEQILNLSKNKSEIEKRVHAIIDNFAERGLRSLGVARQEVPANSKDSPGGPWEFVGLLPLFDPPRHDSAETIRRALDLGVSVKMITGDQLAIAKETGRRLGMGVNMYPSSSLLGDHKDQLLRALPVDELIEKADGFAGVFPEHKYEIVKILQSKKHICGMTGDGVNDAPALKIADIGIAVDDATDAARSASDIVLTEPGLSVIISAVLTSRAIFQRMKNYTIYAVSITIRIVLGFMLLTSFYEFNFPPILVLVIAILNDGTIMTISKDRVKPSPSPDSWKLSEIFATGIVIGTYLALMTVLFFHLASQTNFFAHTFHVESLHKHKGLADDVWKAKLASAVYLQVSTISQALIFVTRSRGWSFTERPGLLLLAAFILAQLFATVMSAYLSWSFAKVHAIGWGWTGVIWLYNILSYMLLDPIKFAVRYALSGRAWGHVINRKTAFSTQKDFGREAREAAWAKEQRTLHGLDTSEAKPFAENYTFRDINMMAEEAKRRAEIARLRELHTLKGKVESFAKLRGLDIDVNPHYTV

# >HHA11

MAEADDIMEAVKKEAVDLETIPVEEVFENLRCSKEGLTSKDAEKRLEIFGHNKLEEKEESKILKFLGFMWNPLSWVMEVAAIMAIALANGGGKPPDWQDFVGIITLLVINSTISFIEENNAGNAAAALMAHLAPKAKVLRDGKWNEEEAAILVPGDIISIKLGDIIPADARLLEGDPLKIDQSALTGESLPVTKLPGDGVYSGSTCKQGEIEAVVIATGVHTFFGKAAHLVDSTNQVGHFQKVLTAIGNFCICSIAIGMVVEIIVMYPIQNRKYRPGIDNLLVLLIGGIPIAMPTVLSVTMAIGSHRLSQQGAITKRMTAIEEMAGMDVLCSDKTGTLTLNKLSVDKNLIEIFAKGVDADMVVLMAARASRVENQDAIDAAIVGMLADPKEARGGIQEVHFLPFNPTDKRTALTYIDGENKMHRVSKGAPEQILNLAHNKSEIERRVHLVIDKFAERGLRSLAVAYQEVPAGKKESPGGPWEFIGLMPLFDPPRHDSAETIRRALNLGVSVKMITGDQLAIGKETGRRLGMGTNMYPSSALLGQNKDESIAALPIDELIEKADGFAGVFPEHKYEIVKRLQARKHICGMTGDGVNDAPALKKADIGIAVADATDAARSASDIVLTQPGLSVIISAVLTSRAIFQRMKNYTIYAVSITIRIVLGFMLLALIWKFDFPPFMVLIIAILNDGTIMTISKDRVKPSPQPDSWKLAEIFATGIILGSYLAMMTVIFFWAAYETDFFPRVFGVSSLQKTGQVTLDDVKKKLASAVYLQVSTISQALIFVTRSRSWSVYERPGALLFGAFLIAQLIATLIAVYADWNFSAIEGIGWGWAGVIWLYNIVFYIPLDFIKLFIRYAISGRAWDLVIDQRVAFTRKRNFGKEDRELKWAQAQRTLHGLDPPEIHSVDRNNHNELNQMAEDAKRRAEMTRLRELLTLKGHVESVVKLKNIDIDTIQQSYTV

# >HHA12

MGEEKPEVLEAVLKETVDLESIPIEEVFENLRCSKEGLTTAAAEERLVIFGHNKLEEKKESKFLKFLGFMWNPLSWVMEAAAIMAIALANGGGKPPDWQDFVGIITLLIINSTISFIEENNAGNAAAALMARLAPKAKILRDGKWNEEDASMLVPGDIISIKLGDIIPADARLLDGDPLKIDQSALTGESLPVTKGPGDGVYSGSTCKQGEIEAVVIATGVHTFFGKAAHLVDSTNQVGHFQKVLTAIGNFCICSIAVGMVIEIIVMFPIQDRQYRPGIDNLLVLLIGGIPIAMPTVLSVTMAIGSHRLAQQGAITKRMTAIEEMAGMDVLCSDKTGTLTLNKLTVDKNLIEVFSKGVDADTVVLMAARASRTENQDAIDAAIVGMLADPKEARADVQELHFLPFNPTDKRTALTYLDNQGKMHRVSKGAPEQILNLAHNKSDIERRVHAVIDKFADRGLRSLAVAYQEVPEGRKESAGGPWQFIGLMPLFDPPRHDSAETIRRALNLGVNVKMITGDQLAIGKETGRRLGMGTNMYPSSALLGQNKDESIAALPIDELIEKADGFAGVFPEHKYEIVKRLQARKHICGMTGDGVNDAPALKKADIGIAVADATDAARSASDIVLTEPGLSVIISAVLTSRAIFQRMKNYTIYAVSITIRIVLGFMLLALIWKFDFPPFMVLIIAILNDGTIMTISKDRVKPSPLPDSWKLAEIFTTGVVLGSYLAMMTVIFFWAAYKTDFFPRTFGVPTLEKTAHDDFRKLASAIYLQVSTISQALIFVTRSRSWSFVERPGWLLVIAFAIAQLVATLIAVYANWSFAAVEGIGWGWAGVIWLYNIVFYFPLDIIKFFIRYALSGRAWDLVIERRIAFTRQKDFGKEQRELQWAHAQRTLHGLEVPDTKMFGDRTNVTELNQMAEEAKRRAEIARLRELHTLKGHVESVVRLKGLDIETIQQAYTV

# >HHA13

MGEEKPEALEAVLKETVDLESIPIEEVFENLRCSKDGLTTSAAEQRLIIFGHNKLEEKKESKFLKFLGFMWNPLSWVMEAAAIMAIALANGGGKPPDWQDFVGIITLLIINSTISFIEENNAGNAAAALMARLAPKAKILRDGKWNEEDASMLVPGDIISIKLGDIIPADARLLDGDPLKIDQSALTGESLPVTKGPGDGVYSGSTCKQGEIEAVVIATGVHTFFGKAAHLVDSTNQVGHFQKVLTAIGNFCICSIAVGMVIEIIVMFPIQDRQYRPGIDNLLVLLIGGIPIAMPTVLSVTMAIGSHRLAQQGAITKRMTAIEEMAGMDVLCSDKTGTLTLNKLTVDKNLIDVFAKGVDADTVVLMAARASRTENQDAIDAAIVGMLADPKEARADIQELHFLPFNPTDKRTALTYLDSQGKMHRVSKGAPEQILNLAHNKSDIERRVHAVIDKFADRGLRSLAVAYQEVPEGRKESPGGPWQFIGLMPLFDPPRHDSAETIRRALNLGVNVKMITGDQLAIGKETGRRLGMGTNMYPSSALLGQNKDESIAALPIDELIEKADGFAGVFPEHKYEIVKRLQARKHICGMTGDGVNDAPALKKADIGIAVADATDAARSASDIVLTEPGLSVIISAVLTSRAIFQRMKNYTIYAVSITIRIVLGFMLLALIWKFDFPPFMVLIIAILNDGTIMTISKDRVKPSPLPDSWKLAEIFTTGVVLGSYLAMMTVIFFWAAYKTDFFPRTFGVPTLEKTAHDDFRKLASAIYLQVSTISQALIFVTRSRSWSFVERPGWLLVIAFAIAQLVATLIAVYANWSFAAVEGIGWGWAGVIWLYNIVFYFPLDIIKFFIRYALSGRAWDLVIERRIAFTRQKDFGKEQRELQWAHAQRTLHGLEVPDTKMFGDRTNVTELNQMAEEAKRRAEIARLRELHTLKGHVESVVRLKGLDIETIQQAYTV
