# Supplementary material for: Genome-Wide Identification and Analysis of P-Type Plasma Membrane H+-ATPase Sub-Gene Family in Sunflower and the Role of HHA4 and HHA11 in the Development of Salt Stress Resistance
Source: Genes (Basel). 2020 Mar 27;11(4):361. doi: 10.3390/genes11040361 (PMC7231311; doi:10.3390/genes11040361)
Supplement: Supplementary file 1 [file genes-11-00361-s001.zip › Supplementary File 5.docx]

Supplementary File 5：Protein sequences of PM H^+^-ATPases derived from other species.

# N. plumbaginifolia

## >PMA1, Q08435

MGEEKPEVLDAVLKEAVDLENIPIEEVFENLRCTKEGLTATAAQERLAIFGYNKLEEKKDSKLLKFLGFMWNPLSWVMEAAAIMAIALANGGGKPPDWQDFVGIITLLIINSTISFIEENNAGNAAAALMARLAPKAKVLRDGRWKEEDAAVLVPGDIISIKLGDIIPADARLLEGDPLKIDQSALTGESLPVTKGPGDGVYSGSTCKQGEIEAIVIATGVHTFFGKAAHLVDSTNQVGHFQKVLTAIGNFCICSIAVGMIIEIIVMYPIQHRAYRPGIDNLLVLLIGGIPIAMPTVLSVTMAIGSHRLAQQGAITKRMTAIEEMAGMDVLCSDKTGTLTLNKLTVDKNLIEVFAKGVDADMVVLMAARASRTENQDAIDAAIVGMLADPKEARAGIREIHFLPFNPTDKRTALTYLDGEGKMHRVSKGAPEQILNLAHNKSDIERRVHAVIDKFAERGLRSLGVAYQEVPEGRKESAGGPWQFIGLLPLFDPPRHDSAETIRRALNLGVNVKMVTGDQLAIGKETGRRLGMGTNMYPSSALLGQTKDESISALPIDELIEKADGFAGVFPEHKYEIVKRLQARKHICGMTGDGVNDAPALKKADIGIAVDDATDAARSASDIVLTEPGLSVIISAVLTSRAIFQRMKNYTIYAVSITIRIVLGFMLLALIWKFDFPPFMVLIIAILNDGTIMTISKDRVKPSPLPDSWKLAEIFTTGIVLGGYLAMMTVIFFWAAYKTNFFPHVFGVSTLEKTATDDFRKLASAIYLQVSIISQALIFVTRSRSWSFVERPGFLLVIAFVIAQLVATLIAVYANWSFAAIEGIGWGWAGVIWIYNLVFYIPLDIIKFFIRYALSGRAWDLVFERRIAFTRKKDFGKEQRELQWAHAQRTLHGLQVPDTKLFSEATNFNELNQLAEEAKRRAEIARLRELHTLKGHVESVVKLKGLDIETIQQAYTV

## >PMA2, Q42932

MGEKPEVLDAVLKETVDLENIPIEEVFENLRCTKEGLSGPAAQERLAIFGYNKLEEKKESKFLKFLGFMWNPLSWVMEAAAIMAIALANGGGKPPDWQDFVGIITLLVINSTISFIEENNAGNAAAALMARLAPKAKVLRDGKWDEQDAAILVPGDIISIKLGDIIPADARLLEGDPLKIDQSALTGESLPVTKGPGDGVYSGSTCKQGEIEAVVIATGVHTFFGKAAHLVDSTNQVGHFQKVLTAIGNFCICSIAVGMIIEIIVMYPIQHRKYRPGIDNLLVLLIGGIPIAMPTVLSVTMAIGSHRLAQQGAITKRMTAIEEMAGMDVLCSDKTGTLTLNKLTVDKNLVEVFAKGVDADTVVLMAARASRTENQDAIDTAIVGMLSDPKEARAGIREIHFLPFNPTDKRTALTYLDGEGKMHRVSKGAPEQILNLAHNKSDIERRVHSVIDKFAERGLRSLGVAYQEVPEGRKESTGGPWQFIGLLPLFDPPRHDSAETIRRALNLGVNVKMITGDQLAIGKETGRRLGMGTNMYPSSALLGQTKDESIASLPIDELIEKADGFAGVFPEHKYEIVKRLQARKHICGMTGDGVNDAPALKKADIGIAVDDATDAARSASDIVLTEPGLSVIISAVLTSRAIFQRMKNYTIYAVSITIRIVLGFMLLALIWKFDFPPFMVLIIAILNDGTIMTISKDRVKPSPLPDSWKLAEIFTTGVVLGGYLAMMTVIFFWAAYETDFFPRVFGVSTLQKTATDDFRKLASAIYLQVSTISQALIFVTRSRSWSFVERPGLLLVVAFLIAQLVATLIAVYANWAFAAIEGIGWGWAGVIWLYNLVFYFPLDIIKFLIRYALSGRAWDLVLEQRIAFTRKKDFGKEQRELQWAHAQRTLHGLQVPDTKLFSEATNFNELNQLAEEAKRRAEIARQRELHTLKGHVESVVKLKGLDIETIQQSYTV

## >PMA3, Q08436

MGEKPEVLDAVLKETVDLENIPIEEVFENLRCTKEGLTATAAQERLSIFGYNKLEEKKESKFSKFLGFMWNPLSWVMEAAAIMAIALANGGGKPPDWQDFVGIITLLIINSTISFIEENNAGNAAAALMARLAPKAKVLRDGKWKEEDAAVLVPGDIISIKLGDIIPADARLLEGDPLKIDQSALTGESLPVTKGPGDGVYSGSTCKQGEIEAVVIATGVHTFFGKAAHLVDSTNQVGHFQKVLTAIGNFCICSIAVGMIIEIIVMYPIQHRKYRPGIDNLLVLLIGGIPIAMPTVLSVTMAIGSHRLAQQGAITKRMTAIEEMAGMDVLCSDKTGTLTLNKLTVDKYLIEVFARGVDADTVVLMAARASRTENQDAIDAAIVGMLADPKEARAGIREIHFLPFNPTDKRTALTYLDGEGKMHRVSKGAPEQILHLAHNKSDIERRVHAVIDKFAERGLRSLAVAYQEVPEGRKESAGGPWQFIALLPLFDPPRHDSAETIRRALNLGVNVKMITGDQLAIGKETGRRLGMGTNMYPSSALLGQTKDESISALPVDELIEKADGFAGVFPEHKYEIVKRLQARKHICGMTGDGVNDAPALKKADIGIAVDDATDAARSASDIVLTEPGLSVIISAVLTSRAIFQRMKNYTIYAVSITIRIVLGFMLLALIWQFDFPPFMVLIIAILNDGTIMTISKDRVKPSPLPDSWKLAEIFTTGVVLGGYLAMMTVIFFWAAYKTNFFPRVFGVSTLEKTATDDFRKLASAIYLQVSTISQALIFVTRSRSWSFMERPGLLLVVAFFIAQLVATLIAVYANWSFAAIEGIGWGWAGVIWLYNIVFYIPLDLXXFLIRYALSGKAWDLVIEQRIAFTRKKDFGKEQRELQWAHAQRTLHGLQVPDPKIFSETTNFNELNQLAEEAKRRAEIARLRELHTLKGHVESVVKLKGLDIETIQQAYTV

## >PMA4, Q03194

MAKAISLEEIKNETVDLEKIPIEEVFEQLKCTREGLSADEGASRLQIFGPNKLEEKNESKILKFLGFMWNPLSWVMEAAAVMAIALANGDGKPPDWQDFIGIICLLVINSTISFIEENNAGNAAAALMAGLAPKTKVLRDGRWSEQEAAILVPGDIISVKLGDIIPADARLLEGDPLKIDQSALTGESLPVTKNPGDEVFSGSTCKQGELEAVVIATGVHTFFGKAAHLVDSTNNVGHFQKVLTAIGNFCICSIAIGMLVEIIVMYPIQHRKYRDGIDNLLVLLIGGIPIAMPTVLSVTMAIGSHRLSQQGAITKRMTAIEEMAGMDVLCSDKTGTLTLNKLSVDRNLVEVFAKGVDKEYVLLLAARASRVENQDAIDACMVGMLADPKEARAGIREVHFLPFNPVDKRTALTYIDNNNNWHRASKGAPEQILDLCNAKEDVRRKVHSMMDKYAERGLRSLAVARRTVPEKSKESPGGRWEFVGLLPLFDPPRHDSAETIRRALNLGVNVKMITGDQLAIAKETGRRLGMGTNMYPSASLLGQDKDSAIASLPIEELIEKADGFAGVFPEHKYEIVKKLQERKHIVGMTGDGVNDAPALKKADIGIAVADATDAARGASDIVLTEPGLSVIISAVLTSRAIFQRMKNYTIYAVSITIRIVFGFMFIALIWKYDFSAFMVLIIAILNDGTIMTISKDRVKPSPMPDSWKLKEIFATGVVLGGYQALMTVVFFWAMHDTDFFSDKFGVKSLRNSDEEMMSALYLQVSIISQALIFVTRSRSWSFLERPGMLLVIAFMIAQLVATLIAVYANWAFARVKGCGWGWAGVIWLYSIIFYLPLDIMKFAIRYILSGKAWNNLLDNKTAFTTKKDYGKEEREAQWALAQRTLHGLQPPEATNLFNEKNSYRELSEIAEQAKRRAEMARLRELHTLKGHVESVVKLKGLDIETIQQHYTV

## >PMA5, AAV49159.1

MASNLSLEDIRNEQVDLENIPVEEVFQILKCSREGLTNEEGQNRLQIFGHNKLEEKKENKVLKFLGFMWNPLSWVMEAAAIMSIALANGGGKPPDWPDFVGIVVLLIINSTISFIEENNAGNAAAALMANLAPKTKILRDGKWSEEDASILVPGDLISVKLGDIIPADARLLEGDPLKIDQAALTGESLPVTKQPGDQVFSGSTVKQGEIEAVVIATGVHTFFGKAAHLVDSTNQVGHFQKVLTSIGNFCICSIVLGIVIEILVMWPIQKRKYRDGIDNLLVLLIGGIPIAMPTVLSVTMAIGSHRLSQQGAITKRMTAIEEMAGMDVLCSDKTGTLTLNKLTVDKNLIEVFPKNADKDTVMLLAARASRVENQDAIDACIVNMLNDPKEAREGIQEVHFFPFNPVDKRTAITYIDDSGNWHRASKGAPEQIIELCDLKGDVLKKAHEIIDNFANRGLRSLGVARQTVPEKNKDSAGSPWEFVGLLPLFDPPRHDSAETIRKALELGVNVKMITGDQLAIGKETGRRLGMGTNMYPSSALLGDHKDESIASIPVEELIEQADGFAGVFPEHKYEIVKKLQERKHICGMTGDGVNDAPALKKADIGIAVDDATDAARSASDIVLTEPGLGVIVSAVLTSRAIFQRMKNYTIYAVSITIRVVMGFMLIALIWEFDFPPFMVLIIAILNDGTIMTISKDRVKPSPLPDSWKLNEIFATGIVLGTYQAIMSVVFFYLAADTDFFTENFHVKSIRDNPYELTAAVYLQVSIISQALIFVTRSRSWSFLERPGFLLVTAFLQAQFVATLITVYANWNFARIHGIGWGWAAIIWIYTIITYIPLDILKFISRYALSGEAWNSIIQNKTAFTTKKDYGKGEREAQWAVAQRTLHGLQTAESNGLFHDKNYRELNEIAEQAKRRAEVAKYTHEP

## >PMA6, Q9SWH2

MADQKGTVSLDDIKKENVDLERIPVDDVFRILISSKEGLASDEAERRLVVFGANKLEEKKENKILKFFGFMWNPLSWVMEVAAVIAIALANGQNRPPDWQDFLGIVVLLVINSTVSFIEENNAGNAAAALMAGLAPKSKVLRDGSWKEMDAAMLVPGDVISIKLGDILPADARLLEGDPLKIDQSALTGESLPVTKHPGEGVFSGSTCKQGEIEAVVIATGISTFFGKAAHLVDSTNNVGHFQKVLTAIGNFCICSILVGIVIEILVMYPIQHRKYRDGIDNLLVLLIGGIPIAMPTVLSVTMAIGSHKLSQQGAITKRMTAIEEMAGMDVLCSDKTGTLTLNKLEVDKNLVEVFAKDIDQDTVILLGARASRVENQDAIDACIVGMLADAKEARAGIQEVHFLPFNPVDKRTAITYIDANGNWHRVSKGAPEQIIELCGLSEDVKRRAHSVIDKFADRGLRSLAVAQQTVPERTKESPGGPWLFVGLLPLFDPPRHDSAETIRRALVLGVNVKMITGDQLAIAKETGRRLGMGTNMYPSSSLLGQHKDENTANLPVDELIEMADGFAGVFPEHKYEIVKKLQERKHICGMTGDGVNDAPALKKADIGIAVADATDAARGASDIVLTEPGLSVIVSAVLTSRAIFQRMKNYTIYAVSITIRIVLGFMLIALIWKFDFSPFMVLIIAILNDGTIMTISKDKVKPSPMPDSWKLREIFATGIVLGTYLAVMTVIFFWAAHQSNFFSDKFGVRSIRDNVHELNSALYLQVSIVSQALIFVTRSRSWSYVERPGLLLLAAFAIAQLVATLIAVYANWGFARIHGIGWGWAGVIWLYSIIFYIPLDILKFAIRYTLSGRAWNNMMENKVAFTSKKDYGKGEREAQWALAQRTLHGLQAPDSSQVFDNKSYKELSEIAEQAKRRAEVARLRELHTLKGHVESVVKLKGLDIETIQQHYTV

## >PMA8, Q9SWH1

MGTENTTTTLEAINNETVDLENIPIKEVFENLKCTEEGLNSTEVEKRLNVFGHNKLEEKKESKILKFLGFMWNPLSWVMEVAAIMALFLPHGKHKEIDYQDFVGIVALLIINSTISFIEENNAGNAAAALMARLAPKAKVLRDGKWSEEDAAVLVPGDIISIKLGDIIPADARLLNGDPLKIDQSALTGESLPVTKNPGDGVYSGSTCKQGEIEAVVIATGVHTFFGKAAHLVENTTHVGHFQKVLASIGNFCICSIAIGMVIELIVIFGGQHRPPREAIDSLLVLLIGGIPIAMPTVLSVTMAIGSHRLSQQGAITKRMTAIEEMAGMDVLCSDKTGTLTLNKLSVDKNLIEVFAKDVEKDTVVLMAARASRLENQDAIDTAIVSMLADPKEARAGITEIHFLPFNPTDKRTALTYVDSAGKMHRVSKGAPEQILNLAWNKSDIQNRVHTVIEKFAERGLRSLAVARQEVPAGTKDSPGGPWEFVGLLPLFDPPRHDSAETIRRALELGVSVKMITGDQLAIGKETGRRLGMGTNMYPSSFLLGEQKDASAAVLPIEELIESADGFAGVFPEHKYEIVRILQSRKHICGMTGDGVNDAPALKKADIGIAVADSTDAARGASDIVLTEPGLSVIIHAVLTSRAIFQRMKNYTIYAVSITIRIVLGFMLLTAFWRFNFPPFMVLVIAILNDGTIMTISKDRVKPSPLPDSWKLSEIFATGIVIGSYLALMTALFFYLMFETSFFAHAFNVEDFNKRIPANKVITDSLNAKLASAVYLQVSTISQALIFVTRSRGWSFMERPGLLLVAAFIVAQMVATFMSAMVTSVKFAGIEKIGWKWTGVIWLFNIVTYFLLDPIKFAVRYALSGRAWGLLLNQKTAFTNRKDFGKEAREAAWAAEQRTIHGLQSVETRTFPENYTFRDISLMAEEAKRRAEIARLRELHTLKGRVESFAKLRGLDVDHVNPHYTV

## >PMA9, Q9SWH0

MAEDLEKPLLGPENLREEGIDLENLPLEEVFEQLITSKEGLSAEDAERRLKIFGPNKLEEKRENKFLKFLRFMWNPCSWVMEAAAIMAIGLANGGGQGPDWQDFVGIVCLLLINSTISFIEENNAGNAAAALMARLAPRTKVLRDGRWQEKDAAILVPGDIISIKLGDIIPADARLLEGDPLKVDQSALTGESLPITKKTGDEVFSGSTCKHGEIEAVVIATGVNSFFGKAAHLVDSTEASGHFQKVLASIGNFCICSIAVGMIFEIIIMYAVQRRSYRTGINNLLVLLIGGIPIAMPTVLSVTLAIGSHRLSQQGAITKRMTAIEEMAGMDVLCSDKTGTLTLNRLTVDRNLIEVFQRDMDKDMVVLLAARASRLENQDAIDAAIINVLADPKEARANIRQVHFLPFNPVDKRTAITYIDSDGKWYRASKGAPEQILDLCQEKQQISAKVHTIIDRFAERGLRSLAVAFQEIPENSKESPGGPWAFCGLLPLFDPPRHDSAETIRRALNLGVCVKMITGDQLAIAKETGRRLGMGTNMYPSFSFFGRDKDENEALPVDELIEKADGFAGVFPEHKYEIVKILQANGHIVGMTGDGVNDAPALKKADIGIAVADATDAARSASDLVLTEPGLSVIVSAVLTSRAIFQRMKNYTIYAVSITIRIVLGFMLLALIWEYDFPPFMVLIIAILNDGTIMTISKDRVKPSPGPDSWKLNEIFATGIVIGTYLALVSVLFYWLADSTLFFETHFHVKSISGNTEEISAAIYLQVSIISQALIFVTRSQSWSFIERPGLLLMFAFVVAQLVATLIAVYAHIEFASISGIGWGWAGVIWLYSLIFYIPLDIIKFIVRYGLTGDAWNLLFDKKTAFTSKKDYGREDRETKWVLSVRTLQGVISPEFETKSRRPSMIAEQAKRRAEITRLRELYTLRGHIESVARLKNLDFNKIQTAHTV

# >O. sativa

## >OSA1, Q43001

MAEDKGGLDAVLKESVDLENIPIEEVFQNLKCCRQGLTSEEAQLRLQLFGPNKLEEKEESKFLKFLGFMWNPLSWVMEAAAIMAIALANGGGKPPDWQDFVGIITLLLINSTISFIEENNAGNAAAALMARLAPKAKVLRNGSWTEEEAAILVPGDIISIKLGDIIPADARLLEGDPLKIDQSALTGESLPATKGPGDGVYSGSTVKQGEIEAVVIATGVHTFFGKAAHLVDSTNQVGHFQKVLTAIGNFCICSIAVGMFVEIIVMYPIQHRPYRPGIDNLLVLLIGGIPIAMPTVLSVTMAIGSHRLSQQGAITKRMTAIEEMAGMDVLCSDKTGTLTLNKLTVDKNLIEIFERGVTQDQVILMAARASRTENQDAIDTALVGMLADPKEARAGIQEVHFLPFNPTDKRTALTYIDSDGKMYRVSKGAPEQILNLAHNKTQIERRVHAVIDKFAERGLRSLAVAYQEVPDGRKESPGGPWRFVALLPLFDPPRHDSAETIRRALNLGVNVKMITGDQLAIGKETGRRLGMGTNMYPSSALLGQNKDESVAALPVDDLIEKADGFAGVFPEHKYEIVKRLQARKHICGMTGDGVNDPPALKKADIGIAVADATDAARSASDIVLTEPGLSVIISAVLTSRAIFQRMKNYTIYAVSITIRIVFGFMLLALIWEFDFPPFMVLIIAILNDGTIMTISKDLVKPSPLPDSWKLAEIFTTGVVLGGYLAMMTVISSGLHTRPTFSLGSFTSKALRRQLQDDYQKLASAVYLQVSTISQALIFVTRSRSWSFIERPGFLLVFAFFVAQLIATLIAVYANWAFTSIKGIGWGWAGIVWLYNLVFYFPLDIIKFLIRYALSGKAWDLVIEQRIAFTRKKDFGKEERELKWAHAHRTLHGLQPPDAKPFPEKTGYSELNQMAEEAKRRAEIARLRELHTLKGHVESVVKLKGLDIDTIHQSYTV

## >OSA2, Q43002

MAEKGDNLEAVLNESVDLENIPLEEVFEHLRCNREGLTSANAEQRLNLFGLNRLEEKKESKFLKFLGFMWNPLSWVMEAAAIMAIVLANGGGKPPDWQDFVGIITLLIINSTISFIEENNAGNAAAALMARLAPKAKVLRNGRWSEEEAAILVPGDIISVKRGDIIPADARLLEGDPLKIDQSALTGESLPVTKGPGDGVYSGSTCKQGEIEAVVIATGVHTFFGKAAHLVDSTNQVGHFQKVLTAIGNFCICSIAIGMVVEIIVMYPIQHRDYRPGIDNLLVLLIGGIPIAMPTVLSVTMAIGSHRLAQQGAITKRMTAIEEMAGMDVLCSDKTGTLTLNKLTVDKSLIEVFQRGVDQDTVILMAARASRTENQDAIDATIVGMLADPKEARAGIQEVHFLPFNPTDKRTALTYIDGEGKMHRVSKGAPEQILNLAHNKTEIERRVRAVIDKFAERGLRSLAVQYHQVPDGRKESPGGPWQFVGLLPLFDPPRHDSAETIRRALNLGVNVKMITGDQLAIGKETARRLGMGTNMYPSSALLGQDKDESIVALPVDELIEKADGFAGVFPEHKYEIVKRLQARKHICGMTGDGVNDAPALKKADIGIAVDDSTDAARSASDIVLTEPGLSVIISAVLTSRAIFQRMKNYTIYAVSITIRIVLGFMLLALIWKFDFPPFMVLIIAILNDGTIMTISKDRVKPSPQPDSWKLSEIFATGVVLGSYLAMMTVIFFWVAYKTDFFPRVFHVESLQKTAQDDFQKLASAVYLQVSTISQALIFVTRSRSWSFVERPGFLLVFAFFVAQLIATLIAVYANWGFASIKGIGWGWAGVIWLYNIVFYLPLDIIKFLIRYALSGRAWDLVLEQRIAFTRKKDFGTQENQLKWATAQRTIHGLQPAATAAVFRDMTSYNDLNQLAEEARRRAEIARLRELTTLKGRMESVVKQKGLDLETIQQSYTV

## >OSA3, [XP_015620234.1](https://www.ncbi.nlm.nih.gov/protein/1002313721)

MAEKEGNLDAVLKEAVDLENIPLEEVFENLRCSREGLTTQQAQQRLEIFGPNKLEEKEESKFLKFLGFMWNPLSWVMEAAAIMAIALANGGGKPPDWQDFVGIITLLVINSTISFIEENNAGNAAAALMARLAPKAKVLRDGRWTEEEAAILVPGDIVSIKLGDIIPADARLLEGDPLKIDQSALTGESLPVTKGPGDGVYSGSTVKQGEIEAIVIATGVHTFFGKAAHLVDSTNQVGHFQKVLTAIGNFCICSIAVGMFVEIIVMYPIQHRAYRPGIDNLLVLLIGGIPIAMPTVLSVTMAIGSHRLSQQGAITKRMTAIEEMAGMDVLCSDKTGTLTLNKLTVDKNLIDVFERGITQDQVILMAARASRTENQDAIDTAIVGMLADPKEARAGIQEVHFLPFNPTDKRTALTYIDGDGKMYRVSKGAPEQILHLAHNKPEIERRVHAVIDKFAERGLRSLAVAYQEVPEGTKESPGGPWHFVGLMPLFDPPRHDSAETIRRALNLGVNVKMITGDQLAIGKETGRRLGMGTNMYPSSALLGQNKDESIAALPVDDLIEKADGFAGVFPEHKYEIVKRLQARKHICGMTGDGVNDAPALKKADIGIAVADATDAARSASDIVLTEPGLSVIISAVLTSRAIFQRMKNYTIYAVSITIRIVLGFMLLALIWKFDFPPFMVLIIAILNDGTIMTISKDRVKPSPLPDSWKLAEIFTTGVVLGGYLAMMTVIFFWAAYKTDFFPRIFHVESLEKTAQDDFQKLASAVYLQVSTISQALIFVTRSRSWSFVERPGFLLVFAFLVAQLIATLIAVYADWAFTSIKGIGWGWAGIVWLYNLIFYFPLDIIKFLIRYALSGKAWDLVIEQRIAFTRKKDFGKEERELKWAHAQRTLHGLQPPDAKMFSEKAGYNELNQMAEEAKRRAEIARLRELHTLKGHVESVVKLKGLDIETIQQSYTV

## >OSA4, AJ440002

MSVSLEDLKKENVDLESIPIQEVFAVLKSSPQGLTSADGNGRLEIFGRNKLEEKKESKLLKFLGFMWNPLSWVMEAAAIMAIALANGGGRPPDWQDFVGIVTLLFINSTISFIEENNAGNAAAALMASLAPQTKARRACALLRDGKWSEQDAAILVPGDIISIKLGDIIPADARLMEGDPLKIDQSALTGESLPVNKMPGDSIYSGSTCKQGEIEAVVIATGVHTFFGKAAHLVDSTNNVGHFQKVLTAIGNFCICSIAAGMLIEIIVMYPIQHRQYRDGIDNLLVLLIGGIPIAMPTVLSVTMAIGSHRLSQQGAITKRMTAIEEMAGMDVLCSDKTGTLTLNKLTVDKNMIEDPFVKDLDKDAIVLYAAKASRTENQDAIDASIVGMLADPSEARAGIQEVHFMPFNPVDKRTAITYIDTKDGSWHRISKGAPEQIIELCRLRDDVSRRVHAIIDKFADRGLRSLAVARQKVPEGSKDAPGTPWQFLAVLPLFDPPRHDSSETIRRALNLGVNVKMITGDQLAIGKETGRRLGMGTNMYPSSSLLKDGDTGGLPVDELIEKADGFAGVFPEHKYEIVRRLQERKHICGMTGDGVNDAPALKKADIGIAVADATDAARGASDIVLTEPGLSVIISAVLTSRAIFQRMKNYTIYAVSITIRVVLGFLLLALIWRFDFAPFMVLIIAILNDGTIMTISKDRVKPSPLPDAWRLQEIFATGIVLGTYLALATVLFFWAVRDTDFFTRTFGVHPIGGSTEELMAAVYLQVSIISQALIFVTRARSWFFVERPGLLLVGAFLIAQLMATLIAVYANWPFAKMKGIGWSWGMVIWLFSIVTFFPLDIFKFAIRYFLSGKAWNNAFDNKTAFANELDYGKSKREAQWAIAQRSLHGLQQAETSTALFDDNKDYLELSEIAEQAKRRAEIARLRELHTLKGHVESVVKLKGLDIDTIQNHYTV

## >OSA5, AJ440216

MAATASSTADALEQIKNEAVDLEHIPLEEVFQHLKCTREGLTNAEGDARTQVFGPNKLEEKKESKILKFLGFMWNPLSWVMEVAAIMAIALANGGGRPPDWQDFVGIIALLLINSTISYWEESNAGSAAAALMKNLAPKTKVLRDGRWSETDAFVLVPGDVINVKLGDIVPADARLLDGDPLKIDQSALTGESLPVTKLPGDCVYSGSTCKQGEIDAVVIATGVHTFFGKAAHLVDTTNQVGHFQKVLRAIGNFCIGAIAIGMAVEVIVMYLIQHRLYRDGIDNLLVLLIGGIPIAMPTVLSVTMAIGSHRLSDQGAITKRMTAIEEMAAMDVLCSDKTGTLTLNKLSVDRGLIEVFVQGVAKDEVILLTARASRVENQDAIDTAMVGMLDDPKEARAGIREEHFLPFNPVDKRTALTYVDLADGSWHRVSKGAPEQILDLCKCRQDVRSKVHAIIDRYADRGLRSLAVARQEVPERRKDGPGGPWEFVGLLPLLDPPRHDSAETIRRALHLGVNVKMITGDQLAIAKETGRRLGMGVNMYPSSALLGQSKDESIASVPVDELIKKADGFAGVFPEHKYEIVKKLQEMKHICGMTGDGVNDAPALKRADIGIAVADATDAARSASDIVLTQPGLSVIISAVLTSRAIFQRMKNYTIYAVSITIRIVLGFMLIALIWKFDFSPFMILVIAILNDGTIMTISKDRVKPSPHPDSWKLPEIFITGIVYGTYLAVMTVLFFWAMRSTDFFTSTFHVKPLMEKDEMMSALYLQVSIISQALIFVTRSRSWCFVERPGMLLCGAFVAAQIIATLVTVYATLGFAHIKGIGWGWAGVIWLYSIVTFLPLDIFKFAVRYALSGRAWDTLIEHKIAFTSKKDYGRGEREAQWATAQRTLHGLQTPEMGTTSAASYRELSEIAEQAKRRAEVARLRELSTLKGQMESTVRLKGLDMDNVQHHYTV

## >OSA6, AJ440217

MASISLEDVRNETVDLETIPVEEVFQHLKCSKQGLSAAEGQNRLNIFGPNKLEEKTESKLLKFLGFMWNPLSWVMEAAAIMAIVLANGGGRPPDWQDFVGIVVLLVINSTISFIEENNAGNAAAALMAGLAPKTKVLRDGKWQEQDASILVPGDIISIKLGDIIPADARLLEGDPLKVDQAALTGESMPVNKHAGQGVFSGSTVKQGEIEAVVIATGVHTFFGKAAHLVDSTNNIGHFQLVLTAIGNFCIISIGVGMIIEIIVMYPIQHRAYRDGIDNLLVLLIGGIPIAMPTVLSVTMAIGSHRLSQQGAITKRMTAIEEMAGMDVLCSDKTGTLTLNKLTVDKTLIEVYGRGLDKDSVLLYAARASRVENQDAIDTCIVGMLADPKEARAGIKEVHFLPFNPVEKRTAITYIDGNGEWHRISKGAPEQIIELCKMSKDAEKKVHTLIDQYADRGLRSLGVSYQKVPEKSKESEGEPWQFVGLLPLFDPPRHDSAETIRRALHLGVNVKMITGDQLAIGKETARRLGMGTNMYPSTTLLGDKSSEMSGLPIDELIEKADGFAGVFPEHKYEIVKRLQDRKHICGMTGDGVNDAPALKKADIGIAVDDATDAARSASDIVLTEPGLSVIVSAVLTSRAIFQRMKNYTIYAVSITIRIVLGFMLVALLWKFDFAPFMVLIIAILNDGTIMTISKDRVKPSPTPDSWKLKEIFATGIVLGTYMALITALFFYLAHDTDFFTETFGVRSIKTNEKEMMAALYLQVSIISQALIFVTRSRSWSFVERPGALLLVATCIAVYAEWEFCKMQGIGWGLGGAIWAFSVVTYFPLDVLKFIIRYALSGRAWNNINNKTAFVNKNDYGKGEREAQWATAQRTLHGLNQSSTSSDLFNDKTGYRELSEIAEQAAKRAEVARLRELHTLKGHVESVVKLKGLDIDTIQQSYTV

## >OSA7, AJ440218

MTGRAHPPIVIENIPIEEVFEQLKCTREGLSSEEGNRRIEMFGPNKLEEKKESKILKFLGFMWNPLSWVMEMAAIMAIALANGGGKPPDWEDFVGIIVLLVINSTISFIEENNAGNAAAALMANLAPKTKVLRDGRWGEQEAAILVPGDIISIKLGDIVPADARLLEGDPLKIDQSALTGESLPVTKNPGDEVFSGSTCKQGEIEAVVIATGVHTFFGKAAHLVDSTNQVGHFQTVLTAIGNFCICSIAVGIVIEIIVMFPIQHRAYRSGIENLLVLLIGGIPIAMPTVLSVTMAIGSHKLSQQGAITKRMTAIEEMAGMDVLCSDKTGTLTLNKLSVDKNLVEVFTKGVDKDHVLLLAARAFRTETQDAIDAAMVGMLADPKEARAGIREVHFLPFNPVDKGTALTYIDADGNWHRASKGAPEQILTLCNCKEDVKRKVHAVIDKYAERGLRSLAVARQEVPEKSKESAGGPWQFVGLLPLFDPPRHDSAETIRKALHLGVNVKMITGDQLAIGKETGRRLGMGTNMYPSSALLGQNKDASLEALPVDELIEKADGFAGVFPEHKYEIVKRLQEKKHIVGMTGDGVNDAPALKKADIGIAVADATDAARSASDIVLTEPGLSVIISAVLTSRCIFQRMKNYTIYAVSITIRIVLGFLLIALIWKYDFSPFMVLIIAILNDGTIMTISKDRVKPSPLPDSWKLKEIFATGIVLGSYLALMTVIFFWAMHKTDFFTDKFGVRSIRNSEHEMMSALYLQVSIVSQALIFVTRSRSWSFIERPGLLLVTAFMLAQLVATFLAVYANWGFARIKGIGWGWAGVIWLYSIVFYFPLDIFKFFIRFVLSGRAWDNLLENKIAFTTKKDYGREEREAQWATAQRTLHGLQPPEVASNTLFNDKSSYRELSEIAEQAKRRAEIARLRELNTLKGHVESVVKLKGLDIDTIQQNYTV

## >OSA8, AJ440219

MEVANAMDAITKETVDLEHIPVEEVLDHLKCTREGLTSEVAQQRIHSFGYNKLEEKQESKLLKFLGFMWNPLSWVMEAAAIMAIALAHGGRDARGKRMRIDYHDFVGIVLLLFINSTISFMEENNAGNAAAALMARLAPKAKVLRDGTWDELDASLLVPGDIISVKLGDIIPADARLLEGDPLKIDQSALTGESLPVTKHPGDGIYSGSTCKQGEIEAVVIATGIHTFFGKAAHLVESTTHVGHFQKVLTSIGNFCICSIAAGMVIELLVMYAVHERKYRQIVDNLLVLLIGGIPIAMPTVLSVTMAIGSHKLAQQGAITKRMTAIEEMAGMDVLCSDKTGTLTLNKLSVDKNLIEVFEKGIEKDDVVLMAARASRLENQDAIDFAIVSMLPDPKEARAGIQEVHFLPFNPTDKRTALTYLDAEGKMHRVSKGAPEQILNLASNKCEIERKVHHVIGNFAERGLRSLAVAYQEVPEGTKESPGGPWQFVGLLPLFDPPRHDSAETIRRALDLGVSVKMITGDQLAIGKETGRRLGMGTNMYPSSSLLGDRKDGDIAVLPVDELIEQADGFAGVFPEHKYEIVQRLQARKHICGMTGDGVNDAPALKKADIGIAVADATDAARSASDIVLTEPGLSVIISAVLTSRAIFQRMKNYTIYAVSITVRIVLGFLLLACFWKFDFPPFLVLVIAILNDGTIMTISKDKVKPSPYPDSWKLTEIFATGVIIGAYLAVTTVLFFWAAYKTQFFVHLFNVDTLNINKKLASAVYLQVSTISQALIFVTRSRGWSFLERPGLLLMAAFVIAQLIATVLAAIATWEVASIRGIGWRWAGAIWVYNIVVYLLLDPMKFAVRYGLSGKAWNLVIDNKVAFTNRKDFGREARVVAWAHEQRTLHGLQSAASREKAASTELNQMAEEARRRAEITRLRELHTLKGKVESVAKLKGIDLEDVNNQHYTV

## >OSA9, AJ440220

MDEPGEPLLGLENFFDEDVDLENLPLEDVFEQLNTSQSGLSSADAAERLKLFGANRLEEKRENKIIKFLSFMWNPLSWVMEAAAVMALVLANGGSQGTDWEDFLGIVCLLIINSTISFIEENNAGDAAAALMARLALKTKVLRDEQWQELDASTLVPGDIISIRLGDIVPADARLLEGDPLKIDQSALTGESLPVTKRTGDIVFTGSTCKHGEIEAVVIATGIHSFFGKAAHLVDSTEVVGHFQKVLTSIGNFCICSIAIGAIVEVIIMFPIQHRSYRDGINNVLVLLIGGIPIAMPTVLSVTLAIGSHHLSQQGAITKRMTAIEEMAGMDVLCCDKTGTLTLNHLTVDKNLIEVFSREMDREMIILLAARASRVENQDAIDMAIINMLADPKEARSSITEVHFLPFNPVDKRTAITYVDSDGNWFRVSKGAPEQILSLCYNKDDISEKVQLIIDRFAERGLRSLAVAYQEVPEKSKHGHGGPWVFCGLLPLFDPPRHDSADTIRRALDLGVCVKMITGDHLAIAKETGRRLGMGTNMYPSASLFGRHGDGGGAAVPVEELVEKADGFAGVFPEHKYEIVRMIQGGGGHVCGMTGDGVNDAPALKKADIGIAVSDATDAARGAADIVLTEPGLSVIVSAVLTSRAIFQRMKNYTIYAVSITIRIVIGFVLLASIWEYDFPPFMVLIIAILNDGTIMTISKDRVKPSPSPDSWKLNEIFAAGVVIGTYLALVTVLFYWTVTRTTFFESHFKVRSLKQNSDEISSAMYLQVSIISQALIFVTRSQGLSFLERPGALLIGAFILAQLVATLIAVYATISFASISAIGWGWAGVIWLYSLVFYAPLDLIKIAVRYTLSGEAWNLLFDRKAAFASRRDYGGNERRPETRALSDHLLSSGWRPTRIAERAKRRAEIARLGDAHMLRAHVQSVMRLKRVDSDVIRSAQTV

## >OSA10, AJ440221

MASSLSLDDINDDSVDLSKAPVAEVFQKLKCDRKGLTGAEGESRLRLYGPNKLEEKKESKLLKFLGFMWNPAVIAAIMAIVLANGGGRPPDWQDFVGIVSLLIINSTISYIEEANAGDAAAALMAGLAPKTKLLRDGRWEEQEAAILVPGDIISIKLGDIIPADARLLEGDPLKIDQSALTGESLPVNKHPGQEVFSGSTVKQGEIEAVVIATGVRTFFGKAAHLVDSTNNVGHFQQVLTAIGNFCIISIGAGMAVEVLVMYPIQHRAYRDGIDNLLVLLIGGIPIAMPTVLSVTMAIGSHRLSQQGAITKRMTAIEEMAGMDVLCSDKTGTLTLNKLTVDKTLIEVCSKGVDKDMVLLYAARASRVENQDAIDTCIVNMLDDPKEARAGIQEVHFLPFNPVDKRTAITYIDGNGDWHRVSKGAPEQIIELCNMAADAEKKVHALIDSYADRGLRSLGVSYQQVPEKSKDSGGDPWQFIGLLPLFDPPRHDSAETIRRALHLGVNVKMITGDQLAIAKETGRRLGMGTNMYPSTTLLGDKNSQVNGLPIDELIERADGFAGVFPEHKYEIVKRLQEMSHICGMTGDGVNDAPALKKADIGIAVDDATDAARSASDIVLTEPGLSVIVSAVLTSRAIFQRMKNYTIYAVSITIRIVLGFLLIAIIWKFDFAPFMVLIIAILNDGTIMTISKDRVKPSPTPDCWKLNEIFLTGVVLGTYMALVTVLFFYLAHDTNFFTDVFGVTSIRESERELMAALYLQVSIISQALIFVTRSRSWSFVERPGFLLLFAFFAAQMVATAIAVYARWDFCRIQGIGWRWGGAVWQFSVVTYLPLDVLKFIIRYALTGGKAGDSAQKKASSPPPTSQP

# >S. tuberosum Phureja

## >PHA1, PGSC0003DMP400055772

MGEKPEVLDAVLKETVDLENIPIEEVFENLRCTKEGLTGTAAQERLAIFGYNKLEEKKESKFLKFLGFMWNPLSWVMEAAAIMAIALANGGGKPPDWQDFVGIITLLVINSTISFIEENNAGNAAAALMARLAPKAKVLRDGKWNEEDAAVLVPGDIISIKLGDIVPADARLLEGDPLKIDQSALTGESLPVTKGPGDGVYSGSTCKQGEIEAVVIATGVHTFFGKAAHLVDSTNQVGHFQKVLTAIGNFCICSIAVGMIIEIIVMYPIQHRKYRPGIDNLLVLLIGGIPIAMPTVLSVTMAIGSHRLAQQGAITKRMTAIEEMAGMDVLCSDKTGTLTLNKLTVDKNLVEVFAKGVDADTVVLMAARASRTENQDAIDTAIVGMLSDPKEARAGIREIHFLPFNPTDKRTALTYLDGEGKMHRVSKGAPEQILNLAHNKSDIERRVHAVIDKFAERGLRSLGVAYQEVPEGRKESSGGPWQFIGLLPLFDPPRHDSAETIRRALNLGVNVKMITGDQLAIGKETGRRLGMGTNMYPSSALLGQTKDESIASLPIDELIEKADGFAGVFPEHKYEIVKRLQARKHICGMTGDGVNDAPALKKADIGIAVDDATDAARSASDIVLTEPGLSVIISAVLTSRAIFQRMKNYTIYAVSITIRIVLGFMLLALIWKFDFPPFMVLIIAILNDGTIMTISKDRVKPSPLPDSWKLAEIFTTGVVLGGYLAMMTVIFFWAAYETDFFPRVFGVSTLQRTATDDFRKLASAIYLQVSTISQALIFVTRSRSWSFVERPGLLLVVAFLIAQLVATLIAVYASWSFAAIEGIGWGWAGVIWLYNLVFYFPLDIIKFLIRYALSGRAWDLVLEQRIAFTRKKDFGKEQRELQWAHAQRTLHGLQVPDTKLFSESTNFNELNQLAEEAKRRAEIARQRELHTLKGHVESVVKLKGLDIETIQQSYTV

## >PHA2, PGSC0003DMP400007331

MAKAISLEEIKNETVDLEKIPIEEVFEQLKCSREGLTSDEGANRLQIFGPNKLEEKKESKILKFLGFMWNPLSWVMEAAAIMAIALANGNGKPPDWQDFVGIVCLLVINSTISFIEENNAGNAAAALMAGLAPKTKVLRDGRWSEQEAAILVPGDIISVKLGDIVPADARLLEGDPLKIDQSALTGESLPVTKNPGDEVFSGSTCKQGELEAVVIATGVHTFFGKAAHLVDSTNNVGHFQKVLTAIGNFCICSIAVGMLIEIIVMYPIQHRKYRDGIDNLLVLLIGGIPIAMPTVLSVTMAIGSHRLSQQGAITKRMTAIEEMAGMDVLCSDKTGTLTLNKLSVDKTLVEVFVKGVDKEYVLLLAARASRVENQDAIDACMVGMLADPKEARAGIREVHFLPFNPVDKRTALTYIDNNGNWHRASKGAPEQILDLCNCKEDVRRKVHSMIDKYAERGLRSLAVARQEVPEKSKESAGGPWQFVGLLPLFDPPRHDSAETIRRALNLGVNVKMITGDQLAIAKETGRRLGMGTNMYPSASLLGQDKDSSIASLPVEELIEKADGFAGVFPEHKYEIVKKLQERKHIVGMTGDGVNDAPALKKADIGIAVADATDAARGASDIVLTEPGLSVIISAVLTSRAIFQRMKNYTIYAVSITIRIVFGFMLIALIWKYDFSAFMVLIIAILNDGTIMTISKDRVKPSPMPDSWKLNEIFATGVVLGGYQALMTVLFFWAMHDTKFFSDKFGVKDIRESDEEMMSALYLQVSIISQALIFVTRSRSWSFVERPGALLMIAFLIAQLVATLIAVYADWTFARVKGCGWGWAGVIWIFSIVTYFPLDIMKFAIRYILSGKAWNNLLDNKTAFTTKKDYGKEEREAQWALAQRTLHGLQPPEASNLFNEKNSYRELSEIAEQAKRRAEMARLRELHTLKGHVESVVKLKGLDIETIQQHYTV

## >PHA3, PGSC0003DMP400043938

MAEKPEVLDAVLKETVDLENIPIEEVFENLRCTREGLTTTAAQERLSIFGYNKLEEKKESKFLKFLGFMWNPLSWVMEAAAIMAIALANGGGKPPDWQDFVGIITLLIINSTISFIEENNAGNAAAALMARLAPKAKVLRDGKWDEEDAAVLVPGDIISIKLGDIIPADARLLEGDPLKIDQSALTGESLPVTKGPGDGVYSGSTCKQGEIEAVVIATGVHTFFGKAAHLVDSTNQVGHFQKASRLFTLLFDAMIWINVSDFYEILHYKFMYGYRQVLTAIGNFCICSIAVGMIIEIIVMYPIQHRKYRPGIDNLLVLLIGGIPIAMPTVLSVTMAIGSHRLAQQGAITKRMTAIEEMAGMDVLCSDKTGTLTLNKLTVDKALIEVFAKGIDADTVVLMAARASRIENQDAIDTAIVGMLADPQEARAGIREIHFLPFNPTDKRTALTYLDGEGKMHRVSKGAPEQILNLAHNKSDIERRVHTVIDKFAERGLRSLGVAYQEVPEGRKESAGGPWQFIALLPLFDPPRHDSAETIRRALNLGVNVKMITGDQLAIGKETGRRLGMGTNMYPSSALLGQTKDESIAALPIDELIEKADGFAGVFPEHKYEIVKRLQARKHICGMTGDGVNDAPALKKADIGIAVDDATDAARSASDIVLTEPGLSVIISAVLTSRAIFQRMKNYTIYAVSITIRIVLGFMLLALIWKFDFPPFMVLIIAILNDGTIMTISKDRVKPSPLPDSWKLAEIFTTGVILGGYLAMMTVIFFWAAYKTNFFPRVFGVSTLEKTATDDFRKLASAIYLQVSTISQALIFVTRSRSWSFVERPGLLLVFAFFVAQLVATLIAVYANWSFAAIEGIGWGWAGVIWLYNIVTYIPLDLIKFLIRYALSGKAWDLVLEQRIAFTRKKDFGKELRELQWAHAQRTLHGLQVPDPKIFSETTNFNELNQLAEEAKRRAEIARLRELHTLKGHVESVVKLKGLDIETIQQSYTV

## >PHA4, PGSC0003DMP400021001

MASNLSLEDIKNEQIDLENIPVEEVFQQLKCSKEGLSSAEGQKRVEIFGPNKLEEKKDNKLLKFLGFMWNPLSWVMECAAIMAIVLANGGGKPPDWPDFVGITVLLIINSTISFIEENSAGNAASALMANLAPKTKILRDGKWSEEEASILVPGDIISIKLGDIVPADARLLEGDPLKVDQAALTGESLPATKFPGAEVFSGSTVKQGEIEAIVIATGVHTFFGKAAHLVDSTNNVGHFQKVLTAIGNFCICSIAVGMVIEIVVMYPIQKRNYRDGIDNLLVLLIGGIPIAMPTVLSVTMAIGSHRLAQQGAITKRMTAIEEMAGMDVLCSDKTGTLTLNKLTVDKNLIEVFPKDADKDTVMLLGARASRIENQDAIDTCIVNMLGDPKEARAGIQEVHFLPFNPVEKRTAITYIDDKGNWHRASKGAPEQIIELCELKGDIKKKALEIIDDYANRGLRSLGLARQTVPEKNKESEGSPWEFVGLLPLFDPPRHDSAETIRKALELGVAVKMITGDQLAIGKETARRLGMGTNMYPSSALLGEHKDAAIASIPVDELIEKADGFAGVFPEHKYEIVKKLQDMKHICGMTGDGVNDAPALKKADIGIAVDDATDAARSASDIVLTEPGLSVIVSAVLTSRAIFQRMKNYTIYAVSITIRVVMGFMLIALIWKFDFSPFMVLIIAILNDGTIMTISKDRVVPSPLPDSWKLNEIFATGVVLGTYQAIMTVVFFYLAADTDFFTENFHVRSIRNSPNELTAALYLQVSIISQALIFVTRSRSWSFVERPGLMLVGAFFAAQLVATVLAVYADWEFARIKGVGWGWAAVIWVYTIITYLPQDVLKFIIRFGLSGRAWDTMIQNKTAFTTKKDYGRGEREAQWALAQRTLHGLQTPEAAGLFNDKNYRELSEIAEQAKRRAEVARLRELHTLKGHVESVVKLKGLDIETIQQHYTV

## >PHA5, PGSC0003DMP400013900

MAANLSLEELKNEKVDLESIPVEEVFQILKCSKEGLTKEEGQKRIEIFGPNKLEEKKENKVLKFLGFMWNPLSWVMEAAAIMSIVLANGGGKPPDWPDFVGIMVLLVINSTISFIEENNAGNAAAALMANLAPKTKVLRDGKWSEEDASLLVPGDLISVKLGDIIPADARLLEGDPLKIDQAALTGESLPVTKQPGDEVFSGSTVKQGELDAVVIATGVHTFFGKAAHLVDSTNQVGHFQKVLTAIGNFCICSILVGIVIEIVVMWPIQKRKYRDGIDNLLVLLIGGIPIAMPTVLSVTMAIGSHRLSQQGAITKRMTAIEEMAGMDVLCSDKTGTLTLNKLTVDQTLIEVFPKNADADTVMLLAARASRVENQDAIDACIVNMLGDAKLAREGIQEVHFFPFNPVDKRTAITYIDGNGDWHRASKGAPEQIIELCGLSGSVLKKSHEIIDNFANRGLRSLGVARQTVPEKDKESAGSPWEFVGLLPLFDPPRHDSAETIRKALELGVNVKMITGDQLAIGKETGRRLGMGTNMYPSSALLGDHKDESIAQIPVEELIEQADGFAGVFPEHKYEIVKKLQERKHICGMTGDGVNDAPALKKADIGIAVADATDAARGASDIVLTEPGLGVIISAVLTSRAIFQRMKNYTIYAVSITIRVVMGFMLIALIWEFDFPPFMVLIIAILNDGTIMTISKDRVKPSPLPDSWKLNEIFATGIILGTYQALMTVLFFYLAASTDFFTEKFGVRSIRENEHGLTAAVYLQVSIISQALIFVTRSRSWSFVERPGFLLVVAFFLAQFVATLITVYANWNFARIHGIGWGWAAIIWVYTIITYLPLDVLKFISRYALSGDAWDSMIQNKTAFTTKKDYGKGEREAQWAVDQRTRHGLQTAESNGLFHDKNYRELNEIAEQAKRRAEVAKYTHE

## >PHA6, [PGSC0003DMP400024836](http://solanaceae.plantbiology.msu.edu/cgi-bin/annotation_report.cgi?gene_id=PGSC0003DMP400024836)

MYPIQHRKYRDGIDNLLVLLIGGIPIAMPTVLSVTMAIGSHKLSEQGAITKRMTAIEEMAGMDVLCSDKTGTLTLNKLEVDKGLVEVFAKDMDQDTVILLGARASRVENQDAIDACIVGMLADAKEARAGIQEVHFLPFNPVDKRTAITYIDTNGNWHRVSKGAPEQIVDLCRLSEHVKRKVHSIIDKFADRGLRSLAVAQQTVPEKTKESPGAPWVFVGLLPLFDPPRHDSAETIRRALVLGVNVKMITGDQLAIGKETGRRLGMGTNMYPSSSLLGQHKDESIANLPVDELIEMADGFAGVFPEHKYEIVRKLQERKHICGMTGDGVNDAPALKKADIGIAVADATDAARSASDIVLTEPGLSVIVSAVLTSRAIFQRMKNYTIYAVSITIRIVLGFMLIALIWKFDFSPFMVLIIAILNDGTIMTISKDKVKPSPMPDSWKLREIFATGIVLGTYLAVMTVIFFWLAHQSNFFSDKFGVRSIRDNVHELNAALYLQVSIVSQALIFVTRSRSWSYVERPGFLLLAAFFVAQLVATIIAVYANWGFARIHGIGWGWAGVIWLYSIIFYIPLDFLKFAIRYILSGRAWNSMIENKVAFTNKKDYGRGEREAQWALAQRTLHGLHPPDTSQMYDNKSYNELSEIAEHAKRRAEVARLRELHTLKGHVESVVKLKGLDIETIQQHYTV

## >PHA7, PGSC0003DMP400060260

MGTEKTTTTLEAINNETVDLENIPIKEVFENLKCTEEGLNSAEVEKRLNVFGHNKLEEKKESKILKFLGFMWNPLSWVMEAAAIMALFLPHGKHKGVDYQDFVGIVALLIINSTISFMEENNAGNAAAALMARLAPKAKVLRDGKWNEEDAAVLVPGDIISIKLGDIIPADARLLNGDPLKIDQSALTGESLPVTKNPGDGVYSGSTCKQGEIEAVVIATGVHTFFGKAAHLVENTTHVGHFQKVLTSIGNFCICSIATGMIIELIVIFGGQHRHPREAVDSLLVLLIGGIPIAMPTVLSVTMAIGSHRLSQQGAITKRMTAIEEMAGMDVLCSDKTGTLTLNKLSVDKNLIEVFAKDVEKDMVVLMAARASRLENQDAIDTAIVSMLADPKEARAGITEVHFLPFNPTDKRTALTYLDSAGKMHRVSKGAPEQILNLAWNKSDIKNRVHSVIDKFAERGLRSLAVARQEVPEGTKDSPGGLWEFVGLLPLFDPPRHDSAETIRRALELGVSVKMITGDQLAIGKETGRRLGMGTNMYPSSFLLGEQKDSSAAVLPIEELIESADGFAGVFPEHKYEIVRILQSRKHICGMTGDGVNDAPALKKADIGIAVADATDAARGASDIVLTEPGLSVIIHAVLTSRAIFQRMKNYTIYAVSITIRIVLGFMLLTAFWKFNFPPFMVLVIAILNDGTIMTISKDRVKPSPLPDSWKLSEIFAIGIVLGSYLALMTALFFYLTFETSFFANAFHVTDFNKHIPENKVITDSLNAKLASAVYLQVSTISQALIFVTRSRGWSFMERPGLLLVVAFIVAQLVATFMSAMVTSVKFAGIEKIGWRWTGVIWLFNIITYFLLDPIKFAVRYALSGRAWGLLLNQKTAFTNRKDFGKEAREAAWAAEQRTIHGLQSVETKTFPENYTFREISVMAEEAKRRADIARLRELHTLKGKVESFAKLRGLDVDHVNPHYTV

# >A.thaliana

## >AHA1 AT2G18960

MSGLEDIKNETVDLEKIPIEEVFQQLKCTREGLTTQEGEDRIVIFGPNKLEEKKESKILKFLGFMWNPLSWVMEAAALMAIALANGDNRPPDWQDFVGIICLLVINSTISFIEENNAGNAAAALMAGLAPKTKVLRDGKWSEQEAAILVPGDIVSIKLGDIIPADARLLEGDPLKVDQSALTGESLPVTKHPGQEVFSGSTCKQGEIEAVVIATGVHTFFGKAAHLVDSTNQVGHFQKVLTSIGNFCICSIAIGIAIEIVVMYPIQHRKYRDGIDNLLVLLIGGIPIAMPTVLSVTMAIGSHRLSQQGAITKRMTAIEEMAGMDVLCSDKTGTLTLNKLSVDKNLVEVFCKGVEKDQVLLFAAMASRVENQDAIDAAMVGMLADPKEARAGIREVHFLPFNPVDKRTALTYIDSDGNWHRVSKGAPEQILDLANARPDLRKKVLSCIDKYAERGLRSLAVARQVVPEKTKESPGGPWEFVGLLPLFDPPRHDSAETIRRALNLGVNVKMITGDQLAIGKETGRRLGMGTNMYPSAALLGTDKDSNIASIPVEELIEKADGFAGVFPEHKYEIVKKLQERKHIVGMTGDGVNDAPALKKADIGIAVADATDAARGASDIVLTEPGLSVIISAVLTSRAIFQRMKNYTIYAVSITIRIVFGFMLIALIWEFDFSAFMVLIIAILNDGTIMTISKDRVKPSPTPDSWKLKEIFATGIVLGGYQAIMSVIFFWAAHKTDFFSDKFGVRSIRDNNDELMGAVYLQVSIISQALIFVTRSRSWSFVERPGALLMIAFVIAQLVATLIAVYADWTFAKVKGIGWGWAGVIWIYSIVTYFPQDILKFAIRYILSGKAWASLFDNRTAFTTKKDYGIGEREAQWAQAQRTLHGLQPKEDVNIFPEKGSYRELSEIAEQAKRRAEIARLRELHTLKGHVESVAKLKGLDIDTAGHHYTV

## >AHA2 AT4G30190

MSSLEDIKNETVDLEKIPIEEVFQQLKCSREGLTTQEGEDRIQIFGPNKLEEKKESKLLKFLGFMWNPLSWVMEMAAIMAIALANGDGRPPDWQDFVGIICLLVINSTISFIEENNAGNAAAALMAGLAPKTKVLRDGKWSEQEAAILVPGDIVSIKLGDIIPADARLLEGDPLKVDQSALTGESLPVTKHPGQEVFSGSTCKQGEIEAVVIATGVHTFFGKAAHLVDSTNQVGHFQKVLTAIGNFCICSIAIGMVIEIIVMYPIQRRKYRDGIDNLLVLLIGGIPIAMPTVLSVTMAIGSHRLSQQGAITKRMTAIEEMAGMDVLCSDKTGTLTLNKLSVDKNLVEVFCKGVEKDQVLLFAAMASRVENQDAIDAAMVGMLADPKEARAGIREVHFLPFNPVDKRTALTYIDGSGNWHRVSKGAPEQILELAKASNDLSKKVLSIIDKYAERGLRSLAVARQVVPEKTKESPGAPWEFVGLLPLFDPPRHDSAETIRRALNLGVNVKMITGDQLAIGKETGRRLGMGTNMYPSSALLGTHKDANLASIPVEELIEKADGFAGVFPGYNLLIYCLDYKPHYMFIAKVVMLVLSFVFFIAEHKYEIVKKLQERKHIVGMTGDGVNDAPALKKADIGIAVADATDAARGASDIVLTEPGLSVIISAVLTSRAIFQRMKNYTIYAVSITIRIVFGFMLIALIWEFDFSAFMVLIIAILNDGTIMTISKDRVKPSPTPDSWKLKEIFATGVVLGGYQAIMTVIFFWAAHKTDFFSDTFGVRSIRDNNHELMGAVYLQVSIISQALIFVTRSRSWSFVERPGALLMIAFLIAQLIATLIAVYANWEFAKIRGIGWGWAGVIWLYSIVTYFPLDVFKFAIRYILSGKAWLNLFENKTAFTMKKDYGKEEREAQWALAQRTLHGLQPKEAVNIFPEKGSYRELSEIAEQAKRRAEIARLRELHTLKGHVESVVKLKGLDIETPSHYTV

## >AHA3 AT5G57350

MASGLEDIVNENVDLEKIPIEEVFQQLKCSREGLSGAEGENRLQIFGPNKLEEKKESKLLKFLGFMWNPLSWVMEAAAIMAIALANGGGKPPDWQDFVGIVCLLVINSTISFVEENNAGNAAAALMAGLAPKTKVLRDGKWSEQEASILVPGDIVSIKLGDIIPADARLLEGDPLKVDQSALTGESLPATKGPGEEVFSGSTCKQGEIEAVVIATGVHTFFGKAAHLVDSTNQVGHFQKVLTAIGNFCICSIAVGIAIEIVVMYPIQRRHYRDGIDNLLVLLIGGIPIAMPTVLSVTMAIGSHKLSQQGAITKRMTAIEEMAGMDVLCSDKTGTLTLNKLSVDKNLIEVYCKGVEKDEVLLFAARASRVENQDAIDAAMVGMLADPKEARAGIREIHFLPFNPVDKRTALTFIDSNGNWHRVSKGAPEQILDLCNARADLRKRVHSTIDKYAERGLRSLAVSRQTVPEKTKESSGSPWEFVGVLPLFDPPRHDSAETIRRALDLGVNVKMITGDQLAIAKETGRRLGMGSNMYPSSSLLGKHKDEAMAHIPVEDLIEKADGFAGVFPEHKYEIVKKLQERKHICGMTGDGVNDAPALKKADIGIAVADATDAARGASDIVLTEPGLSVIISAVLTSRAIFQRMKNYTIYAVSITIRIVFGFMLIALIWKFDFSPFMVLIIAILNDGTIMTISKDRVKPSPTPDSWKLKEIFATGVVLGGYMAIMTVVFFWAAYKTDFFPRTFHVRDLRGSEHEMMSALYLQVSIVSQALIFVTRSRSWSFTERPGYFLLIAFWVAQLIATAIAVYGNWEFARIKGIGWGWAGVIWLYSIVFYFPLDIMKFAIRYILAGTAWKNIIDNRTAFTTKQNYGIEEREAQWAHAQRTLHGLQNTETANVVPERGGYRELSEIANQAKRRAEIARLRELHTLKGHVESVVKLKGLDIETAGHYTV

## >AHA4 AT3G47950

MTTTVEDNREVLEAVLKEAVDLENVPIEEVFENLRCSKEGLTTQAADERLALFGHNKLEEKKESKFLKFLGFMWNPLSWVMEAAAIMAIALANGGGKPPDWQDFVGIITLLVINSTISFIEENNAGNAAAALMARLAPKAKVLRDGRWGEQDAAILVPGDIISIKLGDIVPADARLLEGDPLKIDQSALTGESLPVTKSSGDGVYSGSTCKQGEIEAVVIATGVHTFFGKAAHLVDTTNQIGHFQQVLTAIGNFCICSIAVGMLIEIVVMYPIQHRAYRPGIDNLLVLLIGGIPIAMPTVLSVTMAIGSHRLSQQGAITKRMTAIEEMAGMDVLCSDKTGTLTLNKLTVDKNLIEVFMKGVDADTVVLMAARASRLENQDAIDAAIVGMLADPKDARAGIQEVHFLPFNPTDKRTALTYIDNEGNTHRVSKGAPEQILNLAHNKSEIERRVHAVIDKFAERGLRSLAVAYQDVPEGRKDSAGGPWQFVGLMPLFDPPRHDSAETIRRALNLGVSVKMITGDQLAIGKETGRRLGMGTNMYPSSALLGQNKDESIVALPVDELIEKADGFAGVFPEHKYEIVKRLQARKHICGMTGDGVNDAPALKKADIGIAVADATDAARSASDIVLTEPGLSVIISAVLTSRAIFQRMKNYTIYAVSITIRIVLGFMLLALIWQFDFPPFMVLIIAILNDGTIMTISKDRVKPSPLPDSWKLSEIFATGVVFGSYMAMMTVIFFWVSYKTDFFPRTFGVATLEKTAHDDFRKLASAIYLQVSIISQALIFVTRSRSWSFVERPGIFLMIAFILAQLVATLIAVYANWSFAAIEGIGWGWAGVIWLYNIIFYIPLDFIKFFIRYALSGRAWDLVIEQRVAFTRQKDFGKEQRELQWAHAQRTLHGLQAPDTKMFTDRTHVSELNQMAEEAKRRAEIARLRELHTLKGHVESVVRLKGLDIETIQQAYTV

## >AHA5 AT2G24520

MEEVFEELKCTKQGLTANEASHRLDVFGPNKLEEKKESKLLKFLGFMWNPLSWVMEVAALMAIALANGGGRPPDWQDFVGIVCLLLINSTISFIEENNAGNAAAALMAGLAPKTKVLRDNQWSEQEASILVPGDVISIKLGDIIPADARLLDGDPLKIDQSSLTGESIPVTKNPSDEVFSGSICKQGEIEAIVIATGVHTFFGKAAHLVDNTNQIGHFQKVLTSIGNFCICSIALGIIVELLVMYPIQRRRYRDGIDNLLVLLIGGIPIAMPSVLSVTMATGSHRLFQQGAITKRMTAIEEMAGMDVLCCDKTGTLTLNKLTVDKNLVEVFAKGVGKEHVFLLAARASRIENQDAIDAAIVGMLADPKEARAGVREVHFFPFNPVDKRTALTYVDSDGNWHRASKGAPEQILNLCNCKEDVRRKVHGVIDKFAERGLRSLAVARQEVLEKKKDAPGGPWQLVGLLPLFDPPRHDSAETIRRALNLGVNVKMITGDQLAIGKETGRRLGMGTNMYPSSALLGQVKDSSLGALPVDELIEKADGFAGVFPEHKYEIVHRLQQRNHICGMTGDGVNDAPALKKADIGIAVVDATDAARGASDIVLTEPGLSVIISAVLTSRAIFQRMKNYTIYAVSITIRIVFGFMFIALIWQFDFSPFMVLIIAILNDGTIMTISKDRMKPSPQPDSWKLRDIFSTGVVLGGYQALMTVVFFWVMKDSDFFSNYFGVRPLSQRPEQMMAALYLQVSIISQALIFVTRSRSWSYAECPGLLLLGAFVIAQLVATFIAVYANWSFARIEGAGWGWAGVIWLYSFLTYIPLDLLKFGIRYVLSGKAWLNLLENKTAFTTKKDYGKEEREAQWAAAQRTLHGLQPAEKNNIFNEKNSYSELSQIAEQAKRRAEVVRLREINTLKGHVESVVKLKGLDIDTIQQHYTV

## >AHA6 AT2G07560

MAADISWDEIKKENVDLEKIPVDEVFQQLKCSREGLSSEEGRNRLQIFGANKLEEKVENKFLKFLGFMWNPLSWVMEAAAIMAIVLANGGGRPPDWQDFVGITCLLIINSTISFIEENNAGNAAAALMANLAPKTKVLRDGRWGEQEAAILVPGDLISIKLGDIVPADARLLEGDPLKIDQSALTGESLPATKHQGDEVFSGSTCKQGEIEAVVIATGVHTFFGKAAHLVDSTNNVGHFQKVLTAIGNFCICSIGIGMLIEIIIMYPIQHRKYRDGIDNLLVLLIGGIPIAMPTVLSVTMAIGSHRLSQQGAITKRMTAIEEMAGMDVLCSDKTGTLTLNKLTVDKNLIEVFSKDVDKDYVILLSARASRVENQDAIDTSIVNMLGDPKEARAGITEVHFLPFNPVEKRTAITYIDTNGEWHRCSKGAPEQIIELCDLKGETKRRAHEIIDKFAERGLRSLGVARQRVPEKDKESAGTPWEFVGLLPLFDPPRHDSAETIRRALDLGVNVKMITGDQLAIGKETGRRLGMGTNMYPSSSLLENKDDTTGGVPVDELIEKADGFAGVFPEHKYEIVRKLQERKHIVGMTGDGVNDAPALKKADIGIAVDDATDAARSASDIVLTEPGLSVIVSAVLTSRAIFQRMKNYTIYAVSITIRIVLGFMLVALIWEFDFSPFMVLIIAILNDGTIMTISKDRVKPSPIPDSWKLKEIFATGVVLGTYMALVTVVFFWLAHDTTFFSDKFGVRSLQGKDEELIAVLYLQVSIISQALIFVTRSRSWSFVERPGLLLLIAFFVAQLIATLIATYAHWEFARIKGCGWGWCGVIWIYSIVTYIPLDILKFITRYTLSGKAWNNMIENRTAFTTKKDYGRGEREAQWALAQRTLHGLKPPESMFEDTATYTELSEIAEQAKKRAEVARLREVHTLKGHVESVVKLKGLDIDNLNQHYTV

## >AHA7 AT3G60330

MTDIEALKAITTESIDLENVPVEEVFQHLKCTKEGLTSNEVQERLTLFGYNKLEEKKESKILKFLGFMWNPLSWVMEAAALMAIGLAHGGGKPADYHDFVGIVVLLLINSTISFVEENNAGNAAAALMAQLAPKAKAVRDGKWNEIDAAELVPGDIVSIKLGDIIPADARLLEGDPLKIDQATLTGESLPVTKNPGASVYSGSTCKQGEIEAVVIATGVHTFFGKAAHLVDSTTHVGHFQKVLTAIGNFCICSIAVGMAIEIVVIYGLQKRGYRVGIDNLLVLLIGGIPIAMPTVLSVTMAIGAHRLAQQGAITKRMTAIEEMAGMDVLCSDKTGTLTLNKLSVDKNLIEVFKRGIDRDMAVLMAARAARLENQDAIDTAIVSMLSDPKEARAGIKELHFLPFSPANRRTALTYLDGEGKMHRVSKGAPEEILDMAHNKLEIKEKVHATIDKFAERGLRSLGLAYQEVPDGDVKGEGGPWDFVALLPLFDPPRHDSAQTIERALHLGVSVKMITGDQLAIAKETGRRLGMGTNMYPSSSLLSDNNTEGVSVDELIENADGFAGVFPEHKYEIVKRLQSRKHICGMTGDGVNDAPALKKADIGIAVDDATDAARGASDIVLTEPGLSVIISAVLTSRAIFQRMKNYTIYAVSITIRIVMGFMLLCVFWEFDFPPFMVLVIAILNDGTIMTISKDRVKPSPTPDCWKLKEIFATGVVLGAYLAIMTVVFFWAAYETNFFHNIFHVRNFNQHHFKMKDKKVAAHLNEQMASAVYLQVSTISQALIFVTRSRSWSFVERPGFLLVIAFLIAQLVASVISAMANWPFAGIRSIGWGWTGVIWIFNIVTYMLLDPIKFLVRYALSGKSWDRMVEGRTALTGKKNFGQEERMAAWATEKRTQHGLETGQKPVYERNSATELNNMAEEAKRRAEIARMRELQTLKGKVESAAKLKGYDLEDPNSNNYTI

## >AHA8 AT3G42640

MATEFSWDEIKKENVDLERIPVEEVFEQLKCSKEGLSSDEGAKRLEIFGANKLEEKSENKFLKFLGFMWNPLSWVMESAAIMAIVLANGGGKAPDWQDFIGIMVLLIINSTISFIEENNAGNAAAALMANLAPKTKVLRDGKWGEQEASILVPGDLISIKLGDIVPADARLLEGDPLKIDQSALTGESLPTTKHPGDEVFSGSTCKQGEIEAVVIATGVHTFFGKAAHLVDSTNNVGHFQKVLTSIGNFCICSIGLGMLIEILIMYPIQHRTYRDGIDNLLVLLIGGIPIAMPTVLSVTMAIGSHRLSQQGAITKRMTAIEEMAGMDVLCSDKTGTLTLNKLSVDKSLIEVFPKNMDSDSVVLMAARASRIENQDAIDASIVGMLGDPKEARAGITEVHFLPFNPVDKRTAITYIDESGDWHRSSKGAPEQIIELCNLQGETKRKAHEVIDGFAERGLRSLGVAQQTVPEKTKESDGSPWEFVGLLPLFDPPRHDSAETIRRALELGVNVKMITGDQLAIGIETGRRLGMGTNMYPSTSLLGNSKDESLVGIPIDELIEKADGFAGVFPEHKYEIVKKLQERKHICGMTGDGVNDAPALKKADIGIAVADATDAARSASDIVLTEPGLSVIISAVLTSRAIFQRMKNYTIYAVSITIRIVLGFMLVALIWRFDFAPFMVLIIAILNDGTIMTISKDRVKPSPVPDSWKLNEIFATGVVLGTYMALTTVLFFWLAHDTDFFSKTFGVRSIQGNEEELMAALYLQVSIISQALIFVTRSRSWSFVERPGFLLLIAFVIAQLVATLIAVYANWGFARIVGCGWGWAGGIWVYSIITYIPLDILKFIIRYALTGKAWDNMINQKTAFTTKKDYGKGEREAQWALAQRTLHGLPPPEAMFNDNKNELSEIAEQAKRRAEVARLRELHTLKGHVESVVKLKGLDIDTIQQHYTV

## >AHA9 AT1G80660

MAGNKDSSWDDIKNEGIDLEKIPIEEVLTQLRCTREGLTSDEGQTRLEIFGPNKLEEKKENKVLKFLGFMWNPLSWVMELAAIMAIALANGGGRPPDWQDFVGITVLLIINSTISFIEENNAGNAAAALMAGLAPKTKVLRDGKWSEQEAAILVPGDIISIKLGDIVPADGRLLDGDPLKIDQSALTGESLPVTKHPGQEVYSGSTCKQGELEAVVIATGVHTFFGKAAHLVDSTNQEGHFQKVLTAIGNFCICSIAIGMLIEIVVMYPIQKRAYRDGIDNLLVLLIGGIPIAMPTVLSVTMAIGSHRLSQQGAITKRMTAIEEMAGMDVLCSDKTGTLTLNKLTVDKSMVEVFVKDLDKDQLLVNAARASRVENQDAIDACIVGMLGDPREAREGITEVHFFPFNPVDKRTAITYIDANGNWHRVSKGAPEQIIELCNLREDASKRAHDIIDKFADRGLRSLAVGRQTVSEKDKNSPGEPWQFLGLLPLFDPPRHDSAETIRRALDLGVNVKMITGDQLAIGKETGRRLGMGTNMYPSSALLGQDKDESIASLPVDELIEKADGFAGVFPEHKYEIVKRLQEMKHICGMTGDGVNDAPALKRADIGIAVADATDAARSASDIVLTEPGLSVIVSAVLTSRAIFQRMKNYTIYAVSITIRIVMGFMLLALIWKFDFSPFMVLIVAILNDGTIMTISKDRVKPSPLPDSWKLKEIFATGVVLGTYLAVMTVVFFWAAESTDFFSAKFGVRSISGNPHELTAAVYLQVSIVSQALIFVTRSRSWSYVERPGFWLISAFFMAQLIATLIAVYANWNFARIRGIGWGWAGVIWLYSIVFYIPLDILKFIIRYSLSGRAWDNVIENKTAFTSKKDYGKGEREAQWAQAQRTLHGLQPAQTSDMFNDKSTYRELSEIADQAKRRAEVARLRERHTLKGHVESVVKQKGLDIEAIQQHYTL

## >AHA10 AT1G17260

MAEDLDKPLLDPDTFNRKGIDLGILPLEEVFEYLRTSPQGLLSGDAEERLKIFGPNRLEEKQENRFVKFLGFMWNPLSWVMEAAALMAIALANSQSLGPDWEDFTGIVCLLLINATISFFEENNAGNAAAALMARLALKTRVLRDGQWQEQDASILVPGDIISIKLGDIIPADARLLEGDPLKIDQSVLTGESLPVTKKKGEQVFSGSTCKQGEIEAVVIATGSTTFFGKTARLVDSTDVTGHFQQVLTSIGNFCICSIAVGMVLEIIIMFPVQHRSYRIGINNLLVLLIGGIPIAMPTVLSVTLAIGSHRLSQQGAITKRMTAIEEMAGMDVLCCDKTGTLTLNSLTVDKNLIEVFVDYMDKDTILLLAGRASRLENQDAIDAAIVSMLADPREARANIREIHFLPFNPVDKRTAITYIDSDGKWYRATKGAPEQVLNLCQQKNEIAQRVYAIIDRFAEKGLRSLAVAYQEIPEKSNNSPGGPWRFCGLLPLFDPPRHDSGETILRALSLGVCVKMITGDQLAIAKETGRRLGMGTNMYPSSSLLGHNNDEHEAIPVDELIEMADGFAGVFPEHKYEIVKILQEMKHVVGMTGDGVNDAPALKKADIGIAVADATDAARSSADIVLTDPGLSVIISAVLTSRAIFQRMRNYTVYAVSITIRIVLGFTLLALIWEYDFPPFMVLIIAILNDGTIMTISKDRVRPSPTPESWKLNQIFATGIVIGTYLALVTVLFYWIIVSTTFFEKHFHVKSIANNSEQVSSAMYLQVSIISQALIFVTRSRGWSFFERPGTLLIFAFILAQLAATLIAVYANISFAKITGIGWRWAGVIWLYSLIFYIPLDVIKFVFHYALSGEAWNLVLDRKTAFTYKKDYGKDDGSPNVTISQRSRSAEELRGSRSRASWIAEQTRRRAEIARLLEVHSVSRHLESVIKLKQIDQRMIRAAHTV

## >AHA11 AT5G62670

MGDKEEVLEAVLKETVDLENVPIEEVFESLRCSREGLTTEAADERLALFGHNKLEEKKESKFLKFLGFMWNPLSWVMEAAAIMAIALANGGGKPPDWQDFVGIITLLVINSTISFIEENNAGNAAAALMARLAPKAKVLRDGRWGEQDAAILVPGDIISIKLGDIVPADARLLEGDPLKIDQSSLTGESLPVTKGPGDGVYSGSTCKQGELEAVVIATGVHTFFGKAAHLVDTTNHVGHFQQVLTAIGNFCICSIAVGMIIEIVVMYPIQHRAYRPGIDNLLVLLIGGIPIAMPTVLSVTMAIGSHRLSQQGAITKRMTAIEEMAGMDVLCSDKTGTLTLNKLTVDKNLIEVFTKGVDADTVVLMAAQASRLENQDAIDAAIVGMLADPKEARAGVREVHFLPFNPTDKRTALTYIDSDGKMHRVSKGAPEQILNLAHNRAEIERRVHAVIDKFAERGLRSLAVAYQEVPEGTKESAGGPWQFMGLMPLFDPPRHDSAETIRRALNLGVNVKMITGDQLAIGKETGRRLGMGTNMYPSSALLGQHKDESIGALPIDDLIEKADGFAGVFPEHKYEIVKRLQARKHICGMTGDGVNDAPALKKADIGIAVADATDAARSASDIVLTEPGLSVIISAVLTSRAIFQRMKNYTIYAVSITIRIVLGFMLLALIWKFDFPPFMVLIIAILNDGTIMTISKDRVKPSPLPDSWKLSEIFATGVVFGSYMAMMTVIFFWAAYKTDFFPRTFGVSTLEKTAHDDFRKLASAIYLQVSIISQALIFVTRSRSWSYVERPGMLLVVAFILAQLVATLIAVYANWSFAAIEGIGWGWAGVIWLYNIVFYIPLDIIKFLIRYALSGRAWDLVIEQRVAFTRQKDFGKEQRELQWAHAQRTLHGLQAPDAKMFPERTHFNELSQMAEEAKRRAEIARLRELHTLKGHVESVVRLKGLDIETIQQAYTV
