# Supplementary material for: Genome-Wide Identification and Analysis of P-Type Plasma Membrane H+-ATPase Sub-Gene Family in Sunflower and the Role of HHA4 and HHA11 in the Development of Salt Stress Resistance
Source: Genes (Basel). 2020 Mar 27;11(4):361. doi: 10.3390/genes11040361 (PMC7231311; doi:10.3390/genes11040361)
Supplement: Supplementary file 1 [file genes-11-00361-s001.zip › Supplementary File 6.pdf]

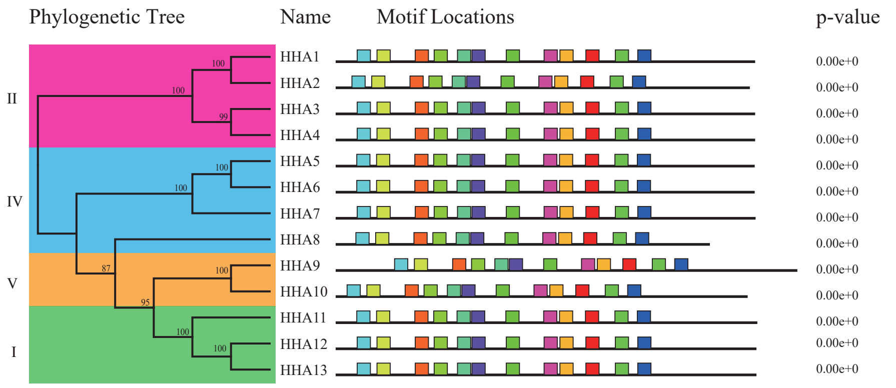

Motif	Symbol	Motif Consensus
1.		FPEHKYEIVKKLQERKHICGMTGDGVNDAP
2.		FGPNKLEEKKESKILKFLGFMWNPLSWVME
3.		FGKAAHLVDSTNQVGHFQKVLTAIGNFCIC
4.		QQGAITKRMTAIEEMAGMDVLCSDKTGTLT
5.		NVKMITGDQLAIGKETGRRLGMGTNMYPSS
6.		VLTSRAIFQRMKNYTIYAVSITIRIVLGFM
7.		NDGTIMTISKDRVKPSPLPDSWKLKEIFAT
8.		KESPGGPWEFVGLLPLFDPPRHDSAETIRR
9.		DQSALTGESLPVTKNPGDGVYSGSTCKQGE
10.		PPDWQDFVGIVTLLVINSTISFIEENNAGN
11.		DPKEARAGIREVHFLPFNPVDKRTALTYID
12.		GIDNLLVLLIGGIPIAMPTVLSVTMAIGSH
