# Supplementary figures and images for: Genome-Wide Identification and Analysis of P-Type Plasma Membrane H+-ATPase Sub-Gene Family in Sunflower and the Role of HHA4 and HHA11 in the Development of Salt Stress Resistance
Source: Genes (Basel). 2020 Mar 27;11(4):361. doi: 10.3390/genes11040361 (PMC7231311; doi:10.3390/genes11040361)

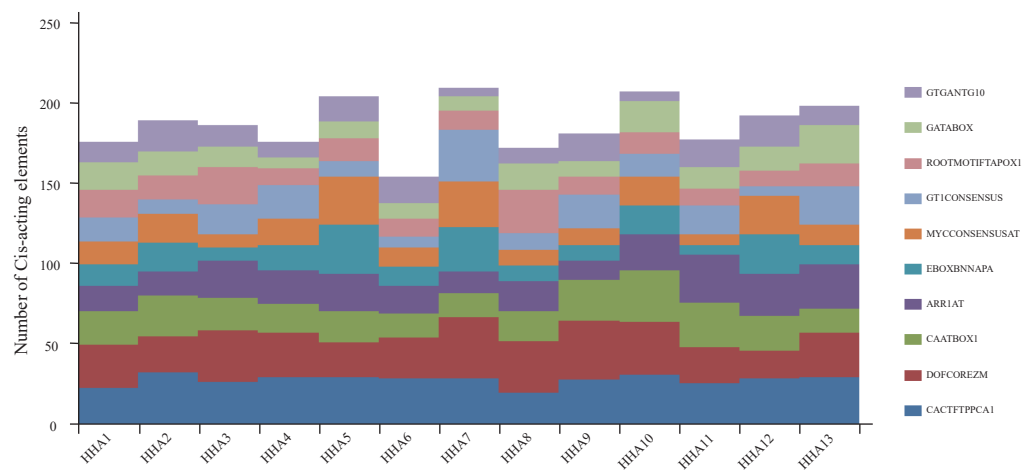

Supplement: Supplementary file 1 [file genes-11-00361-s001.zip › Supplementary File 8.pdf]

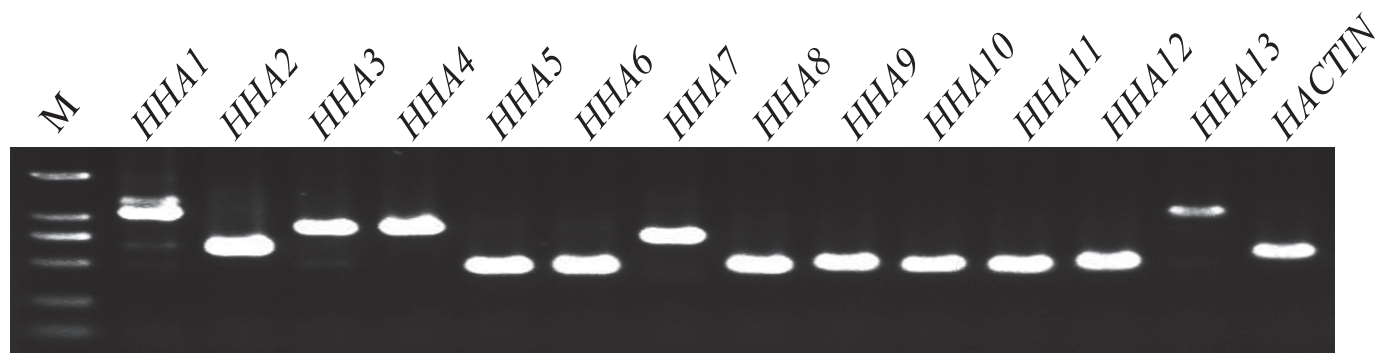

Supplement: Supplementary file 1 [file genes-11-00361-s001.zip › Supplementary File 9.pdf]
